# Rational Design of Palladium(II) Indenyl and Allyl Complexes Bearing Phosphine and Isocyanide Ancillary Ligands with Promising Antitumor Activity

**DOI:** 10.3390/molecules29020345

**Published:** 2024-01-10

**Authors:** Enrica Bortolamiol, Eleonora Botter, Enrico Cavarzerani, Matteo Mauceri, Nicola Demitri, Flavio Rizzolio, Fabiano Visentin, Thomas Scattolin

**Affiliations:** 1 Department of Molecular Sciences and Nanosystems, Università Ca’ Foscari, Campus Scientifico, Via Torino 155, 30174 Venezia, Italy; enrica.bortolamiol@unive.it (E.B.); eleonora.botter@unive.it (E.B.); enrico.cavarzerani@unive.it (E.C.); matteo.mauceri@unive.it (M.M.); flavio.rizzolio@unive.it (F.R.); 2 Elettra-Sincrotrone Trieste, Area Science Park, S.S. 14 Km 163.5 Basovizza, 34149 Trieste, Italy; nicola.demitri@elettra.eu; 3 Pathology Unit, Department of Molecular Biology and Translational Research, Centro di Riferimento Oncologico di Aviano (CRO), Istituto di Ricovero e Cura a Carattere Scientifico (IRCCS), Via Franco Gallini 2, 33081 Aviano, Italy; 4 Dipartimento di Scienze Chimiche, Università Degli Studi di Padova, Via Marzolo 1, 35131 Padova, Italy

**Keywords:** palladium indenyl complexes, phosphine ligands, isocyanide ligands, metallodrugs, antitumor activity, ovarian cancer

## Abstract

A new class of palladium–indenyl complexes characterized by the presence of one bulky alkyl isocyanide and one aryl phosphine serving as ancillary ligands has been prepared, presenting high yields and selectivity. All the new products were completely characterized using spectroscopic and spectrometric techniques (NMR, FT-IR, and HRMS), and, for most of them, it was also possible to define their solid-state structures via X-ray diffractometry, revealing that the indenyl fragment always binds to the metal centre with a hapticity intermediate between ƞ^3^ and ƞ^5^. A reactivity study carried out using piperidine as a nucleophilic agent proved that the indenyl moiety is the eligible site of attack rather than the isocyanide ligand or the metal centre. All complexes were tested as potential anticancer agents against three ovarian cancer cell lines (A2780, A2780*cis*, and OVCAR-5) and one breast cancer cell line (MDA-MB-231), displaying comparable activity with respect to cisplatin, which was used as a positive control. Moreover, the similar cytotoxicity observed towards A2780 and A2780*cis* cells (cisplatin-sensitive and cisplatin-resistant, respectively) suggests that our palladium derivatives presumably act with a mechanism of action different than that of the clinically approved platinum drugs. For comparison, we also synthesized Pd-ƞ^3^-allyl derivatives, which generally showed a slightly higher activity towards ovarian cancer cells and lower activity towards breast cancer cells with respect to their Pd-indenyl congeners.

## 1. Introduction

Metallodrugs are a class of therapeutic compounds that incorporate metal ions as essential components, leveraging the unique properties of metals for medicinal purposes [1,2,3]. These compounds often exhibit promising potential in the treatment of various diseases, including cancer, due to their ability to interact with biological targets in ways distinct from traditional organic drugs [4,5].

Despite their great potential, there are currently no general rules or efficient computational methods that can be used to predict which metal complexes are of relevant biological interest.

In fact, unlike organic compounds, most metal complexes with pharmacological properties react in a biological environment long before performing their function. For example, they can dissociate one or more ligands in aqueous media (a hydrolysis reaction) or react with biomolecules commonly present in the blood or cytoplasm (a ligand exchange reaction) [6]. Other processes that a metal complex can undergo in a biological environment are, for example, the reduction/oxidation of its metal centre, an electrophilic/nucleophilic attack on a coordinated ligand, and the change in the hapticity of polydentate/polyhapto ligands anchored to the metal centre. All these options render the detailed study of the mechanism of action of a metallodrug extremely difficult, if not impossible [7]. In fact, the typical reactivity of organometallic and coordination compounds can lead to a wide range of different complexes within a biological environment (metal speciation). Each of these species can have a specific biological activity, a preferential form of accumulation in cellular organelles (e.g., cationic species are usually located in the mitochondria), and one or more biological/molecular targets.

It is therefore difficult to consider metallodrugs as selective compounds for a given biotarget, thus making it complicated to effectively use approaches based on molecule–biotarget interaction such as molecular docking. It is definitely more intellectually honest to describe metallodrugs as multi-target molecules in which only the starting molecular structure and the observable therapeutic effects are known with certainty [5,6,7].

For this reason, the most effective approach to the search for potential metallodrugs remains the in vitro screening of new metal complexes, modulating their characteristics by acting on the nature of both the metal centre and ligands.

In particular, a potent tool allowing chemists to modulate the features of an organometallic compound lies in the targeted choice of ancillary ligands, which influences the electronic density on the metal centre, the “shape” of the complex, and the regio/stereochemistry of the attack of an external agent (e.g., a nucleophile or an electrophile) on the organic fragment [8,9]. In this regard, a profitable option is the simultaneous use of different ancillary ligands, seeking to take advantage of their peculiar features in a cooperative manner.

Following this strategy, in this work, we focused our attention on Pd(II)-indenyl and Pd(II)-allyl complexes bearing one phosphine and one isocyanide serving as ancillary ligands.

Phosphines are ubiquitous ligands in palladium complexes, especially when the latter are employed as catalysts. The steric and electronic properties of phosphines have been parameterized in great detail, taking advantage of various spectroscopic or theoretical methods [10,11,12,13,14].

Alternatively, the use of isocyanides is more problematic due to their possible reactivity after coordination on a metal centre (the carbon and nitrogen of a Pd(II)-coordinated isocyanide may be subjected to nucleophilic [15] or electrophilic [16] attack, respectively). This aspect makes them sometimes behave as actor ligands and must always be considered [17]. In any case, when employed as spectator ligands, isocyanides generally have a better capability to promote π-back donation than phosphines, although it should be remembered that this ability strictly depends on the nature of the nitrogen substituent. A few interesting applications of palladium-isocyanide catalysts have been reported: complexes of the type [Pd(CNR)_2_] are able to catalyse the bis-silylation of alkynes, whereas [PdCl_2_(CNR)_2_] derivatives promote Suzuki–Miyaura cross-coupling [18]. For both processes, a high steric demand is required for the coordinated isocyanides, as this sort of steric protection ensures higher stability of the catalysts. Interestingly, bulky tertiary alkyl isocyanides are also particularly stable under physiological conditions, showing a high resistance to the most common cellular and extracellular nucleophiles (e.g., glutathione, *N*-acetyl cysteine, and mercaptoethylamine) and a low tendency to undergo oxidation in the presence of NADPH [19]. These favourable aspects have allowed the planning of their utilisation in promising biorthogonal reactions coupled with chlorooximes [20], and, in a specific way, 1-adamantyl isocyanide (Adic) demonstrated antiviral properties (against influenza virus H5N1) that were 10 times greater than those of its isostructural precursor amantadine [21].

To date, no study has been reported in the literature about the biological properties of palladium complexes equipped with tertiary alkyl isocyanides [22,23,24,25,26,27,28,29,30], and our choice to test the anticancer activity of Pd(II)-indenyl and Pd(II)-allyl complexes supported by these kinds of ligands is intended to begin filling this gap.

Some of our recent papers have already proven the effectiveness of these types of organometallic derivatives, especially when phosphines are employed as ancillary ligands [31,32,33]. The choice of such organopalladium fragments was initially based on their well-known reactivity and importance in numerous cross-coupling processes [34,35,36,37,38,39,40,41,42,43] and, at the same time, on the absence of contributions regarding their potential biological activity.

Notably, it has recently been demonstrated that the hapticity of the Pd-indenyl fragment is generally intermediate between η^3^ and η^5^, which leads to a lower reactivity towards nucleophilic agents such as secondary amines [31]. This evidence appears to be in close correlation with the antiproliferative activity data, which show a lower cytotoxicity of Pd-indenyl complexes compared to their Pd-allyl congeners [31,32,33].

Based on all this information, in this contribution, we propose to examine the effect of the introduction of one alkyl isocyanide in the coordination sphere of palladium. Our previous studies have proven that the simultaneous presence of one phosphine and one isocyanide on the palladium(II) centre assures higher stability of the complexes owing to the synergistic effect obtained by combining a σ-electron-donor ligand such as an aryl phosphine with a potential π-acceptor ligand such as an alkyl isocyanide [44].

Therefore, the main goal of this work was the preparation of a new class of organopalladium compounds characterised by a Pd-indenyl fragment and the simultaneous presence of one aryl phosphine and one alkyl isocyanide serving as ancillary ligands. The antitumor properties of these new complexes were assessed with respect to three different ovarian cancer lines and one breast cancer cell line. Moreover, cytotoxicity was assessed with respect to non-tumoral cells. Furthermore, the difference between the biological behaviour of Pd-indenyl derivatives and their Pd-allyl congeners was evaluated.

## 2. Results

### 2.1. Synthesis and Characterization of the Complexes

**Scheme 1:**
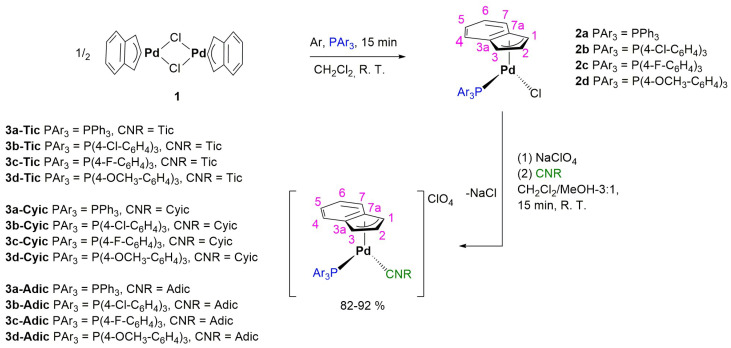
General procedure for the synthesis of Pd(II)-indenyl complexes bearing aryl phosphines and bulky alkyl isocyanides.

The synthetic approach that was followed for the preparation of novel Pd(II)-indenyl complexes equipped with one phosphine and one isocyanide was divided into two stages, as depicted in Scheme 1.

In the first step, starting from a dichloromethane solution of [Pd(Ind)(μ-Cl)]_2_ (**1**) dimeric precursor [45], the neutral intermediate species [Pd(Ind)(Cl)(PAr_3_)] (**2a**–**d**) were obtained by adding a sub-stoichiometric amount of the selected phosphine at room temperature. It is well-established that the slight stoichiometric defect of phosphine prevents the cascade of collateral reactions that lead to the two by-products [Pd_2_(PAr_3_)_2_(μ,η^3^-Ind)(μ-Cl)] and [Pd(PAr_3_)_2_Cl_2_], whose process of formation was previously described in detail in our earlier work and in that of other groups [31,32,46]. This can happen because [Pd(Ind)(μ-Cl)]_2_ might not be perfectly pure if not freshly prepared. Notably, the two by-products, when formed, are hard to eliminate from the reaction mixture.

In the second step, in which the isolation of the [Pd(Ind)(Cl)(PAr_3_)] intermediate is not required, a methanol solution of NaClO_4_ (1.5 equiv.) and two equivalents of the chosen alkyl isocyanide were added in rapid succession. This process induces the formation of the target cationic products [Pd(Ind)(CNR))(PAr_3_)]^+^, promoted by the massive precipitation of NaCl, which, to be optimal, requires a 3:1 ratio between dichloromethane and methanol in the reaction mixture.

This approach, developed by our research group, is an alternative to the use of silver salts as dechlorinating agents, which are more expensive and whose residues could be sources of interference in biological tests. After the easy filtration of NaCl (and the excess NaClO_4_), the final products, [Pd(Ind)(CNR)(PAr_3_)]ClO_4_ (**3a**–**d-Tic, 3a**–**d-Cyic** and **3a**–**d-Adic**), can be smoothly precipitated through the addition of diethyl ether or *n*-pentane.

The need to coordinate the phosphine first and, only in a subsequent step, the isocyanide ligand of interest has already been explained in a previous work by Zargarian and co-workers [45]. Indeed, the addition of isocyanide to the [Pd(Ind)(μ-Cl)]_2_ dimer results in the formation of [Pd(ƞ^1^-Ind)(CNR)(μ-Cl)]_2_. This undesirable species does not allow the formation of the complexes that are the object of this work through the simple addition of a phosphine ligand. Moreover, it is important to underline that isocyanides can also promote insertion side reactions, as demonstrated by us in previous contributions dealing with palladium allyl complexes [47]. This insertion takes place only on neutral complexes, whereas it is not possible on palladium cationic substrates such as those obtained via treating [Pd(Ind)(Cl)(PAr_3_)] with excess NaClO_4_.

Adopting the synthetic protocol represented in Scheme 1, twelve new compounds were prepared, combining four different *para*-substituted aryl phosphines (triphenylphosphine, tris(4-chlorophenyl)phosphine, tris(4-fluorophenyl)phosphine, and tris(4-methoxyphenyl)phosphine) and three distinct bulky alkyl isocyanides (Tic = *tert*-butyl isocyanide; Cyic = cyclohexyl isocyanide; and Adic = 1-adamantyl isocyanide). The choice of the four aryl phosphines was dictated by the stability and cytotoxicity that they can confer to Pd-indenyl complexes [31,32]. Moreover, in this manner, it is possible to evaluate the influence of the four different *para*-substituents on the biological activity of the corresponding complexes. Likewise, as stated above, the presence of bulky alkyl isocyanides should also contribute to enhancing the stability of the metal complexes. Moreover, some of these ligands have shown intrinsic biological activity [21].

A significant contribution to the identification of the final complexes was provided by the analysis of their NMR spectra (see Figures S1–S39). In the ^1^H NMR spectra, all seven distinct signals ascribable to the indenyl moiety are traceable, revealing the maintained integrity of this organic fragment. Moreover, they indirectly testify to the presence of two different ancillary ligands in the palladium coordination sphere. Among indenyl protons, H^3^ lies at the lowest chemical shift (between 5.2 and 5.4 ppm), probably due to the shielding effect of the aromatic rings of the adjacent phosphine ligand, as well as the H^4^ proton belonging to the six-membered ring (with δ between 6.2 and 6.5 ppm). In contrast, the H^1^ proton generates a more downfield signal (around 6.9–7.2 ppm) due to the *trans* influence of the phosphine ligand, and it is often close to or partially overlapping the H^5^ signal. H^2^, the third proton of the five-membered ring falls invariably between 6.6 and 6.9 ppm, while the remaining indenyl protons, H^6^ and H^7^, resonate over 7 ppm, often overlapping the signals of the aromatic protons of the phosphine ligand. Finally, the aliphatic zone of the spectra is characterised by the presence of signals ascribable to the isocyanide alkyl substituents. 

**Figure 1:**
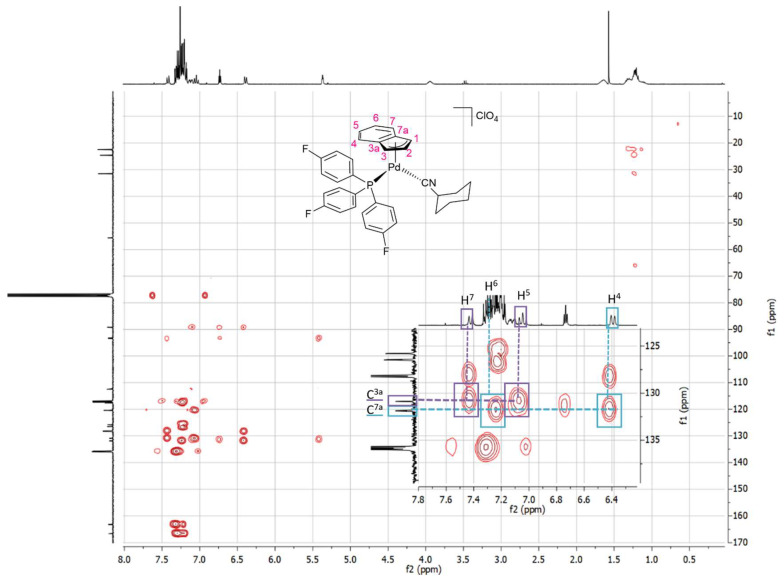
HMBC spectrum of complex **3c-Cyic** in CDCl_3_ at 298 K.

Other important confirmations of the identity of the products can be obtained from the ^13^C{^1^H} NMR spectra, where the expected nine signals of the indenyl moiety can be detected and assigned with the support of HMQC and HMBC bidimensional spectra. Among them, C^1^ and C^3^, which directly interact with the metal centre, resonate in the narrow ranges of 92–94 and 88–90 ppm, respectively, always taking the form of doublets for coupling with the adjacent phosphorus. The different values of the coupling constants allow the distinguishment of the carbon in the *trans* position with respect to the phosphine (C^1^ with J_C-P_ ≈ 19 Hz) and the carbon *trans* to the isocyanide (C^3^ with J_C-P_ ≈ 4 Hz). In addition, the C^2^ signal is a doublet regularly localised at 110–113 ppm. The assignment of the chemical shifts of the ring-junction carbons C^3a^ and C^7a^ is particularly important for the determination of the hapticity of the indenyl fragment in these types of complexes (vide infra). C^3a^ carbon is always recognizable thanks to the cross-peaks with H^7^ and H^5^ protons in HMBC spectra, while C^7a^ carbon shows coupling with H^6^ and H^4^ protons (see Figure 1).

Regarding the signals belonging to the phosphine ligand, it must be said that, with the exception of the complexes **3c-Tic, 3c-Cyic,** and **3c-Adic**, for which we must also consider coupling with fluorine, they always appear as doublets, with J_C-P_ constants decreasing as the number of bonds interposed among the selected carbon and phosphorus increase (i.e., for the *ipso* carbons, ^2^J_C-P_ = 45–55 Hz, whereas for the *para* carbons, ^4^J_C-P_ = 2.5–3.0 Hz). The chemical shifts of the aromatic carbons are not particularly different in the examined phosphines, with the only notable exception being the *para*-carbons, which are heavily influenced by the nature of the *para* substituent.

The ^31^P{^1^H} NMR spectra of the complexes show only one signal resonating at about 20–30 ppm higher than that of the corresponding free phosphine, confirming the uniqueness of the products obtained and the coordination of the phosphine with the metal centre. In the case of complex **3c**, which contain tris(4-fluorophenyl)phosphine, the signal appears as a quartet (J_P-F_ = 2.5 Hz) due to coupling with the three fluorine nuclei. Consistently, in the ^19^F{^1^H} NMR spectra, the only signals present at about −105 ppm are doublets with the same coupling constants.

The impossibility of tracing the very weak signal of coordinated isocyanide carbon in the ^13^C{^1^H} NMR spectra enhances the importance of the IR spectra of the synthesized complexes (Figures S40–S42). In fact, a very intense peak is always observed at ca. 2200 cm^−1^, which is attributable to NC stretching. As shown in Table S1, the frequency of this band significantly increases after the coordination on the palladium centre of each of the three isocyanides employed (Δν = 65–95 cm^−1^). This experimental evidence suggests the high contribution of σ-donation to the M-CNR bond [48].

Moreover, in the IR spectra, the two typical bands of the perchlorate anion, related to strong Cl-O stretching and the weak Cl-O bending, are clearly distinguishable at ca. 1090 cm^−1^ and 620 cm^−1^, respectively. The presence of these two signals indirectly confirms the cationic nature of the prepared complexes.

**Figure 2:**
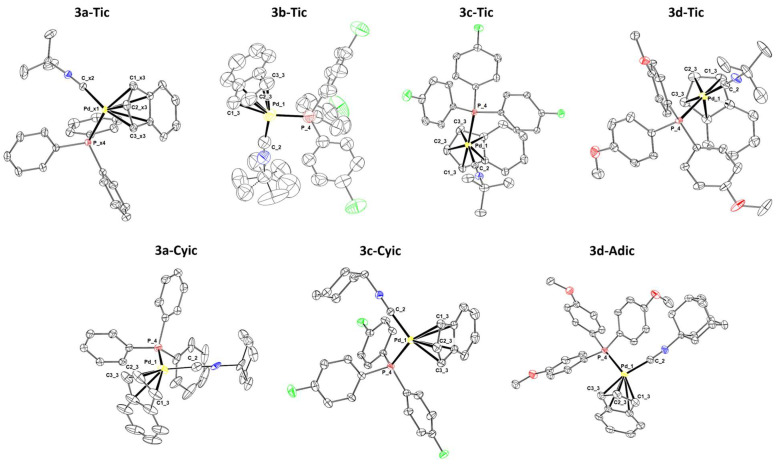
X-ray molecular structures of **3a**–**d-Tic**, **9a-Cyic**, **9c-Cyic**, and **9d-Adic** are presented, showing thermal displacement ellipsoids at the 50% probability level, with hydrogen atoms, counterions, and solvent molecules omitted for clarity.

The characterization of the Pd(II)-indenyl complexes was completed using HRMS spectra, and, in most cases, the structures were resolved using single-crystal X-ray diffraction (see Figure 2).

One of the most important results inferable via the structural data concerns the binding mode of the indenyl fragment in these palladium derivatives. As matter of fact, in all cases, the values of the structural parameters ΔM-C, HA, and FA [49] suggest that the degree of indenyl hapticity is intermediate between ƞ^3^ and ƞ^5^ (ΔM-C = 0.5{(M-C(3a)+M-C(7a)} − 0.5{M-C(1) + M-C(3)}; the hinge angle, HA, is the angle between the planes formed by the atoms C(1), C(2), and C(3) and C(1), C(3), C(3a), and C(7a); the fold angle, FA, is the angle between the planes formed by the atoms C(1), C(2), and C(3) and C(3a), C(4), C(5), C(6), C(7), and C(7a)).

These findings are in perfect agreement with the spectroscopic parameter Δδ^13^C [50], obtainable via the ^13^C NMR spectra of all synthesised complexes, which is always very close to zero (see Table S2).

**Scheme 2:**
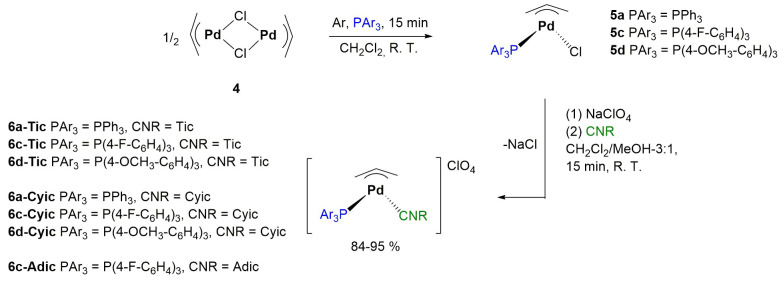
General procedure for the synthesis of Pd(II)-allyl complexes bearing aryl phosphines and bulky alkyl isocyanides.

Since one of the goals of this work is the comparison of the anticancer properties of the mixed phosphine–isocyanide palladium indenyl complexes and their allyl counterparts, we planned to prepare the latter by adopting the same synthetic procedure already described above and now summed up in Scheme 2.

The final cationic products can be isolated with yields close to 90%, and their ^31^P{^1^H} NMR spectra are always marked by the presence of only one singlet, at a chemical shift higher than 30 ppm, compared to the corresponding free phosphine. The presence of two different ancillary ligands affects the spectral structure of the allyl fragment, differentiating the two terminal CH_2_ groups. In the ^13^C{^1^H} NMR spectra, the terminal allyl carbon situated in the *trans* position in relation to the phosphine ligand is distinguishable from that situated in the *trans* position relative to the isocyanide due to its slightly higher chemical shift (Δσ ≅ 6–7 ppm) and, above all, its nature as a doublet (J_C-P_ ≅ 25 Hz) because of its coupling with the phosphorus nucleus. Similarly, terminal allyl protons (both *syn* and *anti*) in the *trans* position relative to the phosphine occur at higher chemical shifts compared to those in the *trans* position relative to the isocyanide and appear as doublets of doublets due to their simultaneous coupling with the central allyl proton and the phosphorus nucleus (see Figures S43–S61). Finally, the coordination of the isocyanide ligand is demonstrated by the positioning of NC stretching, which is always located over 2200 cm^−1^ (see Figure S62).

### 2.2. Reactivity of Indenyl Complexes

**Scheme 3:**
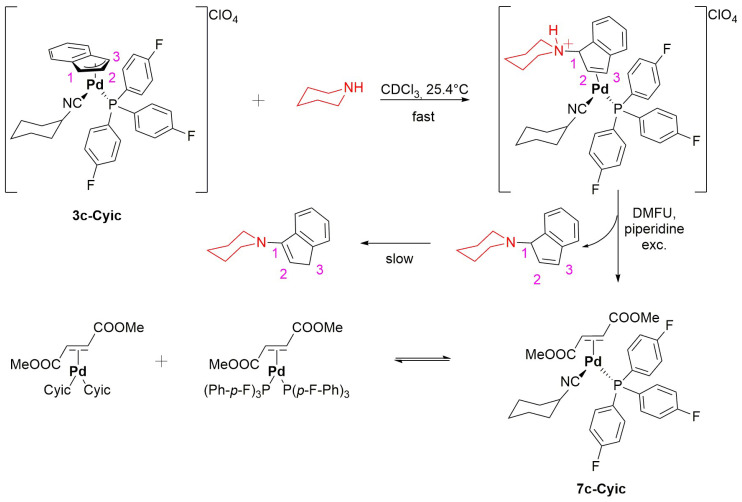
Reaction of indenyl amination with piperidine ([**3c-Cyic**] = 1 equiv., [dimethyl fumarate] = 1.7 equiv., and [piperidine] = 5 equiv.) in CDCl_3_ at 298 K.

Knowledge of the reactivity of organometallic compounds can provide valuable information with which to better understand their mechanisms of action in a biological context. Specifically, considering the large number of nucleophilic agents present in the cellular environment, it is crucial to understand the preferential site of attack on the synthesized complexes. In this case, the metal centre, the coordinated isocyanide, and finally the indenyl moiety are the three potential sites of nucleophilic attack. Of course, the chemoselectivity of the process is strictly dependent on the nature of the nucleophile. In this preliminary study, we focused on the reaction between complex **3c-Cyic** and piperidine, taking advantage of previous studies from our research group [31,51] and considering the great abundance of potential aminic nucleophiles present in the biological environment (i.e., nucleobases and proteins). Piperidine, which is particularly reactive owing to its reduced steric hindrance and high basicity, allows us to obtain fast responses, avoiding collateral decomposition pathways. Furthermore, considering its hard character, it does not have great affinity with the soft palladium(II) centre; therefore, it will preferentially attack the isocyanide to generate a carbene ligand [52] or the indenyl fragment to produce an indenyl amine. The consequence of the second hypothesis is the reduction of the metal centre from Pd(II) to Pd(0), and considering the low coordinative capability of the resulting indenyl amine, the final complexes can easily decompose, making the interpretation of the NMR analyses more challenging. To avoid this eventuality, an electron-withdrawing olefin such as dimethyl fumarate (dmfu) was added to the reaction mixture with the aim of stabilizing the palladium(0) derivatives (Scheme 3), as proven by previous works [51].

The experiment has been carried out in an NMR tube, by reacting **3c-Cyic** with piperidine (5 equiv.) in CDCl_3_ in the presence of dmfu (1.7 equiv.). The results obtained are summarized in Scheme 3.

As can be observed, the process is chemoselective, involving the exclusive attack of piperidine on the C^1^ or C^3^ carbons of the indenyl moiety that leads to the generation of a Pd(0)-η^2^-olefin intermediate species. This species, which is not stable and tends to decompose quickly, is not observable in the NMR spectra of the reaction mixture since the coordinated indenyl piperidine is promptly substituted by the more efficient dmfu, whose electron-withdrawing COOCH_3_ substituents promote a stabilizing metal–ligand backdonation.

It must be said that the final Pd(0)-dmfu complex **7c-Cyic**, coordinating one phosphine and one isocyanide, is in fast equilibrium with small amounts of the two corresponding derivatives bearing two isocyanides or two phosphines, with these last species having been already observed in our previous work [31] and appearing in the ^31^P{^1^H} NMR spectrum of the final mixture. Finally, it is possible to observe the slow conversion of the 1-(1H-indenyl-1-yl)piperidine removed by the metal centre into its more thermodynamically stable isomer 1-(1H-indenyl-3-yl)piperidine (see Figure S63). Anyway, there is no sign of any product deriving from the attack of piperidine on the coordinated isocyanide, an option that had been previously reported for a neutral isocyanide palladium allyl complex [52].

### 2.3. Anti-Cancer Activity of Indenyl and Allyl Complexes

Ovarian cancer represents one of the most problematic forms of neoplasia that affect the female population worldwide [53,54]. Its prognosis is in most cases fatal since, in the first phase of its development, this tumour is practically asymptomatic, and the delayed diagnosis makes its therapeutic treatment very difficult [55]. The first line of chemotherapy protocols includes cisplatin and its derivatives, but unfortunately recurrences are very frequent and often show resistance to platinum-based drugs. Therefore, medical researchers are particularly interested in identifying new therapeutic approaches to overcome this issue. In some of our previous works, we proved that palladium-based drugs can represent a potential interesting option for treating this type of disease [31,32,56,57]; therefore, we planned to test the new complexes prepared in this work to assess their cytotoxicity towards ovarian cancer cells. Moreover, we also decided to explore the anticancer activity of these compounds against one of the most aggressive breast cancer cell lines, known as MDA-MB-231 (triple-negative breast cancer).

For this purpose, after having ascertained that all the complexes were stable in a DMSO-d^6^/D_2_O mixture, we selected the A2780, A2780*cis* (its cisplatin-resistant clone), and OVCAR-5 (high-grade serous ovarian cancer) ovarian cancer cell lines for our antiproliferative activity tests. Finally, MRC-5 lung fibroblasts were chosen to evaluate the compounds’ selectivity towards cancer cells over normal ones. In all cases, cisplatin was used as a positive control, and the results obtained are summarized in Table 1.

In particular, in the analysis of the antiproliferative activity data obtained at the different concentrations used (see experimental section), it was possible to determine the IC_50_ values for each complex (IC_50_ = half inhibitory concentration, which is the concentration of a complex that leads to the death of 50% of the cells present in a culture medium). These values were used to compare the cytotoxicity of our palladium complexes towards the different cell lines investigated.

Our analysis of the in vitro results allows us to draw a few general conclusions:

(a) All the tested complexes exhibit high cytotoxicity towards all three ovarian cancer cell lines. This cytotoxicity is comparable with that of cisplatin and, in many cases, better when we consider the cisplatin-resistant A2780*cis* cell line.
(b) With a few exceptions, the activities of all the compounds are practically the same towards the A2780 and A2780*cis* cell lines. This evidence seems to suggest a mechanism of action different from that of classical platinum-based drugs.
(c) Importantly, the high cytotoxicity of all the compounds was even maintained towards the OVCAR-5 cell line, which is classified as one of the more aggressive according to the High-Grade Serous Ovarian Cancer (HGSOC) classification [58].
(d) The type of phosphine or isocyanide coordinated on the metal centre does not significantly or recurringly influence the cytotoxicity of the complexes. This fact seems to indicate that it is the general structure of these kinds of compounds that determines their biological activity.
(e) Generally speaking, the allyl derivatives are more active than the corresponding indenyl complexes towards the A2780*cis* and OVCAR-5 cell lines, whereas towards A2780 cells, their behaviour remains basically the same.
(f) The IC_50_ values with respect to the MRC-5 cell line point out the significant cytotoxicity of our compounds even towards normal cells, although generally being less marked. In particular, in the case of Pd(II) allyl derivatives, the IC_50_ values obtained for the three ovarian cancer cell lines are of 1–2 orders of magnitude lower than those obtained against MRC-5 fibroblasts, thus suggesting a certain degree of selectivity.
(g) Finally, we tested the potential of our compounds even against another type of cancer cells. We turned our attention to the MDA-MB-231 line, consisting of highly invasive and poorly differentiated cells of triple-negative breast cancer. Our complexes have exhibited an activity significantly higher than that of cisplatin by at least one order of magnitude, with the indenyl derivatives seeming in this case to be slightly more effective than the allyl ones. The only exception is represented by complex **6c-Adic**, which showed an IC_50_ value over two orders of magnitude lower than cisplatin and 40 times lower than that recorded for the MRC-5 cell line.

It is important to underline that the IC_50_ values obtained against ovarian cancer cell lines are generally comparable with those observed in the case of Pd-allyl and Pd-indenyl complexes bearing *N*-heterocyclic carbene and/or phosphine ligands [27,29,31,32]. This fact seems to suggest that the dominant effect is the nature of the organopalladium fragment employed rather than the nature of the ancillary ligands. However, ancillary ligands appear to play a crucial role in whether in vitro selectivity towards cancer cells over normal ones is conferred. For example, in the case of the Pd-allyl complexes, high selectivity was observed in the past both in vitro and ex vivo when an *N*-heterocyclic carbene and 1,3,5-triaza-7-phosphaadamantane were present in the palladium coordination sphere [25].

Regarding the mechanisms of action of the complexes tested in this work, it is plausible that they lead to cell death through apoptosis, inflicting early mitochondrial damage rather than binding with DNA as in the case of platinum-based drugs.

These hypotheses are based on the cationic nature of the synthesized complexes (which favours the localization in the mitochondria) and, above all, on mechanistic studies recently conducted by our group [25,27]. However, in-depth investigations on the modes of action of these compounds, as well as their activity in more complex biological systems such as animal models and patient-derived organoids, will be carried out in the future.

## 3. Materials and Methods

### 3.1. Solvents and Reagents

All syntheses were carried out in an inert atmosphere (Ar) using standard Schlenk techniques. Dichloromethane and diethyl ether were dried under molecular sieves (4 Å, 10%) and stored under Argon atmosphere. All other solvents were commercial-grade products and used as purchased.

[Pd(µ-Cl)(Indenyl)]_2_ (**1**) [59], [Pd(µ-Cl)(ƞ^3^-Allyl)]_2_ (**4**) [60], and [Pd(ƞ^3^-Allyl)(PPh_3_)(Tic)]ClO_4_ (**6a-Tic**) [44] were synthesized according to published protocols. Phosphines and isocyanides were used as supplied.

### 3.2. Instruments

In this study, 1D-NMR and 2D-NMR spectra were recorded using Bruker 300 and 400 MHz Advance spectrometers. Chemical shift values (ppm) are given relative to TMS (^1^H and ^13^C), H_3_PO_4_ (^31^P), and CCl_3_F (^19^F). IR spectra were recorded using a PerkinElmer Spectrum One spectrophotometer (PerkinElmer, Waltham, MA, USA), and UV-Vis spectra were recorded using a PerkinElmer Lambda 40 spectrophotometer equipped with a PerkinElmer PTP 6 (Peltier temperature programmer) apparatus (PerkinElmer, Waltham, MA, USA). HRMS data were collected using a Bruker Compact Q-TOF (Bruker, Billerica, MA, USA).

### 3.3. Synthesis of Cationic Pd(II)-Indenyl Complexes Bearing One Phosphine and One Isocyanide Serving as Ancillary Ligands

#### General Procedure

To 0.1 mmol of [Pd(µ-Cl)(Indenyl)]_2_ (**1**) dissolved in 7 mL of anhydrous CH_2_Cl_2_, a solution of 0.19 mmol of phosphine in 5 mL of anhydrous CH_2_Cl_2_ was added in an inert atmosphere (Ar). The resulting solution was stirred for 15 min at room temperature to obtain the intermediate species **2**. Afterwards, 0.4 mmol of NaClO_4_·H_2_O dissolved in 6 mL of methanol and a solution of 0.2 mmol of isocyanide in 6 mL of CH_2_Cl_2_ were added. The mixture (CH_2_Cl_2_/MeOH ≈ 3/1) was stirred at room temperature for 15 min, and the solvent was removed under vacuum. After the addition of 5 mL of CH_2_Cl_2_, the suspension was filtered through a small pad of Celite, and the solution was concentrated under vacuum. The desired complex **3** was precipitated by addition of diethyl ether, filtrated, and dried under vacuum. 

[Pd(Ind)(PPh_3_)(Tic)]ClO_4_ (**3a-Tic**). Compound **2a** was obtained by employing 0.0644 g (0.125 mmol) of [Pd(µ-Cl)(Indenyl)]_2_ and 0.0623 g (0.238 mmol) of PPh_3_. Concentrations of 0.0547 g (0.389 mmol) of NaClO_4_·H_2_O and 0.0208 g (0.250 mmol) of *tert*-butyl isocyanide were added. A total of 0.1498 g (yield 90%) of complex **3a-Tic** was obtained as an orange powder.

^1^H NMR (300.0 MHz, CDCl_3_, T = 298 K, ppm) δ: 1.13 (s, 9H, ^t^Bu-CH_3_), 5.32 (*pseudo*-t, *J* = 2.5 Hz, 1H, H^3^), 6.28 (d, *J* = 7.6 Hz, 1H, H^4^), 6.68 (t, *J* = 3.2 Hz, 1H, H^2^), 7.02 (t, *J* = 7.6 Hz, 1H, H^5^), 7.09 (m, 1H, H^1^), da 7.15–7.35 (m, 7H, H^6^, Ar-H), 7.36–7.62 (m, 10H, H^7^, Ar-H).

^31^P{^1^H} NMR (121.5 MHz, CDCl_3_, T = 298 K, ppm) δ: 28.9. 

^13^C{^1^H} NMR (75.0 MHz, CDCl_3_, T = 298 K, ppm) δ: 29.6 (CH_3_, ^t^Bu-CH_3_), 59.6 (C, ^t^Bu-C), 88.8 (CH, d, *J*_C-P_ = 4.2 Hz, C^3^), 93.2 (CH, d, *J*_C-P_ = 19.0 Hz, C^1^), 111.7 (CH, d, *J*_C-P_ = 6.1 Hz, C^2^), 117.8 (CH, C^4^), 120.1 (CH, C^7^), 128.0 (CH, C^5^), 128.1 (CH, C^6^), 129.4 (CH, d, *J*_C-P_ = 11.1 Hz, Ar-CH), 130.4 (C, d, *J*_C-P_ = 46.9 Hz, *ipso*-Ar-C), 130.6 (C, d, *J*_C-P_ = 0.5 Hz, C^3a^), 131.8 (C, d, *J*_C-P_ = 4.3 Hz, C^7a^), 132.0–133.7 (CH, Ar-CH).

IR (KBr pellet, cm^−1^): 2212, ν(NC); 1093, ν(ClO); 620, δ(ClO).

HRMS calcd for [C_32_H_31_NPPd]^+^: 566.1236; found: 566.1230.

[Pd(Ind)(P(4-Cl-C_6_H_4_)_3_)(Tic)]ClO_4_ (**3b-Tic**). Compound **2b** was obtained by employing 0.0655 g (0.127 mmol) of [Pd(µ-Cl)(Indenyl)]_2_ and 0.0886 g (0.242 mmol) of tris(4-Cl-phenyl)phosphine. Concentrations of 0.055 g (0.392 mmol) of NaClO_4_·H_2_O and 0.0212 g (0.255 mmol) of *tert*-butyl isocyanide were added. A total of 0.1643 g (yield 84%) of complex **3b-Tic** was obtained as a pale-orange powder.

^1^H NMR (300.0 MHz, CDCl_3_, T = 298 K, ppm) δ: 1.20 (s, 9H, ^t^Bu-CH_3_), 5.39 (*pseudo*-t, *J* = 2.9, Hz, 1H, H^3^), 6.40 (d, *J* = 7.6 Hz, 1H, H^4^), 6.84 (*pseudo-*td, *J* = 3.0, 0.8 Hz, 1H, H^2^), 7.05 (t, *J* = 7.6 Hz, 1H, H^5^), 7.08–7.15 (m, 1H, H^1^), 7.12 (m, 1H, H^6^), 7.16–7.30 (m, 7H, H^6^, *o*-Ar-H), 7.38 (d, *J* = 7.6 Hz, 1H, H^7^), 7.45–7.54 (m, 6H, *m*-Ar-H).

^31^P{^1^H} NMR (121.5 MHz, CDCl_3_, T = 298 K, ppm) δ: 27.2.

^13^C{^1^H} NMR (75.0 MHz, CDCl_3_, T = 298 K, ppm) δ: 29.7 (CH_3_, ^t^Bu-CH_3_), 59.8 (C, ^t^Bu-C), 89.2 (CH, d, *J*_C-P_ = 4.1 Hz, C^3^), 93.8 (CH, d, *J*_C-P_ = 18.8 Hz, C^1^), 113.1 (CH, d, *J*_C-P_ = 6.1 Hz, C^2^), 117.7 (CH, C^4^), 120.3 (CH, C^7^), 128.2 (CH, C^5^), 128.3 (CH, C^6^), 128.3 (C, d, *J*_C-P_ = 48.5 Hz, *ipso*-Ar-C), 129.9 (CH, d, *J*_C-P_ = 11.9 Hz, *m-*Ar-CH), 131.0 (C, d, *J*_C-P_ = 2.5 Hz, C^3a^), 132.0 (C, d, *J*_C-P_ = 4.5 Hz, C^7a^), 134.8 (CH, d, *J*_C-P_ = 13.9 Hz, *o-*Ar-CH), 139.0 (C, d, *J*_C-P_ = 2.9 Hz, *p*-Ar-C). 

IR (KBr pellet, cm^−1^): 2213, ν(NC); 1082, ν(ClO); 623, δ(ClO).

HRMS calcd for [C_32_H_28_Cl_3_NPPd]^+^: 668.0058; found: 668.0042.

[Pd(Ind)(P(4-F-C_6_H_4_)_3_)(Tic)]ClO_4_ (**3c-Tic**). Compound **2c** was obtained by employing 0.0699 g (0.136 mmol) of [Pd(µ-Cl)(Indenyl)]_2_ and 0.0819 g (0.259 mmol) of tris(4-F-phenyl)phosphine. In total, 0.0583 g (0.415 mmol) of NaClO_4_·H_2_O and 0.0227 g (0.273 mmol) of *tert*-butyl isocyanide were added. In total, 0.1467 g (yield 85%) of complex **3c-Tic** was obtained as a pale-brown powder.

^1^H NMR (300.0 MHz, CDCl_3_, T = 298 K, ppm) δ: 1.20 (s, 9H, ^t^Bu-CH_3_), 5.38 (*pseudo-*t, *J* = 2.9 Hz, 1H, H^3^), 6.38 (d, *J* = 7.6 Hz, 1H, H^4^), 6.82 (td, *J* = 3.2, 0.8 Hz, 1H, H^2^), 7.00–7.12 (m, 2H, H^5^, H^1^), 7.15–7.36 (m, 13H, H^6^, Ar-H), 7.39 (d, = 7.6 Hz, 1H, H^7^).

^31^P{^1^H} NMR (121.5 MHz, CDCl_3_, T = 298 K, ppm) δ: 26.5 (q, *J*_P-F_ = 3.1, 2.4 Hz).

^19^F{^1^H} NMR (376.5 MHz, CDCl_3_, T = 298 K, ppm) δ: −105.8 (d, *J*_F-P_ = 2.4 Hz).

^13^C{^1^H} NMR (75.0 MHz, CDCl_3_, T = 298 K, ppm) δ: 30.1 (CH_3_, ^t^Bu-CH_3_), 60.1 (C, ^t^Bu-C), 89.5 (CH, d, *J*_C-P_ = 4.2 Hz, C^3^), 93.9 (CH, d, *J*_C-P_ = 19.0 Hz, C^1^), 113.4 (CH, d, *J*_C-P_ = 6.1 Hz, C^2^), 117.5 (CH, dd, *J*_C-F_, _C-P_ = 21.7, 12.5 Hz, *m-*Ar-CH), 118.1 (CH, C^4^), 120.6 (CH, C^7^), 126.6 (C, dd, *J*_C-P,C-F_ = 50.3, 3.5 Hz, *ipso*-Ar-C), 128.5 (CH, C^5^), 128.6 (CH, C^6^), 131.3 (C, d, *J*_C-P_ = 2.6 Hz, C^3a^), 132.4 (C, d, *J*_C-P_ = 4.4 Hz, C^7a^), 136.3 (CH, dd, *J*_C-P,C-F_ = 14.6, 8.8 Hz, *o-*Ar-CH), 165.9 (C, dd, *J*_C-F,C-P_ = 255.8, 2.7 Hz, *p-*Ar-C).

IR (KBr pellet, cm^−1^): 2201, ν(NC); 1093, ν(ClO); 618, δ(ClO).

HRMS calcd for [C_32_H_28_F_3_NPPd]^+^: 620.0953; found: 620.0945.

[Pd(Ind)(P(4-OCH_3_-C_6_H_4_)_3_)(Tic)]ClO_4_ (**3d-Tic**). Compound **2d** was obtained by employing 0.0608 g (0.118 mmol) of [Pd(µ-Cl)(Indenyl)]_2_ and 0.0791 g (0.225 mmol) of tris(4-OCH_3_-phenyl)phosphine. In total, 0.054 g (0.384 mmol) of NaClO_4_·H_2_O and 0.0196 g (0.236 mmol) of *tert*-butyl isocyanide were added. In total, 0.1628 g (yield 91%) of complex **3d-Tic** was obtained as a pale-orange powder.

^1^H NMR (300.0 MHz, CDCl_3_, T = 298 K, ppm) δ: 1.15 (s, 9H, ^t^Bu-CH_3_), 3.87 (s, 9H, O-CH_3_), 5.25 (*pseudo-*t, *J* = 2.8, 1H, H^3^), 6.42 (d, *J* = 7.6, 1H, H^4^), 6.64 (td, *J* = 3.2, 0.8 Hz, 1H, H^2^), 6.92–7.01 (m, 7H, H^1^, Ar-H), 7.06 (t, *J* = 7.6 Hz, 1H, H^5^), 7.12–7.24 (m, 7H, H^6^, Ar-H), 7.39 (d, = 7.6 Hz, 1H, H^7^).

^31^P{^1^H} NMR (121.5 MHz, CDCl_3_, T = 298 K, ppm) δ: 24.6.

^13^C{^1^H} NMR (75.0 MHz, CDCl_3_, T = 298 K, ppm) δ: 29.7 (CH_3_, ^t^Bu-CH_3_), 55.8 (CH_3_, O-CH_3_), 59.5 (C, ^t^Bu-C), 88.2 (CH, d, *J*_C-P_ = 4.3 Hz, C^3^), 92.4 (CH, d, *J*_C-P_ = 19.6 Hz, C^1^), 111.4 (CH, d, *J*_C-P_ = 6.1 Hz, C^2^), 114.9 (CH, d, *J*_C-P_ = 12.4 Hz, Ar-CH), 117.9 (CH, C^4^), 119.8 (CH, C^7^), 121.8 (C, d, *J*_C-P_ = 54.6 Hz, *ipso*-Ar-C), 127.7 (CH, C^5^), 127.8 (CH,C^6^), 130.4 (C, d, *J*_C-P_ = 1.8 Hz C^3a^), 131.8 (C, d, *J*_C-P_ = 4.2 Hz C^7a^), 135.0 (CH, d, *J*_C-P_ = 13.9 Hz, Ar-CH), 162.3 (C, d, *J*_C-P_ = 2.3 Hz, *p*-Ar-C).

IR (KBr pellet, cm^−1^): 2212, ν(NC); 1093, ν(ClO); 618, δ(ClO).

HRMS calcd for [C_35_H_37_O_3_NPPd]^+^: 656.1554; found: 656.1535.

**[Pd(Ind)(PPh_3_)(Cyic)]ClO_4_** (**3a-Cyic**). Compound **2a** was obtained by employing 0.0537 g (0.104 mmol) of [Pd(µ-Cl)(Indenyl)]_2_ and 0.0521 g (0.199 mmol) of PPh_3_. In total, 0.0461 g (0.328 mmol) of NaClO_4_·H_2_O and 0.0228 g (0.209 mmol) of cyclohexyl isocyanide were added. In total, 0.1328 g (yield 92%) of complex **3a-Cyic** was obtained as a yellow powder.

^1^H NMR (300.0 MHz, CDCl_3_, T = 298 K, ppm) δ: 1.02–1.32 (bm, 8H, Cy-CH_2_), 1.61 (bs, 2H, Cy-CH_2_), 3.82–3.95 (bm, 1H, Cy-CH), 5.31 (*pseudo*-t, *J* = 2.9, Hz, 1H, H^3^), 6.30 (d, *J* = 7.7 Hz, 1H, H^4^), 6.62 (td, *J* = 3.2, 0.8 Hz, 1H, H^2^), 7.03 (t, *J* = 7.5, Hz, 1H, H^5^), 7.07–7.14 (m, 1H, H^1^), 7.18–7.31 (m, 7H, H^6^, *o-*Ar-H), 7.41–7.59 (m, 10H, H^7^, *m,p-*Ar-H).

^31^P{^1^H} NMR (121.5 MHz, CDCl_3_, T = 298 K, ppm) δ: 29.0.

^13^C{^1^H} NMR (75.0 MHz, CDCl_3_, T = 298 K, ppm) δ: 22.4–31.5 (CH_2_, Cy-CH_2_), 55.5 (CH, Cy-CH), 88.9 (CH, d, *J*_C-P_ = 4.3 Hz, C^3^), 93.2 (CH, d, *J*_C-P_ = 18.8 Hz, C^1^), 111.5 (CH, d, *J*_C-P_ = 6.1 Hz, C^2^), 117.7 (CH, C^4^), 120.4 (CH, C^7^), 128.0 (CH, C^5^), 128.1 (CH, C^6^), 129.4 (CH, d, *J*_C-P_ = 11.3 Hz, *m-*Ar-CH), 130.4 (C, d, *J*_C-P_ = 48.1 Hz, *ipso*-Ar-C), 130.6 (C, d, *J*_C-P_ = 1.9 Hz, C^3a^), 131.8 (C, d, *J*_C-P_ = 4.1 Hz, C^7a^), 131.9 (CH, d, *J*_C-P_ = 2.7 Hz, *p*-Ar-CH), 133.6 (CH, d, *J*_C-P_ = 12.5 Hz, *o-*Ar-CH).

IR (KBr pellet, cm^−1^): 2207, ν(NC); 1090, ν(ClO); 615, δ(ClO).

HRMS calcd for [C_34_H_33_NPPd]^+^: 592.1393; found: 592.1372.

[Pd(Ind)(P(4-Cl-C_6_H_4_)_3_)(Cyic)]ClO_4_ (**3b-Cyic**). Compound **2b** was obtained by employing 0.0698 g (0.136 mmol) of [Pd(µ-Cl)(Indenyl)]_2_ and 0.0942 g (0.258 mmol) of tris(4-Cl-phenyl)phosphine. In total, 0.0582 g (0.414 mmol) of NaClO_4_·H_2_O and 0.0296 g (0.271 mmol) of cyclohexyl isocyanide were added. In total, 0.1643 g (yield 84%) of complex **3b-Cyic** was obtained as a pale-orange powder.

^1^H NMR (300.0 MHz, CDCl_3_, T = 298 K, ppm) δ: 1.04–1.36 (bm, 8H, Cy-CH_2_), 1.64 (bs, 2H, Cy-CH_2_), 3.87–3.98 (bm, 1H, Cy-CH), 5.39 (*pseudo*-t, *J* = 2.9 Hz, 1H, H^3^), 6.41 (d, *J* = 7.6 Hz, 1H, H^4^), 6.74 (td, *J* = 3.2, 0.7 Hz, 1H, H^2^), 7.05 (t, *J* = 7.5 Hz, 1H, H^5^), 7.13–7.25 (m, 8H, H^1^, H^6^, *o-*Ar-H), 7.42 (d, *J* = 7.6 Hz, 1H, H^7^), 7.45–7.51 (m, 6H, *m-*Ar-H).

^31^P{^1^H} NMR (121.5 MHz, CDCl_3_, T = 298 K, ppm) δ: 27.4.

^13^C{^1^H} NMR (75.0 MHz, CDCl_3_, T = 298 K, ppm) δ: 22.6–31.6 (CH_2_, Cy-CH_2_), 55.7 (CH, Cy-CH), 89.2 (CH, d, *J*_C-P_ = 3.8 Hz, C^3^), 94.0 (CH, d, *J*_C-P_ = 19.2 Hz, C^1^), 112.7 (CH, d, *J*_C-P_ = 6.2 Hz, C^2^), 117.6 (CH, C^4^), 120.6 (CH, C^7^), 128.2 (CH, C^5^), 128.4 (CH, C^6^), 128.8 (C, d, *J*_C-P_ = 48.3 Hz, *ipso-*Ar-C), 130.0 (CH, d, *J*_C-P_ = 11.9 Hz, *m-*Ar-CH), 131.0 (C, d, *J*_C-P_ = 1.8 Hz, C^3a^), 131.9 (C, d, *J*_C-P_ = 4.5 Hz, C^7a^), 134.8 (CH, d, *J*_C-P_ = 13.9 Hz, *o-*Ar-CH), 139.0 (C, d, *J*_C-P_ = 2.8 Hz, *p*-Ar-C).

IR (KBr pellet, cm^−1^): 2212, ν(NC); 1082, ν(ClO); 620, δ(ClO).

HRMS calcd for [C_34_H_30_Cl_3_NPPd]^+^: 694.0215; found: 694.0185.

[Pd(Ind)(P(4-F-C_6_H_4_)_3_)(Cyic)]ClO_4_ (**3c-Cyic**). Compound **2c** was obtained by employing 0.0706 g (0.137 mmol) of [Pd(µ-Cl)(Indenyl)]_2_ and 0.0826 g (0.261 mmol) of tris(4-F-phenyl)phosphine. In total, 0.0583 g (0.415 mmol) of NaClO_4_·H_2_O and 0.030 g (0.275 mmol) of cyclohexyl isocyanide were added. In total, 0.1820 g (yield 89%) of complex **3c-Cyic** was obtained as a pale-green powder.

^1^H NMR (300.0 MHz, CDCl_3_, T = 298 K, ppm) δ: 1.03–1.38 (bm, 8H, Cy-CH_2_), 1.64 (bs, 2H, Cy-CH_2_), 3.84–4.02 (bm, 1H, Cy-CH), 5.37 (*pseudo*-t, *J* = 2.9 Hz, 1H, H^3^), 6.39 (d, *J* = 7.6 Hz, 1H, H^4^), 6.74 (td, *J* = 3.2, 0.8 Hz, 1H, H^2^), 7.04 (t, *J* = 8.4, Hz, 1H, H^5^), 7.06–7.18 (m, 1H, H^1^), 7.13–7.36 (m, 13H, H^6^, Ar-H), 7.42 (d, *J* = 8.0 Hz, 1H, H^7^).

^31^P{^1^H} NMR (121.5 MHz, CDCl_3_, T = 298 K, ppm) δ: 29.6 (q, *J*_P-F_ = 2.5 Hz).

^19^F{^1^H} NMR (376.5 MHz, CDCl_3_, T = 298 K, ppm) δ: −105.8 (d, *J*_F-P_ = 2.5 Hz).

^13^C{^1^H} NMR (75.0 MHz, CDCl_3_, T = 298 K, ppm) δ: 22.5–31.6 (CH_2_, Cy-CH_2_), 55.6 (CH, Cy-CH), 89.2 (CH, d, *J*_C-P_ = 4.2 Hz, C^3^), 93.3 (CH, d, *J*_C-P_ = 19.2 Hz, C^1^), 112.4 (CH, d, *J*_C-P_ = 6.1 Hz, C^2^), 117.1 (CH, dd, *J*_C-F,C-P_ = 21.6, 12.5 Hz, *m-*Ar-CH), 117.6 (CH, C^4^), 120.4 (CH, C^7^), 126.1 (C, dd, *J*_C-P,C-F_ = 50.2, 3.5 Hz, *ipso*-Ar-C), 128.1 (CH, C^5^), 128.3 (CH, C^6^), 130.9 (C, d, *J*_C-P_ = 2.3 Hz, C^3a^), 131.9 (C, d, *J*_C-P_ = 4.5 Hz, C^7a^), 135.8 (CH, dd, *J*_C-P,C-F_ = 14.6, 8.7 Hz, *o-*Ar-CH), 164.9 (C, dd, *J*_C-F,C-P_ = 255.8, 2.6 Hz, *p-*Ar-C).

IR (KBr pellet, cm^−1^): 2207, ν(NC); 1085, ν(ClO); 620, δ(ClO).

HRMS calcd for [C_34_H_30_F_3_NPPd]^+^: 646.1110; found: 646.1096.

[Pd(Ind)(P(4-OCH_3_-C_6_H_4_)_3_)(Cyic)]ClO_4_ (**3d-Cyic**). Compound **2d** was obtained by employing 0.0704 g (0.137 mmol) of [Pd(µ-Cl)(Indenyl)]_2_ and 0.0917 g (0.260 mmol) of tris(4-OCH_3_-phenyl)phosphine. In total, 0.0597 g (0.425 mmol) of NaClO_4_·H_2_O and 0.0299 g (0.274 mmol) of cyclohexyl isocyanide were added. In total, 0.1916 g (yield 89%) of complex **3d-Cyic** was obtained as an orange powder.

^1^H NMR (300.0 MHz, CDCl_3_, T = 298K, ppm) δ: 1.03–1.36 (bm, 8H, Cy-CH_2_), 1.57 (bs, 2H, Cy-CH_2_), 3.87 (s, 9H, O-CH_3_) 3.88–3.96 (bm, 1H, Cy-CH), 5.24 (*pseudo*-t, *J* = 2.6 Hz, 1H, H^3^), 6.43 (d, *J* = 7.6 Hz, 1H, H^4^), 6.60 (td, *J* = 3.2, 0.8 Hz, 1H, H^2^), 6.91–7.02 (m, 7H, H^1^, *m-*Ar-H), 7.06 (t, *J* = 7.6 Hz, 1H, H^5^), 7.12–7.24 (m, 7H, H^6^, *o-*Ar-H), 7.40 (d, *J* = 7.7 Hz, 1H, H^7^)

^31^P{^1^H} NMR (121.5 MHz, CDCl_3_, T = 298 K, ppm) δ: 24.7.

^13^C{^1^H} NMR (75.0 MHz, CDCl_3_, T = 298 K, ppm) δ: 22.4–31.5 (CH_2_, Cy-CH_2_), 55.4 (CH, Cy-CH), 55.8 (CH_3_, O-CH_3_), 88.3 (CH, d, *J*_C-P_ = 4.2 Hz, C^3^), 92.2 (CH, d, *J*_C-P_ = 18.8 Hz, C^1^), 111.2 (CH, d, *J*_C-P_ = 6.1 Hz, C^2^), 114.9 (CH, d, *J*_C-P_ = 12.4 Hz, *m-*Ar-CH), 117.8 (CH, C^4^), 120.0 (CH, C^7^), 122.0 (C, d, *J*_C-P_ = 48.1 Hz, *ipso-*Ar-C), 127.7 (CH, C^5^) 127.8 (CH, C^6^), 130.5 (C, d, *J*_C-P_ = 1.4 Hz, C^3a^), 131.8 (C, d, *J*_C-P_ = 3.9 Hz, C^7a^), 135.1 (CH, d, *J*_C-P_ = 14.0 Hz, *o-*Ar-CH), 162.3 (C, d, *J*_C-P_ = 2.5 Hz, *p-*Ar-C).

IR (KBr pellet, cm^−1^): 2212, ν(NC); 1093, ν(ClO); 618, δ(ClO).

HRMS calcd for [C_37_H_39_O_3_NPPd]^+^: 682.1711; found: 682.1690.

[Pd(Ind)(PPh_3_)(Adic)]ClO_4_ (**3a-Adic**). Compound **2a** was obtained by employing 0.0277 g (0.0539 mmol) of [Pd(µ-Cl)(Indenyl)]_2_ and 0.0268 g (0.102 mmol) of PPh_3_. In total, 0.0256 g (0.182 mmol) of NaClO_4_·H_2_O and 0.0173 g (0.107 mmol) of adamantyl isocyanide were added. In total, 0.7331 g (yield 91%) of complex **3a-Adic** was obtained as a yellow powder.

^1^H NMR (300.0 MHz, CDCl_3_, T = 298 K, ppm) δ: 1.42–1.55 (m, 6H, Ad-CH_2_), 1.62 (d, *J* = 2.9 Hz, 6H, Ad-CH_2_), 1.97 (bs, 3H, Ad-CH), 5.32 (*pseudo-*t, *J* = 2.2 Hz, 1H, H^3^), 6.29 (d, *J* = 7.6, 1H, H^4^), 6.69 (td, *J* = 3.2, 0.8 Hz, 1H, H^2^), 6.95–7.11 (m, 2H, H^5^, H^1^), 7.22 (t, *J* = 7.6 Hz, 1H, H^6^), 7.23–7.35 (m, 6H, *o-*Ar-H), 7.40 (d, *J* = 7.7 Hz, 1H, H^7^), 7.45–7.61 (m, 9H, *m-*Ar-H)

^31^P{^1^H} NMR (121.5 MHz, CDCl_3_, T = 298 K, ppm) δ: 29.0.

^13^C{^1^H} NMR (75.0 MHz, CDCl_3_, T = 298 K, ppm) δ: 28.6 (CH, Ad-CH), 35.1 (CH_2_, Ad-CH_2_), 42.2 (CH_2_, Ad-CH_2_), 59.3 (C, Ad-C), 89.0 (CH, d, *J*_C-P_ = 3.8 Hz, C^3^), 93.2 (CH, d, *J*_C-P_ = 18.8 Hz, C^1^), 111.9 (CH, d, *J*_C-P_ = 6.0 Hz, C^2^), 117.8 (CH, C^4^), 120.1 (CH, C^7^), 128.0 (CH, C^5^), 128.1 (CH, C^6^), 129.4 (CH, d, *J*_C-P_ = 11.7 Hz, *m-*Ar-CH), 130.3 (d, C, *J*_C-P_ = 48.3 Hz, *ipso*-Ar-C), 130.7 (C, d, *J*_C-P_ = 1.4 Hz, C^3a^), 131.9 (C, d, *J*_C-P_ = 4.4 Hz, C^7a^), 132.0 (CH, d, *J*_C-P_ = 2.7 Hz, *p*-Ar-CH), 133.6 (CH, d, *J*_C-P_ = 12.5 Hz, *o-*Ar-CH).

IR (KBr pellet, cm^−1^): 2201, ν(NC); 1088, ν(ClO); 620, δ(ClO).

HRMS calcd for [C_38_H_37_NPPd]^+^: 644.1707; found: 644.1688.

[Pd(Ind)(P(4-Cl-C_6_H_4_)_3_)(Adic)]ClO_4_ (**3b-Adic**). Compound **2b** was obtained by employing 0.0781 g (0.152 mmol) of [Pd(µ-Cl)(Indenyl)]_2_ and 0.1056 g (0.289 mmol) of tris(4-Cl-phenyl)phosphine. In total, 0.064 g (0.456 mmol) of NaClO_4_·H_2_O and 0.049 g (0.304 mmol) of adamantyl isocyanide were added. In total, 0.2213 g (yield 86%) of complex **3b-Adic** was obtained as a dark-orange powder.

^1^H NMR (300.0 MHz, CDCl_3_, T = 298 K, ppm) δ: 1.55 (q, *J* = 12.5 Hz, 6H, Ad-CH_2_), 1.67 (d, *J* = 2.9 Hz, 6H, Ad-CH_2_), 2.01 (s, 3H, Ad-CH), 5.43 (bt, 2.2 Hz, 1H, H^3^), 6.41 (d, *J* = 7.6, 1H, H^4^), 6.85 (td, *J* = 3.3, 0.8 Hz, 1H, H^2^), 6.98–7.14 (m, 2H, H^5^, H^1^), 7.16–7.28 (m, 7H, H^6^, *o-*Ar-H), 7.36 (bd, *J* = 7.6, 1H, H^7^), 7.45–7.52 (m, 6H, *m-*Ar-H).

^31^P{^1^H} NMR (121.5 MHz, CDCl_3_, T = 298 K, ppm) δ: 27.4.

^13^C{^1^H} NMR (75.0 MHz, CDCl_3_, T = 298 K, ppm) δ: 28.6 (CH, Ad-CH), 35.1 (CH_2_, Ad-CH_2_), 42.3 (CH_2_, Ad-CH_2_), 59.5 (C, Ad-C), 89.2 (CH, d, *J*_C-P_ = 4.0 Hz, C^3^), 93.7 (CH, d, *J*_C-P_ = 19.0 Hz, C^1^), 113.2 (CH, d, *J*_C-P_ = 6.2 Hz, C^2^), 117.7 (CH, C^4^), 120.2 (CH, C^7^), 128.1 (CH, C^5^), 128.3 (CH, C^6^), 128.4 (C, d, *J*_C-P_ = 48.5 Hz, *ipso*-Ar-C), 130.0 (CH, d, *J*_C-P_ = 11.8 Hz, *m-*Ar-CH), 131.0 (C, d, *J*_C-P_ = 2.2 Hz, C^3a^), 132.0 (C, d, *J*_C-P_ = 4.4 Hz, C^7a^), 134.8 (CH, d, *J*_C-P_ = 14.1, *o-*Ar-CH), 139.0 (C, d, *J*_C-P_ = 2.8 Hz, *p-*Ar-C).

IR (KBr pellet, cm^−1^): 2207, ν(NC); 1082, ν(ClO); 620, δ(ClO).

HRMS calcd for [C_38_H_34_Cl_3_NPPd]^+^: 746.0529; found: 746.0501.

[Pd(Ind)(P(4-F-C_6_H_4_)_3_)(Adic)]ClO_4_ (**3c-Adic**). Compound **2c** was obtained by employing 0.080 g (0.156 mmol) of [Pd(µ-Cl)(Indenyl)]_2_ and 0.0935 g (0.296 mmol) of tris(4-F-phenyl)phosphine. In total, 0.0694 g (0.494 mmol) of NaClO_4_·H_2_O and 0.0501 g (0.311 mmol) of adamantyl isocyanide were added. In total, 0.2115 g (yield 85%) of complex **3c-Adic** was obtained as a pale-green powder.

^1^H NMR (300.0 MHz, CDCl_3_, T = 298 K, ppm) δ: 1.40–1.64 (m, 6H, Ad-CH_2_), 1.68 (d, *J* = 2.9 Hz, 6H, Ad-CH_2_), 2.00 (bs, 3H, Ad-CH), 5.40 (*pseudo-*t, *J* = 2.2 Hz, 1H, H^3^), 6.39 (d, *J* = 7.8, 1H, H^4^), 6.83 (td, *J* = 3.2, 0.8 Hz, 1H, H^2^), 6.99–7.10 (m, 2H, H^5^, H^1^), 7.16–7.34 (m, 13H, H^6^, Ar-H), 7.36 (d, *J* = 7.8, 1H, H^7^).

^31^P{^1^H} NMR (121.5 MHz, CDCl_3_, T = 298 K, ppm) δ: 26.6 (q, *J*_P-F_ = 2.5 Hz).

^19^F{^1^H} NMR (376.5 MHz, CDCl_3_, T = 298 K, ppm) δ: −105.9 (d, *J*_F-P_ = 2.5 Hz).

^13^C{^1^H} NMR (75.0 MHz, CDCl_3_, T = 298 K, ppm) δ: 28.6 (CH, Ad-CH), 35.1 (CH_2_, Ad-CH_2_), 42.3 (CH_2_, Ad-CH_2_), 59.5 (C, Ad-C), 89.3 (CH, d, *J*_C-P_ = 4.2 Hz, C^3^), 93.3 (CH, d, *J*_C-P_ = 19.1 Hz, C^1^), 112.9 (CH, d, *J*_C-P_ = 6.0 Hz, C^2^), 117.1 (CH, dd, *J*_C-F,C-P_ = 21.5, 12.5 Hz, *m-*Ar-CH), 117.8 (CH, C^4^), 120.2 (CH, C^7^), 126.1 (C, dd, *J*_C-P,C-F_ = 50.3, 3.4 Hz, *ipso*-Ar-C), 128.1 (CH, C^5^), 128.2 (CH, C^6^), 131.0 (C, d, *J*_C-P_ = 1.3 Hz, C^3a^), 132.0 (C, d, *J*_C-P_ = 4.1 Hz, C^7a^), 135.9 (CH, dd, *J*_C-P,C-F_ = 14.6, 8.7 Hz, *o-*Ar-CH), 164.9 (C, dd, *J*_C-F,C-P_ = 255.8, 2.7 Hz, *p-*Ar-C). 

IR (KBr pellet, cm^−1^): 2218, ν(NC); 1090, ν(ClO); 620, δ(ClO).

HRMS calcd for [C_38_H_34_F_3_NPPd]^+^: 698.1424; found: 698.1397.

[Pd(Ind)(P(4-OCH_3_-C_6_H_4_)_3_)(Adic)]ClO_4_ (**3d-Adic**). Compound **2d** was obtained by employing 0.0622 g (0.112 mmol) of [Pd(µ-Cl)(Indenyl)]_2_ and 0.0809 g (0.230 mmol) of tris(4-OCH_3_-phenyl)phosphine. In total, 0.0526 g (0.375 mmol) of NaClO_4_·H_2_O and 0.039 g (0.242 mmol) of adamantyl isocyanide were added. In total, 0.1827 g (yield 90%) of complex **3d-Adic** was obtained as a pale-brown powder.

^1^H NMR (300.0 MHz, CDCl_3_, T = 298 K, ppm) δ: 1.52 (q, *J* = 12.9 Hz, 6H, Ad-CH_2_), 1.63 (d, *J* = 2.3 Hz, 6H, Ad-CH_2_), 1.98 (bs, 3H, Ad-CH), 3.87 (s, 9H, O-CH_3_), 5.28 (bt, 1H, H^3^), 6.42 (d, *J* = 7.6, 1H, H^4^), 6.63 (t, *J* = 3.2 Hz, 1H, H^2^), 6.87–6.95 (m, 1H,H^1^), 6.95–7.01 (m, 6H, Ar-H), 7.05 (t, *J* = 7.6, 1H, H^5^), 7.11–7.24 (m, 7H, H^6^, Ar-H), 7.37 (d, *J* = 7.7, 1H, H^7^)

^31^P{^1^H} NMR (121.5 MHz, CDCl_3_, T = 298 K, ppm) δ: 24.8.

^13^C{^1^H} NMR (75.0 MHz, CDCl_3_, T = 298 K, ppm) δ: 28.6 (CH, Ad-CH), 35.1 (CH_2_, Ad-CH_2_), 42.3 (CH_2_, Ad-CH_2_), 55.8 (CH3, O-CH3), 59.2 (C, Ad-C), 88.3 (CH, d, *J*_C-P_ = 3.5 Hz, C^3^), 92.3 (CH, d, *J*_C-P_ = 19.5 Hz, C^1^), 111.5 (CH, d, *J*_C-P_ = 6.2 Hz, C^2^), 114.9 (CH, d, *J*_C-P_ = 12.4 Hz, Ar-CH), 118.0 (CH, C^4^), 119.8 (CH, C^7^), 121.9 (C, d, *J*_C-P_ = 54.5 Hz, *ipso*-Ar-C), 127.8 (CH, C^5^, C^6^), 130.5 (C, d, *J*_C-P_ = 1.7 Hz, C^3a^), 131.8 (C, d, *J*_C-P_ = 4.2 Hz, C^7a^), 135.1 (CH, d, *J*_C-P_ = 14.0, Ar-CH), 162.3 (C, d, *J*_C-P_ = 2.5 Hz, *p-*Ar-C).

IR (KBr pellet, cm^−1^): 2212, ν(NC); 1093, ν(ClO); 618, δ(ClO).

HRMS calcd for [C_41_H_43_O_3_NPPd]^+^: 734.2025; found: 734.2003.

### 3.4. Synthesis of Cationic Pd(II)-Allyl Complexes Bearing One Phosphine and One Isocyanide as Ancillary Ligands

#### General Procedure

To 0.1 mmol of [Pd(µ-Cl)(ƞ^3^-Allyl)]_2_ (**4**) dissolved in 5 mL of anhydrous CH_2_Cl_2_, a solution of 0.2 mmol of phosphine in 3 mL of anhydrous CH_2_Cl_2_, 0.4 mmol of NaClO_4_·H_2_O dissolved in 3 mL of methanol, and 0.2 mmol of isocyanide in 3 mL of CH_2_Cl_2_ (CH_2_Cl_2_/MeOH ≈ 3/1) was added. The mixture was stirred at room temperature for 30 min, and the solvent was removed under vacuum. Afterwards, 5 mL of CH_2_Cl_2_ was added, and the mixture was filtered through a small pad of Celite. The solution was concentrated under vacuum, and the desired complex **6** was precipitated via the addition of diethyl ether, filtrated, and dried under vacuum. 

[Pd(ƞ^3^-Allyl)(P(4-F-C_6_H_4_)_3_)(Tic)]ClO_4_ (**6c-Tic**). Compound **6c-Tic** was obtained by employing 0.0489 g (0.134 mmol) of [Pd(µ-Cl)(ƞ^3^-Allyl)]_2_ and 0.0845 g (0.267 mmol) of tris(4-F-phenyl)phosphine. In total, 0.0750 g (0.535 mmol) of NaClO_4_·H_2_O and 0.0222 g (0.267 mmol) of *tert*-butyl isocyanide were added. In total, 0.1616 g (yield 92%) of complex **6c-Tic** was obtained as a white powder.

^1^H NMR (CDCl_3_, T = 298 K, ppm) δ: 1.36 (s, 9H, ^t^Bu-CH_3_), 3.31 (d, *J*_H-*H*_ = 13.2 Hz, 1H, *anti* allyl-H *trans* C), 3.87–3.95 (m, 2H, *syn* allyl-H *trans* C, *anti* allyl-H *trans* P), 5.18 (dd, *J*_H-H,H-P_ = 6.2, 6.2 Hz, 1H, *syn* allyl-H *trans* P), 5.72–5.84 (m, 1H, *central* allyl-H), 7.21–7.28 (m, 6H, Ar-H), 7.46–7.54 (m, 6H, Ar-H).

^13^C{^1^H} NMR (CDCl_3_, T = 298 K, ppm) δ: 29.7 (CH_3_, ^t^Bu-CH_3_), 59.0 (C, C(CH_3_)), 70.9 (CH_2_, allyl-CH_2_ *trans* C), 76.8 (CH_2_, allyl-CH_2_ *trans* P, obscured by solvent peak and detected by HMQC), 116.8 (CH, dd, *J*_C-F,C-P_ = 21.5, 12.0 Hz, *m*-Ar-CH), 123.3 (CH, d, *J*_C-P_ = 5.4 Hz, *central* allyl-CH), 127.1 (C, d, *J*_C-P,C-F_ = 46.8, 2.9 Hz, *ipso*-Ar-C), 135.8 (CH, dd, *J*_C-P,C-F_ = 15.1, 8.6 Hz, *o-*Ar-CH), 164.6 (C, d, *J*_C-F_ = 253.6 Hz, *p*-Ar-C); NC: not detectable.

^31^P{^1^H} NMR (CDCl_3_, T = 298 K, ppm) δ: 21.6 (q, *J*_P-F_ = 2.7 Hz).

IR (KBr pellet, cm^−1^): 2217 ν(NC); 1092 ν(ClO); 622 δ (ClO).

HRMS calcd for [C_26_H_26_F_3_NPPd]^+^: 546.0794; found: 546.0779.

[Pd(ƞ^3^-Allyl)(P(4-OCH_3_-C_6_H_4_)_3_)(Tic)]ClO_4_ (**6d-Tic**). Compound **6d-Tic** was obtained by employing 0.0352 g (0.0962 mmol) of [Pd(µ-Cl)(ƞ^3^-Allyl)]_2_ and 0.0678 g (0.1924 mmol) of tris(4-F-phenyl)phosphine. In total, 0.0541 g (0.3848 mmol) of NaClO_4_·H_2_O and 0.0160 g (0.1924 mmol) of *tert*-butyl isocyanide were added. In total, 0.1268 g (yield 95%) of complex **6d-Tic** was obtained as a white powder.

^1^H NMR (CDCl_3_, T = 298 K, ppm) δ: 1.31 (s, 9H, ^t^Bu-CH_3_), 3.17 (d, *J*_H-H_ = 13.2 Hz, 1H, *anti* allyl-H *trans* C), 3.70 (dd, *J*_H-H,H-P_ = 13.6, 9.8 Hz, 1H, *anti* allyl-H *trans* P), 3.80–3.87 (m, 11H, OCH_3_, *syn* allyl-H *trans* C), 5.11 (dd, *J*_H-H,H-P_ = 6.0, 6.0 Hz, 1H, *syn* allyl-H *trans* P), 5.67–5.81 (m, 1H, *central* allyl-H), 6.96–7.03 (m, 6H, Ar-H), 7.30–7.40 (m, 6H, Ar-H). 

^13^C{^1^H} NMR (CDCl_3_, T = 298 K, ppm) δ: 29.8 (CH_3_, ^t^Bu-CH_3_), 55.6 (CH_3_, OCH_3_), 59.0 (C, ^t^Bu-CH_3_), 70.6 (CH_2_, d, *J*_C-P_ = 3.1 Hz, allyl-CH_2_ *trans* C), 76.0 (CH_2_, d, *J*_C-P_ = 25.8 Hz, allyl-CH_2_ *trans* P), 114.9 (CH, d, *J*_C-P_ = 11.9 Hz, *m*-Ar-CH), 122.8 (C, d, *J*_C-P_ = 50.7 Hz, *ipso*-Ar-C), 123.1 (CH, d, *J*_C-P_ = 5.8 Hz, *central* allyl-CH), 135.2 (CH, d, *J*_C-P_ = 14.5 Hz, *o*-Ar-CH), 162.0 (C, d, *J*_C-P_ = 2.2 Hz, *p*-Ar-C), NC: not detectable.

^31^P{^1^H} NMR (CDCl_3_, T = 298 K, ppm) δ: 20.0.

IR (KBr pellet, cm^−1^): 2216 ν(NC); 1092 ν(ClO); 623δ (ClO); 1092; 622.

HRMS calcd for [C_29_H_35_O_3_NPPd]^+^: 582.1395; found: 582.1378.

[Pd(ƞ^3^-Allyl)(PPh_3_)(Cyic)]ClO_4_ (**6a-Cyic**). Compound **6a-Cyic** was obtained by employing 0.0526 g (0.143 mmol) of [Pd(µ-Cl)(ƞ^3^-Allyl)]_2_ and 0.0753 g (0.287 mmol) of PPh_3_. In total, 0.0806 g (0.574 mmol) of NaClO_4_·H_2_O and 0.0314 g (0.287 mmol) of cyclohexyl isocyanide were added. In total, 0.1602 g (yield 84%) of complex **6a-Cyic** was obtained as a white powder.

^1^H NMR (CDCl_3_, T = 298 K, ppm) δ: 1.19–1.53 (bm, 8H, cyclohexyl-CH_2_), 1.69–1.82 (bm, 2H, cyclohexyl-CH_2_), 3.23 (d, *J*_H-H_ = 13.2 Hz, 1H, *anti* allyl-H *trans* C), 3.77 (dd, *J*_H-H,H-P_ = 13.7, 9.8 Hz, 1H, *anti* allyl-H *trans* P), 3.84–3.96 (m, 2H, *syn* allyl-H *trans* C, cyclohexyl-CH), 5.23 (dd, *J*_H-H,H-P_ = 6.0, 6.0 Hz, 1H, *syn* allyl-H *trans* P), 5.78 (m, 1H, *central* allyl-H), 7.40–7.62 (m, 15H, PPh_3_).

^13^C{^1^H} NMR (CDCl_3_, T = 298 K, ppm) δ: 22.5 (CH_2_, cyclohexyl-CH_2_), 24.7 (CH_2_, cyclohexyl-CH_2_), 31.6 (CH_2_, cyclohexyl-CH_2_), 55.0 (CH, cyclohexyl-CH), 70.7 (CH_2_, allyl-CH_2_ *trans* C), 76.3 (CH_2_, d, *J*_C-P_ = 23.9 Hz, allyl-CH_2_ *trans* P), 123.1 (CH, d, *J*_C-P_ = 5.2 Hz, *central* allyl), 129.3 (CH, d, *J*_C-P_ = 10.7 Hz, PPh_3_), 131.0 (C, d, *J*_C-P_ = 45.3 Hz, *ipso*-PPh_3_), 133.6 (CH, d, *J*_C-P_ = 13.1, PPh_3_), NC: not detectable.

^31^P{^1^H} NMR (CDCl_3_, T = 298 K, ppm) δ: 24.0.

IR (KBr pellet, cm^−1^): = 2217 ν (NC); 1093 ν (ClO); 623 δ (ClO).

HRMS calcd for [C_28_H_31_NPPd]^+^: 518.1234; found: 518.1224.

[Pd(ƞ^3^-Allyl)(P(4-F-C_6_H_4_)_3_)(Cyic)]ClO_4_ (**6c-Cyic**). Compound **6c-Cyic** was obtained by employing 0.0518 g (0.142 mmol) of [Pd(µ-Cl)(ƞ^3^-Allyl)]_2_ and 0.0895 g (0.283 mmol) of tris(4-F-phenyl)phosphine. In total, 0.0795 g (0.566 mmol) of NaClO_4_·H_2_O and 0.0309 g (0.283 mmol) of cyclohexyl isocyanide were added. In total, 0.1650 g (yield 90%) of complex **6c-Cyic** was obtained as a pale-yellow powder.

^1^H NMR (CDCl_3_, T = 298 K, ppm) δ: 1.25–1.58 (bm, 8H, cyclohexyl-CH_2_), 1.72–1.87 (bm, 2H, cyclohexyl-CH_2_), 3.30 (d, *J*_H-H_ = 13.0 Hz, 1H, *anti* allyl-H *trans* C), 3.85–3.92 (m, 3H, *syn* allyl-H *trans* C, *anti* allyl-H *trans* P, cyclohexyl-CH), 5.20 (dd, *J*_H-H,H-P_ = 6.1, 6.1 Hz, 1H, *syn* allyl-H *trans* P), 5.81 (m, 1H, *central* allyl-H), 7.21–7.28 (m, 6H, Ar-H), 7.45–7.54 (m, 6H, Ar-H).

^13^C{^1^H} NMR (CDCl_3_, T = 298 K, ppm) δ: 22.6 (CH_2_, cyclohexyl-CH_2_), 24.6 (CH_2_, cyclohexyl-CH_2_), 31.6 (CH_2_, cyclohexyl-CH_2_), 55.1 (CH, cyclohexyl-CH), 71.0 (CH_2_, d, *J*_C-P_ = 2.4 Hz, allyl-CH_2_ *trans* C), 76.9 (CH_2_, allyl-CH_2_ *trans* P, obscured by solvent peak and detected by HMQC), 116,9 (CH, dd, *J*_C-F,C-P_ = 21.5, 12.0 Hz, *m*-Ar-CH), 123.3 (CH, d, *J*_C-P_ = 5.4 Hz, *central* allyl), 126.8 (C, dd, *J*_C-P,C-F_ = 46.9, 3.4 Hz, *ipso*-Ar-C), 135.8 (CH, dd, *J*_C-P,C-F_ = 15.1, 8.6 Hz, *o*-Ar-CH), 164.7 (C, dd, *J*_C-F,C-P_ = 254.8, 2.4 Hz, *p*-Ar-C); NC: not detectable.

^31^P{^1^H} NMR (CDCl_3_, T = 298 K, ppm) δ: 21.7 (q, *J*_P-F_ = 2.5 Hz).

IR (KBr pellet, cm^−1^): 2218 ν (NC); 1090 ν (ClO); 622 δ (ClO).

HRMS calcd for [C_28_H_28_F_3_NPPd]^+^: 572.0972; found: 572.0934

[Pd(ƞ^3^-Allyl)(P(4-OCH_3_-C_6_H_4_)_3_)(Cyic)]ClO_4_ (**6d-Cyic**). Compound **6d-Cyic** was obtained by employing 0.040 g (0.1093 mmol) of [Pd(µ-Cl)(ƞ^3^-Allyl)]_2_ and 0.0771 g (0.2186 mmol) of tris(4-OCH_3_-phenyl)phosphine. In total, 0.0615 g (0.4373 mmol) of NaClO_4_·H_2_O and 0.0239 g (0.2186 mmol) of cyclohexyl isocyanide were added. In total, 0.1313 g (yield 95%) of complex **6d-Cyic** was obtained as a pale-yellow powder.

^1^H NMR (CDCl_3_, T = 298 K, ppm) δ: 1.18–1.55 (bm, 8H, cyclohexyl-CH_2_), 1.67–1.84 (bm, 2H, cyclohexyl-CH_2_), 3.17 (d, *J*_H-H_ = 12.9 Hz, 1H, *anti* allyl-H *trans* C), 3.71 (dd, *J*_H-H,H-P_ = 13.7, 9.7 Hz, 1H, *anti* allyl-H *trans* P), 3.80–3.97 (m, 11H, OCH_3_, *syn* allyl-H *trans* C, cyclohexyl-CH), 5.15 (dd, *J*_H-H,H-P_ = 6.8, 6.8 Hz, 1H, *syn* allyl-H *trans* P), 5.74 (m, 1H, *central* allyl-H), 7.02 (d, *J*_H-H_ = 8.7 Hz, 6H, *m*-Ar-H), 7.37 (dd, *J*_H-P,H-H_ = 11.5, 8.7 Hz, 6H, *o*-Ar-H).

^13^C{^1^H} NMR (CDCl_3_, T = 298 K, ppm) δ: 22.5 (CH_2_, cyclohexyl-CH_2_), 24.6 (CH_2_, cyclohexyl-CH_2_), 31.5 (CH_2_, cyclohexyl-CH_2_), 55.1 (CH, cyclohexyl-CH), 55.5 (CH_3_, OCH_3_), 69.8 (CH_2_, allyl-CH_2_ *trans* C), 75.8 (CH_2_, d, *J*_C-P_ = 26.5 Hz, allyl-CH_2_ *trans* P), 114.8 (CH, d, *J*_C-P_ = 11.9 Hz, *m*-Ar-CH), 122.6 (C, d, *J*_C-P_ = 50.8 Hz, *ipso*-Ar-C), 122.8 (CH, d, *J*_C-P_ = 5.9 Hz, *central* allyl), 135.0 (CH, d, *J*_C-P_ = 14.5, *o*-Ar-CH), 161.9 (C, *p*-Ar-C); NC: not detectable.

IR (KBr pellet, cm^−1^): 2210 ν (NC); 1099 ν (ClO); 622 δ (ClO)

HRMS calcd for [C_31_H_37_O_3_NPPd]^+^: 608.1552; found: 608.1518

[Pd(ƞ^3^-Allyl)(P(4-F-C_6_H_4_)_3_)(Adic)]ClO_4_ (**6c-Adic**). Compound **6c-Adic** was obtained by employing 0.0494 g (0.135 mmol) of [Pd(µ-Cl)(ƞ^3^-Allyl)]_2_ and 0.0853 g (0.270 mmol) of tris(4-F-phenyl)phosphine. In total, 0.0609 g (0.434 mmol) of NaClO_4_·H_2_O and 0.0435 g (0.270 mmol) of 1-Adamantyl isocyanide were added. In total, 0.1394 g (yield 71%) of complex **6c-Adic** was obtained as a white powder.

^1^H NMR (300.0 MHz, CDCl_3_, T = 298 K, ppm) δ: 1.57–1.72 (m, 6H, Ad-CH_2_), 1.85 (bs, *J*_H-H_ = 1.4 Hz, 6H, Ad-CH_2_), 2.07 (bs, 3H, Ad-CH), 3.30 (d, *J*_H-H_ = 13.2 Hz, 1H, *anti* allyl-H *trans* C), 3.90 (m, 2H, *syn* allyl-H *trans* C, *anti* allyl-H *trans* P), 5.13 (bs, 1H, *syn* allyl-H *trans* P), 5.66–5.91 (m, 1H, *central* allyl*-*H), 7.15–7.25 (m, 9H, Ar-H), 7.39–7.55 (m, 6H, Ar-H).

^13^C{^1^H} NMR (75.0 MHz, CDCl_3_, T = 298 K, ppm) δ: 28.6 (CH, Ad-CH), 35.2 (CH_2_, Ad-CH_2_), 42.5 (CH_2_, Ad-CH_2_), 58.9 (C, Ad-C), 71.2 (CH_2_, d, *J*_C-P_ = 2.7 Hz, *allyl*-CH_2_ *trans*-C), 77.0 (CH_2_, *allyl*-CH_2_ *trans* P, obscured by solvent peak and detected by HMQC), 117.0 (CH, dd, *J*_C-F,C-P_ = 21.6, 12.0 Hz, Ar-CH), 123.5 (CH_2_, d, *J*_C-P_ = 5.6 Hz, *central allyl-*CH), 127.0 (C, dd, *J*_C-P,C-F_ = 46.8, 3.6 Hz, *ipso*-Ar-C), 136.0 (CH, dd, *J*_C-P,C-F_ = 15.2, 8.6 Hz, Ar-CH), 164.8 (C, dd, *J*_C-F,C-P_ = 254.6, 2.4 Hz, *p-*Ar-C); NC: not detectable.

^31^P{^1^H} NMR (121.5 MHz, CDCl_3_, T = 298 K, ppm) δ: 21.6 (q, *J*_P-F_ = 2.8 Hz).

^19^F{^1^H} NMR (376.5 MHz, CDCl_3_, T = 298 K, ppm) δ: −106.8 (d, *J*_F-P_ = 2.8 Hz).

IR (KBr pellet, cm^−1^): 2201, ν(NC); 1085, ν(ClO); 620, δ(ClO).

HRMS calcd for [C_32_H_32_F_3_NPPd]^+^: 624.1266; found: 624.1232.

### 3.5. Crystal Structure Determination

**3a-Tic**, **3c-Tic**, **3d-Tic**, **3b-Tic**, **3c-Cyic**, **3d-Adic**, and **3a-Cyic** crystal data were collected at XRD2 beamline of the Elettra Synchrotron, Trieste (Italy) [61], using a monochromatic wavelength of 0.620 Å at 100 K or 298 K. The data sets were integrated, scaled, and corrected for Lorentz, absorption, and polarization effects using XDS package [62]. Data from two random orientations were merged to obtain complete datasets for the triclinic **3d-Adic** crystal forms using CCP4-Aimless [63,64]. The structures were determined via direct methods using SHELXT program [65] and refined using full-matrix least-squares implemented in SHELXL-2019/3 [66].

Thermal motions were treated anisotropically for all non-hydrogen atoms with occupancies greater than 50%, and hydrogens were included at calculated positions (riding on their carrier atoms). Data from **3a-Cyic** at 100 K were modelled as a 2-component non merohedral twin, with domains related by twofold rotation around (1 0 0) reciprocal lattice direction (twin fraction refined to ~10%). Twin law was identified through Platon TWINROTMAT [67] routine. Geometric and thermal restrains (SADI, SAME, DFIX, DANG, and SIMU) were used to properly model disordered and poorly defined fragments. The Coot program was used for structure building [68]. Pictures were prepared using Ortep3 (1.0.3) [69] and Pymol [70] software. The crystal data are given in Table S3.

Crystallographic data were deposited at the Cambridge Crystallographic Data Centre and allocated the deposition numbers CCDC 2290052, 2290053, 2290054, 2290061, 2290055, 2290056, 2290058, 2290057, 2290059, and 2290060 for **3a-Tic** at 100 K, **3c-Tic** at 100 K, **3d-Tic** at 100 K, **3d-Tic** at 100 K (eenantiomorphic packing), **3b-Tic** at 298 K, **3c-Cyic** at 100 K, **3d-Adic** at 100 K, **3d-Adic** at 298 K, **3a-Cyic** at 100 K, and **3a-Cyic** at 298 K, respectively. These data can be obtained free of charge via https://www.ccdc.cam.ac.uk/structures (accessed on 7 January 2024).

### 3.6. NMR Studies of Indenyl Amination

Concentrations of 0.0043 g (0.0057 mmol) of complex **3c-Cyic** and 0.0014 g (0.0097 mmol) of dimethylfumarate (dmfu) were dissolved in 0.7 mL of CDCl_3_ and transferred into an NMR tube. ^1^H NMR and ^31^P{^1^H} NMR data were recorded at room temperature (t = 0).

A total of 0.02 mL of a 1.47 M solution of piperidine (in CDCl_3_) was added, and ^1^H NMR and ^31^P{^1^H} NMR data were recorded at t = 7, 30, and 120 min and t = 10, 33, and 123 min, respectively.

### 3.7. Cytotoxicity Assay

Three ovarian cancer cell lines (A2780, A2780*cis*, and OVCAR-5), one breast cancer cell line (MDAMB-231), and one normal cell line (MRC-5) were grown in accordance with the supplier’s instructions and maintained at 37 °C in humidified atmosphere of 5% of CO_2_. In total, 1 × 10^3^ cancer cells and 8 × 10^3^ MRC-5 were seeded in a 96-well plate and treated after 24 h with six different concentrations of Pd(II) complexes (0.001, 0.01, 0.1, 1, 10, and 100 µM). Notably, stock solutions (10 mM) of all palladium complexes were prepared using DMSO as solvent. After 96 h from the treatment, cell viability was measured with a CellTiter-Glo assay (Promega, Madison, WI, USA) using Tecan M1000 or Synergy^H1^ microplate readers. IC_50_ values were calculated from logistical dose–response curves using GraphPad Prism 8 software. Averages were obtained from triplicates, and error bars are standard deviations.

## 4. Conclusions

In this work, we developed a synthetic protocol for a class of new palladium–indenyl complexes characterized by the simultaneous presence of one aryl phosphine and one bulky alkyl isocyanide. These compounds, which were obtained selectively, without contamination of the derivatives bearing two phosphines or two isocyanides, were fully characterized using NMR, IR, and HRMS. Moreover, in many cases, it was also possible to obtain the crystal structures via X-ray diffractometry. The spectroscopic and diffractometric data agree with respect to the presence of intermediate hapticity between ƞ^3^ and ƞ^5^ of the indenyl fragment in all the synthesized complexes, confirming, as already noted in some our previous works, that this feature is substantially independent of the nature of the ancillary ligands.

Our study on the regioselectivity of amine nucleophilic attacks on these compounds proved that they involve the indenyl fragment selectively producing the most thermodynamically stable indenyl amine and reducing the oxidation state of the palladium centre from +2 to 0. Neither coordinated isocyanide nor the metal centre were involved in the attack of amine, and this information could be useful for understanding behaviour in cellular and extra-cellular environments where many aminic nucleophiles are present.

We also prepared some palladium allyl complexes bearing the same phosphine and isocyanide ligands, adopting a synthetic approach analogous to that of the indenyl derivatives, with the aim of comparing the anticancer activity promoted by the two different organometallic fragments.

All the compounds prepared in this work showed a level of cytotoxicity towards three different lines of ovarian cancer that was completely comparable to cisplatin, which was taken as a reference. In further detail, the efficacy of our complexes remains the same passing from cisplatin-sensitive A2780 to cisplatin-resistant A2780*cis* cells, thus suggesting a mechanism of action probably different from that of classical platinum-based drugs. In perspective, this property can be important for the treatment of ovarian cancer relapses, taking into account even the high activity shown by our complexes against OVCAR-5 high-grade serous ovarian cancer cells.

Concerning the structure–activity relationships, our analysis of the cytotoxicity data points out the substantial independence of IC_50_ values from the type of aryl phosphine or isocyanide employed, while the allyl fragment seems to promote a slightly higher activity with respect to the indenyl one. 

Regrettably, the antiproliferative activity of our complexes towards non-cancer cells MRC-5 is not negligible, although it is slightly lower than that in ovarian cancer cells. In particular, in the case of the allyl derivatives, it always remains lower by at least one order of magnitude, ensuring a small margin of selectivity.

Our complexes were also tested on one breast cancer cell line (MDA-MB-231), with the results showing significantly greater activity than cisplatin, as evidenced by the IC_50_ values that were always 1–2 orders of magnitude lower. Intriguingly, in this case, the indenyl derivatives are mildly more cytotoxic than the corresponding allyls, with the only exception being complex **6c-Adic**.

Ultimately, these preliminary in vitro results lead us to believe that this class of palladium derivatives deserves to be considered in further studies aiming to better define their modes of action and the maintenance of their efficiency on more complex in vitro models as well and to develop a strategy for selectively delivering them to tumour tissue.

## Figures and Tables

**Table 1:** Antiproliferative activity on A2780, A2780*cis*, OVCAR-5, MDA-MB-231, and MRC-5 cell lines.

Compound	IC_50_ (µM)				
	A2780	A2780*cis*	OVCAR-5	MDA-MB-231	MRC-5
cisplatin	0.30 ± 0.02	7.2 ± 0.8	0.9 ± 0.1	11.5 ± 0.8	4.5 ± 0.1
**3a-Tic**	0.70 ± 0.09	1.5 ± 0.2	2.4 ± 0.3	0.32 ± 0.05	3.1 ± 0.2
**3b-Tic**	0.43 ± 0.08	0.3 ± 0.1	0.44 ± 0.03	0.36 ± 0.08	3.38 ± 0.09
**3c-Tic**	0.6 ± 0.1	1.8 ± 0.3	1.60 ± 0.06	1.3 ± 0.2	3.1 ± 0.2
**3d-Tic**	0.32 ± 0.02	1.6 ± 0.8	3.3 ± 0.2	0.46 ± 0.09	4.11 ± 0.05
**3a-Cyic**	0.29 ± 0.06	1.87 ± 0.08	2.6 ± 0.5	0.65 ± 0.05	3.0 ± 0.1
**3b-Cyic**	0.45 ± 0.05	0.8 ± 0.2	0.43 ± 0.08	0.33 ± 0.05	3.4 ± 0.1
**3c-Cyic**	0.69 ± 0.01	0.97 ± 0.03	1.13 ± 0.07	1.4 ± 0.2	5.3 ± 0.5
**3d-Cyic**	0.28 ± 0.03	2.8 ± 0.1	3.0 ± 0.4	1.1 ± 0.4	3.8 ± 0.1
**3a-Adic**	0.40 ± 0.01	2.1 ± 0.3	0.33 ± 0.07	1.2 ± 0.5	3.2 ± 0.1
**3b-Adic**	0.21 ± 0.08	0.73 ± 0.07	0.26 ± 0.04	2.0 ± 0.5	2.8 ± 0.3
**3c-Adic**	0.12 ± 0.02	1.9 ± 0.1	0.26 ± 0.06	0.9 ± 0.5	3.2 ± 0.2
**3d-Adic**	0.32 ± 0.05	1.27 ± 0.09	1.14 ± 0.07	0.36 ± 0.03	2.5 ± 0.4
**6a-Tic**	0.10 ± 0.02	0.30 ± 0.06	0.37 ± 0.07	1.2 ± 0.2	10 ± 2
**6c-Tic**	0.14 ± 0.03	0.55 ± 0.09	0.34 ± 0.03	3.9 ± 0.7	4.7 ± 0.8
**6d-Tic**	0.33 ± 0.03	0.41 ± 0.07	0.35 ± 0.07	1.21 ± 0.09	3.4 ± 0.2
**6aCyic**	0.27 ± 0.08	0.3 ± 0.1	0.241 ± 0.007	2.9 ± 0.4	3.6 ± 0.2
**6c-Cyic**	0.23 ± 0.03	0.41 ± 0.03	0.26 ± 0.02	2.5 ± 0.2	4.2 ± 0.5
**6a-Cyic**	0.20 ± 0.04	0.28 ± 0.04	0.26 ± 0.03	1.3 ± 0.4	3.7 ± 0.2
**6c-Adic**	0.27 ± 0.05	0.4 ± 0.2	0.29 ± 0.06	0.071 ± 0.007	2.9 ± 0.4

Data after 96 h of incubation. Stock solutions were prepared in DMSO for all complexes; stock solutions were prepared in H_2_O/NaCl for cisplatin. A2780 (cisplatin-sensitive ovarian cancer cells), A2780*cis* (cisplatin-resistant ovarian cancer cells), OVCAR-5 (high-grade serous ovarian cancer cells), MDA-MB-231 (triple-negative breast cancer cells), and MRC-5 (normal lung fibroblasts).

## Data Availability

Data are contained within the article and Supplementary Materials.

## Supplementary Materials

The following supporting information can be downloaded at: https://www.mdpi.com/article/10.3390/molecules29020345/s1, Figure S1: ^1^H NMR spectra of compound **3a-Tic** in CDCl_3_ at 298K; Figure S2: ^13^C{^1^H} NMR spectra of compound **3a-Tic** in CDCl_3_ at 298K; Figure S3. ^31^P{^1^H} NMR spectra of compound **3a-Tic** in CDCl_3_ at 298K; Figure S4. ^1^H NMR spectra of compound **3b-Tic** in CDCl_3_ at 298K; Figure S5: ^13^C{^1^H} NMR spectra of compound **3b-Tic** in CDCl_3_ at 298K; Figure S6: ^31^P{^1^H} NMR spectra of compound **3b-Tic** in CDCl_3_ at 298K; Figure S7: ^1^H NMR spectra of compound **3c-Tic** in CDCl_3_ at 298K; Figure S8: ^13^C{^1^H} NMR spectra of compound **3c-Tic** in CDCl_3_ at 298K; Figure S9: ^31^P{^1^H} NMR spectra of compound **3c-Tic** in CDCl_3_ at 298K; Figure S10: ^91^F{^1^H} NMR spectra of compound **3c-Tic** in CDCl_3_ at 298K; Figure S11: ^1^H NMR spectra of compound **3d-Tic** in CDCl_3_ at 298K; Figure S12: ^13^C{^1^H} NMR spectra of compound **3d-Tic** in CDCl_3_ at 298K; Figure S13. ^31^P{^1^H} NMR spectra of compound **3d-Tic** in CDCl_3_ at 298K; Figure S14: ^1^H NMR spectra of compound **3a-Cyic** in CDCl_3_ at 298K; Figure S15. ^13^C{^1^H} NMR spectra of compound **3a-Cyic** in CDCl_3_ at 298K; Figure S16: ^31^P{^1^H} NMR spectra of compound **3a-Cyic** in CDCl_3_ at 298K; Figure S17: ^1^H NMR spectra of compound **3b-Cyic** in CDCl_3_ at 298K; Figure S18: ^13^C{^1^H} NMR spectra of compound **3b-Cyic** in CDCl_3_ at 298K; Figure S19: ^31^P{^1^H} NMR spectra of compound **3b-Cyic** in CDCl_3_ at 298K; Figure S20. ^1^H NMR spectra of compound **3c-Cyic** in CDCl_3_ at 298K; Figure S21. ^13^C{^1^H} NMR spectra of compound **3c-Cyic** in CDCl_3_ at 298K; Figure S22. ^31^P{^1^H} NMR spectra of compound **3c-Cyic** in CDCl_3_ at 298K; Figure S23. ^19^F{^1^H} NMR spectra of compound **3c-Cyic** in CDCl_3_ at 298K; Figure S24. ^1^H NMR spectra of compound **3d-Cyic** in CDCl_3_ at 298K; Figure S25. ^13^C{^1^H} NMR spectra of compound **3d-Cyic** in CDCl_3_ at 298K; Figure S26. ^31^P{^1^H} NMR spectra of compound **3d-Cyic** in CDCl_3_ at 298K; Figure S27. ^1^H NMR spectra of compound **3a-Adic** in CDCl_3_ at 298K; Figure S28: ^13^C{^1^H} NMR spectra of compound **3a-Adic** in CDCl_3_ at 298K; Figure S29: ^31^P{^1^H} NMR spectra of compound **3a-Adic** in CDCl_3_ at 298K; Figure S30: ^1^H NMR spectra of compound **3b-Adic** in CDCl_3_ at 298K; Figure S31: ^13^C{^1^H} NMR spectra of compound **3b-Adic** in CDCl_3_ at 298K; Figure S32: ^31^P{^1^H} NMR spectra of compound **3b-Adic** in CDCl_3_ at 298K; Figure S33: ^1^H NMR spectra of compound **3c-Adic** in CDCl_3_ at 298K; Figure S34: ^13^C{^1^H} NMR spectra of compound **3c-Adic** in CDCl_3_ at 298K; Figure S35: ^31^P{^1^H} NMR spectra of compound **3c-Adic** in CDCl_3_ at 298K; Figure S36: ^19^F{^1^H} NMR spectra of compound **3c-Adic** in CDCl_3_ at 298K; Figure S37: ^1^H NMR spectra of compound **3d-Adic** in CDCl_3_ at 298K; Figure S38: ^13^C{^1^H} NMR spectra of compound **3d-Adic** in CDCl_3_ at 298K; Figure S 39: ^31^P{^1^H} NMR spectra of compound **3d-Adic** in CDCl_3_ at 298K; Figure S40. IR spectra of compound **3c-Tic**; Figure S41: IR spectra of compound **3c-Cyic**; Figure S42: IR spectra of compound **3c-Adic**; Figure S43: ^1^H NMR spectra of compound **6c-Tic** in CDCl_3_ at 298K; Figure S44: ^13^C{^1^H} NMR spectra of compound **6c-Tic** in CDCl_3_ at 298K; Figure S45: ^31^P{^1^H} NMR spectra of compound **6c-Tic** in CDCl_3_ at 298K; Figure S46: ^1^H NMR spectra of compound **6d-Tic** in CDCl_3_ at 298K; Figure S47: ^13^C{^1^H} NMR spectra of compound **6d-Tic** in CDCl_3_ at 298K; Figure S48: ^31^P{^1^H} NMR spectra of compound **6d-Tic** in CDCl_3_ at 298K; Figure S49: ^1^H NMR spectra of compound **6a-Cyic** in CDCl_3_ at 298K; Figure S50: ^13^C{^1^H} NMR spectra of compound **6a-Cyic** in CDCl_3_ at 298K; Figure S51: ^31^P{^1^H} NMR spectra of compound **6a-Cyic** in CDCl_3_ at 298K; Figure S52: ^1^H NMR spectra of compound **6c-Cyic** in CDCl_3_ at 298K; Figure S53: ^13^C{^1^H} NMR spectra of compound **6c-Cyic** in CDCl_3_ at 298K; Figure S54: ^31^P{^1^H} NMR spectra of compound **6c-Cyic** in CDCl_3_ at 298K; Figure S55: ^1^H NMR spectra of compound **6d-Cyic** in CDCl_3_ at 298K; Figure S56: ^13^C{^1^H} NMR spectra of compound **6d-Cyic** in CDCl_3_ at 298K; Figure S57: ^31^P{^1^H} NMR spectra of compound **6d-Cyic** in CDCl_3_ at 298K; Figure S58: ^1^H NMR spectra of compound **6c-Adic** in CDCl_3_ at 298K; Figure S59: ^13^C{^1^H} NMR spectra of compound **6c-Adic** in CDCl_3_ at 298K; Figure S60: ^31^P{^1^H} NMR spectra of compound **6c-Adic** in CDCl_3_ at 298K; Figure S61: ^19^F{^1^H} NMR spectra of compound **6c-Adic** in CDCl_3_ at 298K; Figure S62: IR spectra of compound **6c-Adic;** Figure S63: Amination reaction of compound **3c-Cyic** monitored by NMR spectroscopy (^1^H NMR spectra in CDCl3 at 298 K); Figure S64: HRMS spectra of compound **3a-Tic**; Figure S65: HRMS spectra of compound **3b-Tic**; Figure S66: HRMS spectra of compound **3c-Tic**; Figure S67: HRMS spectra of compound **3d-Tic**; Figure S68: HRMS spectra of compound **3a-Cyic**; Figure S69: HRMS spectra of compound **3b-Cyic**; Figure S70: HRMS spectra of compound **3c-Cyic**; Figure S71: HRMS spectra of compound **3d-Cyic;** Figure S72: HRMS spectra of compound **3a-Adic;** Figure S73: HRMS spectra of compound **3b-Adic**; Figure S74: HRMS spectra of compound **3c-Adic**; Figure S75: HRMS spectra of compound **3d-Adic**; Figure S76: HRMS spectra of compound **6c-Tic**; Figure S77: HRMS spectra of compound **6d-Tic**; Figure S78: HRMS spectra of compound **6a-Cyic**; Figure S79: HRMS spectra of compound **6c-Cyic**; Figure S80: HRMS spectra of compound **6d-Cyic**; Figure S81: HRMS spectra of compound **6c-Adic;** Figure S82: Ortep representations of asymmetric unit contents for **3a-Tic** at 100 K (A), **3c-Tic** at 100 K (B), **3d-Tic** at 100 K (C), **3d-Tic** at 100 K (enantiomorphic packing—D), **3b-Tic** at 298 K (E), **3c-Cyic** at 100 K (F), **3d-Adic** at 100 K (G), **3d-Adic** at 298 K (H), **3a-Cyic** at 100 K (I) and **3a-Cyic** at 298 K (L). Ellipsoids dimensions correspond to 50% probability; Figure S83: Overlap of crystallographically independent molecules bearing tert-butyl isocyanide complexes (**3a-Tic**, **3c-Tic**, **3d-Tic**, **3b-Tic**—yellow sticks), 1-adamantyl isocyanide complexes (**3d-Adic**—blue sticks) and cyclohexyl isocyanide complexes (**3c-Cyic**, **3a-Cyic**—pink sticks). Hydrogens omitted for clarity: Table S1: ν(NC)_free_, ν(NC)_coord_ and ∆ν(NC) values; Table S2: C^3a^ and C^7a^ chemical shifts data and **Δδ^13^C** values and HA, FA and ΔM-C values calculated by X-ray crystal structures; Table S3: Crystallographic data; Table S4: Selected palladium distances and angles for **3a-Tic** at 100 K; Table S5: Selected palladium distances and angles for **3c-Tic** at 100 K; Table S6: Selected palladium distances and angles for **3d-Tic** at 100 K; Table S7: Selected palladium distances and angles for **3b-Tic** at 298 K; Table S8: Selected palladium distances and angles for **3c-Cyic** at 100 K; Table S9: Selected palladium distances and angles for **3d-Cyic** at 100 K and 298 K; Table S10: Selected palladium distances and angles for **EB6x** at 100 K and 298 K. Reference [71] is cited in the supplementary materials.
