# Supplementary material for: Rational Design of Palladium(II) Indenyl and Allyl Complexes Bearing Phosphine and Isocyanide Ancillary Ligands with Promising Antitumor Activity
Source: Molecules. 2024 Jan 10;29(2):345. doi: 10.3390/molecules29020345 (PMC10819880; doi:10.3390/molecules29020345)

checkCIF/PLATON report

Structure factors have been supplied for datablock(s) EB42

THIS REPORT IS FOR GUIDANCE ONLY. IF USED AS PART OF A REVIEW PROCEDURE FOR PUBLICATION, IT SHOULD NOT REPLACE THE EXPERTISE OF AN EXPERIENCED CRYSTALLOGRAPHIC REFEREE.

No syntax errors found. CIF dictionary Interpreting this report

Datablock: EB42

Bond precision:	C-C = 0.0048 A		Wavelength=0.62000
Cell:	a=9.905(2)	b=34.151(7)	c=9.978(2)
	alpha=90	beta=90.58(3)	gamma=90
Temperature:	100 K		

	Calculated	Reported
Volume	3375.0(12)	3375.0(12)
Space group	P 21	P 21
Hall group	P 2yb	P 2yb
Moiety formula	C32 H31 N P Pd, Cl O4, C H Cl3	C32 H31 N P Pd, Cl O4, C H Cl3
Sum formula	C33 H32 Cl4 N O4 P Pd	C33 H32 Cl4 N O4 P Pd
Mr	785.77	785.76
Dx,g cm-3	1.546	1.546
Z	4	4
Mu (mm-1)	0.647	0.647
F000	1592.0	1592.0
F000'	1589.01	
h,k,lmax	16,56,16	15,56,16
Nref	32786[16609]	28759
Tmin,Tmax	0.962,0.987	
Tmin'	0.937	

Correction method= Not given

Data completeness= 1.73/0.88 Theta(max)= 31.121

R(reflections)= 0.0343(27801) wR2(reflections)= 0.0866(28759)

S = 1.008 Npar= 799

The following ALERTS were generated. Each ALERT has the format
test-name_ALERT_alert-type_alert-level.
Click on the hyperlinks for more details of the test.

Alert level C

```
PLAT220_ALERT_2_C NonSolvent   Resd 1  C   Ueq(max)/Ueq(min) Range        3.3 Ratio
PLAT242_ALERT_2_C Low    'MainMol' Ueq as Compared to Neighbors of      Cl_12 Check
PLAT244_ALERT_4_C Low    'Solvent' Ueq as Compared to Neighbors of      Cl_15 Check
PLAT244_ALERT_4_C Low    'Solvent' Ueq as Compared to Neighbors of      Cl_25 Check
PLAT244_ALERT_4_C Low    'Solvent' Ueq as Compared to Neighbors of      C1_16 Check
PLAT911_ALERT_3_C Missing FCF Refl Between Thmin & STh/L=    0.600         33 Report
PLAT971_ALERT_2_C Check Calcd Resid. Dens.  1.16Ang From Cl3_16          2.11 eA-3
PLAT971_ALERT_2_C Check Calcd Resid. Dens.  0.81Ang From Cl2_16          1.98 eA-3
PLAT977_ALERT_2_C Check Negative Difference Density on H5_23     .      -0.41 eA-3
```

Alert level G

```
ABSMU01_ALERT_1_G  Calculation of _exptl_absorpt_correction_mu
                not performed for this radiation type.
PLAT092_ALERT_4_G Check: Wavelength Given is not Cu,Ga,Mo,Ag,In Ka    0.62000 Ang.
PLAT232_ALERT_2_G Hirshfeld Test Diff (M-X)  Pd_21    --C_22     .        5.1 s.u.
PLAT720_ALERT_4_G Number of Unusual/Non-Standard Labels ..........        152 Note
PLAT790_ALERT_4_G Centre of Gravity not Within Unit Cell: Resd.  #          5 Note
              C H Cl3
PLAT912_ALERT_4_G Missing # of FCF Reflections Above STh/L=  0.600       1572 Note
PLAT933_ALERT_2_G Number of HKL-OMIT Records in Embedded .res File         32 Note
PLAT965_ALERT_2_G The SHELXL WEIGHT Optimisation has not Converged     Please Check
PLAT978_ALERT_2_G Number C-C Bonds with Positive Residual Density.          0 Info
```

0 **ALERT level A** = Most likely a serious problem - resolve or explain
0 **ALERT level B** = A potentially serious problem, consider carefully
9 **ALERT level C** = Check. Ensure it is not caused by an omission or oversight
9 **ALERT level G** = General information/check it is not something unexpected

1 ALERT type 1 CIF construction/syntax error, inconsistent or missing data
9 ALERT type 2 Indicator that the structure model may be wrong or deficient
1 ALERT type 3 Indicator that the structure quality may be low
7 ALERT type 4 Improvement, methodology, query or suggestion
0 ALERT type 5 Informative message, check

It is advisable to attempt to resolve as many as possible of the alerts in all categories. Often the minor alerts point to easily fixed oversights, errors and omissions in your CIF or refinement strategy, so attention to these fine details can be worthwhile. In order to resolve some of the more serious problems it may be necessary to carry out additional measurements or structure refinements. However, the purpose of your study may justify the reported deviations and the more serious of these should normally be commented upon in the discussion or experimental section of a paper or in the "special_details" fields of the CIF. checkCIF was carefully designed to identify outliers and unusual parameters, but every test has its limitations and alerts that are not important in a particular case may appear. Conversely, the absence of alerts does not guarantee there are no aspects of the results needing attention. It is up to the individual to critically assess their own results and, if necessary, seek expert advice.

Publication of your CIF in IUCr journals

A basic structural check has been run on your CIF. These basic checks will be run on all CIFs submitted for publication in IUCr journals (*Acta Crystallographica*, *Journal of Applied Crystallography*, *Journal of Synchrotron Radiation*); however, if you intend to submit to *Acta Crystallographica Section C* or *E* or *IUCrData*, you should make sure that full publication checks are run on the final version of your CIF prior to submission.

Publication of your CIF in other journals

Please refer to the *Notes for Authors* of the relevant journal for any special instructions relating to CIF submission.

PLATON version of 12/09/2022; check.def file version of 09/08/2022

Datablock EB42 - ellipsoid plot

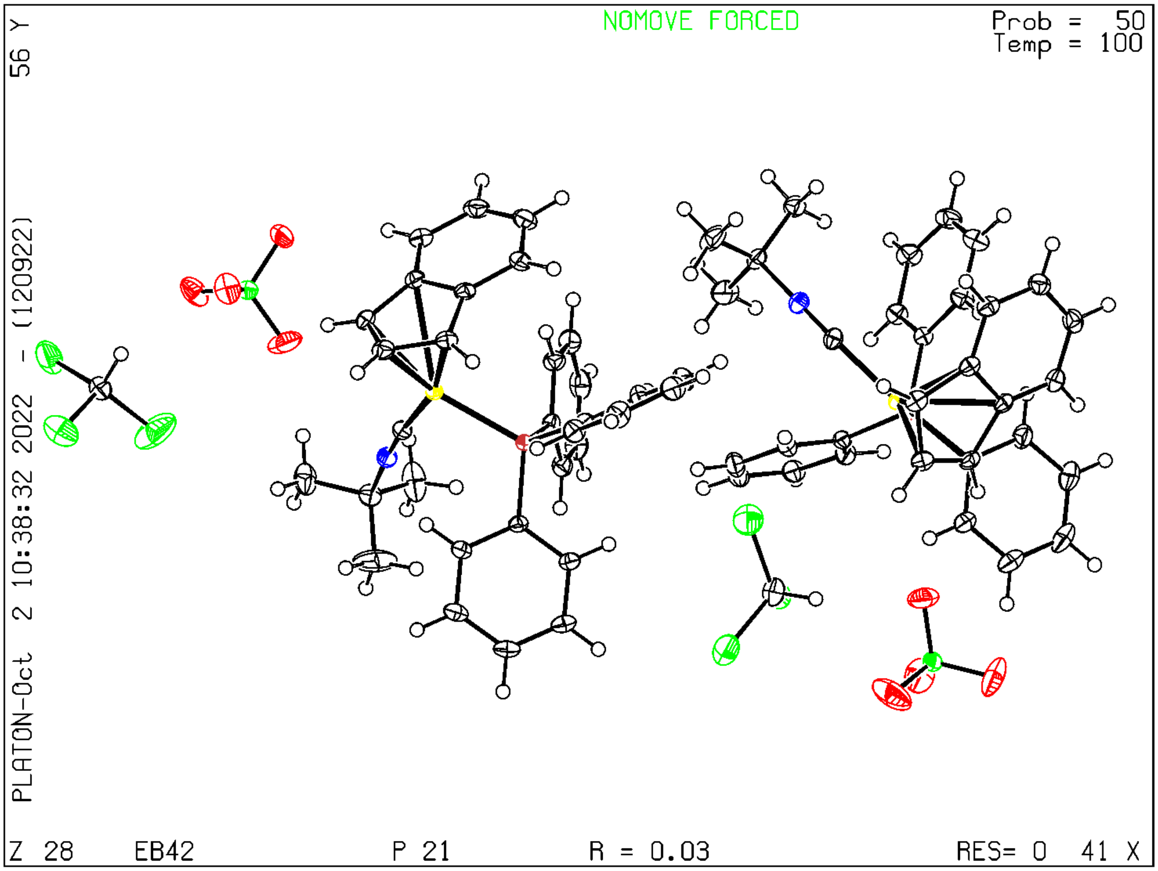

Supplement: Supplementary file 1 [file molecules-29-00345-s001.zip › CIF/EB42_checkcif.pdf]
