# Supplementary material for: Rational Design of Palladium(II) Indenyl and Allyl Complexes Bearing Phosphine and Isocyanide Ancillary Ligands with Promising Antitumor Activity
Source: Molecules. 2024 Jan 10;29(2):345. doi: 10.3390/molecules29020345 (PMC10819880; doi:10.3390/molecules29020345)

checkCIF/PLATON report

Structure factors have been supplied for datablock(s) EB47

THIS REPORT IS FOR GUIDANCE ONLY. IF USED AS PART OF A REVIEW PROCEDURE FOR PUBLICATION, IT SHOULD NOT REPLACE THE EXPERTISE OF AN EXPERIENCED CRYSTALLOGRAPHIC REFEREE.

No syntax errors found. CIF dictionary Interpreting this report

Datablock: EB47

```
Bond precision:   C-C = 0.0047 A          Wavelength=0.62000

Cell:               a=10.170(2)      b=18.904(4)        c=17.274(3)
                    alpha=90         beta=90.09(3)      gamma=90
Temperature:        298 K

                 Calculated                       Reported
Volume           3321.0(11)                       3321.0(11)
Space group      P 21/n                           P 21/n
Hall group       -P 2yn                           -P 2yn
Moiety formula   C32 H28 Cl3 N P Pd, Cl O4        C32 H28 Cl3 N P Pd, Cl O4
Sum formula      C32 H28 Cl4 N O4 P Pd            C32 H28 Cl4 N O4 P Pd
Mr               769.72                           769.72
Dx,g cm-3        1.539                            1.539
Z                4                                4
Mu (mm-1)        0.656                            0.656
F000             1552.0                           1552.0
F000'            1549.01
h,k,lmax         16,31,28                         16,31,26
Nref             16067                            14452
Tmin,Tmax        0.961,0.987
Tmin'            0.937

Correction method= Not given

Data completeness= 0.899          Theta(max)= 31.096

R(reflections)= 0.0591( 8661)                       wR2(reflections)=
                                                    0.2077( 14452)
S = 1.031                  Npar= 404
```

The following ALERTS were generated. Each ALERT has the format
test-name_ALERT_alert-type_alert-level.
Click on the hyperlinks for more details of the test.

Alert level C

```
PLAT220_ALERT_2_C NonSolvent   Resd 1  C   Ueq(max)/Ueq(min) Range        3.4 Ratio
PLAT242_ALERT_2_C Low    'MainMol' Ueq as Compared to Neighbors of      C16_4 Check
PLAT242_ALERT_2_C Low    'MainMol' Ueq as Compared to Neighbors of       C4_4 Check
PLAT260_ALERT_2_C Large Average Ueq of Residue Including      Cl_5      0.230 Check
PLAT334_ALERT_2_C Small <C-C> Benzene Dist.   C18_4    -C13_4    .       1.37 Ang.
PLAT911_ALERT_3_C Missing FCF Refl Between Thmin & STh/L=    0.600         23 Report
PLAT976_ALERT_2_C Check Calcd Resid. Dens.  0.86Ang From O4_5    .      -0.43 eA-3
```

Alert level G

```
ABSMU01_ALERT_1_G  Calculation of _exptl_absorpt_correction_mu
                not performed for this radiation type.
PLAT002_ALERT_2_G Number of Distance or Angle Restraints on AtSite          8 Note
PLAT092_ALERT_4_G Check: Wavelength Given is not Cu,Ga,Mo,Ag,In Ka    0.62000 Ang.
PLAT168_ALERT_4_G The CIF-Embedded .res File Contains EXYZ Records          1 Report
PLAT171_ALERT_4_G The CIF-Embedded .res File Contains EADP Records          1 Report
PLAT175_ALERT_4_G The CIF-Embedded .res File Contains SAME Records          1 Report
PLAT244_ALERT_4_G Low    'Solvent' Ueq as Compared to Neighbors of       Cl_5 Check
PLAT301_ALERT_3_G Main Residue  Disorder .............(Resd  1  )        11% Note
PLAT432_ALERT_2_G Short Inter X...Y Contact  C8_3     ..C3B_2    .       3.09 Ang.
                                        3/2-x,-1/2+y,3/2-z  =      2_646 Check
PLAT720_ALERT_4_G Number of Unusual/Non-Standard Labels ..........         84 Note
PLAT721_ALERT_1_G Bond    Calc     0.97000, Rep     0.96000 Dev...       0.01 Ang.
              C3B_2    -H3E_2          1_555   1_555 ........     #   87 Check
PLAT860_ALERT_3_G Number of Least-Squares Restraints ............          6 Note
PLAT912_ALERT_4_G Missing # of FCF Reflections Above STh/L=  0.600       1459 Note
PLAT933_ALERT_2_G Number of HKL-OMIT Records in Embedded .res File          5 Note
PLAT952_ALERT_5_G Calculated (ThMax) and CIF-Reported Lmax Differ.          2 Units
PLAT958_ALERT_1_G Calculated (ThMax) and Actual (FCF) Lmax Differ.          2 Units
PLAT965_ALERT_2_G The SHELXL WEIGHT Optimisation has not Converged     Please Check
PLAT978_ALERT_2_G Number C-C Bonds with Positive Residual Density.          1 Info
```

0 **ALERT level A** = Most likely a serious problem - resolve or explain
0 **ALERT level B** = A potentially serious problem, consider carefully
7 **ALERT level C** = Check. Ensure it is not caused by an omission or oversight
18 **ALERT level G** = General information/check it is not something unexpected

3 ALERT type 1 CIF construction/syntax error, inconsistent or missing data
11 ALERT type 2 Indicator that the structure model may be wrong or deficient
3 ALERT type 3 Indicator that the structure quality may be low
7 ALERT type 4 Improvement, methodology, query or suggestion
1 ALERT type 5 Informative message, check

It is advisable to attempt to resolve as many as possible of the alerts in all categories. Often the minor alerts point to easily fixed oversights, errors and omissions in your CIF or refinement strategy, so attention to these fine details can be worthwhile. In order to resolve some of the more serious problems it may be necessary to carry out additional measurements or structure refinements. However, the purpose of your study may justify the reported deviations and the more serious of these should normally be commented upon in the discussion or experimental section of a paper or in the "special_details" fields of the CIF. checkCIF was carefully designed to identify outliers and unusual parameters, but every test has its limitations and alerts that are not important in a particular case may appear. Conversely, the absence of alerts does not guarantee there are no aspects of the results needing attention. It is up to the individual to critically assess their own results and, if necessary, seek expert advice.

Publication of your CIF in IUCr journals

A basic structural check has been run on your CIF. These basic checks will be run on all CIFs submitted for publication in IUCr journals (*Acta Crystallographica*, *Journal of Applied Crystallography*, *Journal of Synchrotron Radiation*); however, if you intend to submit to *Acta Crystallographica Section C* or *E* or *IUCrData*, you should make sure that full publication checks are run on the final version of your CIF prior to submission.

Publication of your CIF in other journals

Please refer to the *Notes for Authors* of the relevant journal for any special instructions relating to CIF submission.

PLATON version of 18/05/2022; check.def file version of 17/05/2022

Datablock EB47 - ellipsoid plot

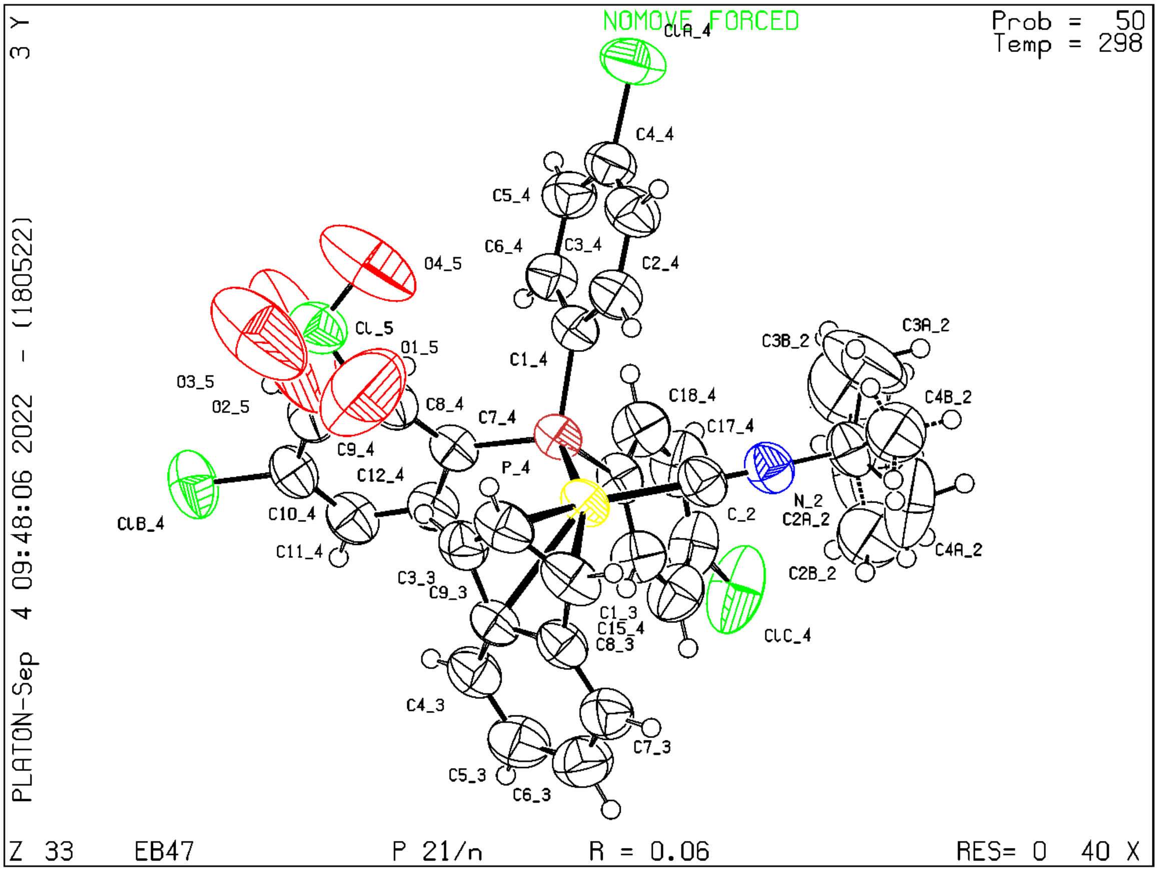

Supplement: Supplementary file 1 [file molecules-29-00345-s001.zip › CIF/EB47RT_checkcif.pdf]
