# Supplementary material for: Rational Design of Palladium(II) Indenyl and Allyl Complexes Bearing Phosphine and Isocyanide Ancillary Ligands with Promising Antitumor Activity
Source: Molecules. 2024 Jan 10;29(2):345. doi: 10.3390/molecules29020345 (PMC10819880; doi:10.3390/molecules29020345)

checkCIF/PLATON report

Structure factors have been supplied for datablock(s) EB48

THIS REPORT IS FOR GUIDANCE ONLY. IF USED AS PART OF A REVIEW PROCEDURE FOR PUBLICATION, IT SHOULD NOT REPLACE THE EXPERTISE OF AN EXPERIENCED CRYSTALLOGRAPHIC REFEREE.

No syntax errors found.   CIF dictionary   Interpreting this report

Datablock: EB48

Bond precision:	C-C = 0.0016 A		Wavelength=0.62000
Cell:	a=15.295(3)	b=10.691(2)	c=19.120(4)
	alpha=90	beta=99.74(3)	gamma=90
Temperature:	100 K		

	Calculated	Reported
Volume	3081.4(11)	3081.5(11)
Space group	P 21/c	P 21/c
Hall group	-P 2ybc	-P 2ybc
Moiety formula	C34 H30 F3 N P Pd, Cl O4	C34 H30 F3 N P Pd, Cl O4
Sum formula	C34 H30 Cl F3 N O4 P Pd	C34 H30 Cl F3 N O4 P Pd
Mr	746.41	746.41
Dx,g cm-3	1.609	1.609
Z	4	4
Mu (mm-1)	0.548	0.548
F000	1512.0	1512.0
F000’	1507.72	
h,k,lmax	25,17,31	25,17,31
Nref	14919	13566
Tmin,Tmax	0.968,0.989	
Tmin’	0.947	

Correction method= Not given

Data completeness= 0.909   Theta(max)= 31.098

R(reflections)= 0.0260(12785)   wR2(reflections)= 0.0741(13566)

S = 1.051   Npar= 406

The following ALERTS were generated. Each ALERT has the format
test-name_ALERT_alert-type_alert-level.
Click on the hyperlinks for more details of the test.

Alert level C

```
PLAT230_ALERT_2_C Hirshfeld Test Diff for    N_2       --C1_2     .        5.5 s.u.
PLAT244_ALERT_4_C Low    'Solvent' Ueq as Compared to Neighbors of       Cl_5 Check
PLAT911_ALERT_3_C Missing FCF Refl Between Thmin & STh/L=    0.600         36 Report
PLAT913_ALERT_3_C Missing # of Very Strong Reflections in FCF ....         11 Note
PLAT976_ALERT_2_C Check Calcd Resid. Dens.  0.71Ang From O3_5    .      -0.85 eA-3
```

Alert level G

```
ABSMU01_ALERT_1_G  Calculation of _exptl_absorpt_correction_mu
                  not performed for this radiation type.
PLAT092_ALERT_4_G Check: Wavelength Given is not Cu,Ga,Mo,Ag,In Ka    0.62000 Ang.
PLAT232_ALERT_2_G Hirshfeld Test Diff (M-X)  Pd_1     --C_2      .       10.8 s.u.
PLAT232_ALERT_2_G Hirshfeld Test Diff (M-X)  Pd_1     --C9_3     .        5.8 s.u.
PLAT232_ALERT_2_G Hirshfeld Test Diff (M-X)  Pd_1     --C8_3     .        5.8 s.u.
PLAT720_ALERT_4_G Number of Unusual/Non-Standard Labels .........          75 Note
PLAT912_ALERT_4_G Missing # of FCF Reflections Above STh/L=  0.600       1318 Note
PLAT933_ALERT_2_G Number of HKL-OMIT Records in Embedded .res File         22 Note
PLAT978_ALERT_2_G Number C-C Bonds with Positive Residual Density.         18 Info
```

0 **ALERT level A** = Most likely a serious problem - resolve or explain
0 **ALERT level B** = A potentially serious problem, consider carefully
5 **ALERT level C** = Check. Ensure it is not caused by an omission or oversight
9 **ALERT level G** = General information/check it is not something unexpected

1 ALERT type 1 CIF construction/syntax error, inconsistent or missing data
7 ALERT type 2 Indicator that the structure model may be wrong or deficient
2 ALERT type 3 Indicator that the structure quality may be low
4 ALERT type 4 Improvement, methodology, query or suggestion
0 ALERT type 5 Informative message, check

It is advisable to attempt to resolve as many as possible of the alerts in all categories. Often the minor alerts point to easily fixed oversights, errors and omissions in your CIF or refinement strategy, so attention to these fine details can be worthwhile. In order to resolve some of the more serious problems it may be necessary to carry out additional measurements or structure refinements. However, the purpose of your study may justify the reported deviations and the more serious of these should normally be commented upon in the discussion or experimental section of a paper or in the "special_details" fields of the CIF. checkCIF was carefully designed to identify outliers and unusual parameters, but every test has its limitations and alerts that are not important in a particular case may appear. Conversely, the absence of alerts does not guarantee there are no aspects of the results needing attention. It is up to the individual to critically assess their own results and, if necessary, seek expert advice.

Publication of your CIF in IUCr journals

A basic structural check has been run on your CIF. These basic checks will be run on all CIFs submitted for publication in IUCr journals (*Acta Crystallographica*, *Journal of Applied Crystallography*, *Journal of Synchrotron Radiation*); however, if you intend to submit to *Acta Crystallographica Section C* or *E* or *IUCrData*, you should make sure that full publication checks are run on the final version of your CIF prior to submission.

Publication of your CIF in other journals

Please refer to the *Notes for Authors* of the relevant journal for any special instructions relating to CIF submission.

PLATON version of 18/05/2022; check.def file version of 17/05/2022

Datablock EB48 - ellipsoid plot

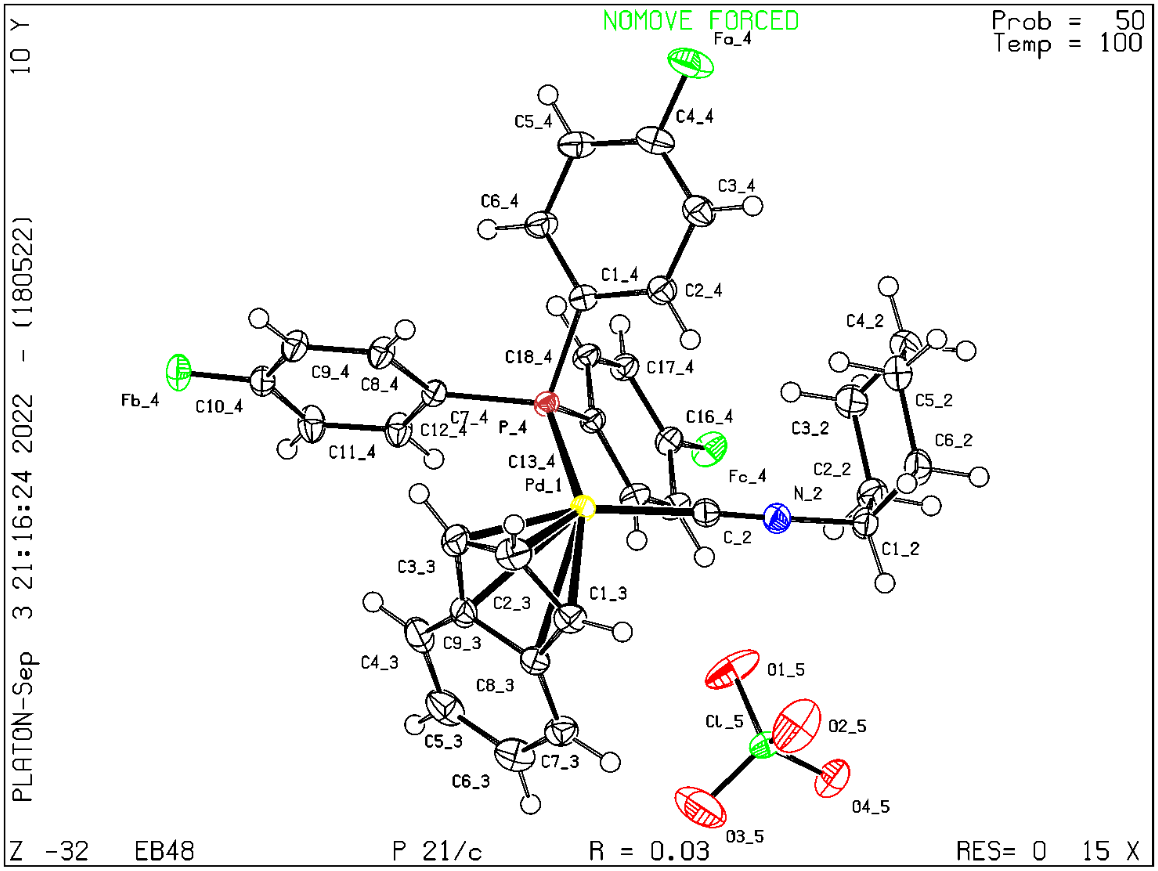

Supplement: Supplementary file 1 [file molecules-29-00345-s001.zip › CIF/EB48_checkcif.pdf]
