# Supplementary material for: Rational Design of Palladium(II) Indenyl and Allyl Complexes Bearing Phosphine and Isocyanide Ancillary Ligands with Promising Antitumor Activity
Source: Molecules. 2024 Jan 10;29(2):345. doi: 10.3390/molecules29020345 (PMC10819880; doi:10.3390/molecules29020345)

checkCIF/PLATON report

Structure factors have been supplied for datablock(s) EB52RT

THIS REPORT IS FOR GUIDANCE ONLY. IF USED AS PART OF A REVIEW PROCEDURE FOR PUBLICATION, IT SHOULD NOT REPLACE THE EXPERTISE OF AN EXPERIENCED CRYSTALLOGRAPHIC REFEREE.

No syntax errors found. CIF dictionary Interpreting this report

Datablock: EB52RT

```
Bond precision:   C-C = 0.0034 A          Wavelength=0.62000

Cell:            a=12.186(2)       b=12.335(2)       c=13.003(3)
                 alpha=83.54(3)    beta=81.05(3)     gamma=88.43(3)
Temperature:     298 K

                Calculated                       Reported
Volume          1918.4(7)                        1918.6(7)
Space group     P -1                             P -1
Hall group      -P 1                             -P 1
Moiety formula  C41 H43 N O3 P Pd, Cl O4         C41 H43 N O3 P Pd, Cl O4
Sum formula     C41 H43 Cl N O7 P Pd             C41 H43 Cl N O7 P Pd
Mr              834.58                           834.58
Dx,g cm-3       1.445                            1.445
Z               2                                2
Mu (mm-1)       0.444                            0.444
F000            860.0                            860.0
F000'           857.84
h,k,lmax                                         18,19,20
Nref                                             16038
Tmin,Tmax       0.974,0.991
Tmin'           0.957

Correction method= Not given

Data completeness=                    Theta(max)= 31.096

R(reflections)= 0.0405( 14298)                   wR2(reflections)=
                                                 0.1263( 16038)
S = 1.042                 Npar= 569
```

The following ALERTS were generated. Each ALERT has the format
test-name_ALERT_alert-type_alert-level.
Click on the hyperlinks for more details of the test.

Alert level C

```
PLAT241_ALERT_2_C High   'MainMol' Ueq as Compared to Neighbors of       C2_3 Check
PLAT242_ALERT_2_C Low    'MainMol' Ueq as Compared to Neighbors of       Pd_1 Check
PLAT242_ALERT_2_C Low    'MainMol' Ueq as Compared to Neighbors of       C1_2 Check
PLAT260_ALERT_2_C Large Average Ueq of Residue Including     ClA_5      0.240 Check
PLAT260_ALERT_2_C Large Average Ueq of Residue Including     ClB_5      0.261 Check
PLAT906_ALERT_3_C Large K Value in the Analysis of Variance ......      2.159 Check
PLAT911_ALERT_3_C Missing FCF Refl Between Thmin & STh/L=    0.600        138 Report
               11  0  0,  12  0  0,  13  0  0,  14  0  0, -14  1  0, -13  1  0,
              -12  1  0, -11  1  0, -10  1  0,  -9  1  0,  -4  1  0,   0  1  0,
                6  1  0,  13  1  0,  14  1  0, -14  2  0, -13  2  0, -12  2  0,
              -11  2  0, -10  2  0,  -9  2  0,  -6  2  0,  14  2  0, -14  3  0,
              -13  3  0, -12  3  0, -11  3  0, -10  3  0, -13  4  0, -12  4  0,
              -11  4  0, -13  5  0, -12  5  0,  -5  5  0,   1  5  0, -13  6  0,
                1 10  0,  -2 -6  1,   3 -6  1,   4 -4  1,  13 -4  1,  13 -3  1,
               14 -3  1, -14 -2  1, -13 -2  1,   8 -2  1,  12 -2  1,  13 -2  1,
               14 -2  1, -14 -1  1, -13 -1  1, -12 -1  1, -11 -1  1,  13 -1  1,
               14 -1  1, -14  0  1, -13  0  1, -12  0  1, -11  0  1, -10  0  1,
               -9  0  1,  -8  0  1,   5  0  1,  13  0  1,  14  0  1, -14  1  1,
              -13  1  1, -12  1  1, -11  1  1, -10  1  1,  -9  1  1,  -8  1  1,
              -14  2  1, -13  2  1, -12  2  1, -11  2  1, -10  2  1,  -9  2  1,
              -13  3  1, -12  3  1, -11  3  1, -10  3  1, -13  4  1, -12  4  1,
              -11  4  1, -13  5  1, -12  5  1,   3 -5  2, -13 -2  2,  -1 -2  2,
              -13 -1  2, -12 -1  2, -11 -1  2,  -6 -1  2,   0 -1  2, -14  0  2,
```

Alert level G

```
ABSMU01_ALERT_1_G  Calculation of _exptl_absorpt_correction_mu
                not performed for this radiation type.
PLAT002_ALERT_2_G Number of Distance or Angle Restraints on AtSite         27 Note
PLAT003_ALERT_2_G Number of Uiso or Uij Restrained non-H Atoms ...          2 Report
PLAT092_ALERT_4_G Check: Wavelength Given is not Cu,Ga,Mo,Ag,In Ka    0.62000 Ang.
PLAT154_ALERT_1_G The s.u.'s on the Cell Angles are Equal ..(Note)       0.03 Degree
PLAT175_ALERT_4_G The CIF-Embedded .res File Contains SAME Records          2 Report
PLAT176_ALERT_4_G The CIF-Embedded .res File Contains SADI Records          4 Report
PLAT177_ALERT_4_G The CIF-Embedded .res File Contains DELU Records          1 Report
PLAT232_ALERT_2_G Hirshfeld Test Diff (M-X)  Pd_1     --C_2      .        8.4 s.u.
PLAT301_ALERT_3_G Main Residue  Disorder .............(Resd  1  )        17% Note
PLAT302_ALERT_4_G Anion/Solvent/Minor-Residue Disorder (Resd  2  )       100% Note
PLAT302_ALERT_4_G Anion/Solvent/Minor-Residue Disorder (Resd  3  )       100% Note
PLAT304_ALERT_4_G Non-Integer Number of Atoms in ..... (Resd  2  )       3.47 Check
PLAT304_ALERT_4_G Non-Integer Number of Atoms in ..... (Resd  3  )       1.54 Check
PLAT410_ALERT_2_G Short Intra H...H Contact  H3_3     ..H8A_4    .       2.14 Ang.
                                                      x,y,z  =      1_555 Check
PLAT720_ALERT_4_G Number of Unusual/Non-Standard Labels ..........        115 Note
              Pd_1    N_2     C_2     C1_2    C2_2    H2A_2   H2B_2   C3_2
              H3A_2   H3B_2   C4_2    H4A_2   H4B_2   C5_2    H5_2    C6_2
              H6A_2   H6B_2   C7_2    H7_2    C8_2    H8A_2   H8B_2   C9_2
              H9_2    C10_2   H10A_2  H10B_2  ClA_5   O1A_5   O2A_5   O3A_5
              O4A_5   ClB_5   O1B_5   O2B_5   O3B_5   O4B_5   C4_3    H4_3
              C5_3    H5_3    C6_3    H6_3    C9_3    C3_3    H3_3    C8_3
```

```
              C7_3    H7_3    C1_3    H1_3    C2_3    H2_3    P_4     C1_4
              C6_4    H6_4    C5_4    H5_4    C4_4    C3_4    H3_4    C2_4
              H2_4    C13_4   C14_4   H14_4   C15_4   H15_4   C16_4   C17_4
              H17_4   C18_4   H18_4   Oa_4    Ca_4    Ha1_4   Ha2_4   Ha3_4
              Oc_4    Cc_4    Hc1_4   Hc2_4   Hc3_4   C12A_4  H12A_4  C11A_4
              H11A_4  C10A_4  C9A_4   H9A_4   C8A_4   H8A_4   C7A_4   ObA_4
              CbA_4   Hb1_4   Hb2_4   Hb3_4   C12B_4  H12B_4  C11B_4  H11B_4
              C10B_4  C9B_4   H9B_4   C8B_4   H8B_4   C7B_4   ObB_4   CbB_4
              Hb4_4   Hb5_4   Hb6_4
PLAT860_ALERT_3_G Number of Least-Squares Restraints .............         74 Note
PLAT912_ALERT_4_G Missing # of FCF Reflections Above STh/L=  0.600       2051 Note
PLAT933_ALERT_2_G Number of HKL-OMIT Records in Embedded .res File         10 Note
               -6  2  0,   5 -3  3,   3 -6  1,   1  3  2,  -1 -2  2,   1 -4  3,
                4 -4  1,   3 -3  3,   5  0  1,  -4  1  0,
PLAT950_ALERT_5_G Calculated (ThMax) and CIF-Reported Hmax Differ           2 Units
PLAT956_ALERT_1_G Calculated (ThMax) and Actual (FCF) Hmax Differ           2 Units
PLAT978_ALERT_2_G Number C-C Bonds with Positive Residual Density.         10 Info
PLAT992_ALERT_5_G Repd & Actual _reflns_number_gt Values Differ by          7 Check
```

```
   0 ALERT level A = Most likely a serious problem - resolve or explain
   0 ALERT level B = A potentially serious problem, consider carefully
   7 ALERT level C = Check. Ensure it is not caused by an omission or oversight
  23 ALERT level G = General information/check it is not something unexpected

   3 ALERT type 1 CIF construction/syntax error, inconsistent or missing data
  11 ALERT type 2 Indicator that the structure model may be wrong or deficient
   4 ALERT type 3 Indicator that the structure quality may be low
  10 ALERT type 4 Improvement, methodology, query or suggestion
   2 ALERT type 5 Informative message, check
```

It is advisable to attempt to resolve as many as possible of the alerts in all categories. Often the minor alerts point to easily fixed oversights, errors and omissions in your CIF or refinement strategy, so attention to these fine details can be worthwhile. In order to resolve some of the more serious problems it may be necessary to carry out additional measurements or structure refinements. However, the purpose of your study may justify the reported deviations and the more serious of these should normally be commented upon in the discussion or experimental section of a paper or in the "special_details" fields of the CIF. checkCIF was carefully designed to identify outliers and unusual parameters, but every test has its limitations and alerts that are not important in a particular case may appear. Conversely, the absence of alerts does not guarantee there are no aspects of the results needing attention. It is up to the individual to critically assess their own results and, if necessary, seek expert advice.

Publication of your CIF in IUCr journals

A basic structural check has been run on your CIF. These basic checks will be run on all CIFs submitted for publication in IUCr journals (*Acta Crystallographica*, *Journal of Applied Crystallography*, *Journal of Synchrotron Radiation*); however, if you intend to submit to *Acta Crystallographica Section C* or *E* or *IUCrData*, you should make sure that full publication checks are run on the final version of your CIF prior to submission.

Publication of your CIF in other journals

Please refer to the *Notes for Authors* of the relevant journal for any special instructions relating to CIF submission.

PLATON version of 14/11/2023; check.def file version of 14/09/2023

Datablock EB52RT - ellipsoid plot

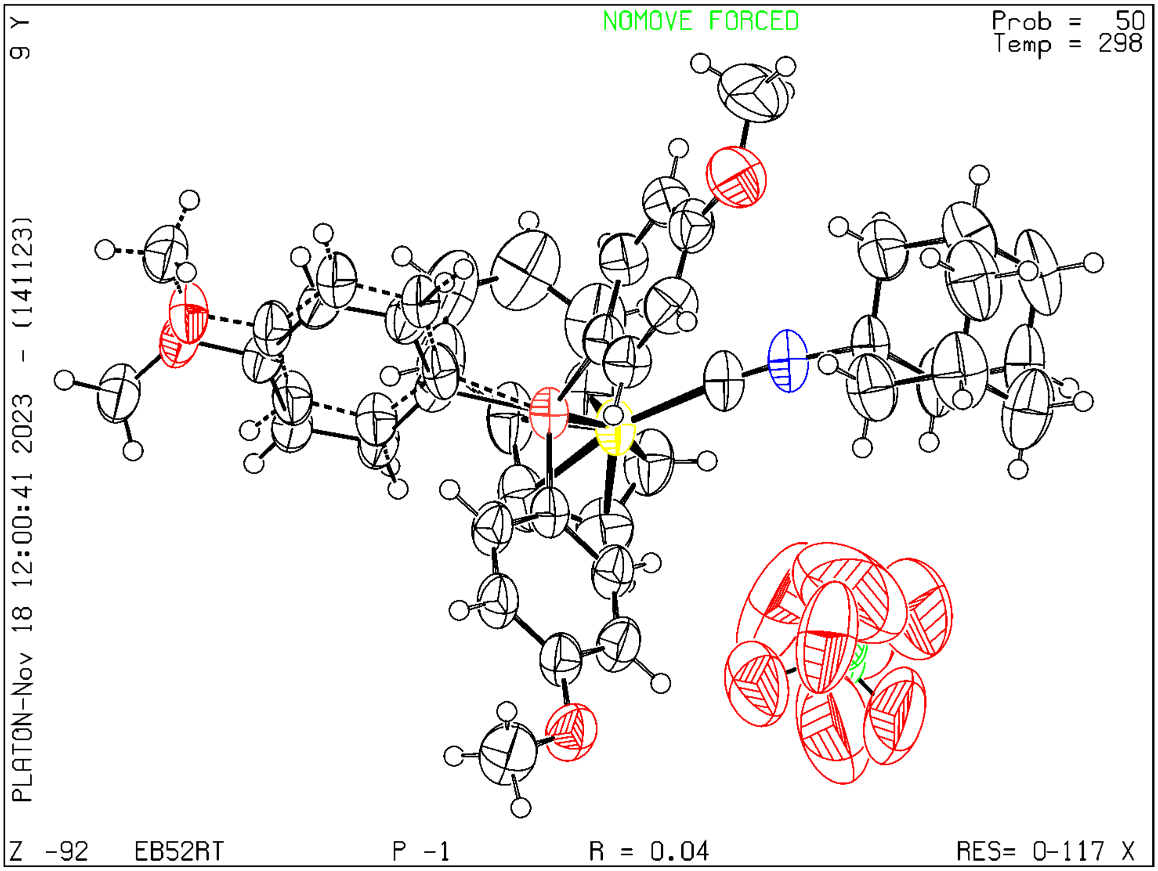

Supplement: Supplementary file 1 [file molecules-29-00345-s001.zip › CIF/EB52RT_checkcif.pdf]
