# Supplementary material for: Rational Design of Palladium(II) Indenyl and Allyl Complexes Bearing Phosphine and Isocyanide Ancillary Ligands with Promising Antitumor Activity
Source: Molecules. 2024 Jan 10;29(2):345. doi: 10.3390/molecules29020345 (PMC10819880; doi:10.3390/molecules29020345)

checkCIF/PLATON report

Structure factors have been supplied for datablock(s) EB6xRT

THIS REPORT IS FOR GUIDANCE ONLY. IF USED AS PART OF A REVIEW PROCEDURE FOR PUBLICATION, IT SHOULD NOT REPLACE THE EXPERTISE OF AN EXPERIENCED CRYSTALLOGRAPHIC REFEREE.

No syntax errors found. CIF dictionary Interpreting this report

Datablock: EB6xRT

```
Bond precision:   C-C = 0.0035 A          Wavelength=0.62000

Cell:              a=15.157(3)     b=13.336(3)        c=17.127(3)
                   alpha=90        beta=112.74(3)     gamma=90
Temperature:       298 K

                 Calculated                     Reported
Volume           3192.8(13)                     3192.8(13)
Space group      P 21/c                         P 21/c
Hall group       -P 2ybc                        -P 2ybc
Moiety formula   C34 H33 N P Pd, Cl O4          C34 H33 N P Pd, Cl O4
Sum formula      C34 H33 Cl N O4 P Pd           C34 H33 Cl N O4 P Pd
Mr               692.43                         692.43
Dx,g cm-3        1.441                          1.441
Z                4                              4
Mu (mm-1)        0.516                          0.516
F000             1416.0                         1416.0
F000'            1411.57
h,k,lmax                                        23,21,26
Nref                                            14031
Tmin,Tmax        0.970,0.990
Tmin'            0.950

Correction method= Not given

Data completeness=                 Theta(max)= 31.086

R(reflections)= 0.0351( 11737)                       wR2(reflections)=
                                                     0.1150( 14031)
S = 1.041                  Npar= 381
```

The following ALERTS were generated. Each ALERT has the format
test-name_ALERT_alert-type_alert-level.
Click on the hyperlinks for more details of the test.

Alert level C

```
PLAT220_ALERT_2_C NonSolvent   Resd 1  C   Ueq(max)/Ueq(min) Range        3.4 Ratio
PLAT241_ALERT_2_C High   'MainMol' Ueq as Compared to Neighbors of       C4_2 Check
PLAT242_ALERT_2_C Low    'MainMol' Ueq as Compared to Neighbors of       Pd_1 Check
PLAT242_ALERT_2_C Low    'MainMol' Ueq as Compared to Neighbors of       C1_2 Check
PLAT260_ALERT_2_C Large Average Ueq of Residue Including      Cl_5      0.117 Check
PLAT911_ALERT_3_C Missing FCF Refl Between Thmin & STh/L=    0.600          23 Report
                 0  2  0,   1  1  0,   3  0  0,   1  1  1,   1  3  1,   3  6  1,
                 5  6  1,  -1  0  2,   0  1  2,   2  0  2,  -2  1  3,   0  2  3,
                -2  4  4,   0  0  4,   1  0  4,   2  0  4,   0  0  8,   1  0 10,
                -6  4 13,  -7  0 14,  -4  0 14,  -4  1 14,  -4  6 14,
PLAT913_ALERT_3_C Missing # of Very Strong Reflections in FCF ....           4 Note
                 0  2  0,   3  0  0,   2  0  2,   0  0  4,
```

Alert level G

```
ABSMU01_ALERT_1_G  Calculation of _exptl_absorpt_correction_mu
                 not performed for this radiation type.
PLAT003_ALERT_2_G Number of Uiso or Uij Restrained non-H Atoms ...           2 Report
PLAT092_ALERT_4_G Check: Wavelength Given is not Cu,Ga,Mo,Ag,In Ka    0.62000 Ang.
PLAT178_ALERT_4_G The CIF-Embedded .res File Contains SIMU Records           1 Report
PLAT232_ALERT_2_G Hirshfeld Test Diff (M-X)  Pd_1     --C_2      .         8.0 s.u.
PLAT244_ALERT_4_G Low    'Solvent' Ueq as Compared to Neighbors of       Cl_5 Check
PLAT720_ALERT_4_G Number of Unusual/Non-Standard Labels ..........          75 Note
               O4_5    Cl_5    O1_5    O3_5    O2_5    Pd_1    C4_3    H4_3
               C5_3    H5_3    C6_3    H6_3    C9_3    C3_3    H3_3    C8_3
               C7_3    H7_3    C1_3    H1_3    C2_3    H2_3    P_4     C12_4
               H12_4   C11_4   H11_4   C10_4   H10_4   C9_4    H9_4    C8_4
               H8_4    C7_4    C1_4    C6_4    H6_4    C5_4    H5_4    C4_4
               H4_4    C3_4    H3_4    C2_4    H2_4    C13_4   C14_4   H14_4
               C15_4   H15_4   C16_4   H16_4   C17_4   H17_4   C18_4   H18_4
               C1_2    H1_2    C2_2    H2A_2   H2B_2   C3_2    H3A_2   H3B_2
               C4_2    H4A_2   H4B_2   C5_2    H5A_2   H5B_2   C6_2    H6A_2
               H6B_2   N_2     C_2
PLAT790_ALERT_4_G Centre of Gravity not Within Unit Cell: Resd.  #           2 Note
               Cl O4
PLAT860_ALERT_3_G Number of Least-Squares Restraints ............            6 Note
PLAT910_ALERT_3_G Missing # of FCF Reflection(s) Below Theta(Min).           1 Note
                 1  0  0,
PLAT912_ALERT_4_G Missing # of FCF Reflections Above STh/L=  0.600        1157 Note
PLAT933_ALERT_2_G Number of HKL-OMIT Records in Embedded .res File          14 Note
                 3  0  0,   0  0  4,   0  2  0,  -2  4  4,   2  0  2,   0  1  2,
                 0  2  3,   1  0  0,   1  1  0,   1  1  1,   1  3  1,  -2  1  3,
                 5  6  1,   3  6  1,
PLAT950_ALERT_5_G Calculated (ThMax) and CIF-Reported Hmax Differ            2 Units
PLAT952_ALERT_5_G Calculated (ThMax) and CIF-Reported Lmax Differ.           2 Units
PLAT956_ALERT_1_G Calculated (ThMax) and Actual (FCF) Hmax Differ            2 Units
PLAT958_ALERT_1_G Calculated (ThMax) and Actual (FCF) Lmax Differ.           2 Units
PLAT978_ALERT_2_G Number C-C Bonds with Positive Residual Density.          10 Info
PLAT992_ALERT_5_G Repd & Actual _reflns_number_gt Values Differ by           5 Check
```

```
  0 ALERT level A = Most likely a serious problem - resolve or explain
  0 ALERT level B = A potentially serious problem, consider carefully
  7 ALERT level C = Check. Ensure it is not caused by an omission or oversight
 18 ALERT level G = General information/check it is not something unexpected

  3 ALERT type 1 CIF construction/syntax error, inconsistent or missing data
  9 ALERT type 2 Indicator that the structure model may be wrong or deficient
  4 ALERT type 3 Indicator that the structure quality may be low
  6 ALERT type 4 Improvement, methodology, query or suggestion
  3 ALERT type 5 Informative message, check
```

It is advisable to attempt to resolve as many as possible of the alerts in all categories. Often the minor alerts point to easily fixed oversights, errors and omissions in your CIF or refinement strategy, so attention to these fine details can be worthwhile. In order to resolve some of the more serious problems it may be necessary to carry out additional measurements or structure refinements. However, the purpose of your study may justify the reported deviations and the more serious of these should normally be commented upon in the discussion or experimental section of a paper or in the "special_details" fields of the CIF. checkCIF was carefully designed to identify outliers and unusual parameters, but every test has its limitations and alerts that are not important in a particular case may appear. Conversely, the absence of alerts does not guarantee there are no aspects of the results needing attention. It is up to the individual to critically assess their own results and, if necessary, seek expert advice.

Publication of your CIF in IUCr journals

A basic structural check has been run on your CIF. These basic checks will be run on all CIFs submitted for publication in IUCr journals (*Acta Crystallographica*, *Journal of Applied Crystallography*, *Journal of Synchrotron Radiation*); however, if you intend to submit to *Acta Crystallographica Section C* or *E* or *IUCrData*, you should make sure that full publication checks are run on the final version of your CIF prior to submission.

Publication of your CIF in other journals

Please refer to the *Notes for Authors* of the relevant journal for any special instructions relating to CIF submission.

PLATON version of 14/11/2023; check.def file version of 14/09/2023

Datablock EB6xRT - ellipsoid plot

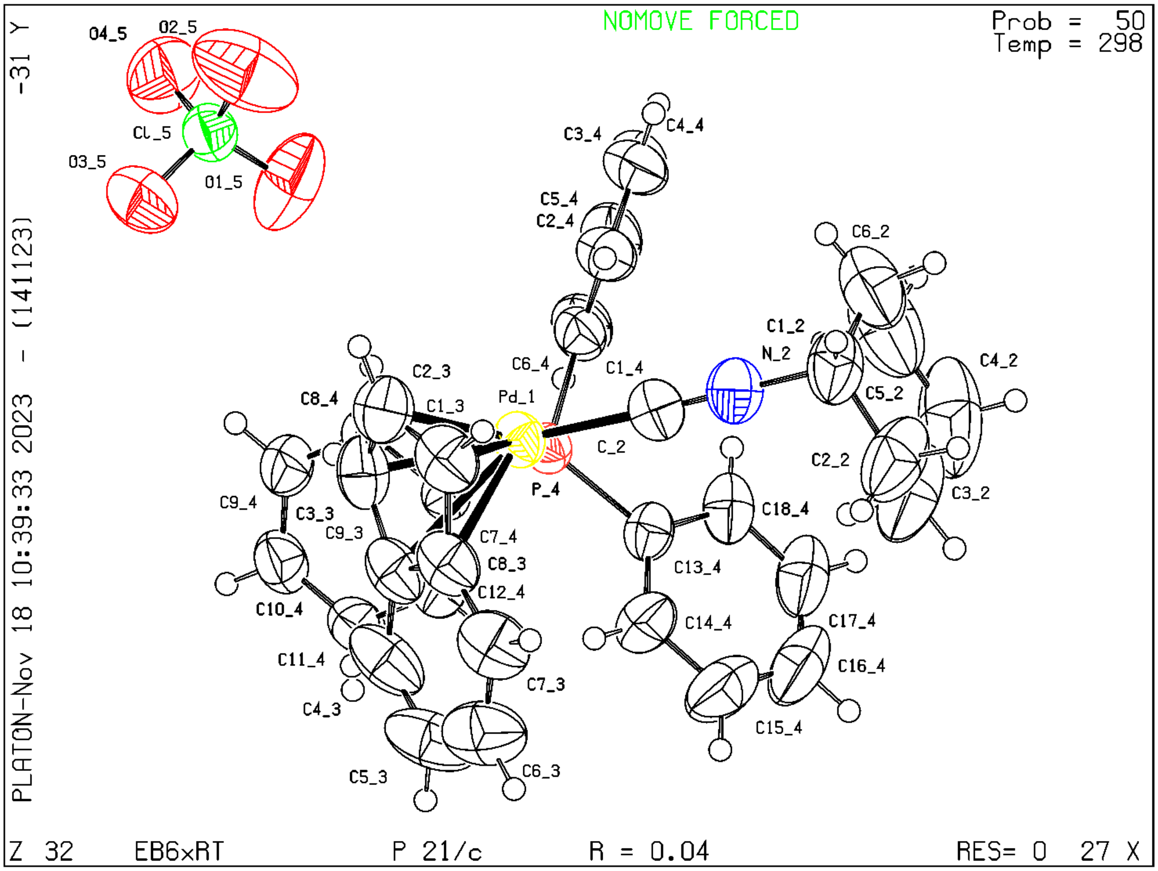

Supplement: Supplementary file 1 [file molecules-29-00345-s001.zip › CIF/EB6xRT_checkcif.pdf]
