# Supplementary material for: Rational Design of Palladium(II) Indenyl and Allyl Complexes Bearing Phosphine and Isocyanide Ancillary Ligands with Promising Antitumor Activity
Source: Molecules. 2024 Jan 10;29(2):345. doi: 10.3390/molecules29020345 (PMC10819880; doi:10.3390/molecules29020345)

checkCIF/PLATON report

Structure factors have been supplied for datablock(s) EB6x

THIS REPORT IS FOR GUIDANCE ONLY. IF USED AS PART OF A REVIEW PROCEDURE FOR PUBLICATION, IT SHOULD NOT REPLACE THE EXPERTISE OF AN EXPERIENCED CRYSTALLOGRAPHIC REFEREE.

No syntax errors found. CIF dictionary Interpreting this report

Datablock: EB6x

Bond precision: C-C = 0.0079 A Wavelength=0.62000

Cell:	a=14.982(3)	b=13.237(3)	c=16.829(3)
	alpha=90	beta=112.78(3)	gamma=90
Temperature:	100 K		

	Calculated	Reported
Volume	3077.1(13)	3077.2(12)
Space group	P 21/c	P 21/c
Hall group	-P 2ybc	-P 2ybc
Moiety formula	C34 H33 N P Pd, Cl O4	C34 H33 N P Pd, Cl O4
Sum formula	C34 H33 Cl N O4 P Pd	C34 H33 Cl N O4 P Pd
Mr	692.43	692.43
Dx,g cm-3	1.495	1.495
Z	4	4
Mu (mm-1)	0.535	0.535
F000	1416.0	1416.0
F000'	1411.57	
h,k,lmax		24,21,28
Nref		13007
Tmin,Tmax	0.968,0.989	
Tmin'	0.948	

Correction method= Not given

Data completeness= Theta(max)= 31.103

R(reflections)= 0.0746(11398) wR2(reflections)= 0.2458(13007)

S = 1.138 Npar= 409

The following ALERTS were generated. Each ALERT has the format
test-name_ALERT_alert-type_alert-level.
Click on the hyperlinks for more details of the test.

Alert level C

```
DIFMN02_ALERT_2_C  The minimum difference density is < -0.1*ZMAX*0.75
            _refine_diff_density_min given =     -4.082
            Test value =     -3.450
DIFMN03_ALERT_1_C  The minimum difference density is < -0.1*ZMAX*0.75
            The relevant atom site should be identified.
PLAT029_ALERT_3_C _diffrn_measured_fraction_theta_full value Low .      0.967 Why?
PLAT098_ALERT_2_C Large Reported Min.  (Negative) Residual Density      -4.08 eA-3
PLAT213_ALERT_2_C Atom C4_4             has ADP max/min Ratio .....       3.3 prolat
PLAT213_ALERT_2_C Atom C16_4            has ADP max/min Ratio .....       3.5 prolat
PLAT213_ALERT_2_C Atom C17_4            has ADP max/min Ratio .....       3.1 prolat
PLAT213_ALERT_2_C Atom C1A_2            has ADP max/min Ratio .....       3.5 prolat
PLAT220_ALERT_2_C NonSolvent   Resd 1  C   Ueq(max)/Ueq(min) Range        3.5 Ratio
PLAT244_ALERT_4_C Low    'Solvent' Ueq as Compared to Neighbors of       Cl_5 Check
PLAT906_ALERT_3_C Large K Value in the Analysis of Variance ......      2.512 Check
PLAT911_ALERT_3_C Missing FCF Refl Between Thmin & STh/L=    0.600        185 Report
                 2  2  0,   3  0  0,   3  1  0,   3  3  0,   5  0  0,   5  3  0,
                 5  4  0,   6  0  0,   6  5  0,   7  6  0,   8  0  0,   8  1  0,
                 8  6  0,   9  0  0,  10  0  0,  10  1  0,  10  6  0,  11  0  0,
                11  1  0,  12  0  0,  12  1  0,  12  2  0,  12  3  0,  12  4  0,
                13  6  0,  14  0  0,  14  1  0,  14  2  0,  14  7  0,  15  2  0,
                16  0  0,  16  1  0,  16  3  0,   5  0  2,  13  3  2, -11  3  3,
                -8  5  3,  -7  3  3,  -6  1  3,  -6  3  3,  -6 14  3,  -5  2  3,
                -5  4  3,  -5 10  3,  -4  2  3,  -4  5  3,  -4 13  3,  -3  2  3,
                -3  3  3,  -3  9  3,  -3 10  3,  -2  8  3,  -2 10  3,   0  1  3,
                 0  3  3,   0  6  3,   0  7  3,   1  7  3,   2  4  3,   2 15  3,
                 3  3  3,   3  5  3,   3  9  3,   4  4  3,   4  5  3,   4  9  3,
                 5  2  3,   5  8  3,   5 10  3,   6  8  3,   6 12  3,   7  8  3,
                 7  9  3,  -6  4  4,   0  0  4,  -5  3  5,   1  9  5,   2  5  5,
                 4  9  5,  -6 11  6,  -3 15  6,   8  0  6,  12  0  6,  -5  9  7,
                -3 14  7, -17  3  8, -16  0  8, -16  1  8, -14  3  8, -12  0  8,
               -10  0  8,  -9 12  8,  -8  8  8,  -7  0  8,  -7 12  8,   1  7  8,
PLAT918_ALERT_3_C Reflection(s) with I(obs) much Smaller I(calc) .          4 Check
```

Alert level G

```
ABSMU01_ALERT_1_G  Calculation of _exptl_absorpt_correction_mu
                not performed for this radiation type.
PLAT002_ALERT_2_G Number of Distance or Angle Restraints on AtSite         16 Note
PLAT003_ALERT_2_G Number of Uiso or Uij Restrained non-H Atoms ...          5 Report
PLAT083_ALERT_2_G SHELXL Second Parameter in WGHT  Unusually Large       9.70 Why ?
PLAT092_ALERT_4_G Check: Wavelength Given is not Cu,Ga,Mo,Ag,In Ka    0.62000 Ang.
PLAT168_ALERT_4_G The CIF-Embedded .res File Contains EXYZ Records          1 Report
PLAT171_ALERT_4_G The CIF-Embedded .res File Contains EADP Records          1 Report
PLAT172_ALERT_4_G The CIF-Embedded .res File Contains DFIX Records          2 Report
PLAT173_ALERT_4_G The CIF-Embedded .res File Contains DANG Records          1 Report
PLAT175_ALERT_4_G The CIF-Embedded .res File Contains SAME Records          1 Report
PLAT176_ALERT_4_G The CIF-Embedded .res File Contains SADI Records          2 Report
PLAT178_ALERT_4_G The CIF-Embedded .res File Contains SIMU Records          1 Report
PLAT301_ALERT_3_G Main Residue  Disorder .............(Resd  1  )         22% Note
PLAT720_ALERT_4_G Number of Unusual/Non-Standard Labels .........          94 Note
```

```
              O4_5    Cl_5    O1_5    O3_5    O2_5    Pd_1    C4_3    H4_3
              C5_3    H5_3    C6_3    H6_3    C9_3    C3_3    H3_3    C8_3
              C7_3    H7_3    C1_3    H1_3    C2_3    H2_3    P_4     C12_4
              H12_4   C11_4   H11_4   C10_4   H10_4   C9_4    H9_4    C8_4
              H8_4    C7_4    C1_4    C6_4    H6_4    C5_4    H5_4    C4_4
              H4_4    C3_4    H3_4    C2_4    H2_4    C13_4   C14_4   H14_4
              C15_4   H15_4   C16_4   H16_4   C17_4   H17_4   C18_4   H18_4
              C1A_2   H1A_2   C2A_2   H2A_2   H2B_2   C3A_2   H3A_2   H3B_2
              C4A_2   H4A_2   H4B_2   C5A_2   H5A_2   H5B_2   C6A_2   H6A_2
              H6B_2   Na_2    Ca_2    C1B_2   H1B_2   C2B_2   H2C_2   H2D_2
              C3B_2   H3C_2   H3D_2   C4B_2   H4C_2   H4D_2   C5B_2   H5C_2
              H5D_2   C6B_2   H6C_2   H6D_2   Nb_2    Cb_2
PLAT790_ALERT_4_G Centre of Gravity not Within Unit Cell: Resd.  #          2 Note
              Cl O4
PLAT860_ALERT_3_G Number of Least-Squares Restraints ............          67 Note
PLAT870_ALERT_4_G ALERTS Related to Twinning Effects Suppressed ..          ! Info
PLAT910_ALERT_3_G Missing # of FCF Reflection(s) Below Theta(Min).          1 Note
                1  0  0,
PLAT912_ALERT_4_G Missing # of FCF Reflections Above STh/L=  0.600       1691 Note
PLAT913_ALERT_3_G Missing # of Very Strong Reflections in FCF ....          2 Note
                3  0  0,   0  0  4,
PLAT931_ALERT_5_G CIFcalcFCF  Twin Law ( 1 0 0)         Est.d BASF       0.25 Check
PLAT933_ALERT_2_G Number of HKL-OMIT Records in Embedded .res File         23 Note
               -3  6 10,  -1 13 10,  -3  9  3,  -1  0 16,  -3 12 10,  -5 10  3,
               -3  3  3,  -3 10 10,  -3 14 10,  -6  0 16,  -1 11 10,  -8  0 18,
                0  7  3,  -6  2 13,   0  1  3,   3  9  3,   2  7 16,   5  2  3,
               -4 13 10,  -4 13  3,  -2  2 13,  -4  1 13,  -2  4 13,
PLAT941_ALERT_3_G Average HKL Measurement Multiplicity ..........        1.0 Low
PLAT965_ALERT_2_G The SHELXL WEIGHT Optimisation has not Converged     Please Check
PLAT992_ALERT_5_G Repd & Actual _reflns_number_gt Values Differ by          4 Check
```

```
  0 ALERT level A = Most likely a serious problem - resolve or explain
  0 ALERT level B = A potentially serious problem, consider carefully
 13 ALERT level C = Check. Ensure it is not caused by an omission or oversight
 25 ALERT level G = General information/check it is not something unexpected

  2 ALERT type 1 CIF construction/syntax error, inconsistent or missing data
 12 ALERT type 2 Indicator that the structure model may be wrong or deficient
  9 ALERT type 3 Indicator that the structure quality may be low
 13 ALERT type 4 Improvement, methodology, query or suggestion
  2 ALERT type 5 Informative message, check
```

It is advisable to attempt to resolve as many as possible of the alerts in all categories. Often the minor alerts point to easily fixed oversights, errors and omissions in your CIF or refinement strategy, so attention to these fine details can be worthwhile. In order to resolve some of the more serious problems it may be necessary to carry out additional measurements or structure refinements. However, the purpose of your study may justify the reported deviations and the more serious of these should normally be commented upon in the discussion or experimental section of a paper or in the "special_details" fields of the CIF. checkCIF was carefully designed to identify outliers and unusual parameters, but every test has its limitations and alerts that are not important in a particular case may appear. Conversely, the absence of alerts does not guarantee there are no aspects of the results needing attention. It is up to the individual to critically assess their own results and, if necessary, seek expert advice.

Publication of your CIF in IUCr journals

A basic structural check has been run on your CIF. These basic checks will be run on all CIFs submitted for publication in IUCr journals (*Acta Crystallographica*, *Journal of Applied Crystallography*, *Journal of Synchrotron Radiation*); however, if you intend to submit to *Acta Crystallographica Section C* or *E* or *IUCrData*, you should make sure that full publication checks are run on the final version of your CIF prior to submission.

Publication of your CIF in other journals

Please refer to the *Notes for Authors* of the relevant journal for any special instructions relating to CIF submission.

PLATON version of 14/11/2023; check.def file version of 14/09/2023

Datablock EB6x - ellipsoid plot

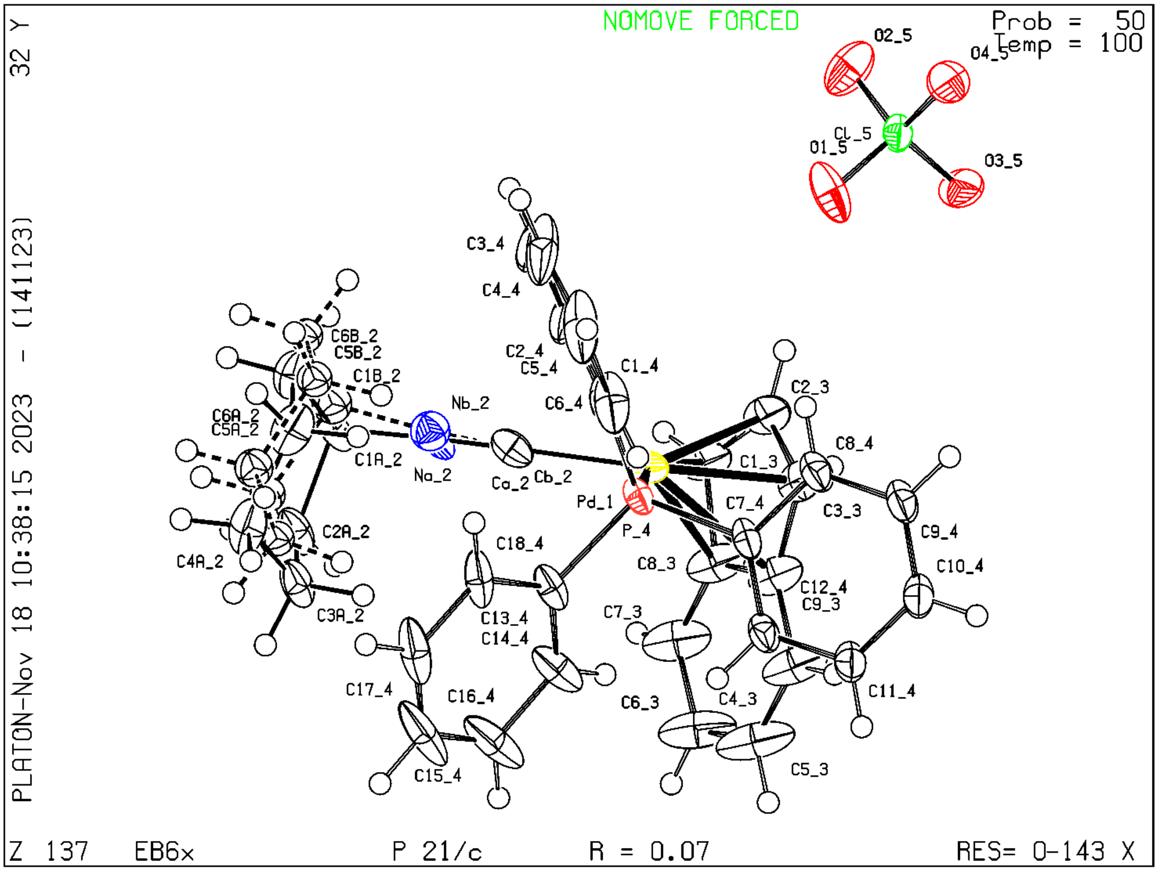

Supplement: Supplementary file 1 [file molecules-29-00345-s001.zip › CIF/EB6x_checkcif.pdf]
