# Supplementary material for: Rational Design of Palladium(II) Indenyl and Allyl Complexes Bearing Phosphine and Isocyanide Ancillary Ligands with Promising Antitumor Activity
Source: Molecules. 2024 Jan 10;29(2):345. doi: 10.3390/molecules29020345 (PMC10819880; doi:10.3390/molecules29020345)
Supplement: Supplementary file 1 [file molecules-29-00345-s001.zip › Supplementary Information.pdf]

Supplementary Information

Rational Design of Palladium(II) Indenyl and Allyl Complexes bearing Phosphine and Isocyanide Ancillary Ligands with Promising Antitumor Activity

Enrica Bortolamiol[1], Eleonora Botter[1], Enrico Cavarzerani[1], Matteo Mauceri[1], Nicola Demitri[2], Flavio Rizzolio[1,3], Fabiano Visentin[1*] and Thomas Scattolin[4*]

[1] Department of Molecular Sciences and Nanosystems Università Ca' Foscari Campus Scientifico Via Torino 155, 30174, Venezia-Mestre, Italy: enrica.bortolamiol@unive.it (E.B); eleonora.botter@unive.it (E.B); enrico.cavarzerani@unive.it (E.C.); matteo.mauceri@unive.it (M.M.); flavio.rizzolio@unive.it

[2] Area Science Park Elettra-Sincrotrone Trieste, S.S. 14 Km 163.5 Basovizza, 34149, Trieste, Italy; nicola.demitri@elettra.eu (N.D.)

[3] Pathology Unit, Department of Molecular Biology and Translational Research Centro di Riferimento Oncologico di Aviano (CRO) IRCCS via Franco Gallini 2, 33081, Aviano, Italy

[4] Dipartimento di Scienze Chimiche, Università degli Studi di Padova, via Marzolo 1, 35131 Padova, Italy

* Correspondence: thomas.scattolin@unipd.it (T.S); fvise@unive.it (F.V.)

Table of content

NMR and IR spectra 3

Hapticity evaluation 34

NMR reactivity data 35

HRMS data 36

Crystal Structure Determination 45

X-ray diffraction analysis - Structural characterization 53

References 58

NMR and IR spectra

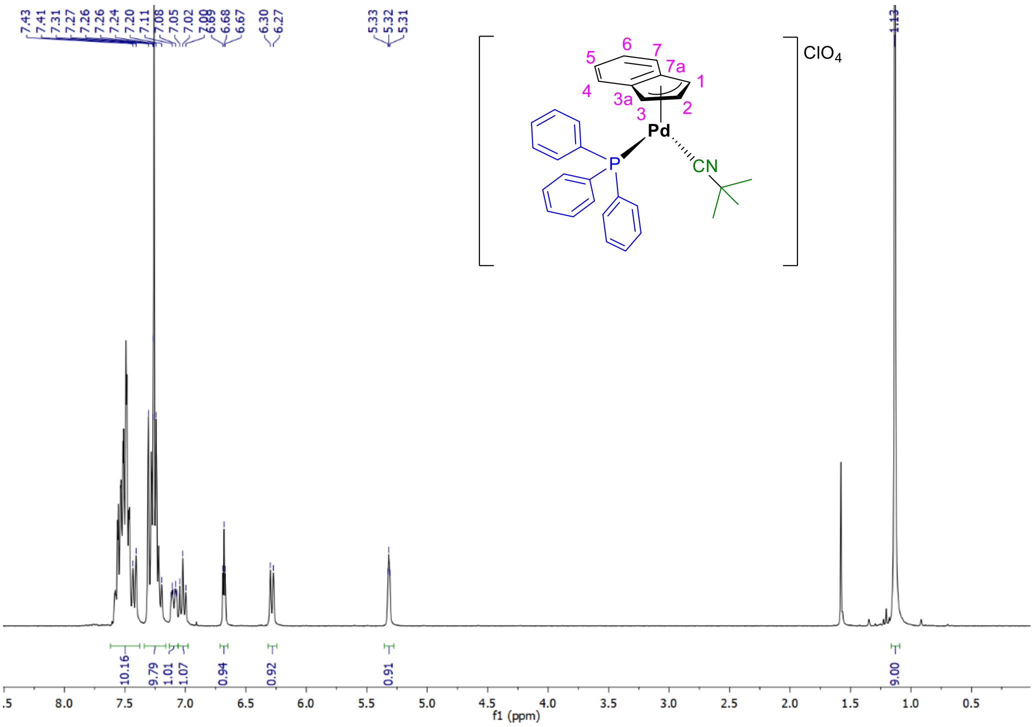

S 1. ^{1}H NMR spectra of compound **3a-Tic** in $CDCl_3$ at 298K

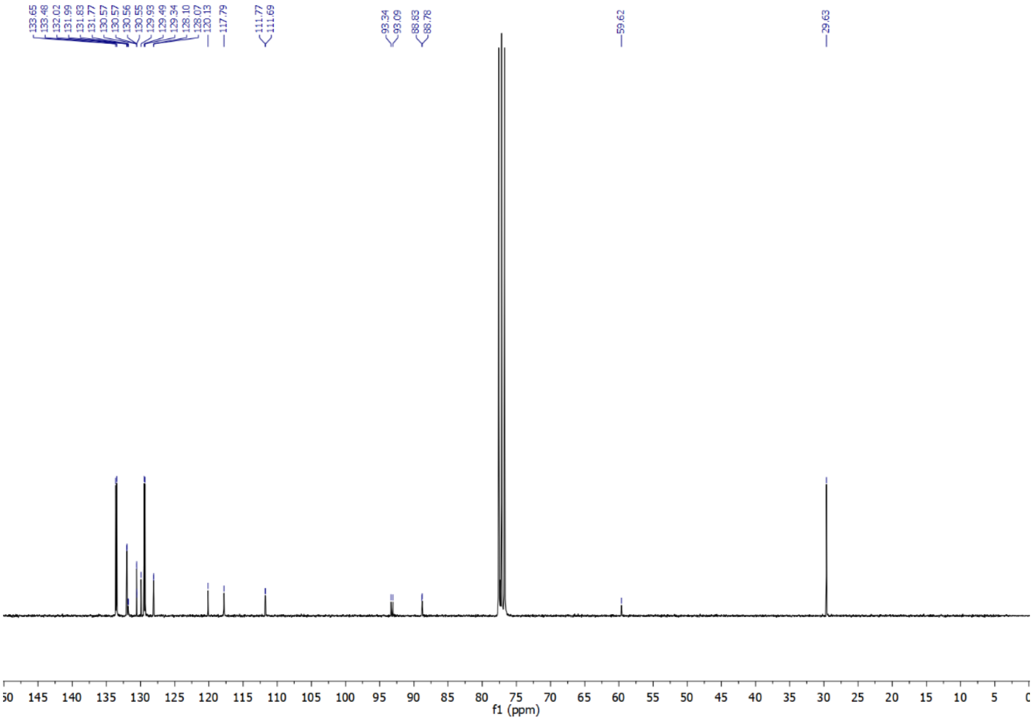

S 2. $^{13}C\{^{1}H\}$ NMR spectra of compound **3a-Tic** in $CDCl_3$ at 298K

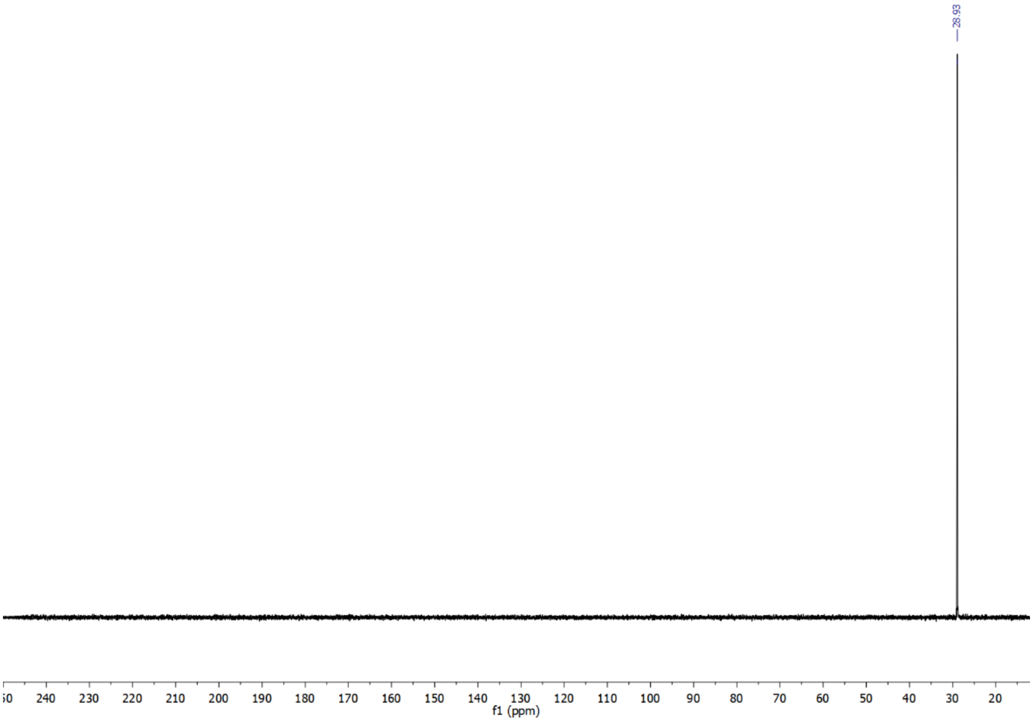

S 3. $^{31}P\{^{1}H\}$ *NMR spectra of compound* ***3a-Tic*** *in* $CDCl_3$ *at 298K*

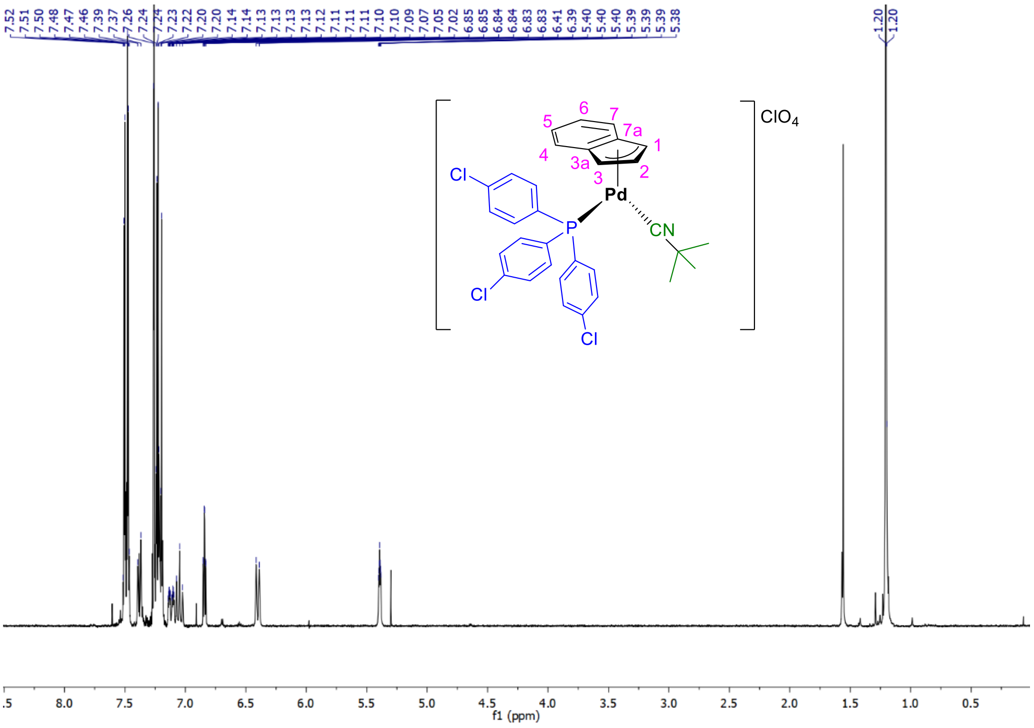

S 4. ^{1}H *NMR spectra of compound* ***3b-Tic*** *in* $CDCl_3$ *at 298K*

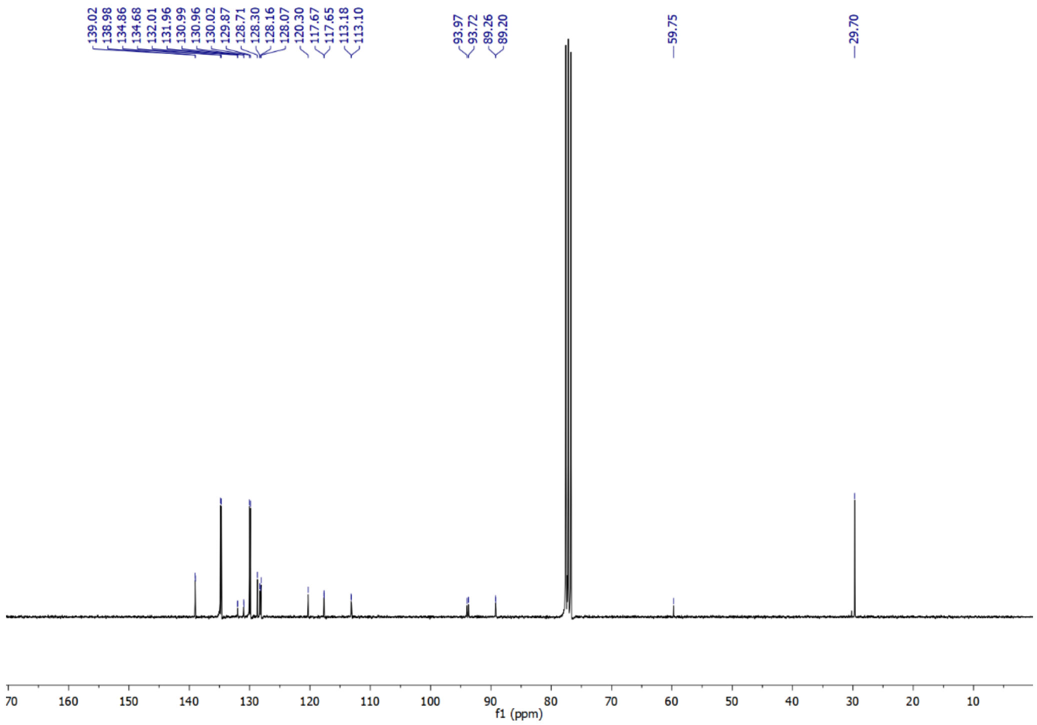

*S 5. $^{13}C\{^{1}H\}$ NMR spectra of compound **3b-Tic** in $CDCl_3$ at 298K*

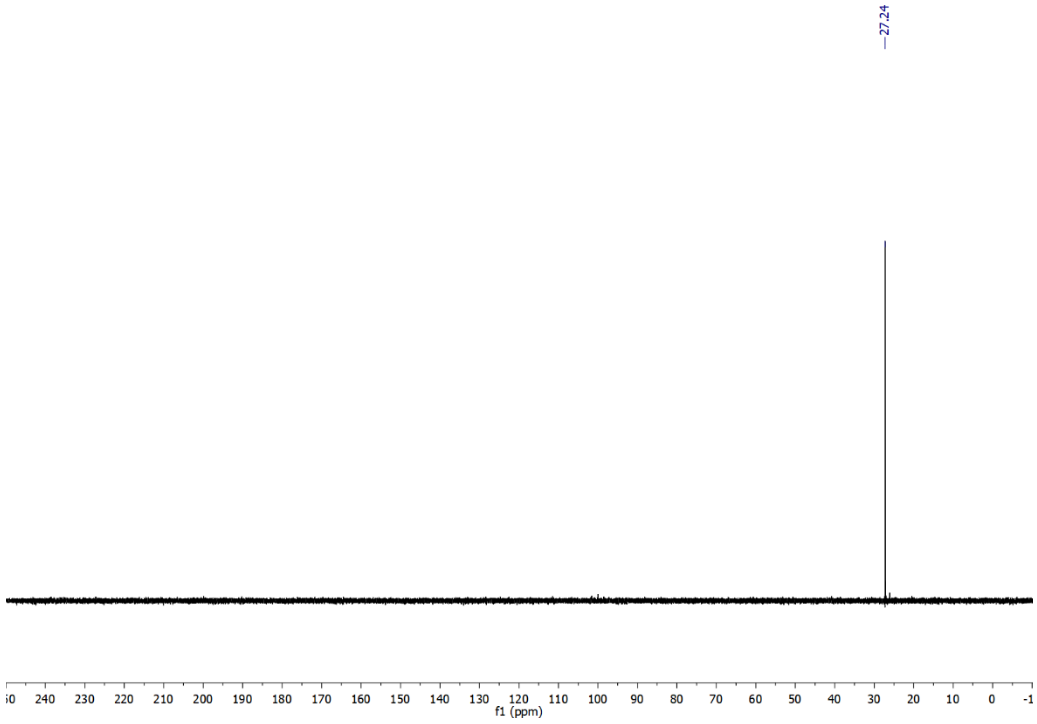

*S 6. $^{31}P\{^{1}H\}$ NMR spectra of compound **3b-Tic** in $CDCl_3$ at 298K*

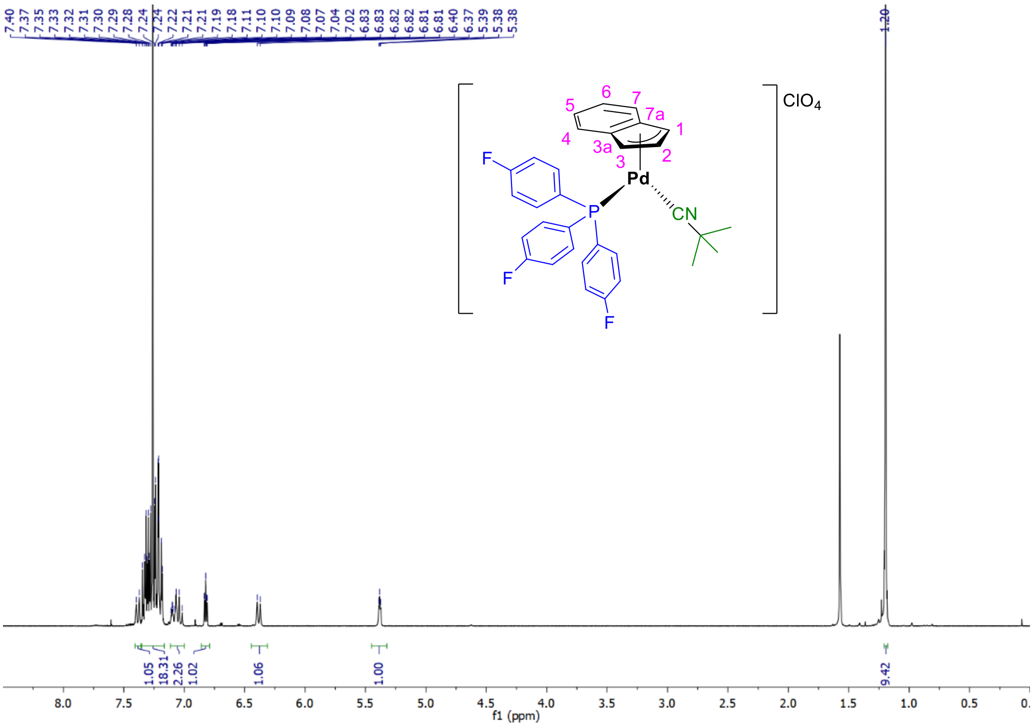

S 7. ¹H NMR spectra of compound ***3c-Tic*** *in CDCl₃ at 298K*

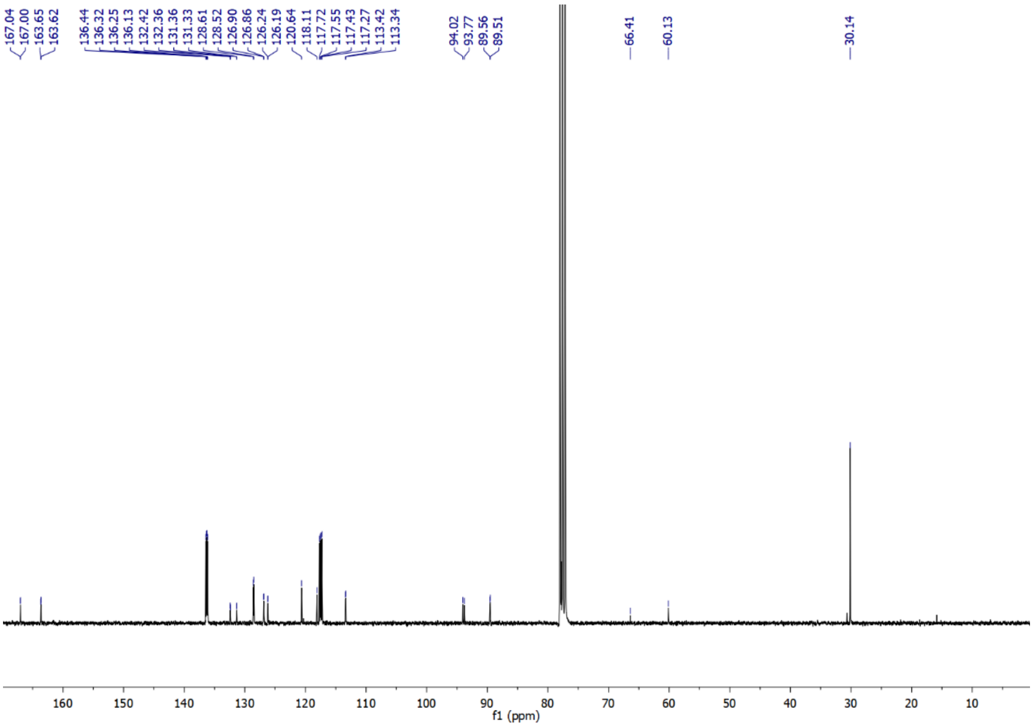

S 8. ¹³C{¹H} NMR spectra of compound ***3c-Tic*** *in CDCl₃ at 298K*

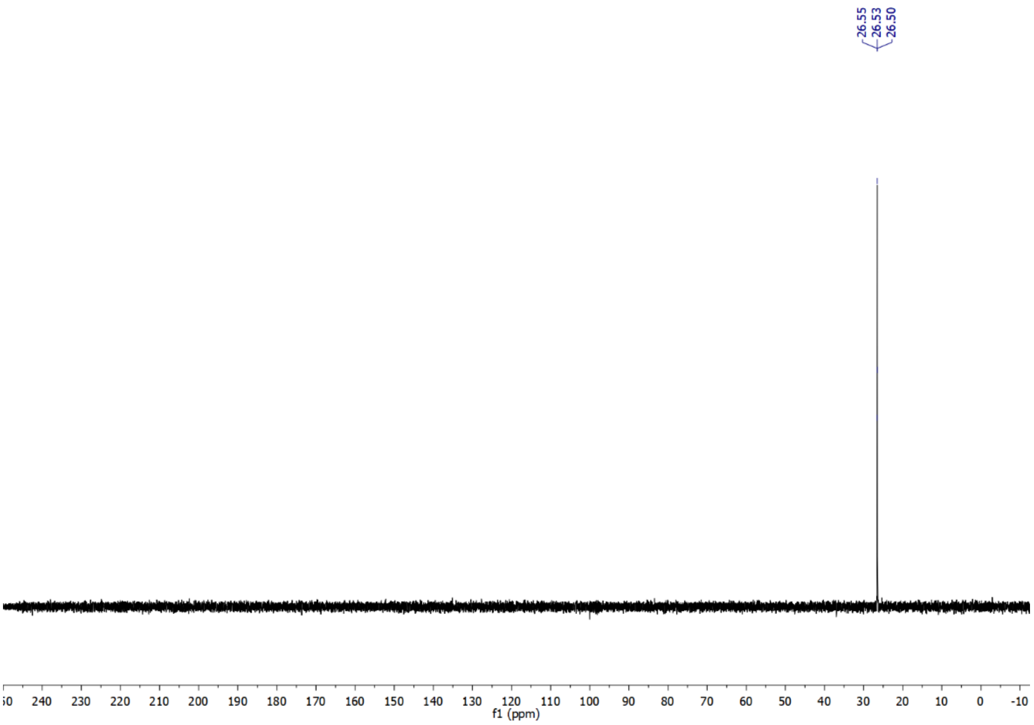

*S 9. $^{31}P\{^{1}H\}$ NMR spectra of compound **3c-Tic** in $CDCl_3$ at 298K*

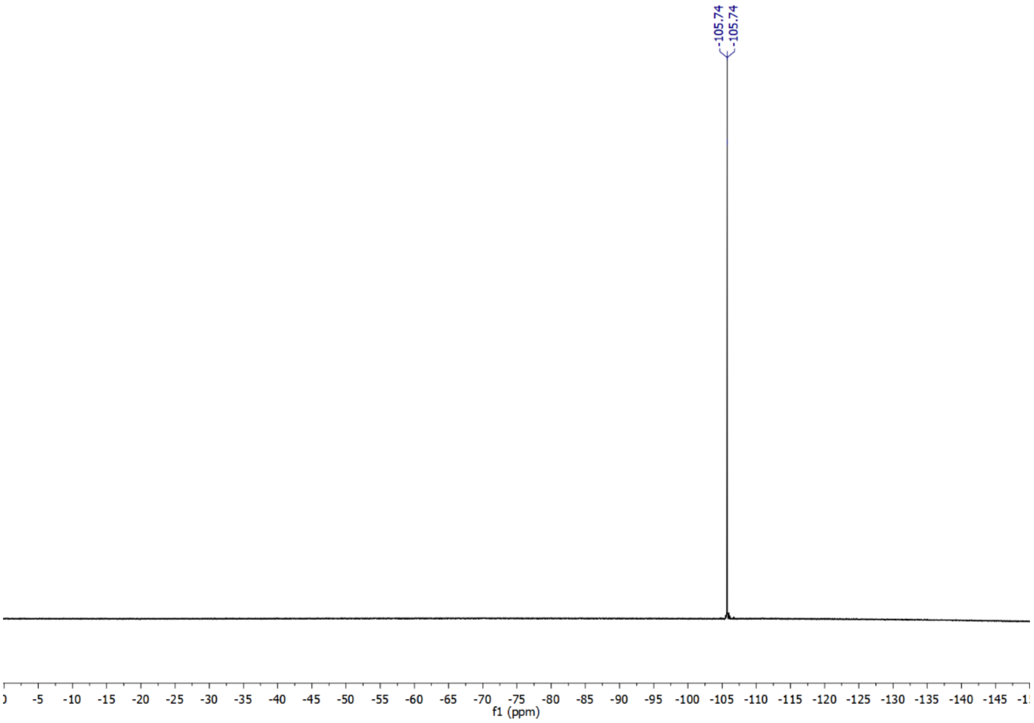

*S 10. $^{91}F\{^{1}H\}$ NMR spectra of compound **3c-Tic** in $CDCl_3$ at 298K*

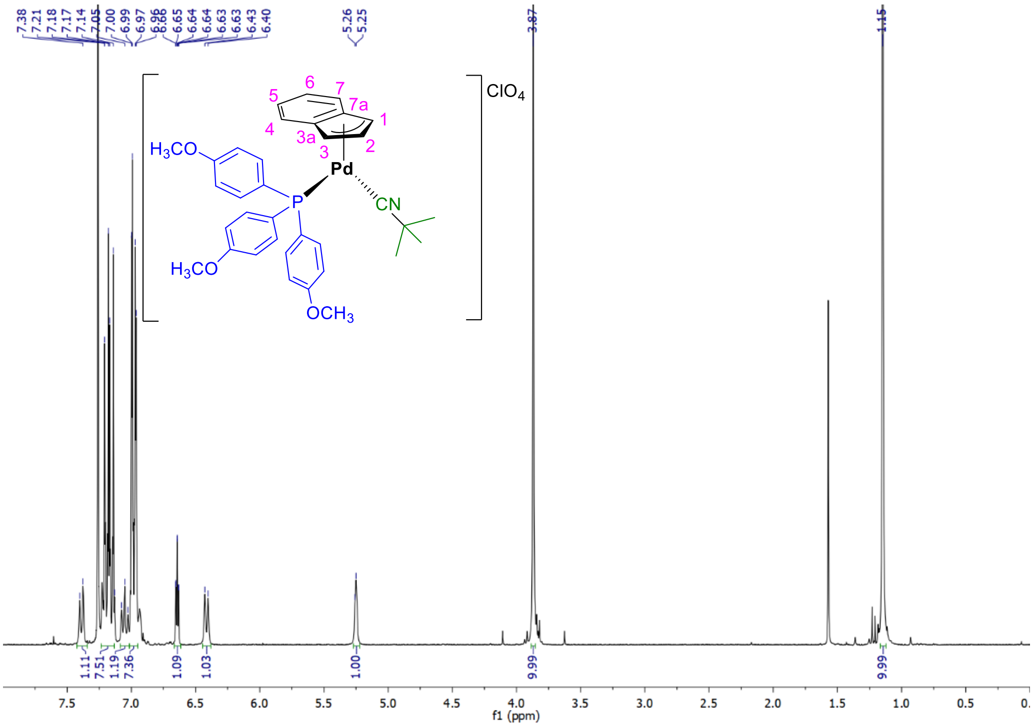

S 11. ^{1}H NMR spectra of compound **3d-Tic** in $CDCl_3$ at 298K

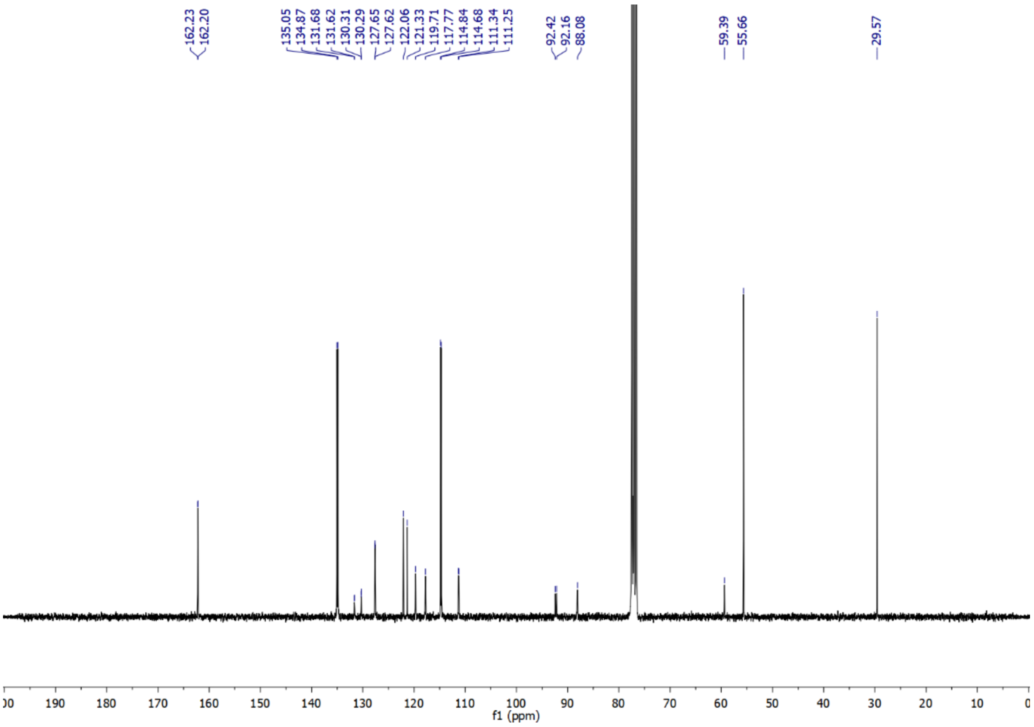

S 12. $^{13}C\{^{1}H\}$ NMR spectra of compound **3d-Tic** in $CDCl_3$ at 298K

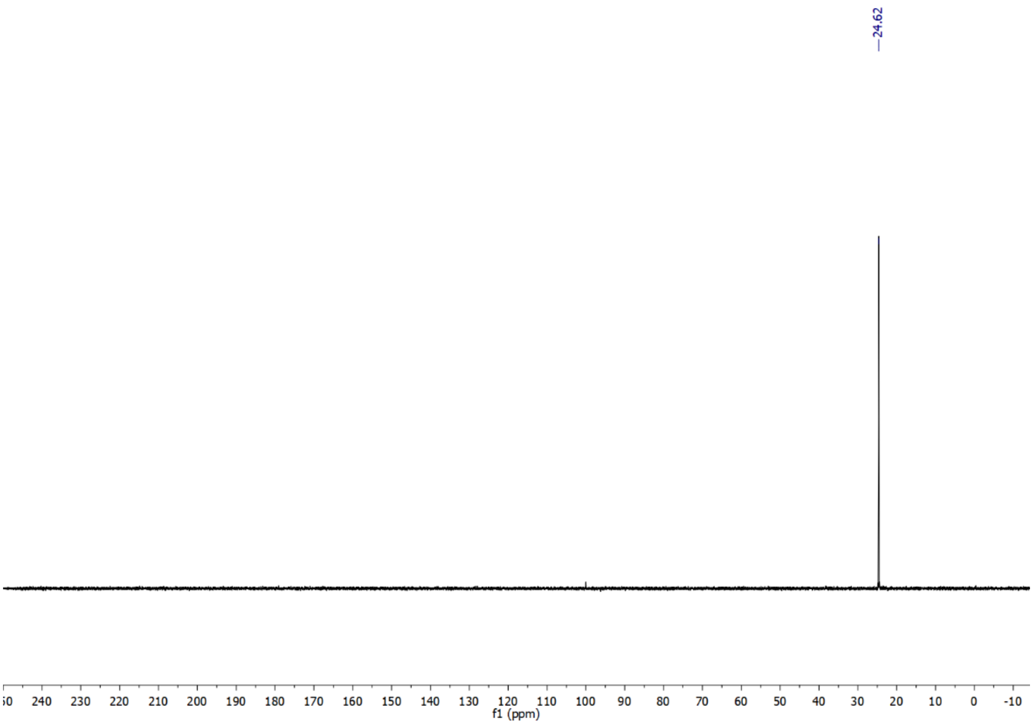

S 13. $^{31}P\{^{1}H\}$ NMR spectra of compound **3d-Tic** in $CDCl_3$ at 298K

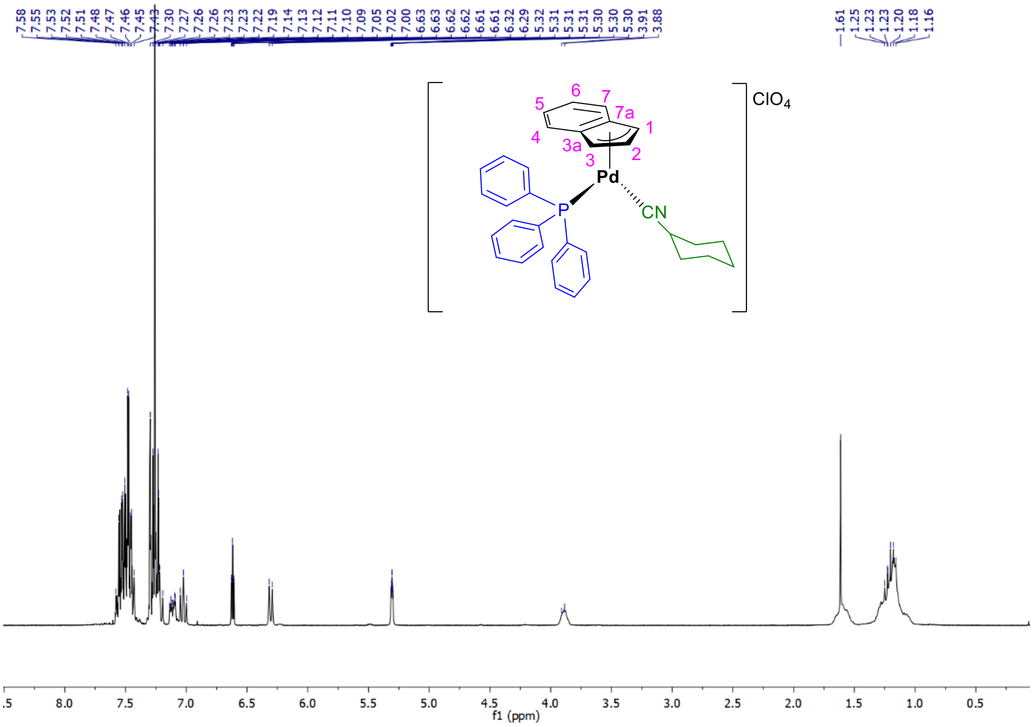

S 14. ^{1}H NMR spectra of compound **3a-Cyic** in $CDCl_3$ at 298K

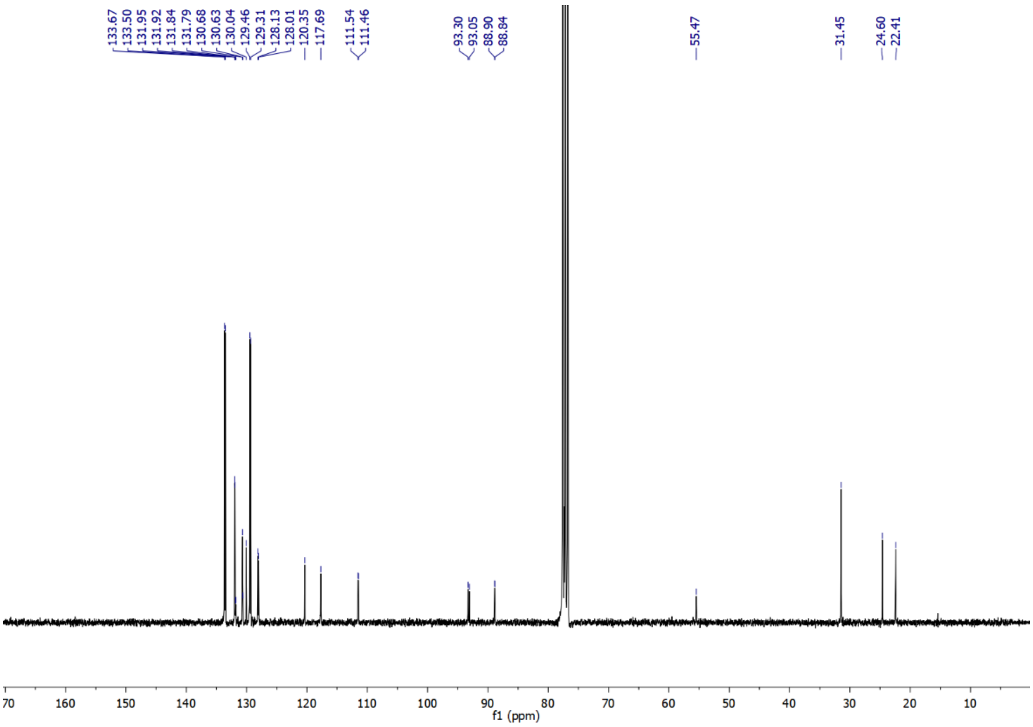

*S 15. $^{13}C\{^1H\}$ NMR spectra of compound **3a-Cyic** in $CDCl_3$ at 298K*

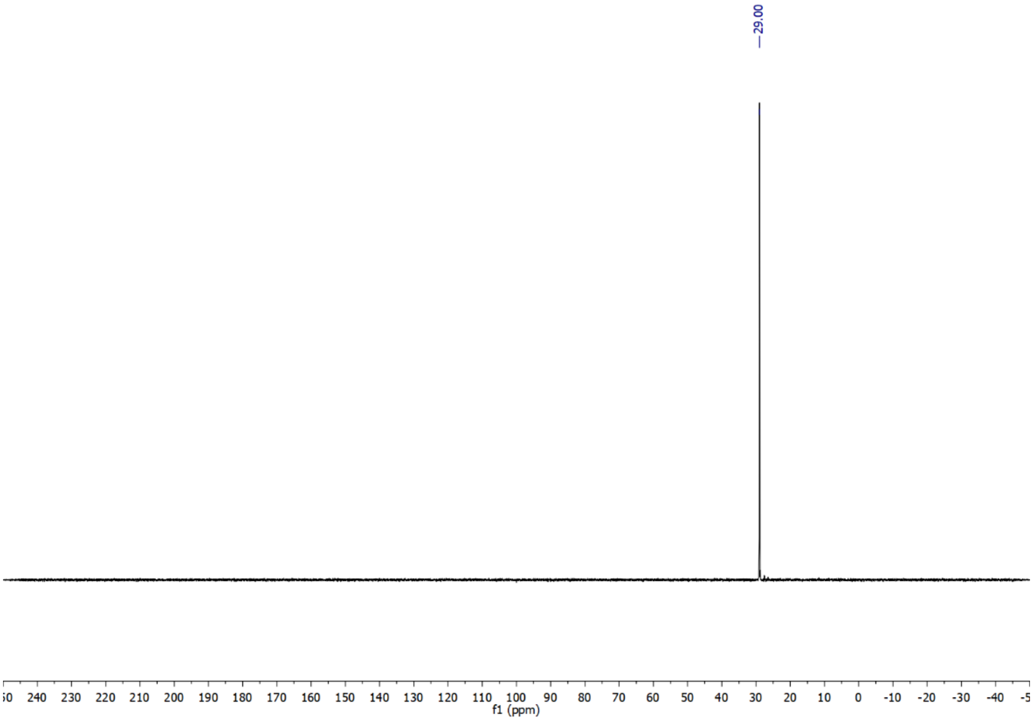

*S 16. $^{31}P\{^1H\}$ NMR spectra of compound **3a-Cyic** in $CDCl_3$ at 298K*

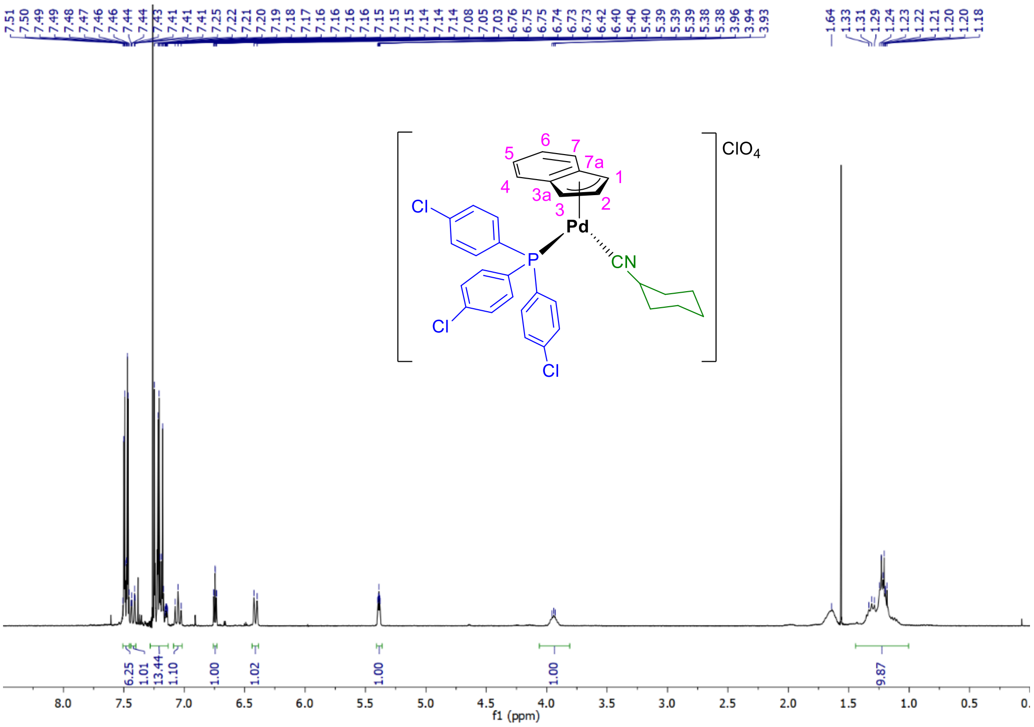

S 17. ^{1}H NMR spectra of compound ***3b-Cyic*** *in $CDCl_3$ at 298K*

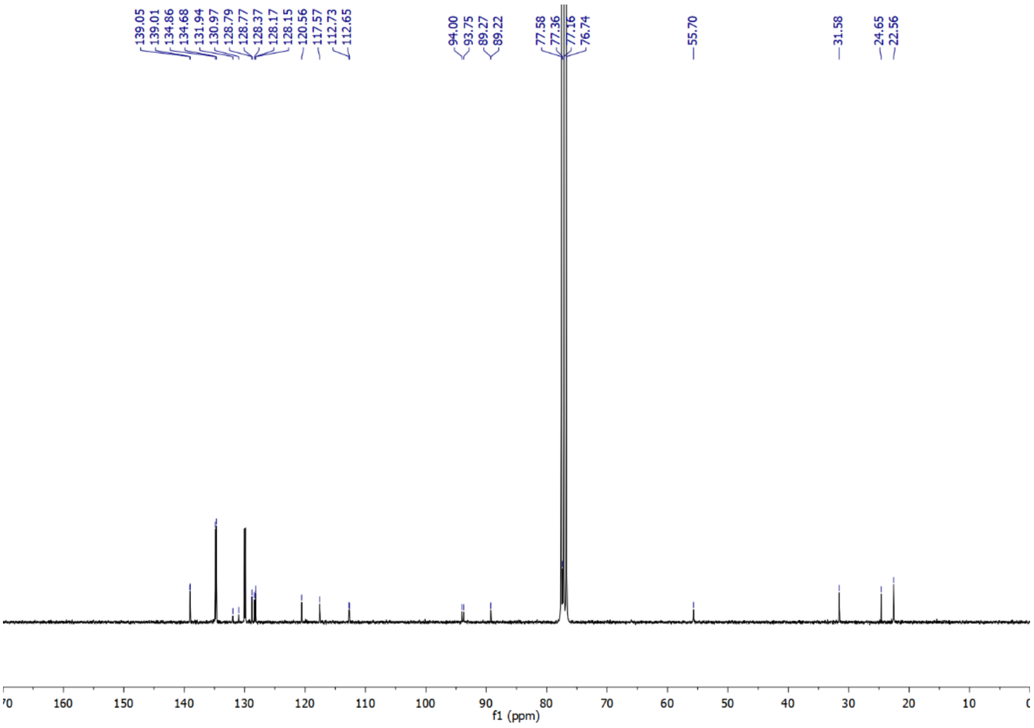

S 18. $^{13}C\{^{1}H\}$ NMR spectra of compound ***3b-Cyic*** *in $CDCl_3$ at 298K*

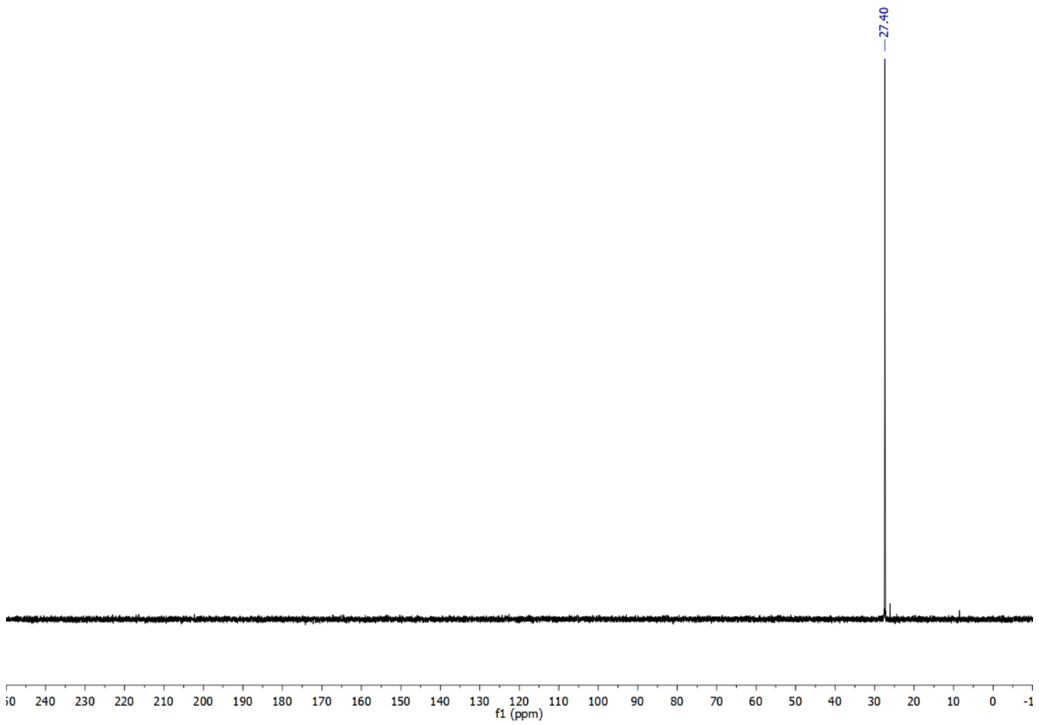

S 19. $^{31}P\{^{1}H\}$ *NMR spectra of compound* ***3b-Cyic*** *in* $CDCl_3$ *at 298K*

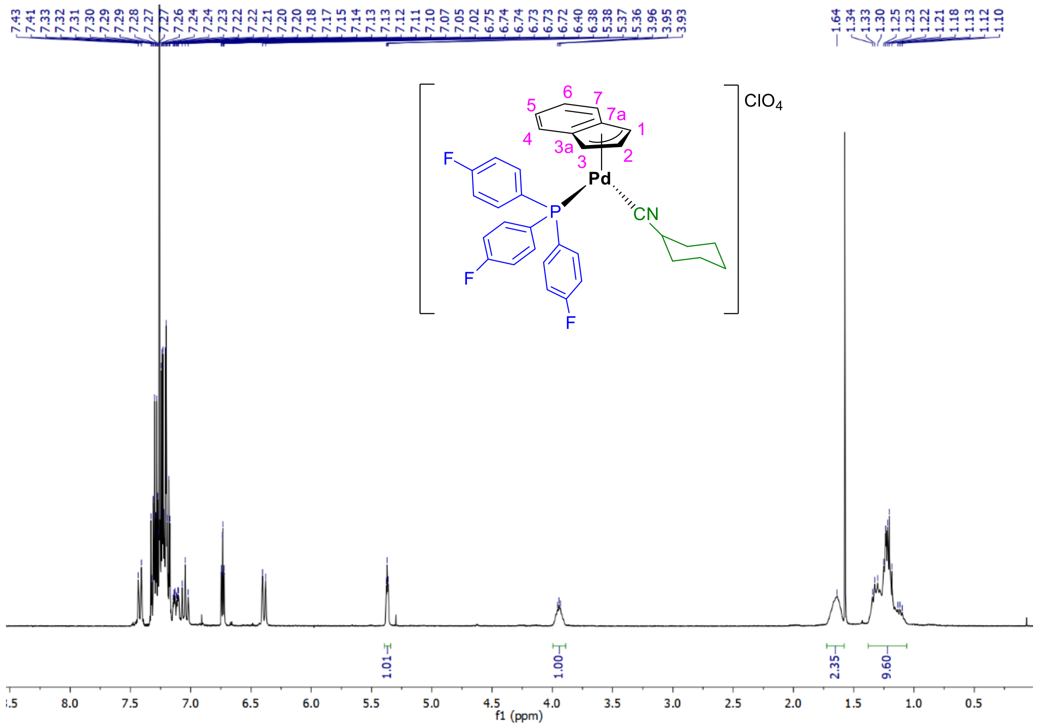

S 20. ^{1}H *NMR spectra of compound* ***3c-Cyic*** *in* $CDCl_3$ *at 298K*

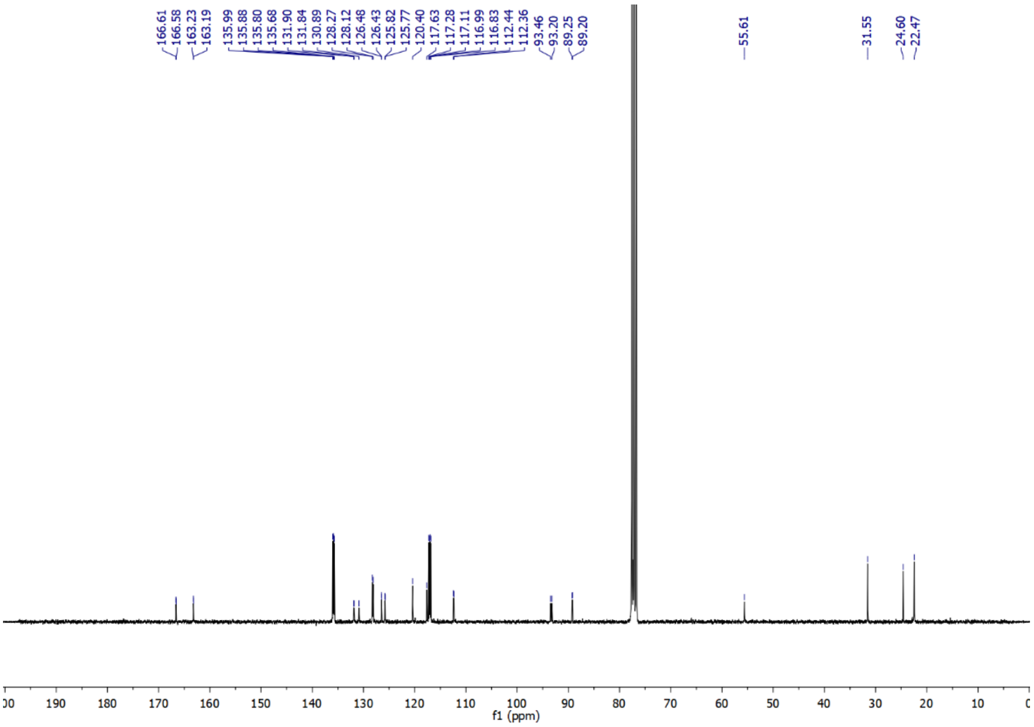

S 21. $^{13}C\{^{1}H\}$ NMR spectra of compound **3c-Cyic** in $CDCl_3$ at 298K

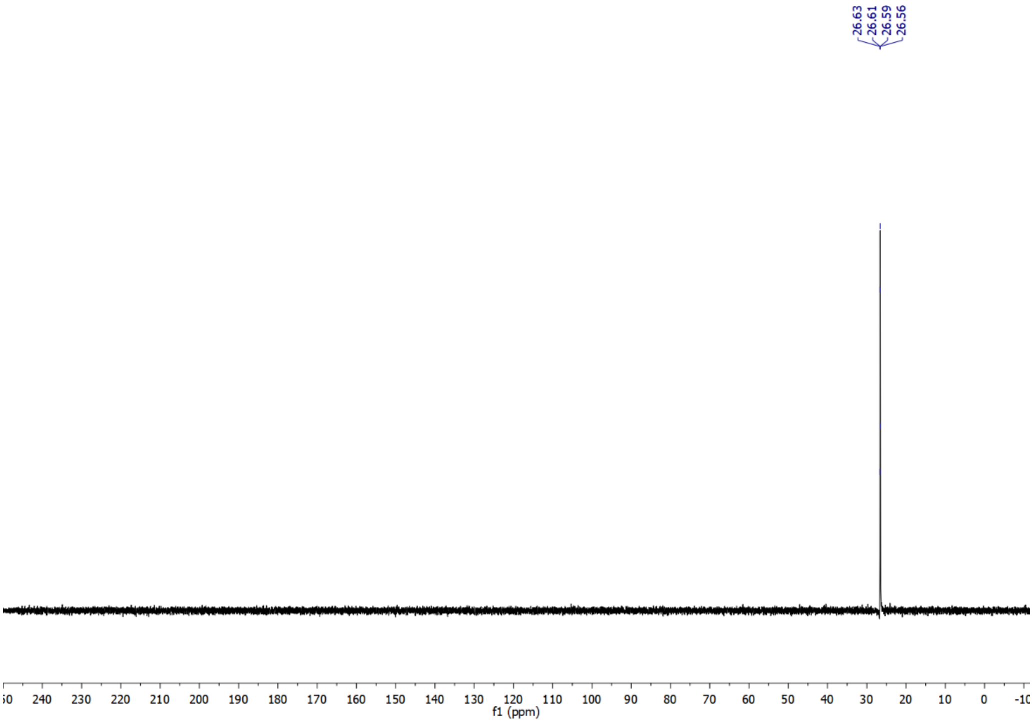

S 22. $^{31}P\{^{1}H\}$ NMR spectra of compound **3c-Cyic** in $CDCl_3$ at 298K

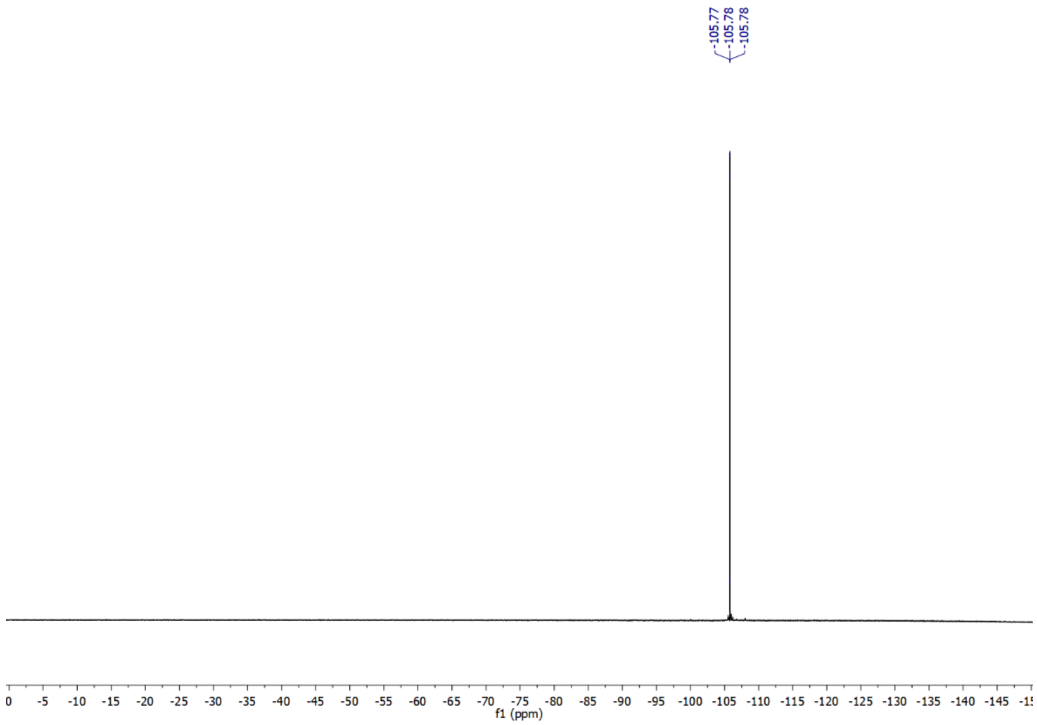

S 23. $^{19}F\{^{1}H\}$ NMR spectra of compound **3c-Cyic** in $CDCl_3$ at 298K

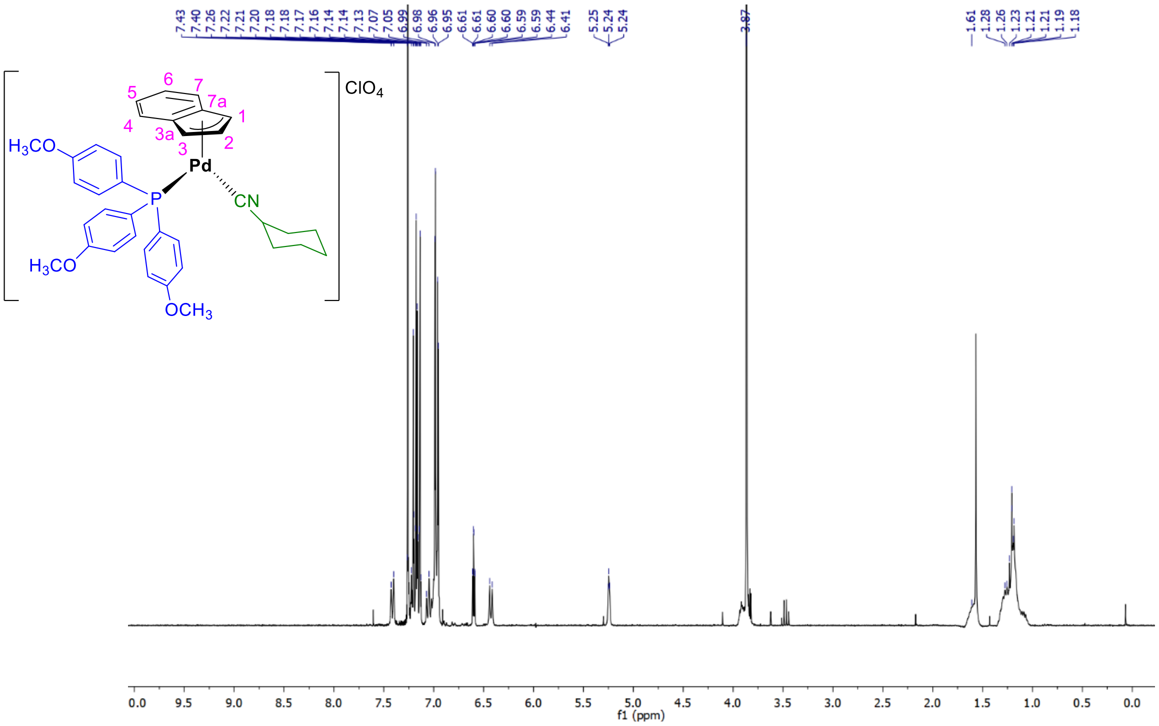

S 24. ^{1}H NMR spectra of compound **3d-Cyic** in $CDCl_3$ at 298K

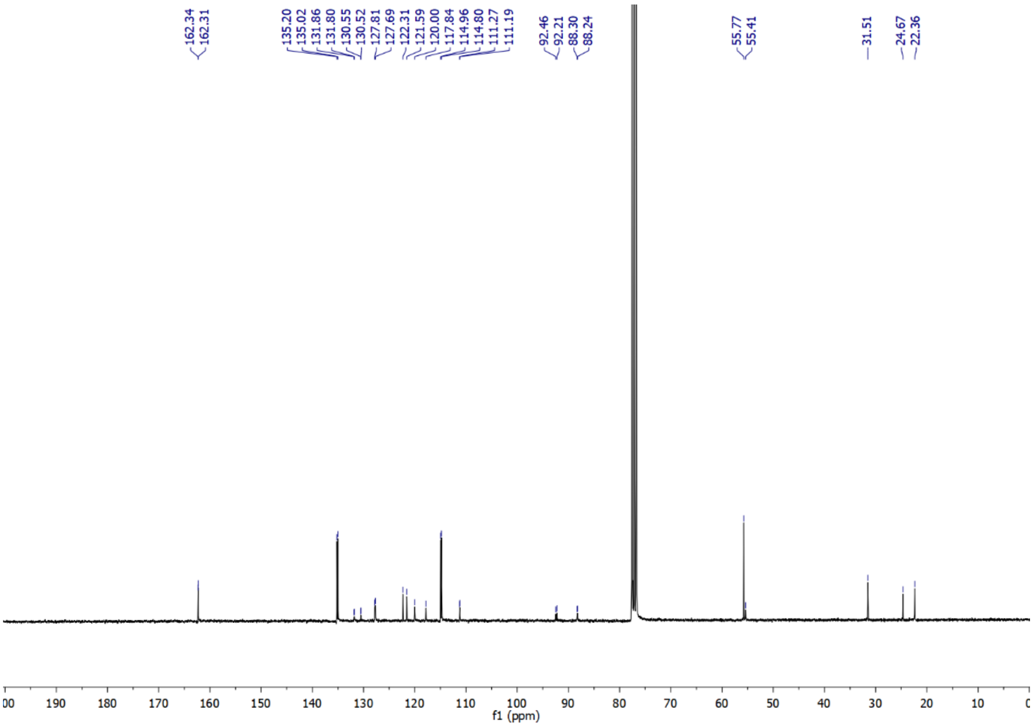

*S 25. $^{13}C\{^{1}H\}$ NMR spectra of compound **3d-Cyic** in $CDCl_3$ at 298K*

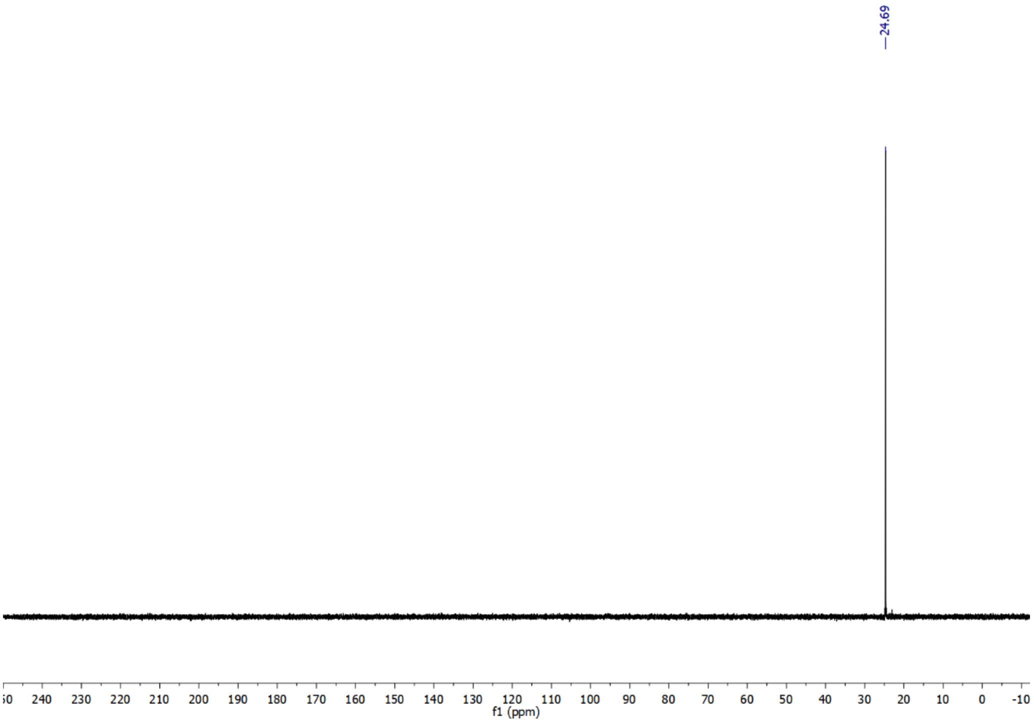

*S 26. $^{31}P\{^{1}H\}$ NMR spectra of compound **3d-Cyic** in $CDCl_3$ at 298K*

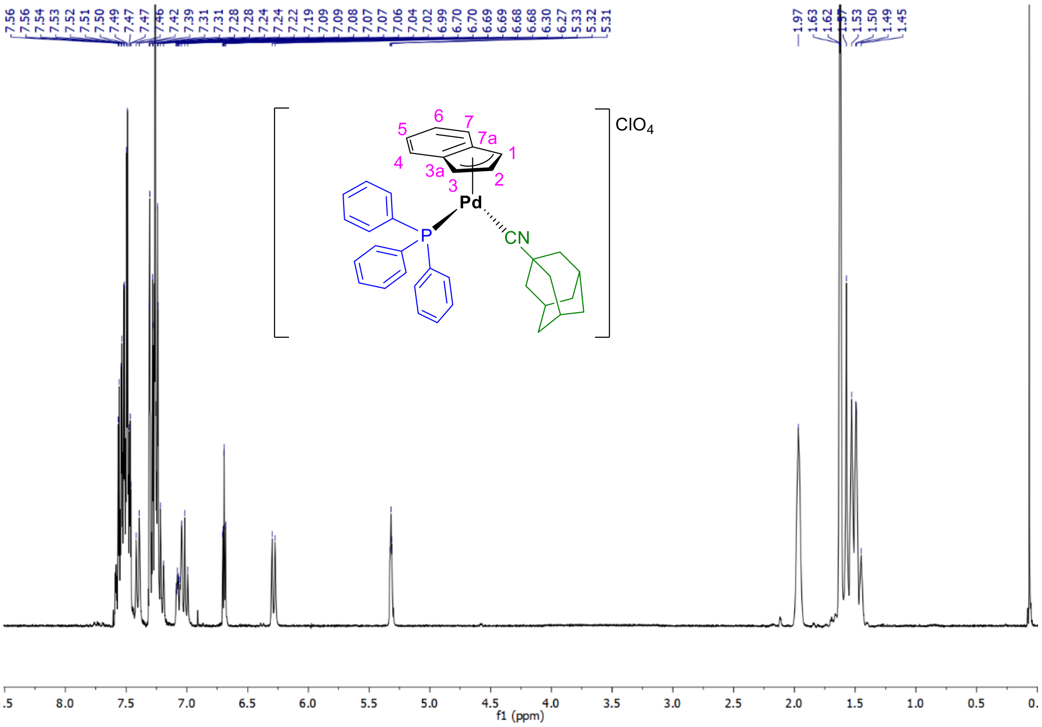

*S 27. ¹H NMR spectra of compound **3a-Adic** in CDCl₃ at 298K*

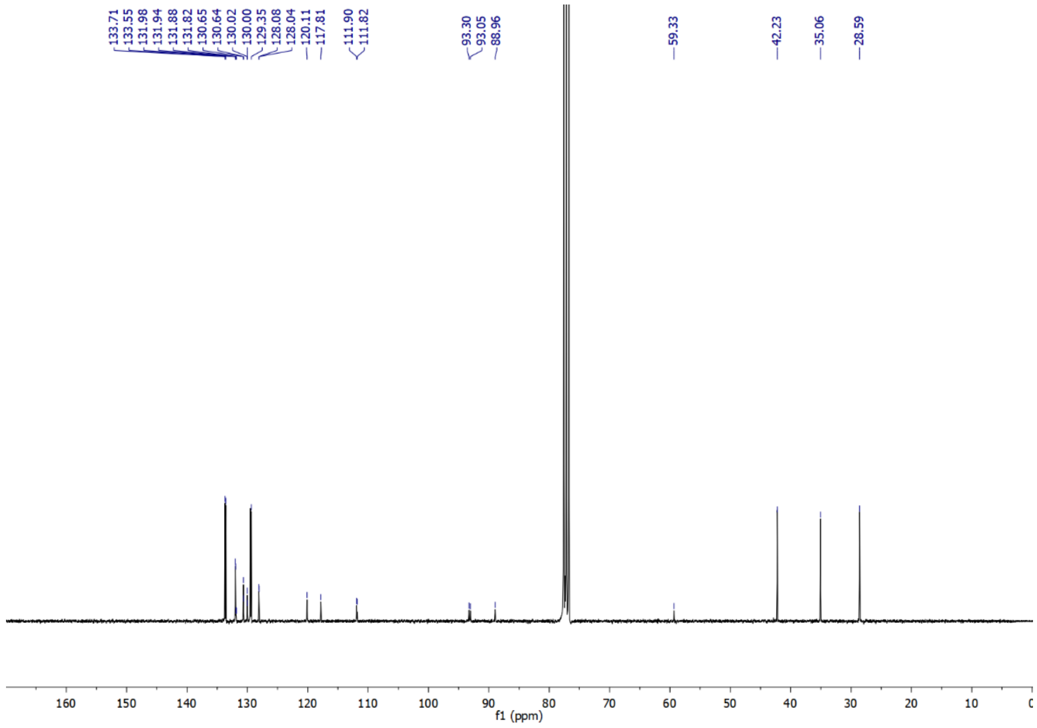

*S 28. ¹³C{¹H} NMR spectra of compound **3a-Adic** in CDCl₃ at 298K*

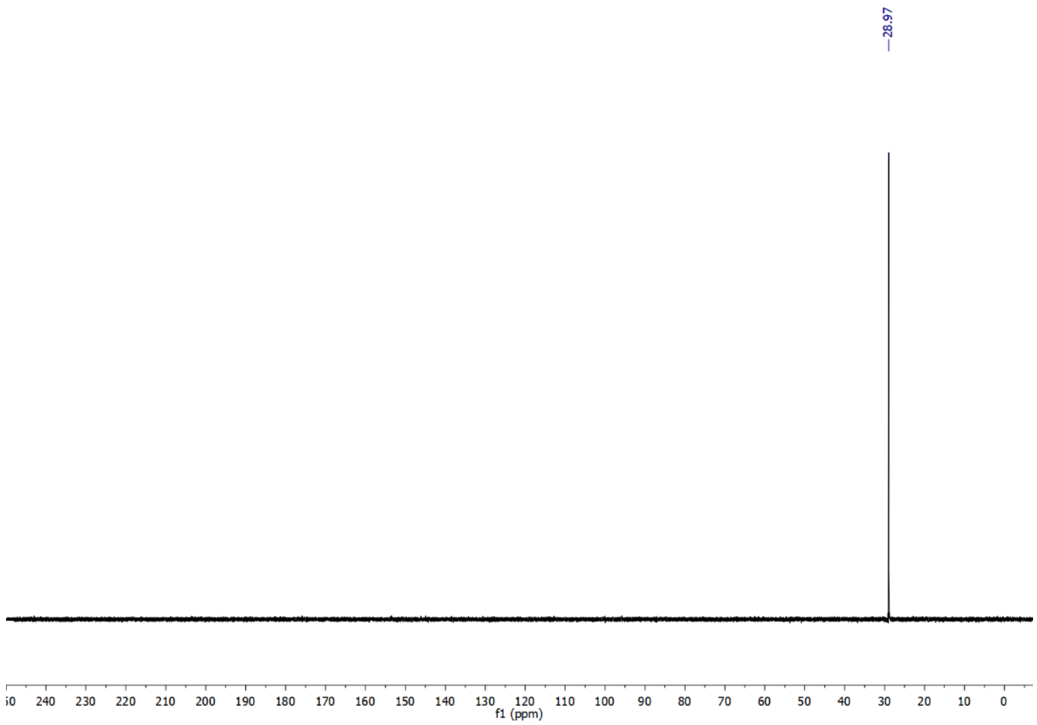

S 29.^{31}P{^{1}H} NMR spectra of compound **3a-Adic** in CDCl$_3$ at 298K

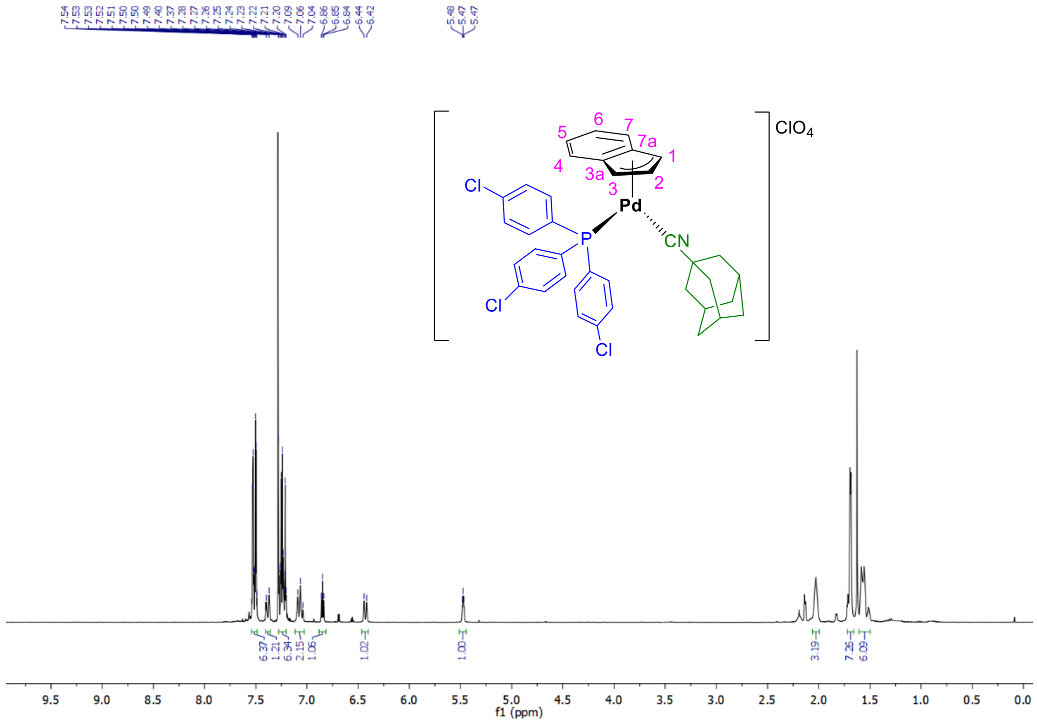

S 30. ^{1}H NMR spectra of compound **3b-Adic** in CDCl$_3$ at 298K

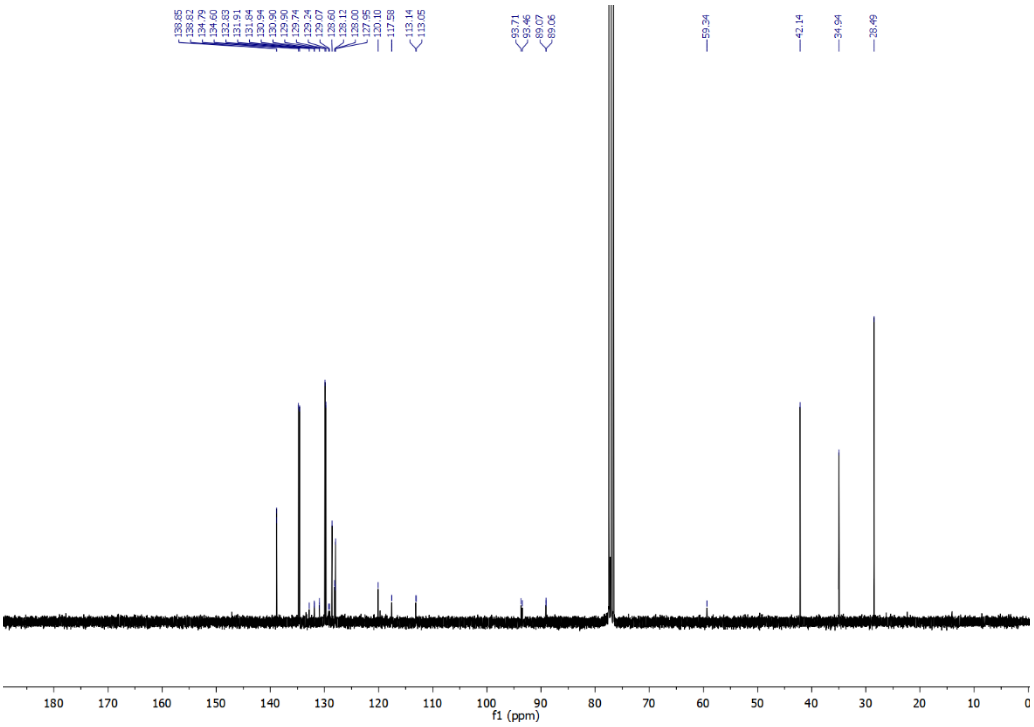

*S 31. $^{13}C\{^1H\}$ NMR spectra of compound **3b-Adic** in $CDCl_3$ at 298K*

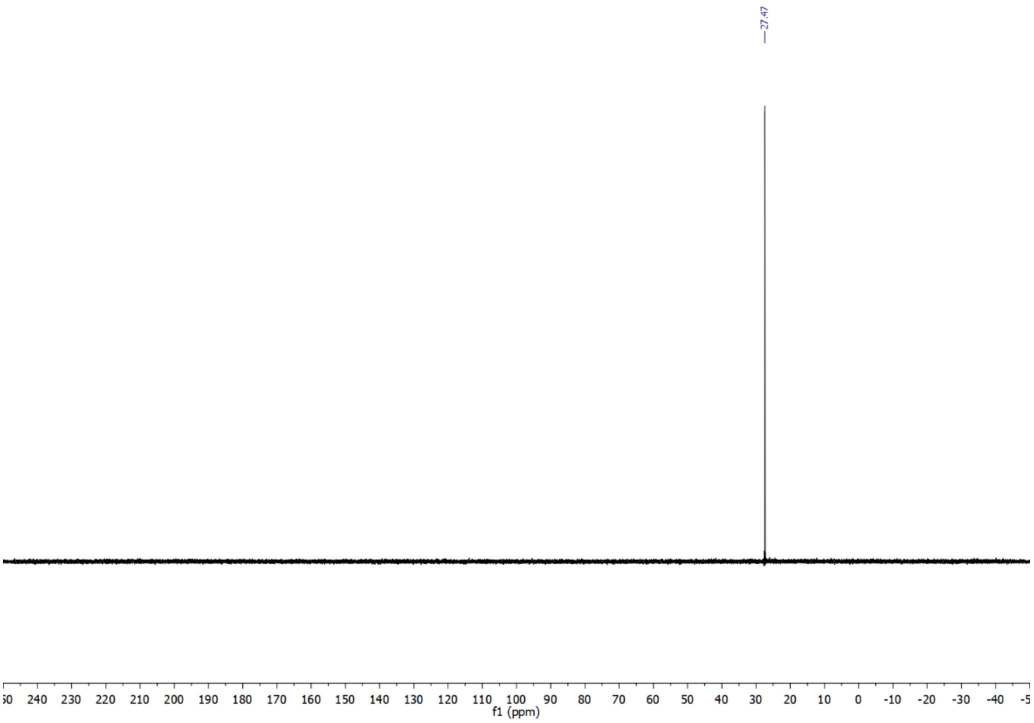

*S 32. $^{31}P\{^1H\}$ NMR spectra of compound **3b-Adic** in $CDCl_3$ at 298K*

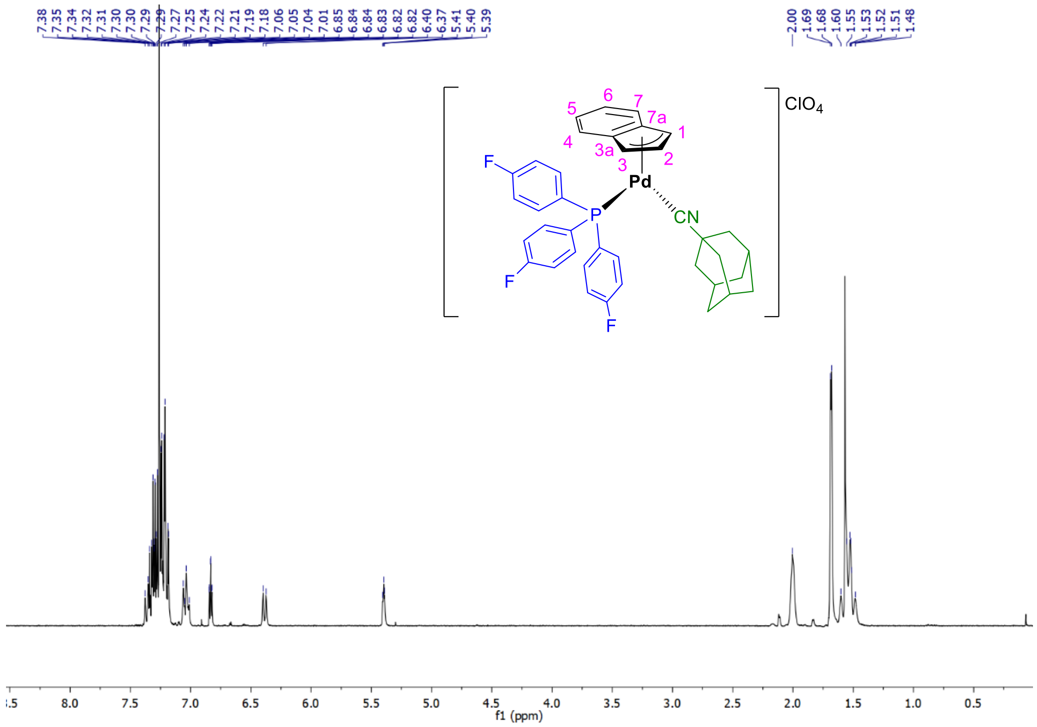

S 33. ¹H NMR spectra of compound ***3c-Adic*** *in CDCl₃ at 298K*

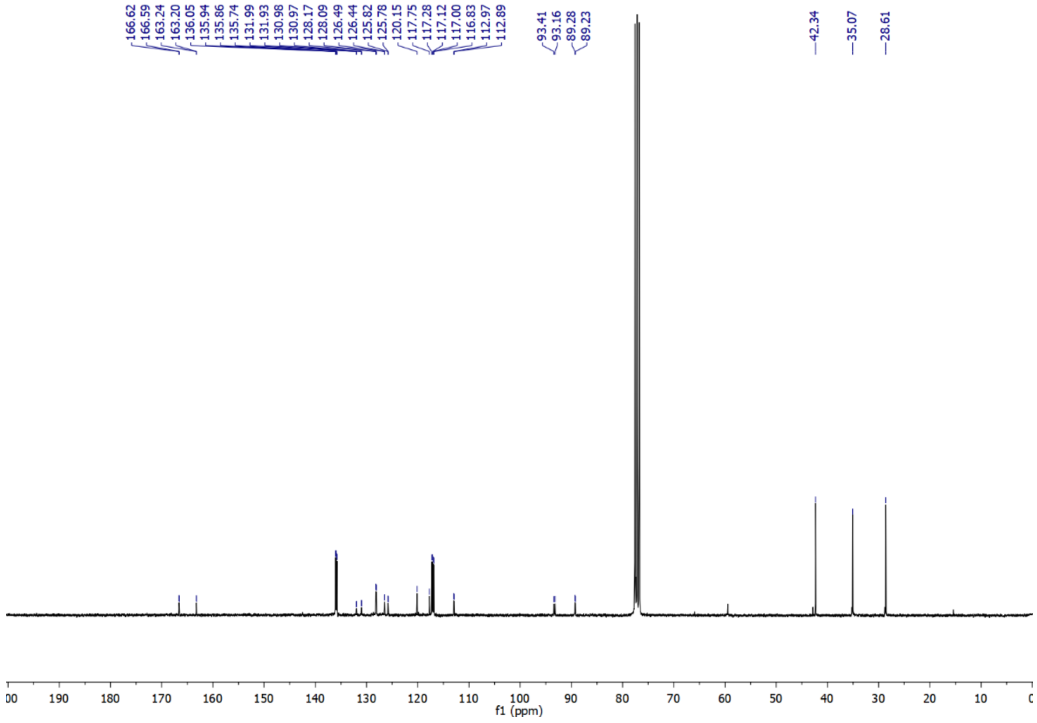

S 34.¹³C{¹H} NMR spectra of compound ***3c-Adic*** *in CDCl₃ at 298K*

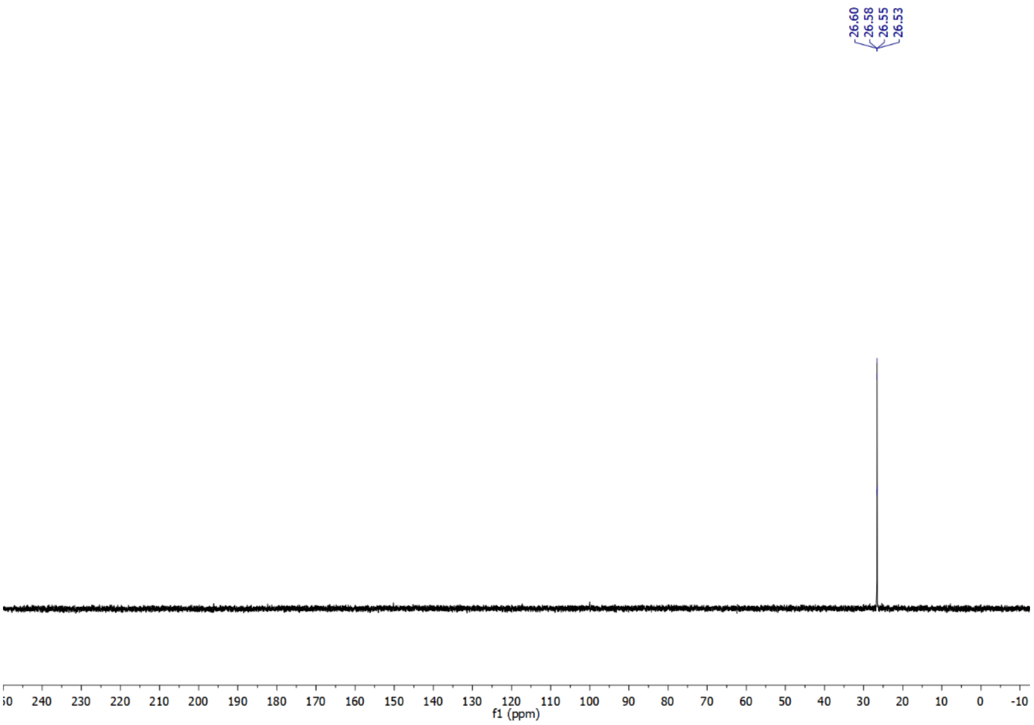

*S 35. $^{31}P\{^{1}H\}$ NMR spectra of compound **3c-Adic** in $CDCl_3$ at 298K*

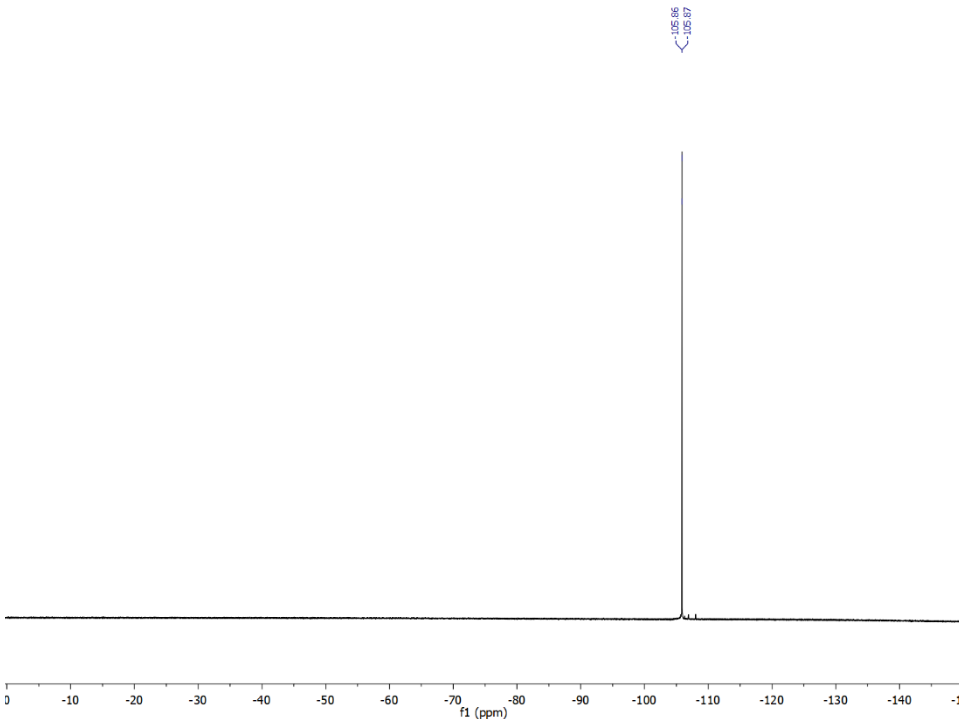

*S 36. $^{19}F\{^{1}H\}$ NMR spectra of compound **3c-Adic** in $CDCl_3$ at 298K*

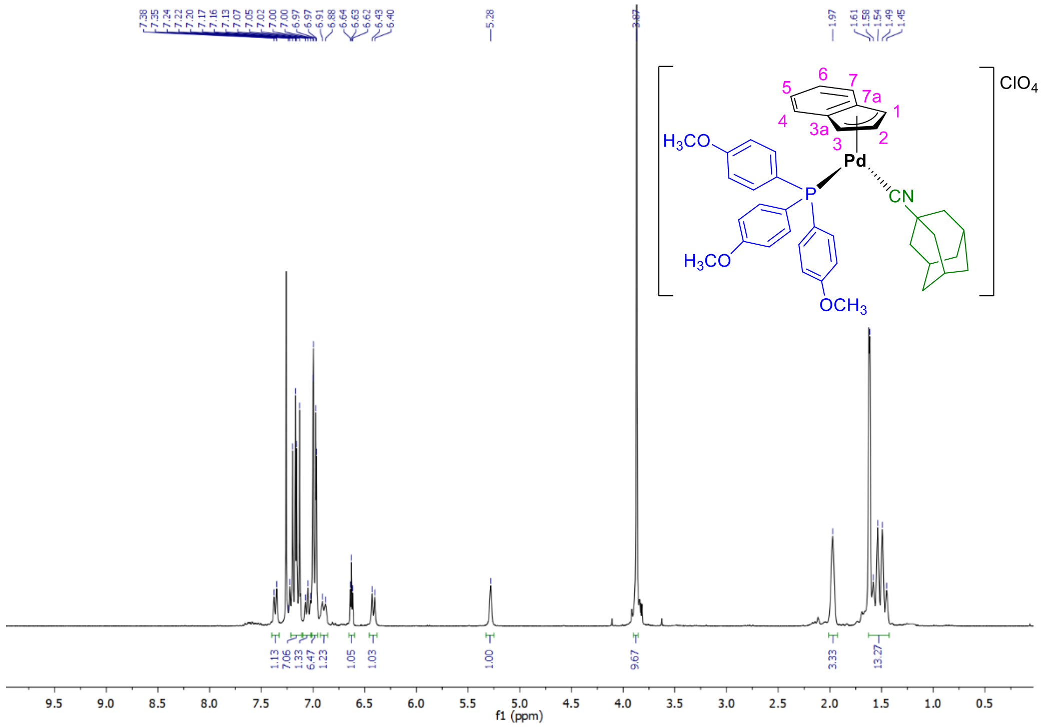

S 37. ^{1}H NMR spectra of compound **3d-Adic** in $CDCl_3$ at 298K

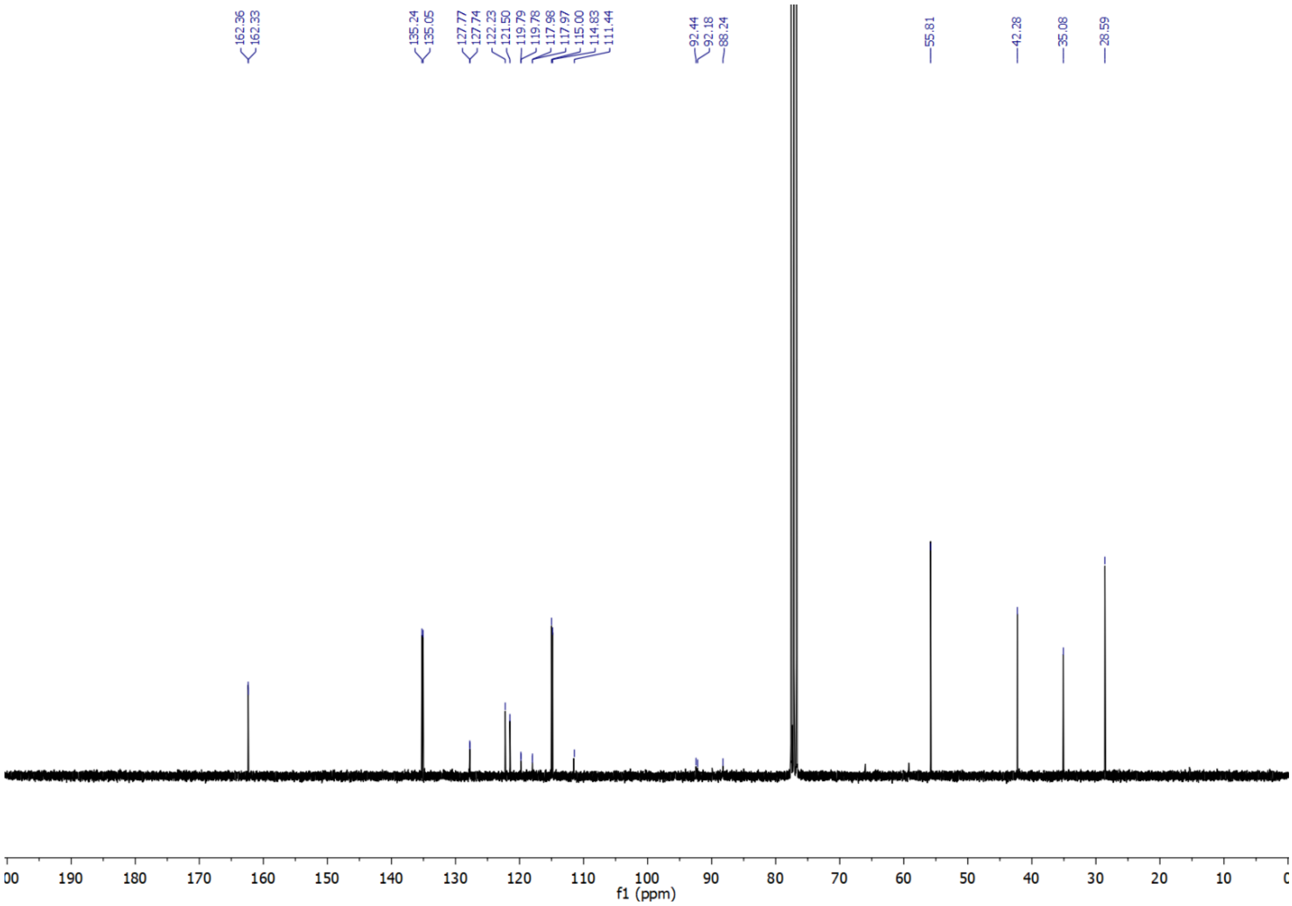

S 38. $^{13}C\{^{1}H\}$ NMR spectra of compound **3d-Adic** in $CDCl_3$ at 298K

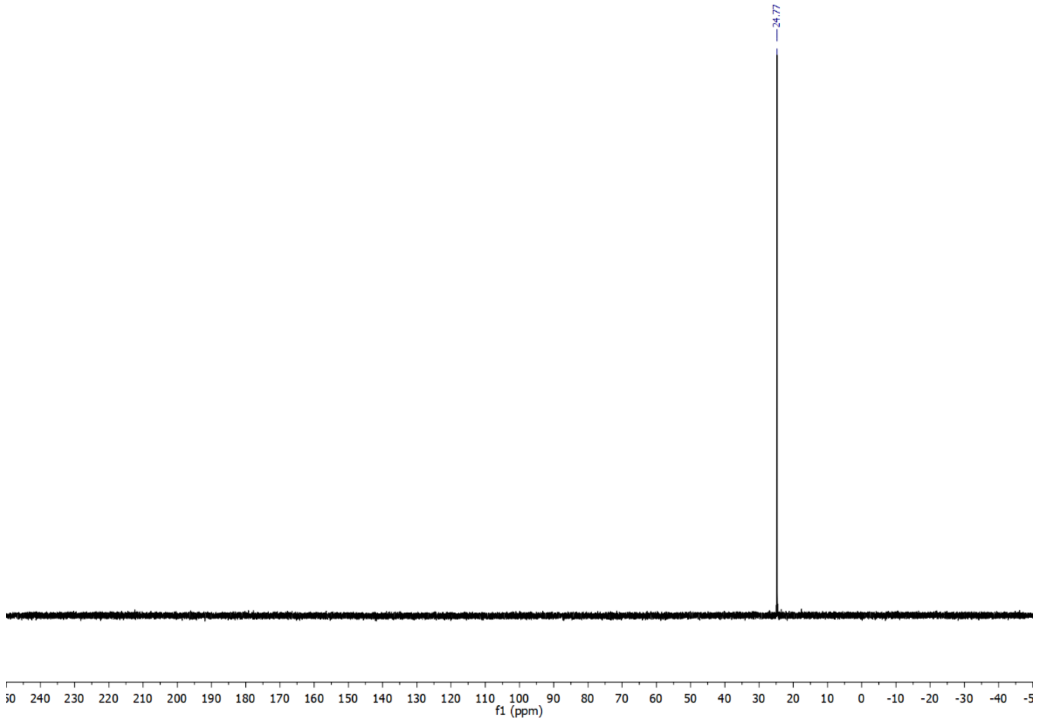

*S 39. $^{31}P\{^{1}H\}$ NMR spectra of compound **3d-Adic** in $CDCl_3$ at 298K*

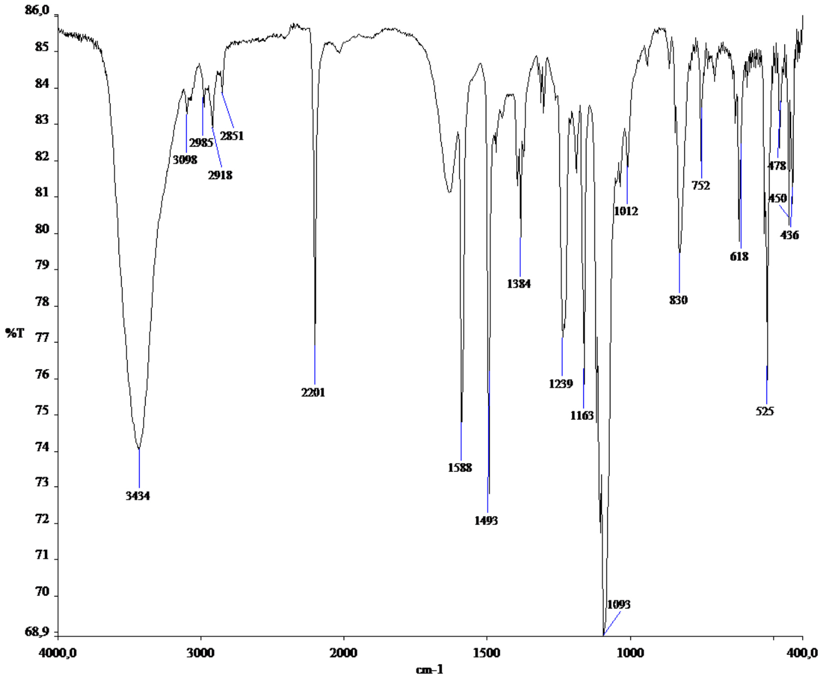

*S 40. IR spectra of compound **3c-Tic***

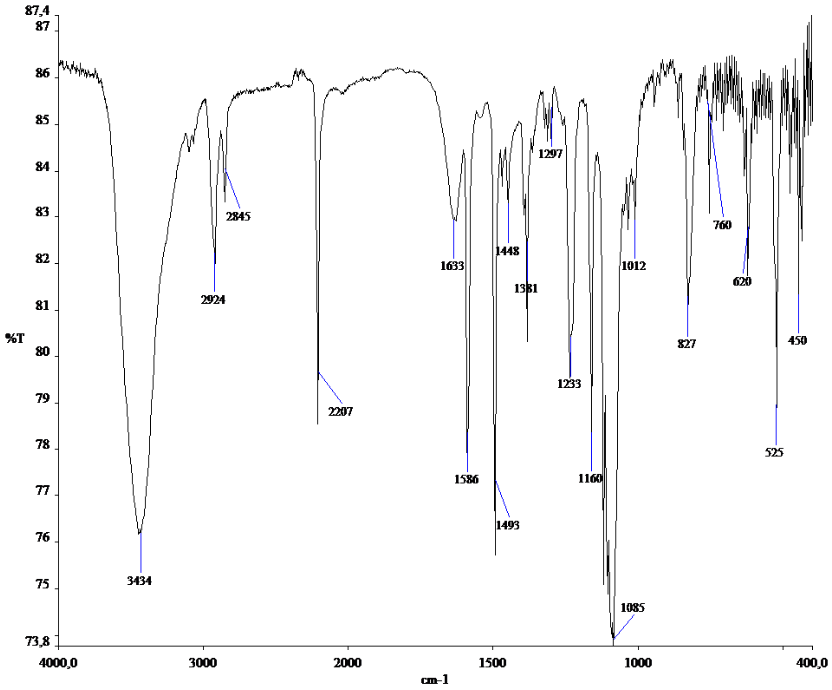

*S 41. IR spectra of compound **3c-Cyic***

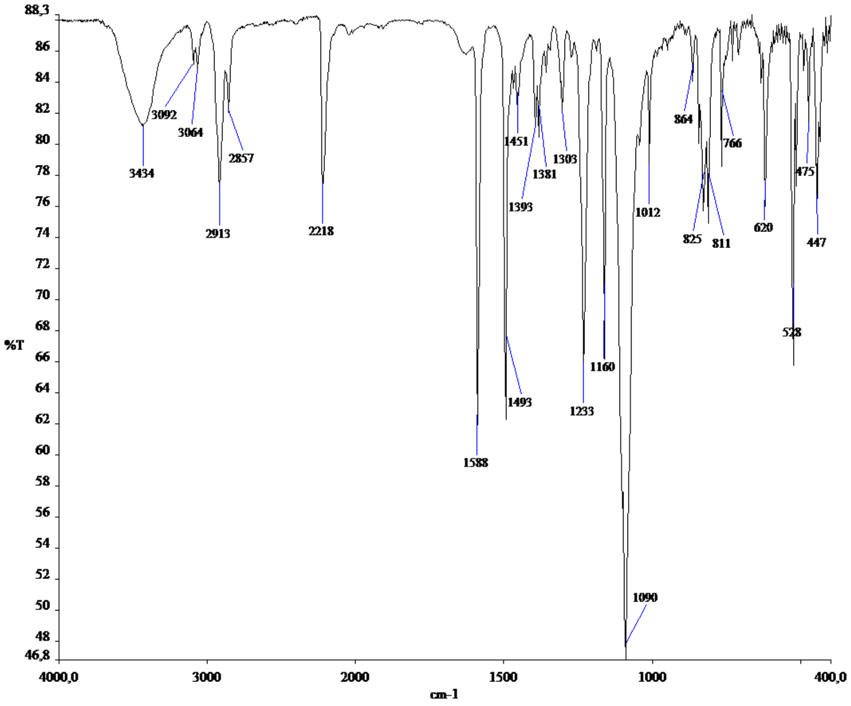

*S 42. IR spectra of compound **3c-Adic***

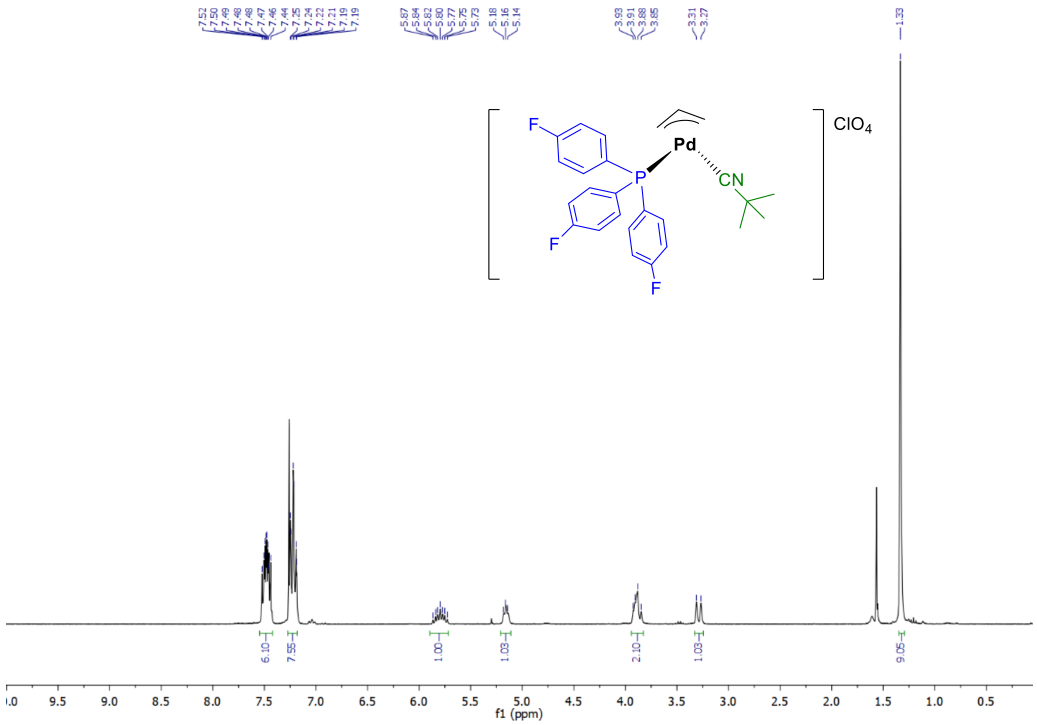

S 43 .¹H NMR spectra of compound ***6c-Tic*** *in* $CDCl_3$ *at 298K*

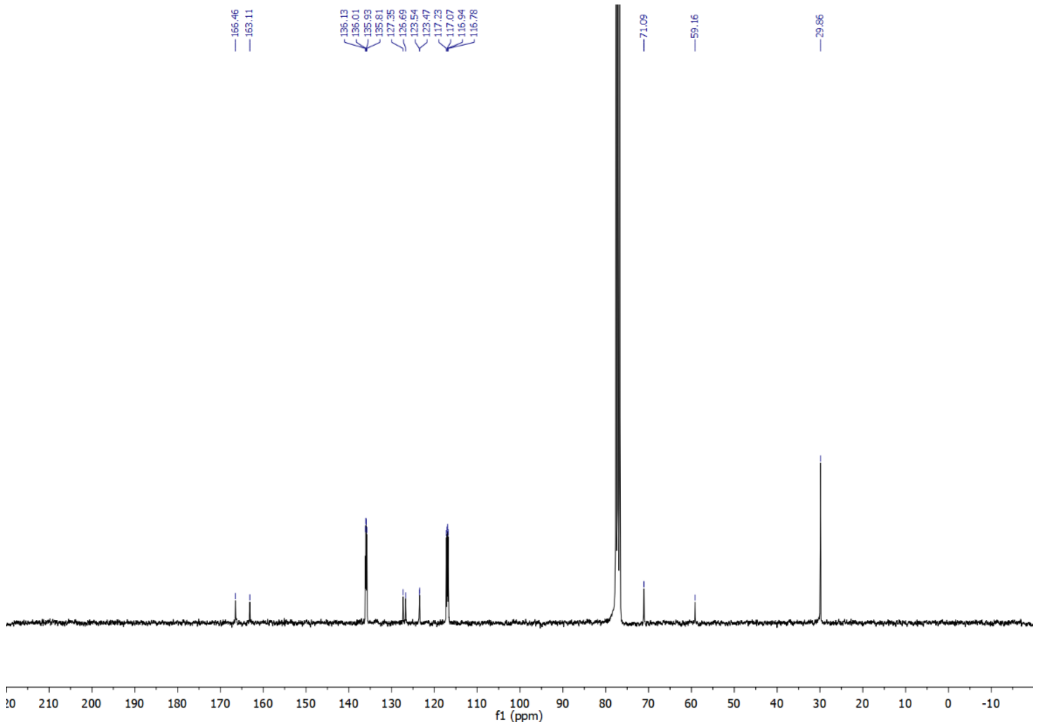

S 44. $^{13}C\{^1H\}$ *NMR spectra of compound* ***6c-Tic*** *in* $CDCl_3$ *at 298K*

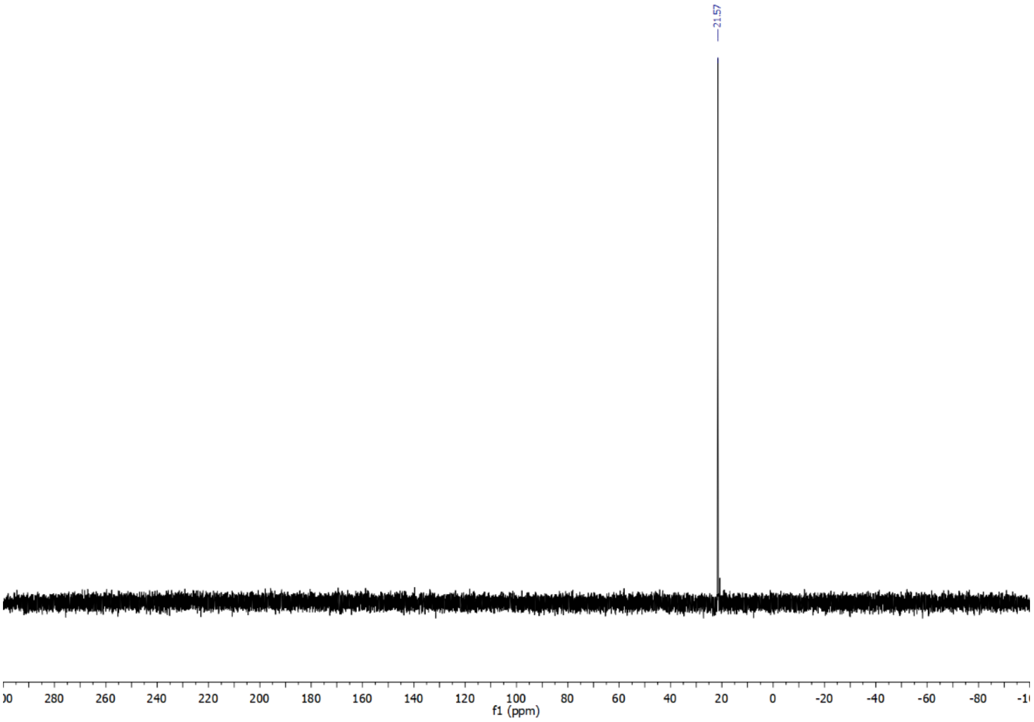

*S 45. $^{31}P\{^{1}H\}$ NMR spectra of compound **6c-Tic** in $CDCl_3$ at 298K*

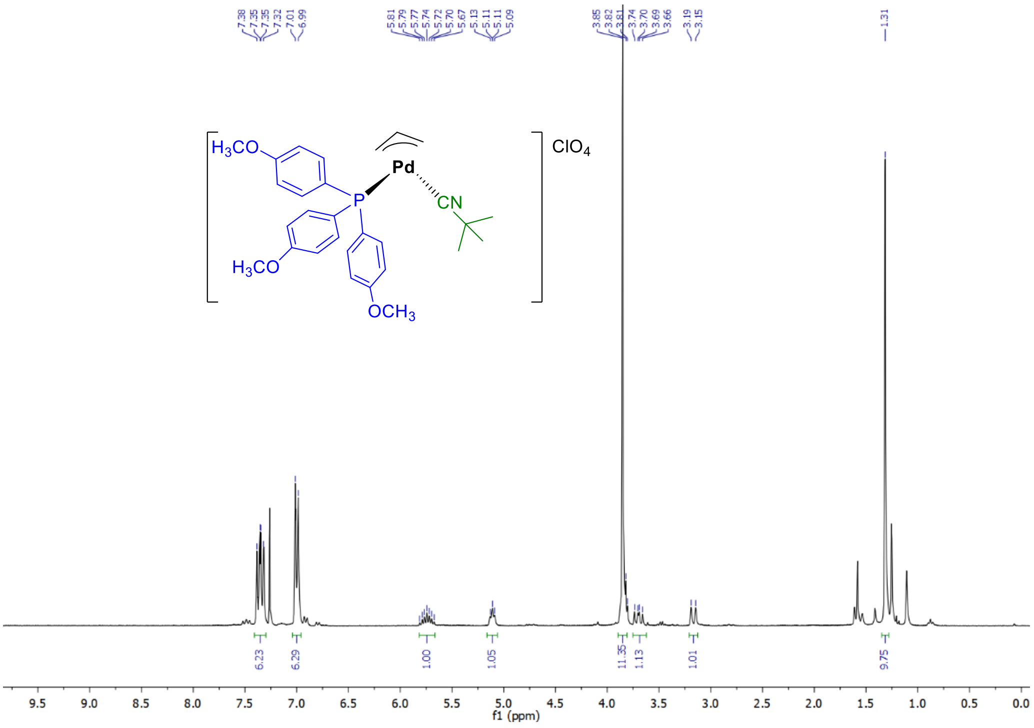

*S 46. ^{1}H NMR spectra of compound **6d-Tic** in $CDCl_3$ at 298K*

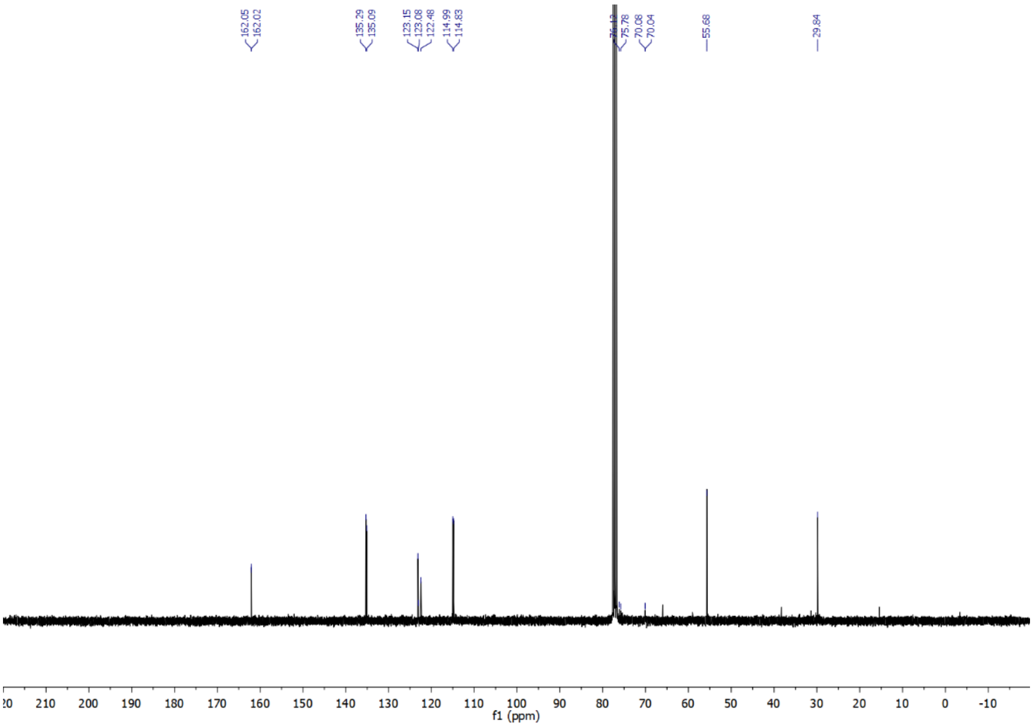

*S 47. $^{13}C\{^{1}H\}$ NMR spectra of compound **6d-Tic** in $CDCl_3$ at 298K*

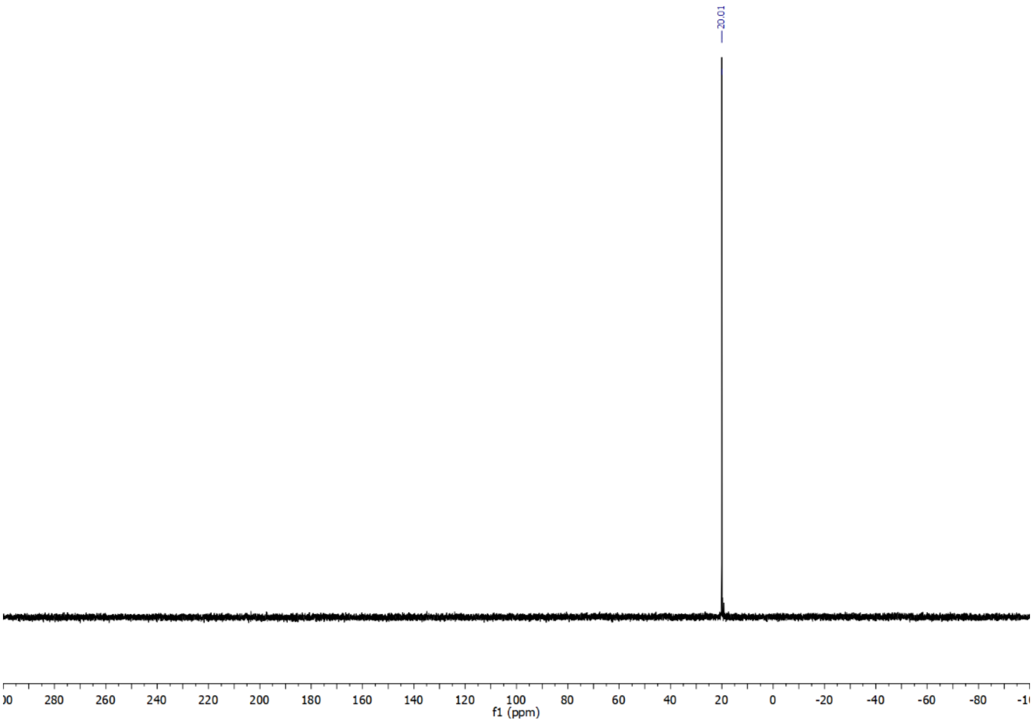

*S 48. $^{31}P\{^{1}H\}$ NMR spectra of compound **6d-Tic** in $CDCl_3$ at 298K*

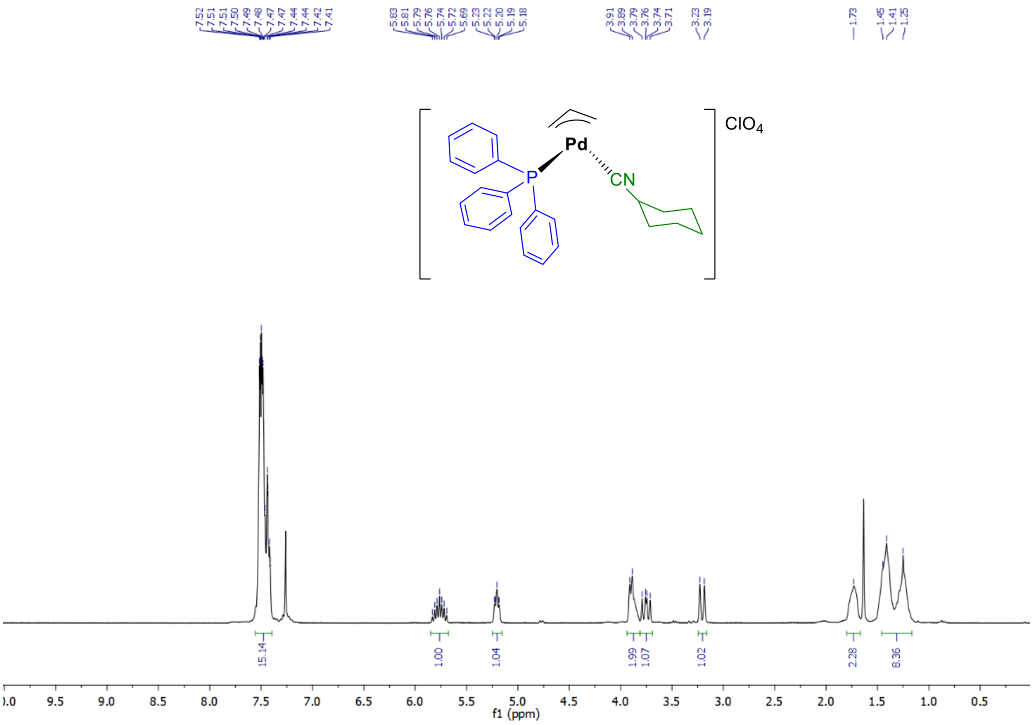

S 49. ^{1}H NMR spectra of compound **6a-Cyic** in $CDCl_3$ at 298K

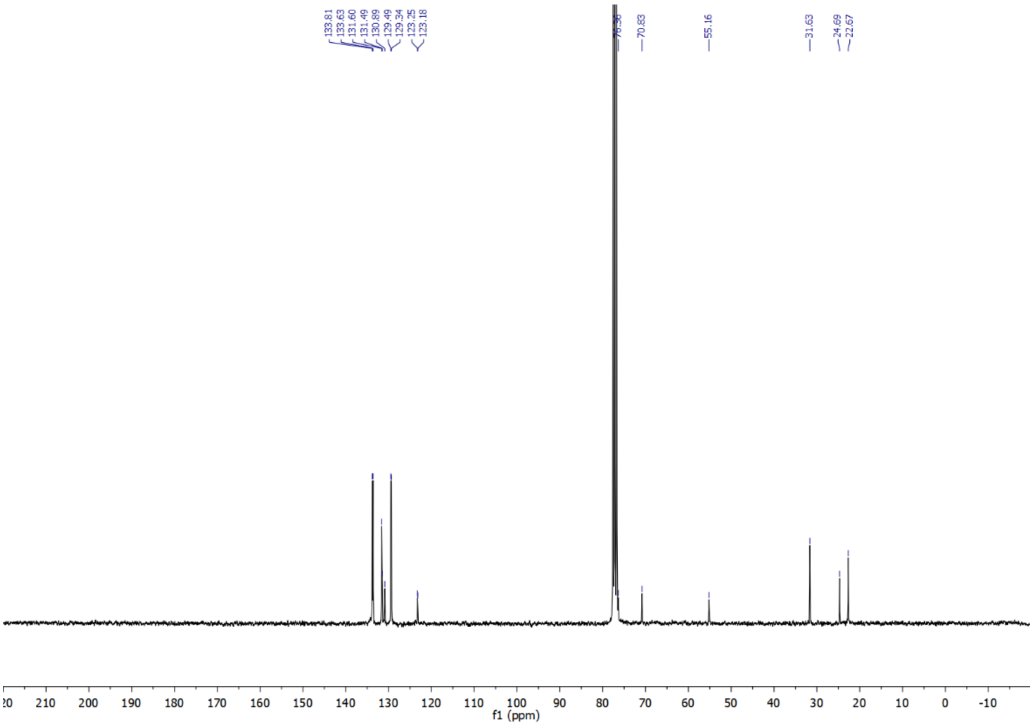

S 50. $^{13}C\{^{1}H\}$ NMR spectra of compound **6a-Cyic** in $CDCl_3$ at 298K

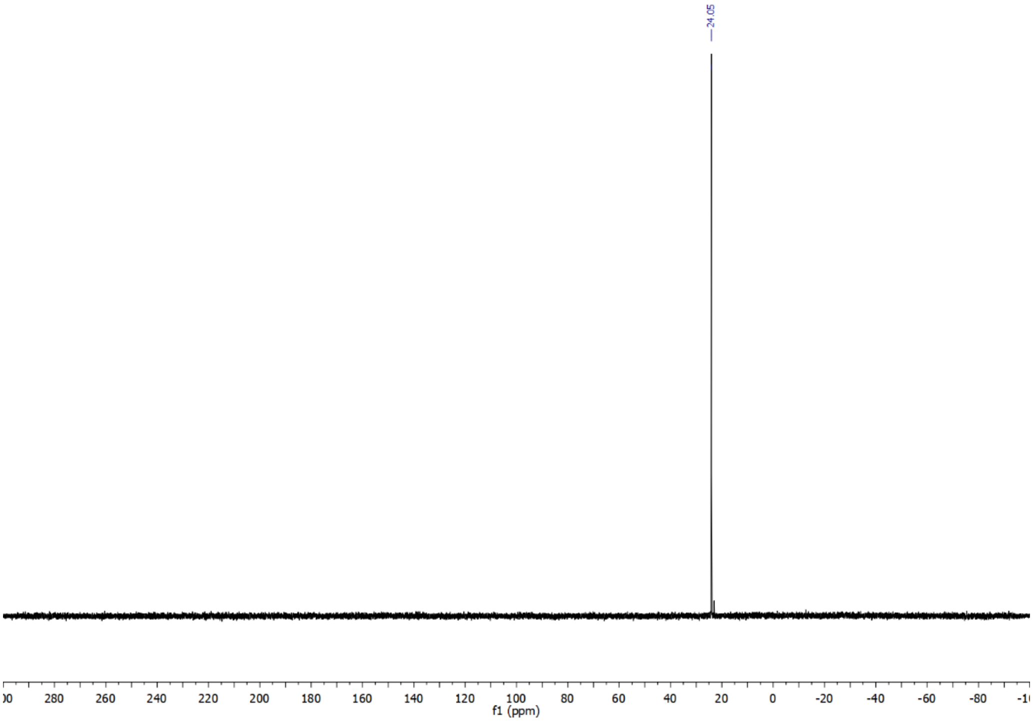

*S 51. $^{31}P\{^{1}H\}$ NMR spectra of compound **6a-Cyic** in $CDCl_3$ at 298K*

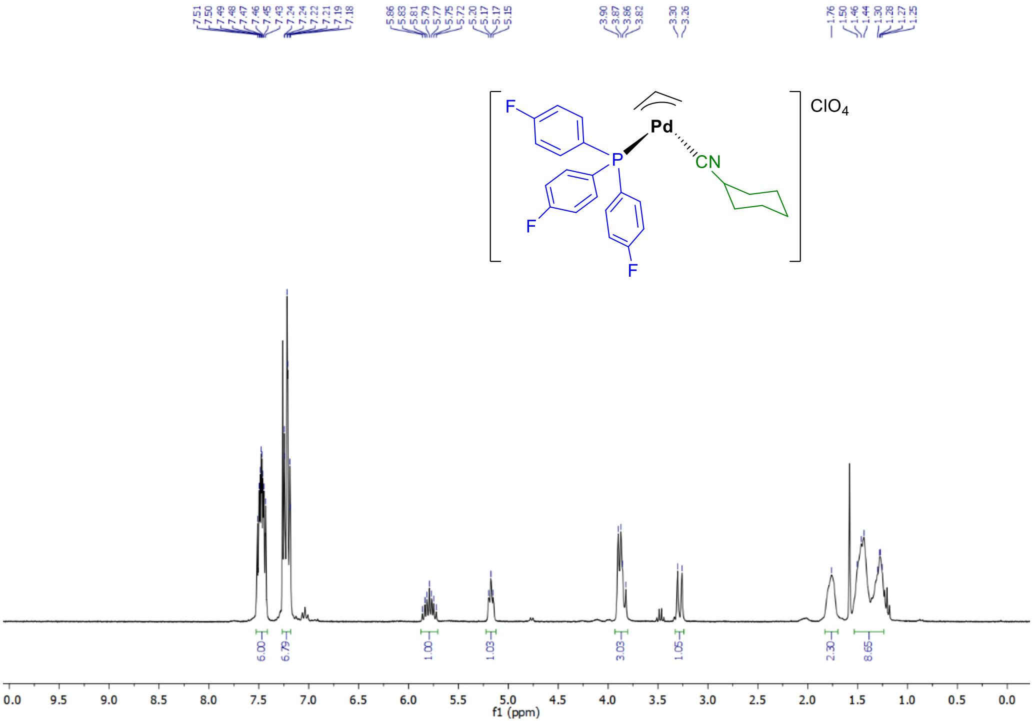

*S 52. ^{1}H NMR spectra of compound **6c-Cyic** in $CDCl_3$ at 298K*

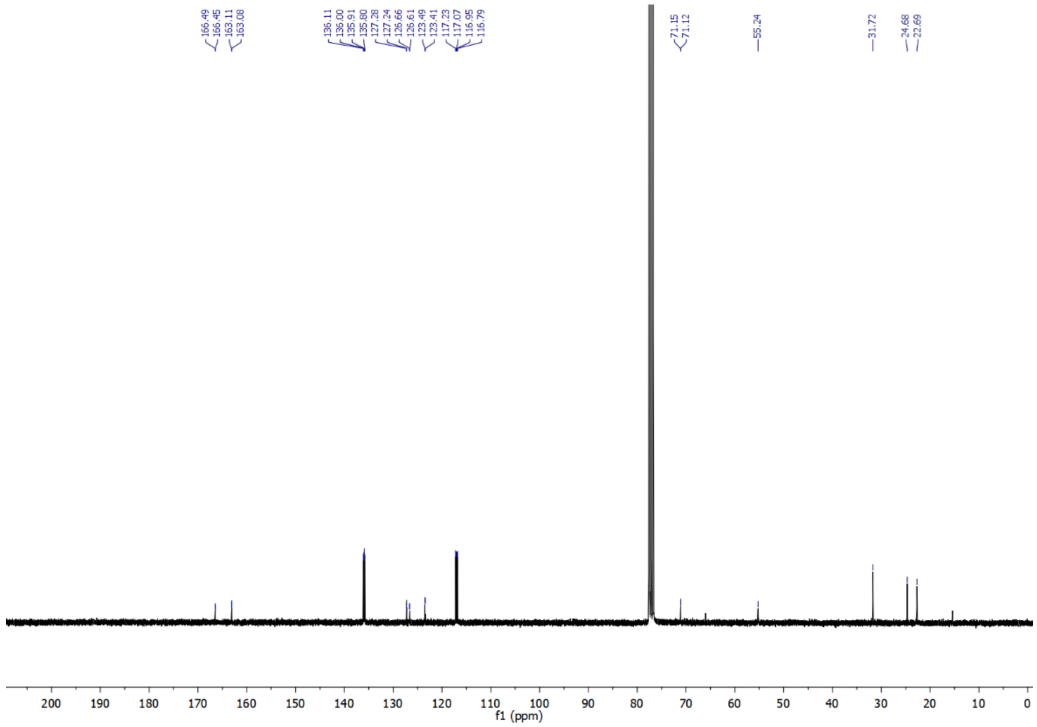

S 53. $^{13}C\{^{1}H\}$ NMR spectra of compound **6c-Cyic** in $CDCl_3$ at 298K

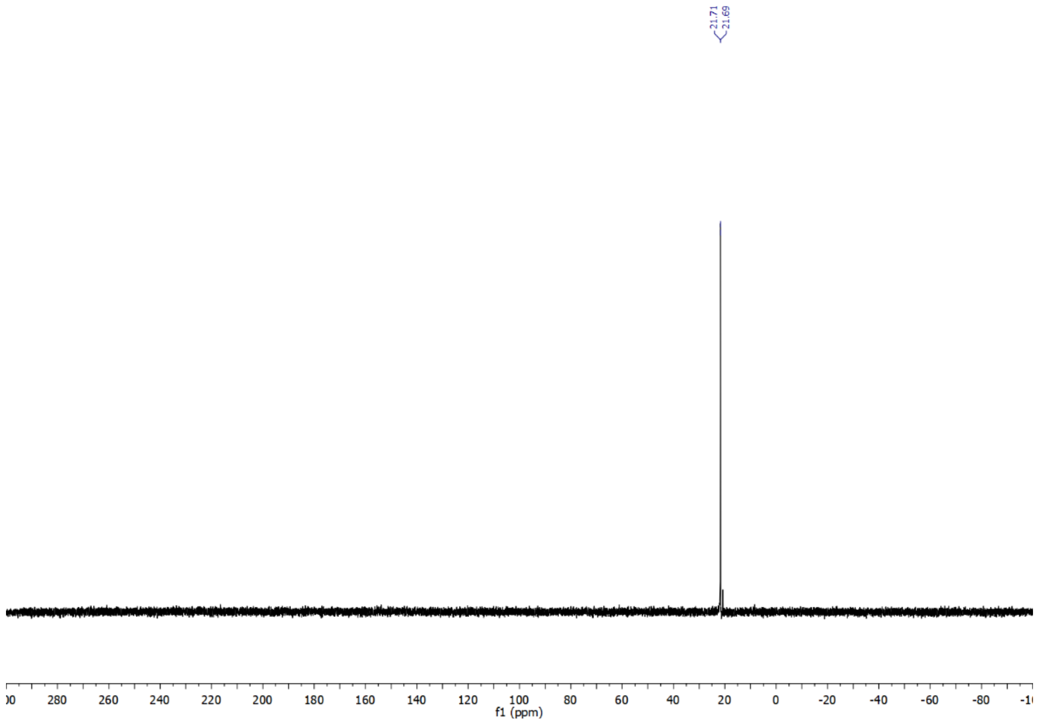

S 54. $^{31}P\{^{1}H\}$ NMR spectra of compound **6c-Cyic** in $CDCl_3$ at 298K

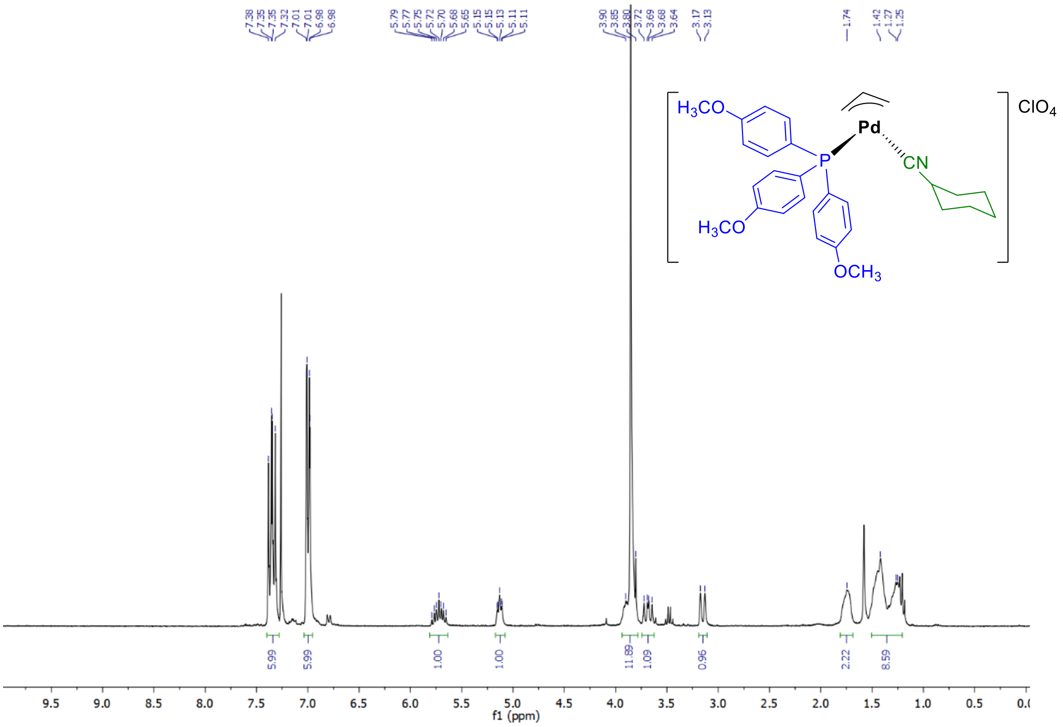

S 55. ^{1}H NMR spectra of compound **6d-Cyic** in $CDCl_3$ at 298K

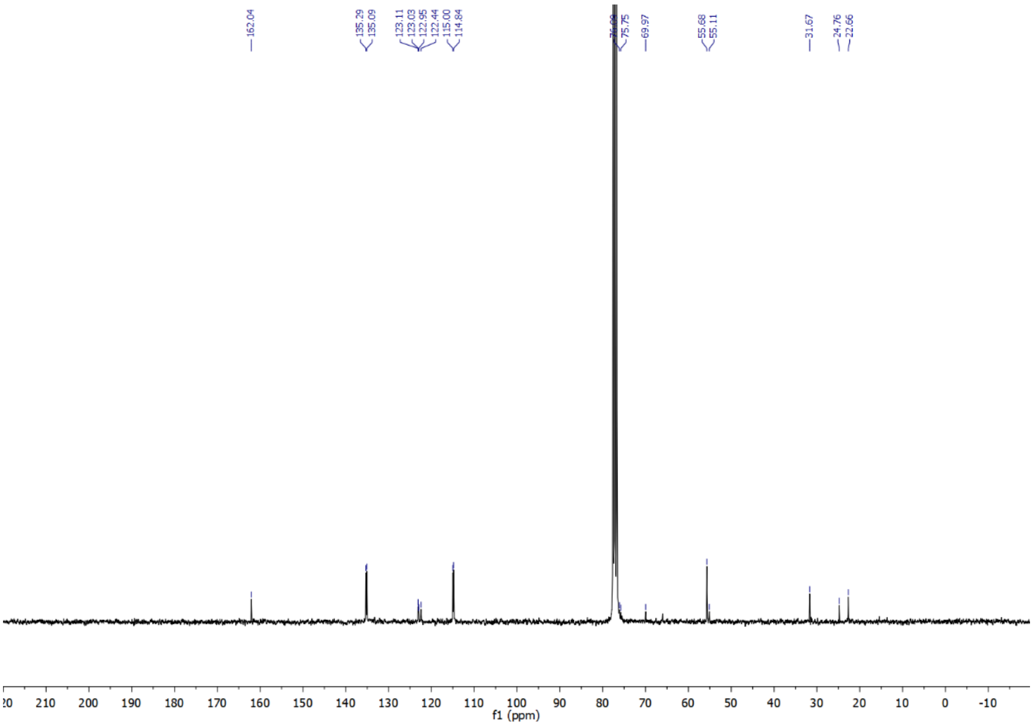

S 56. $^{13}C\{^{1}H\}$ NMR spectra of compound **6d-Cyic** in $CDCl_3$ at 298K

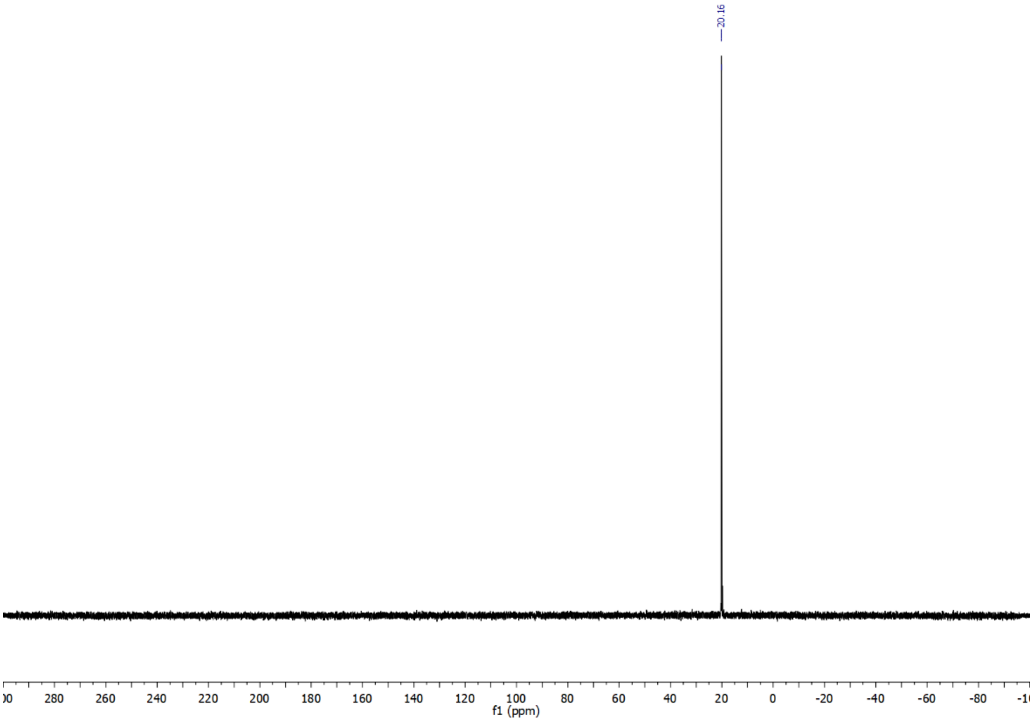

S 57. $^{31}P\{^{1}H\}$ *NMR spectra of compound* ***6d-Cyic*** *in* $CDCl_3$ *at 298K*

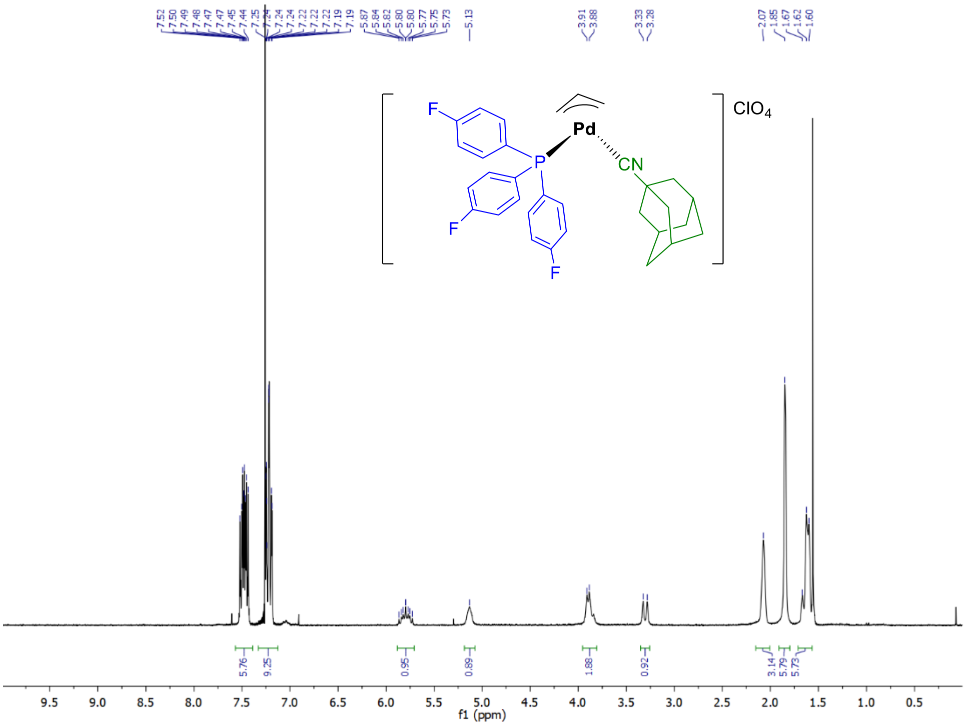

S 58. ^{1}H *NMR spectra of compound* ***6c-Adic*** *in* $CDCl_3$ *at 298K*

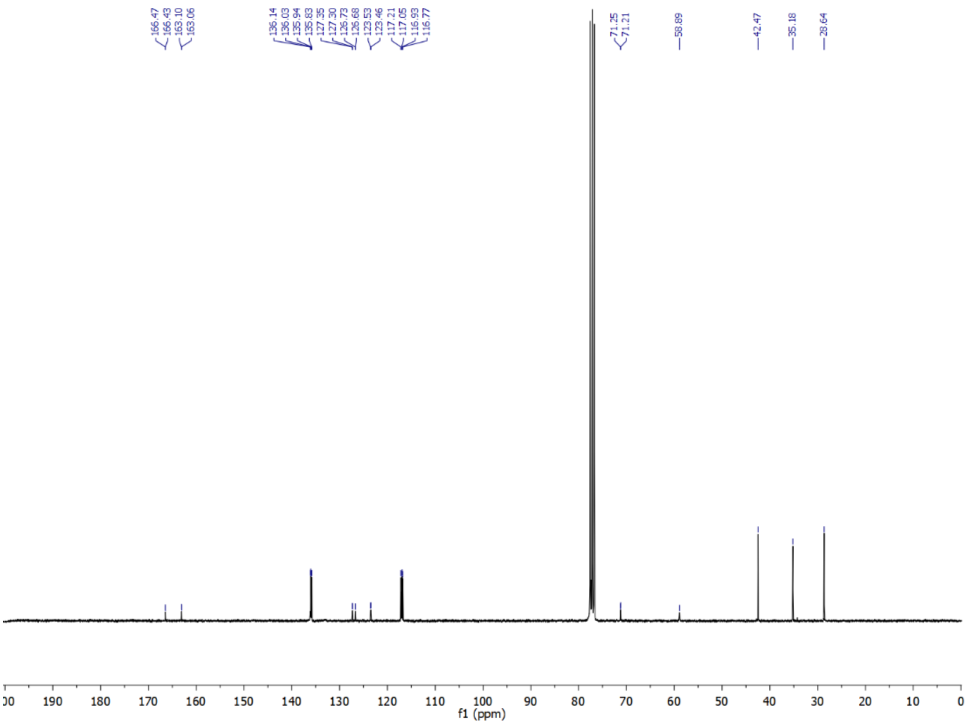

*S 59. $^{13}C\{^{1}H\}$ NMR spectra of compound **6c-Adic** in $CDCl_3$ at 298K*

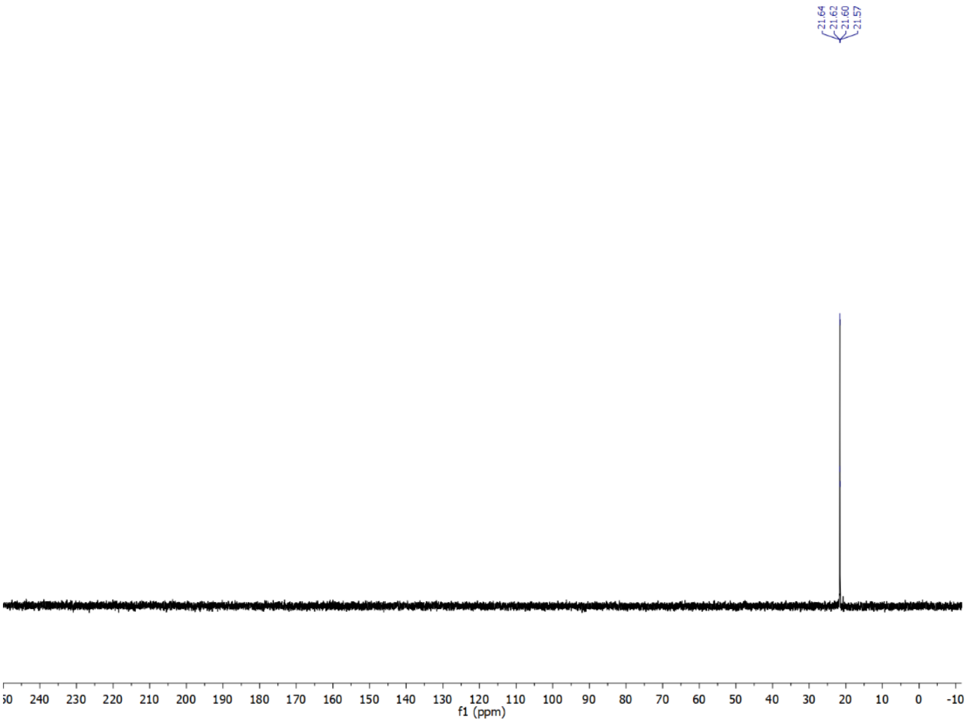

*S 60. $^{31}P\{^{1}H\}$ NMR spectra of compound **6c-Adic** in $CDCl_3$ at 298K*

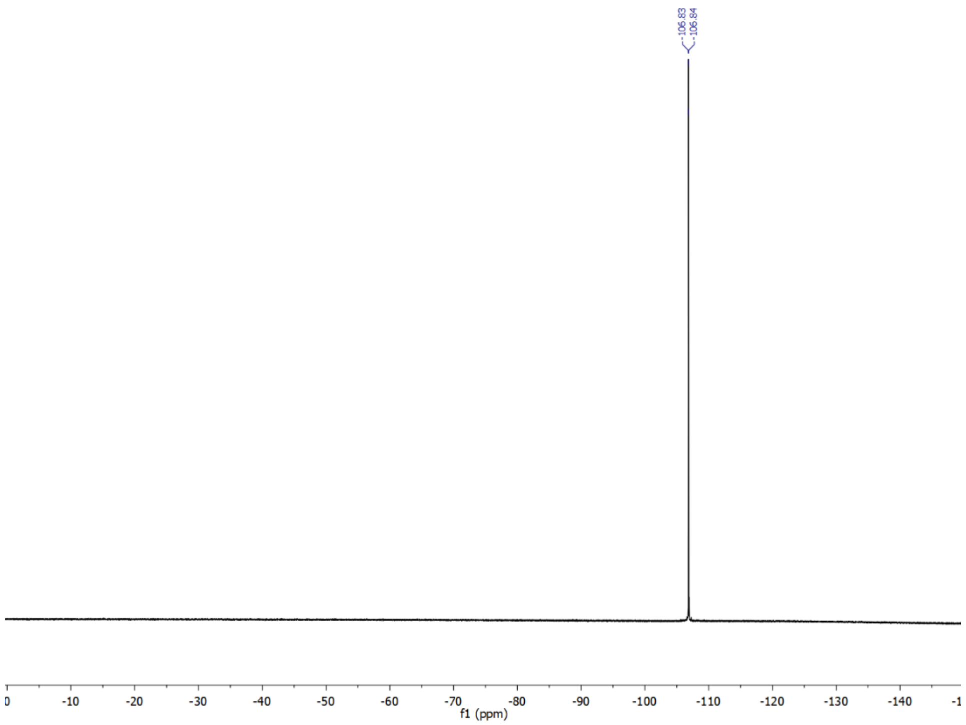

*S 61. $^{19}F\{^{1}H\}$ NMR spectra of compound **6c-Adic** in $CDCl_3$ at 298K*

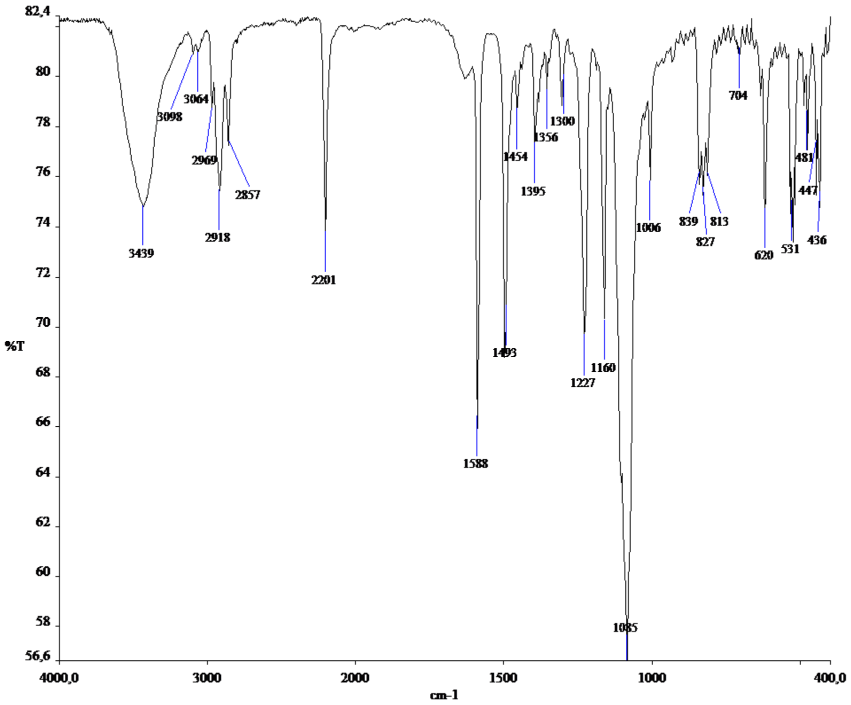

*S 62. IR spectra of compound **6c-Adic***

Table S 1. $\nu(NC)_{free}$, $\nu(NC)_{coord}$ and $\Delta\nu(NC)$ values

Compound	$\nu(NC)_{coord}$ (cm^{-1})	$\nu(NC)_{free}$ (cm^{-1})	$\Delta\nu(NC)$ (cm^{-1})
**3a-Tic**	2212		75
**3b-Tic**	2213	2137	76
**3c-Tic**	2201		64

**3d-Tic**	2212		75
**3a-Cyc**	2207	2140	67
**3b-Cyc**	2212		72
**3c-Cyc**	2207		67
**3d-Cyc**	2207		67
**3a-Adic**	2201	2123	78
**3b-Adic**	2207		84
**3c-Adic**	2218		95
**3d-Adic**	2212		89

Hapticity evaluation

Table S 2. C^{3a} and C^{7a} chemical shifts data and ***$\Delta\delta^{13}C$*** *values and HA, FA and ΔM-C values calculated by X-ray crystal structures*

Compound	δC^{3a}	δC^{7a}	$\Delta\delta^{13}C$	HA (°)	FA (°)	ΔM-C (Å)
**3a-Tic**	130.6	131.8	0.50	13.53	9.20	0.282
**3b-Tic**	131.0	132.0	0.80	13.11	11.18	0.269
**3c-Tic**	131.3	132.4	1.15	14.10	12.33	0.294
**3d-Tic**	130.3	131.7	0.30	13.75	12.23	0.288
**3a-Cyc**	130.6	131.8	0.50	13.36	10.29	0.281
**3b-Cyc**	131.0	131.9	0.75	-	-	-
**3c-Cyc**	130.9	131.9	0.70	14.21	12.36	0.297
**3d-Cyc**	130.5	131.8	0.45	-	-	-
**3a-Adic**	130.7	131.9	0.60	-	-	-
**3b-Adic**	131.0	132.0	0.80	-	-	-
**3c-Adic**	131.0	132.0	0.80	-	-	-
**3d-Adic**	130.5	131.8	0.45	13.82	13.56	0.311

$\Delta\delta^{13}C = \left(\frac{\delta C^{3a} + \delta C^{7a}}{2}\ Ind\right) - \left(\frac{\delta C^{3a} + \delta C^{7a}}{2}\ Na - Ind\right)$; HA (Hinge Angle): is the angle formed between the planes formed by the atoms C^1, C^2, C^3 and C^1, C^3, C^{3a}, C^{7a}; FA (Fold Angle): is the angle formed between the planes formed by the atoms C^1, C^2, C^3 and C^{3a}, C^4, C^5, C^6, C^7, C^{7a}; $\Delta M - C = \left(\frac{M-C^{3a} - M-C^{7a}}{2}\right) - \left(\frac{M-C^1 + M-C^3}{2}\right)$

NMR reactivity data

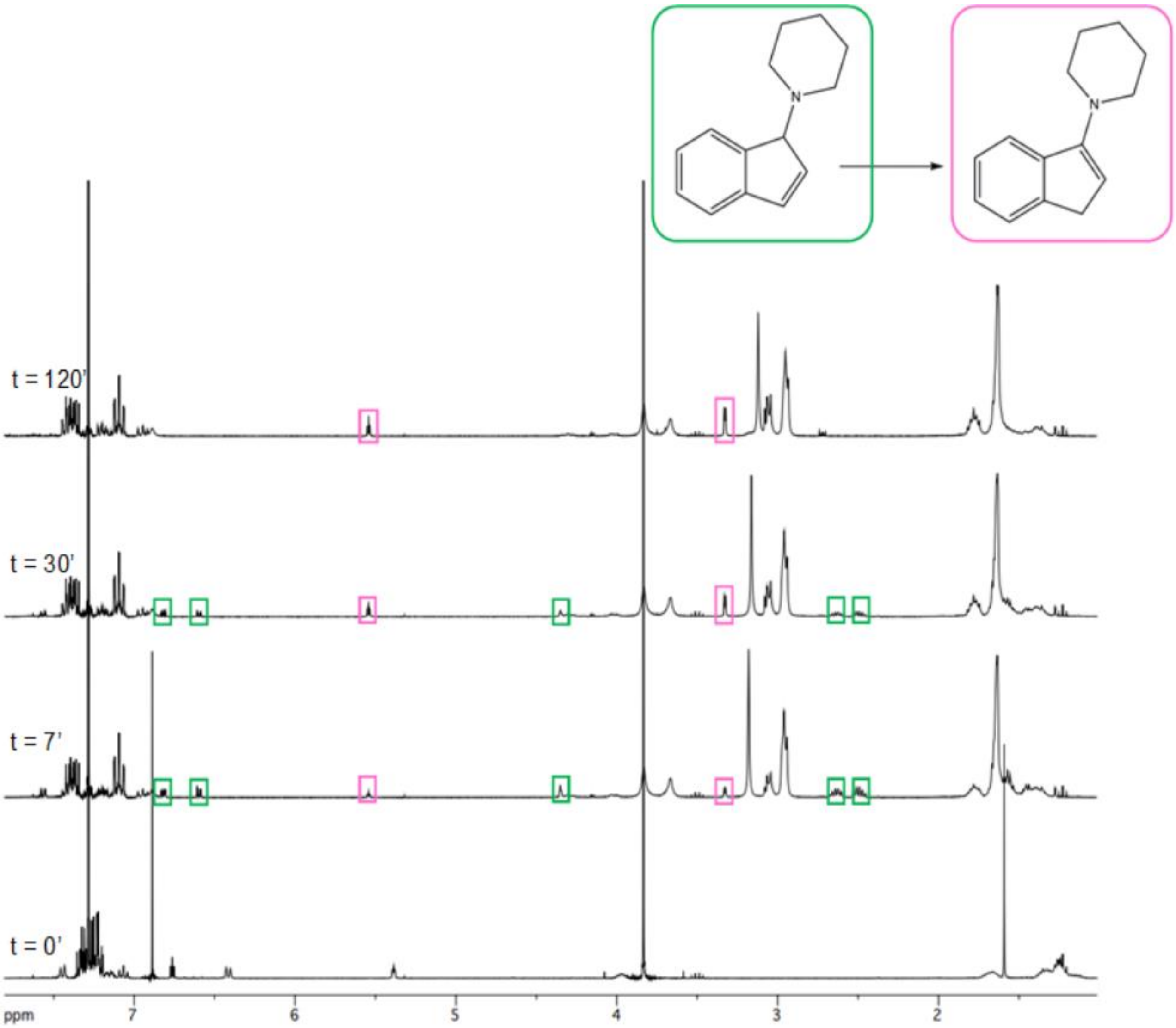

S 63. Amination reaction of compound ***3c-Cyic*** *monitored by NMR spectroscopy (^{1}H NMR spectra in CDCl3 at 298 K)*

HRMS data

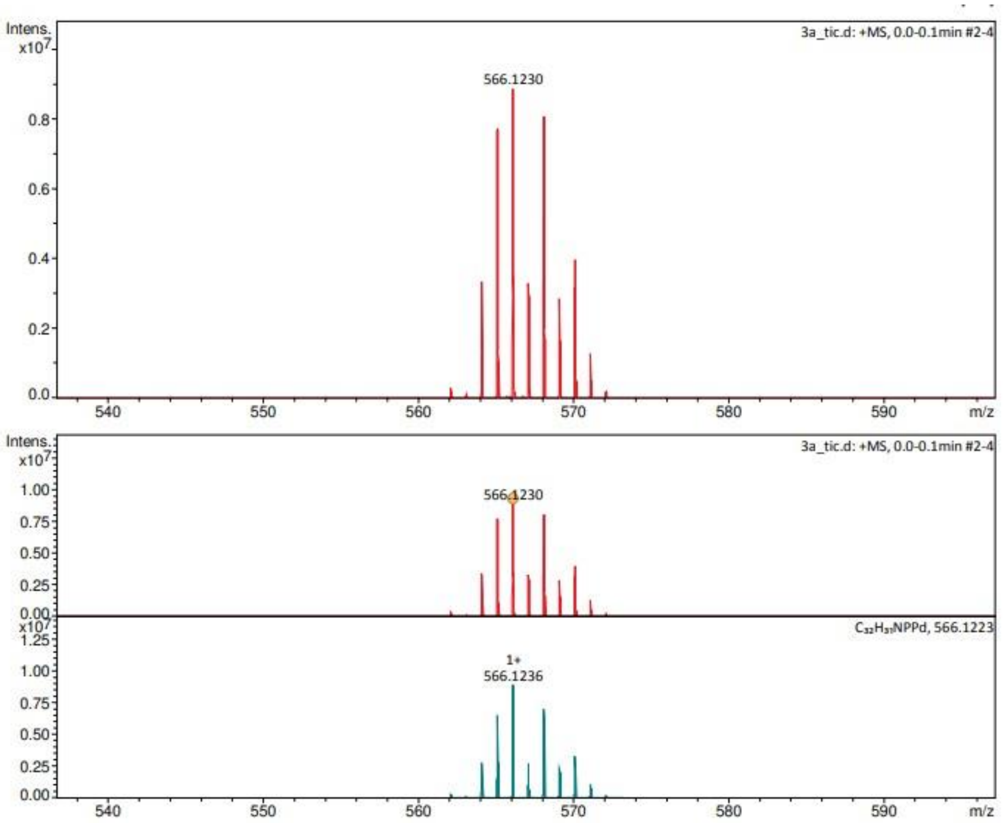

*S 64. HRMS spectra of compound **3a-Tic***

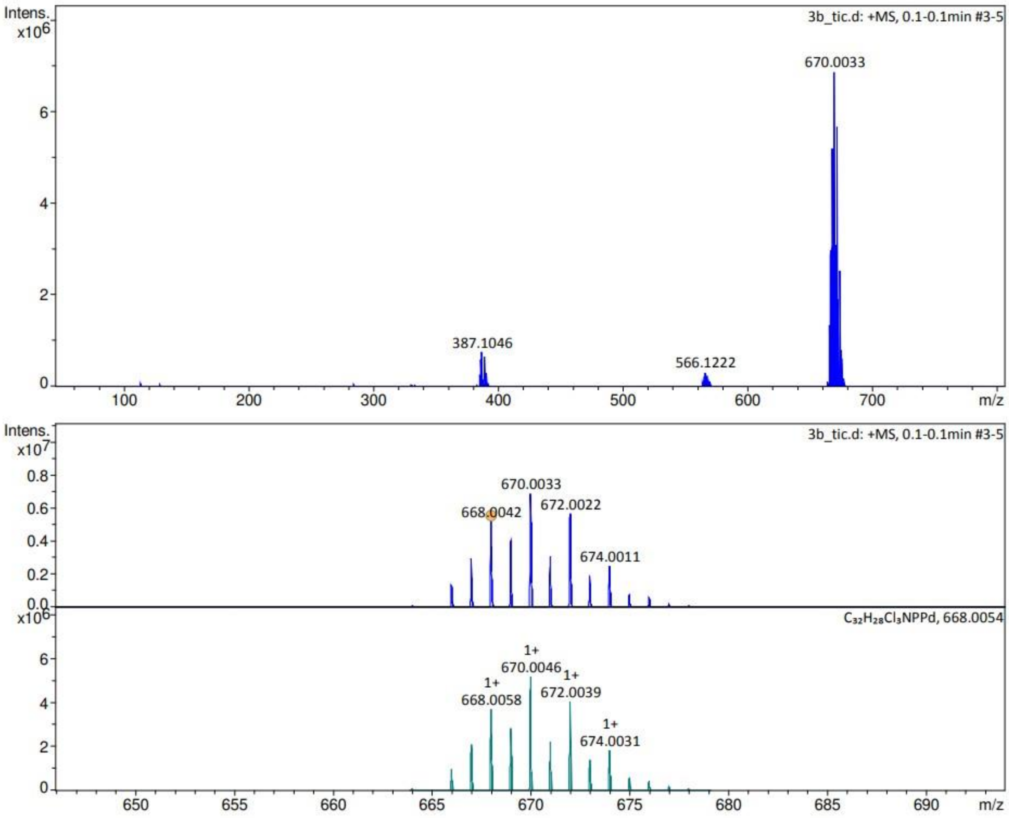

*S 65. HRMS spectra of compound **3b-Tic***

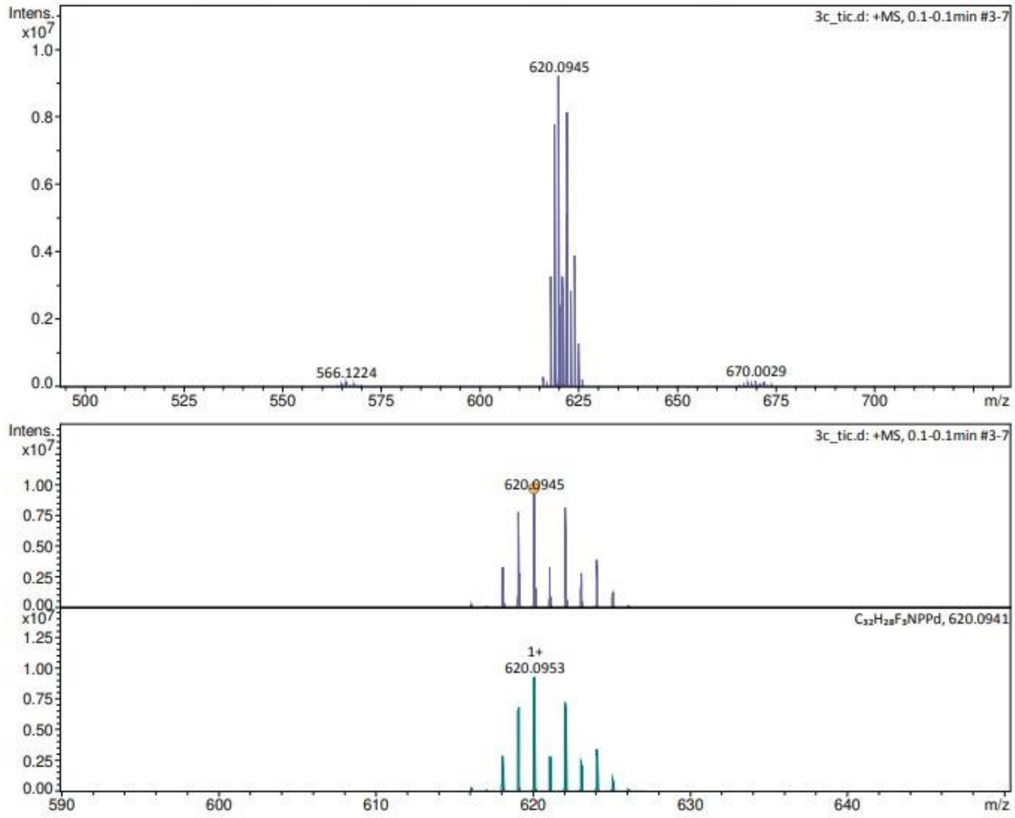

S 66. HRMS spectra of compound ***3c-Tic***

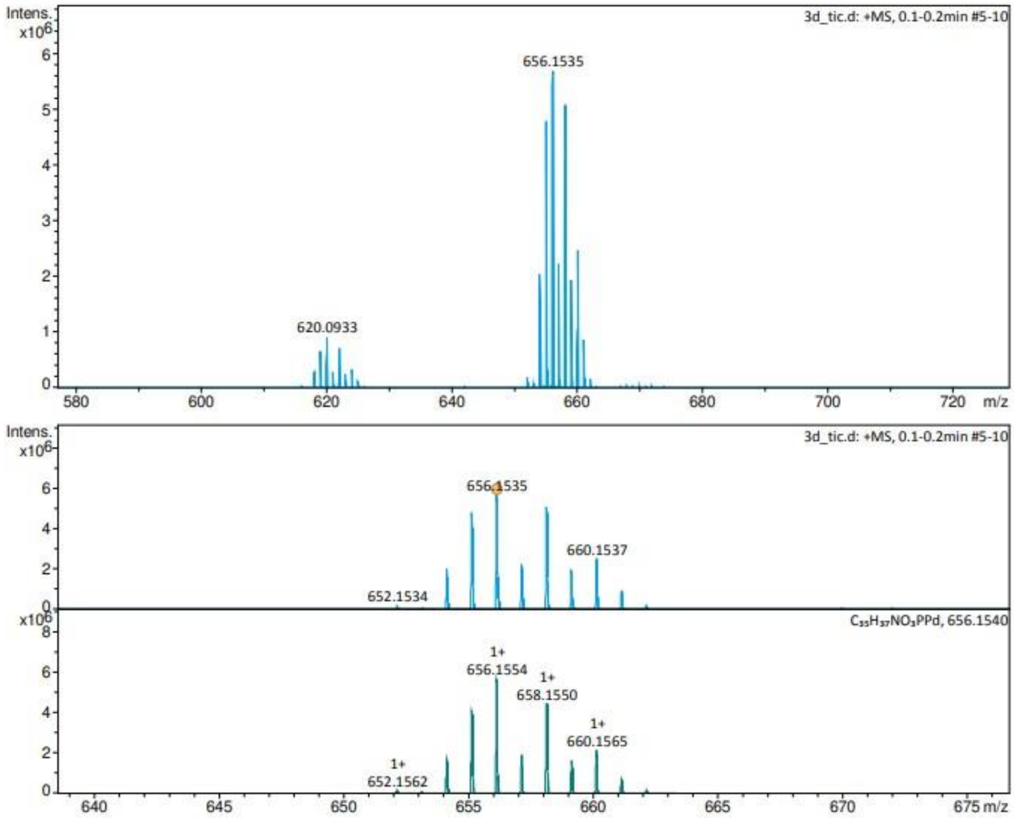

S 67. HRMS spectra of compound ***3d-Tic***

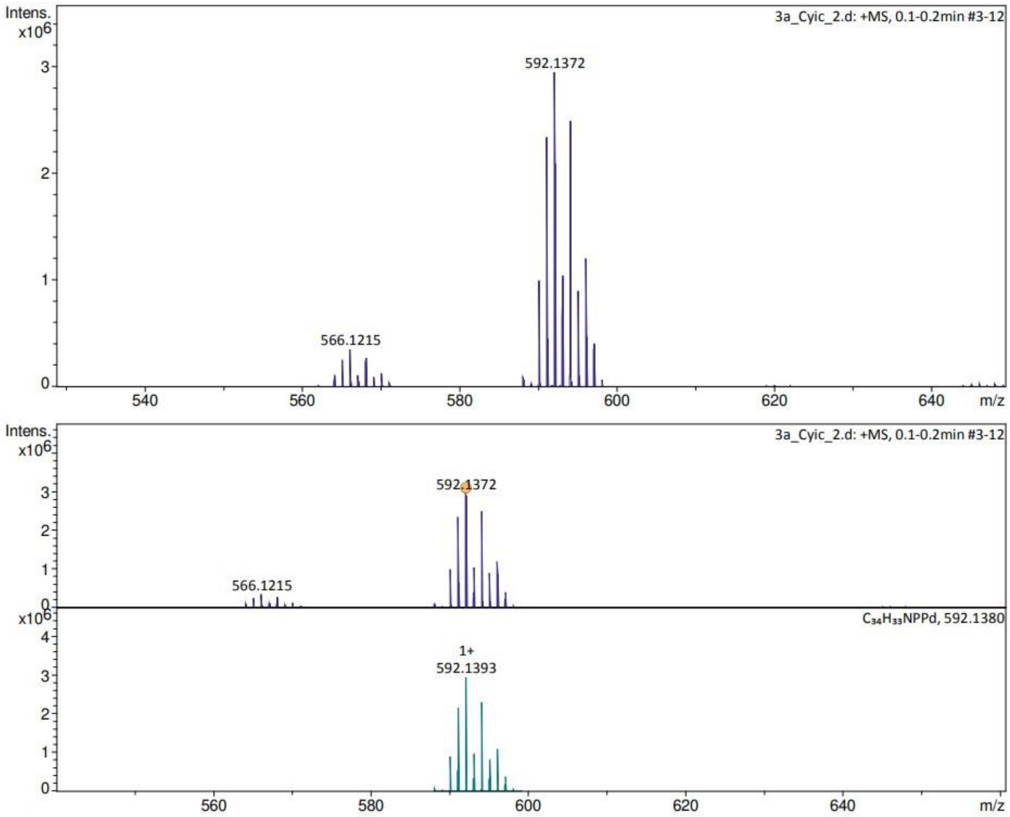

*S 68. HRMS spectra of compound **3a-Cyic***

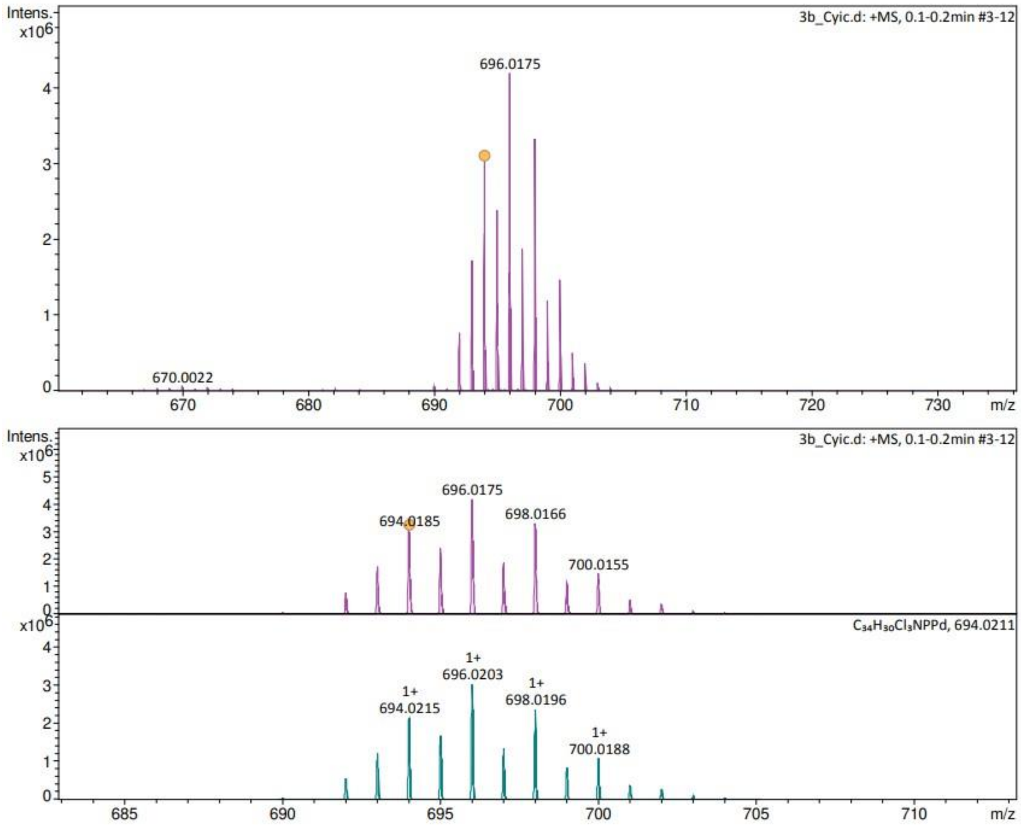

*S 69. HRMS spectra of compound **3b-Cyic***

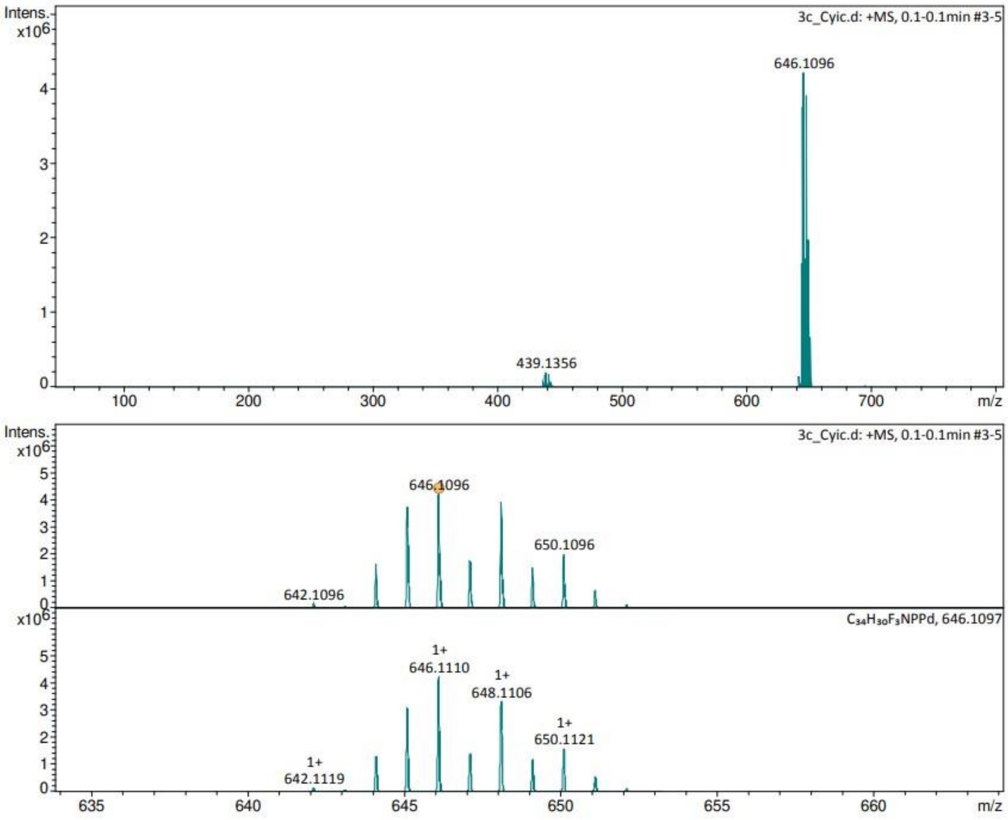

*S 70. HRMS spectra of compound **3c-Cyic***

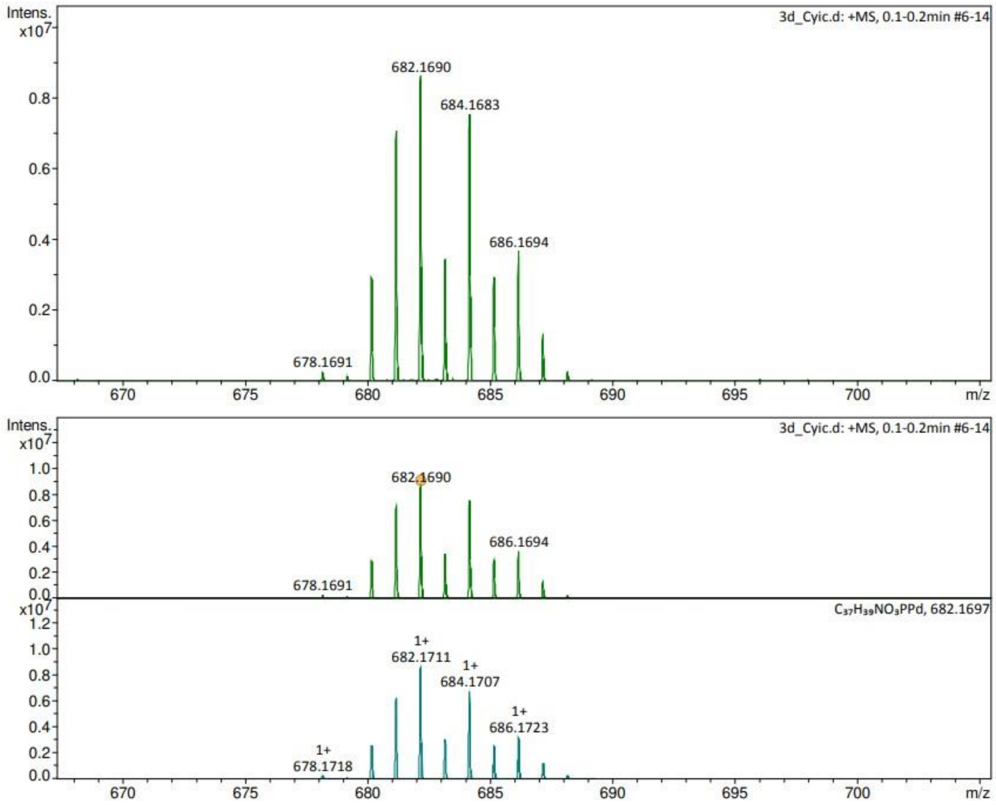

*S 71. HRMS spectra of compound **3d-Cyic***

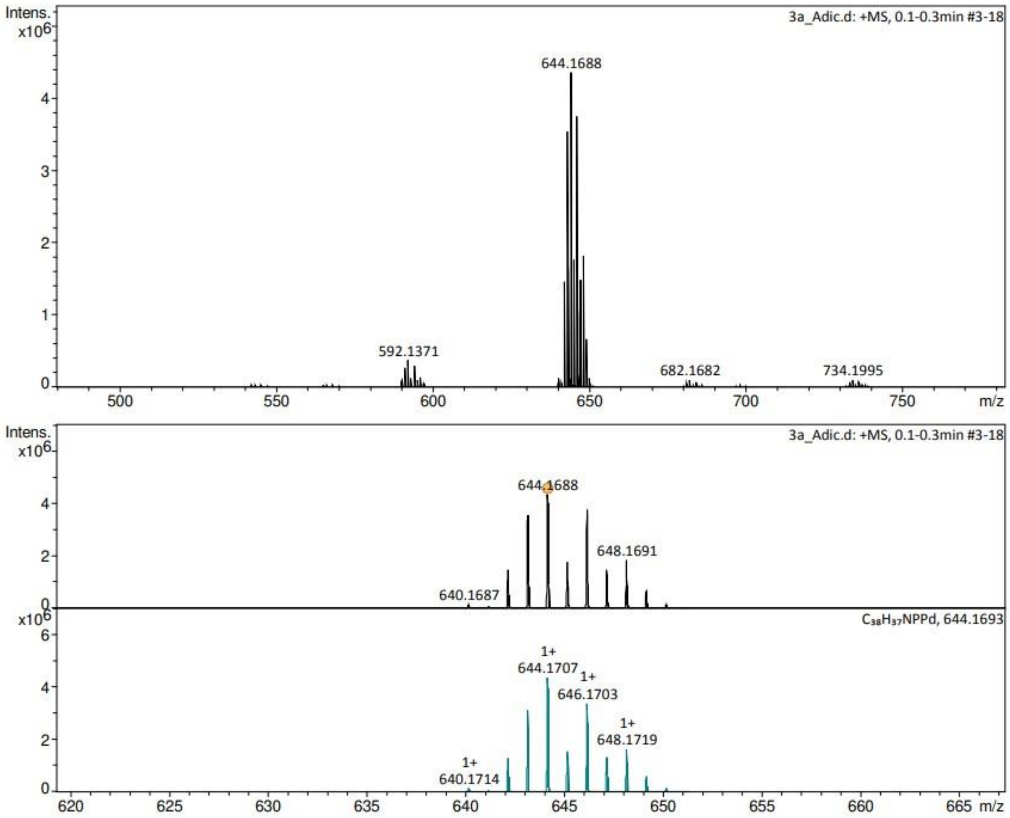

*S 72. HRMS spectra of compound **3a-Adic***

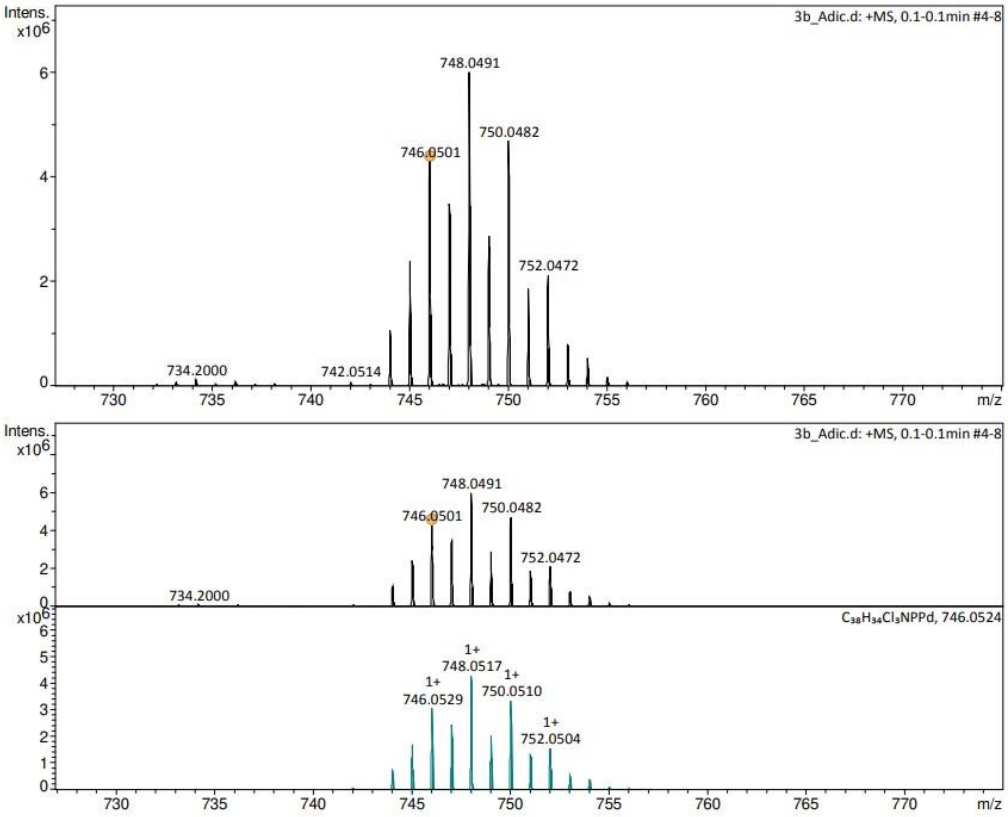

*S 73. HRMS spectra of compound **3b-Adic***

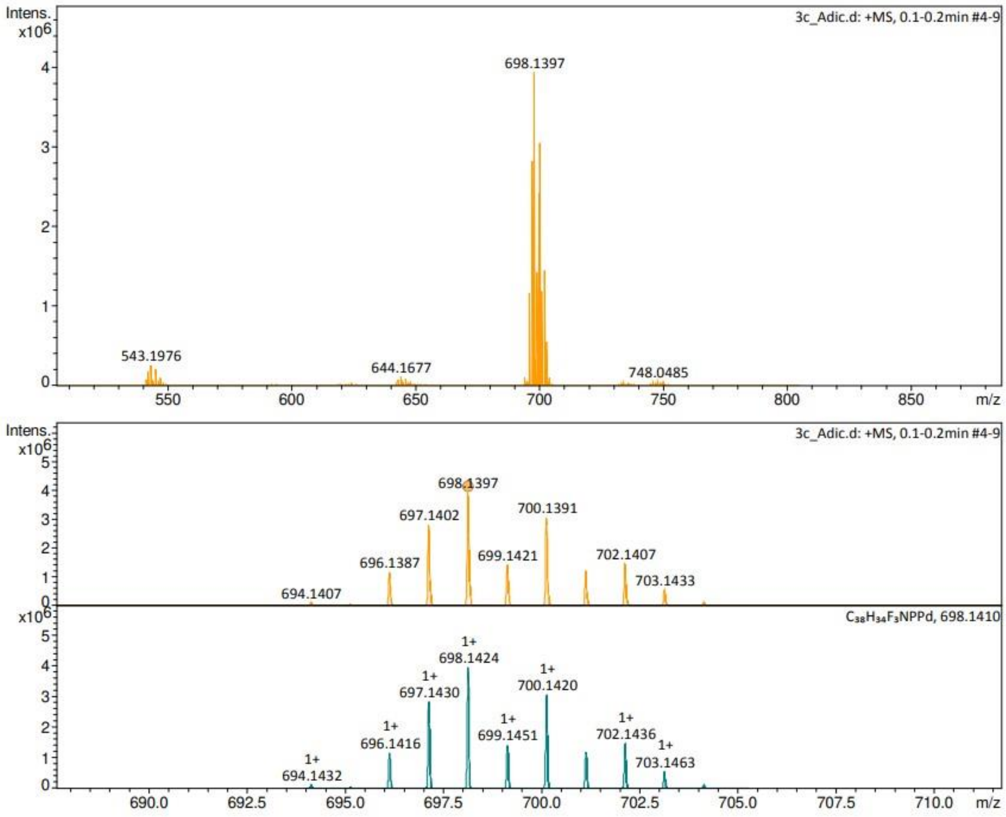

*S 74. HRMS spectra of compound **3c-Adic***

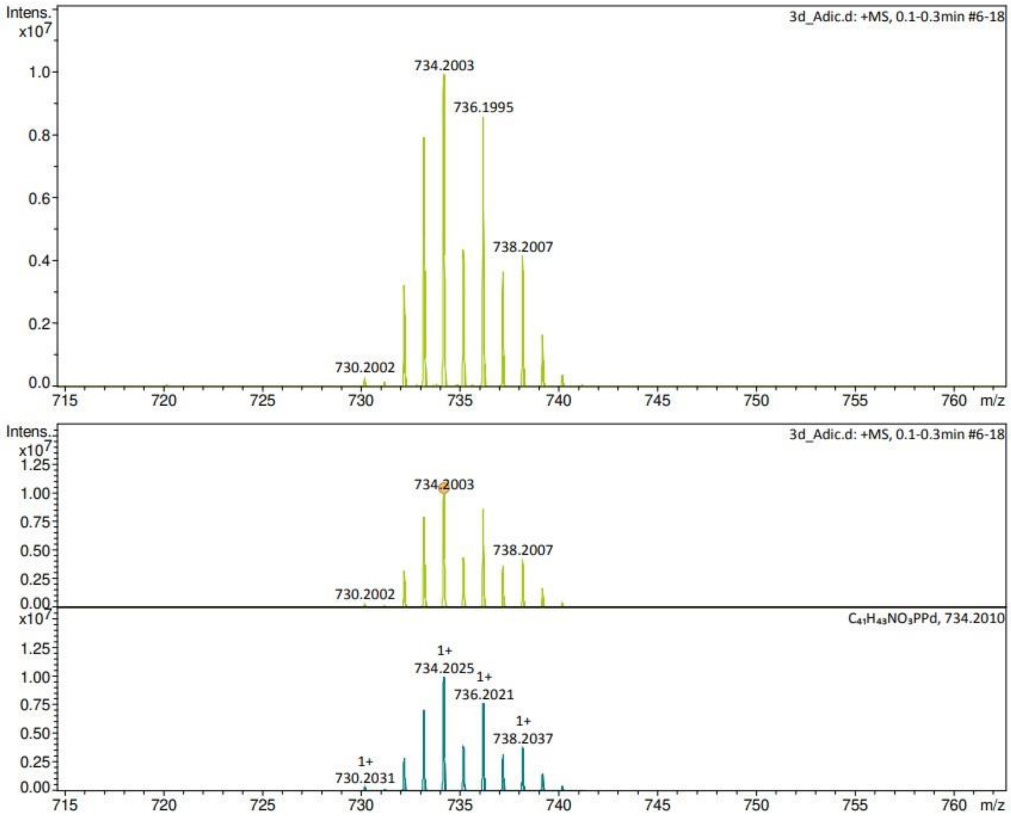

*S 75. HRMS spectra of compound **3d-Adic***

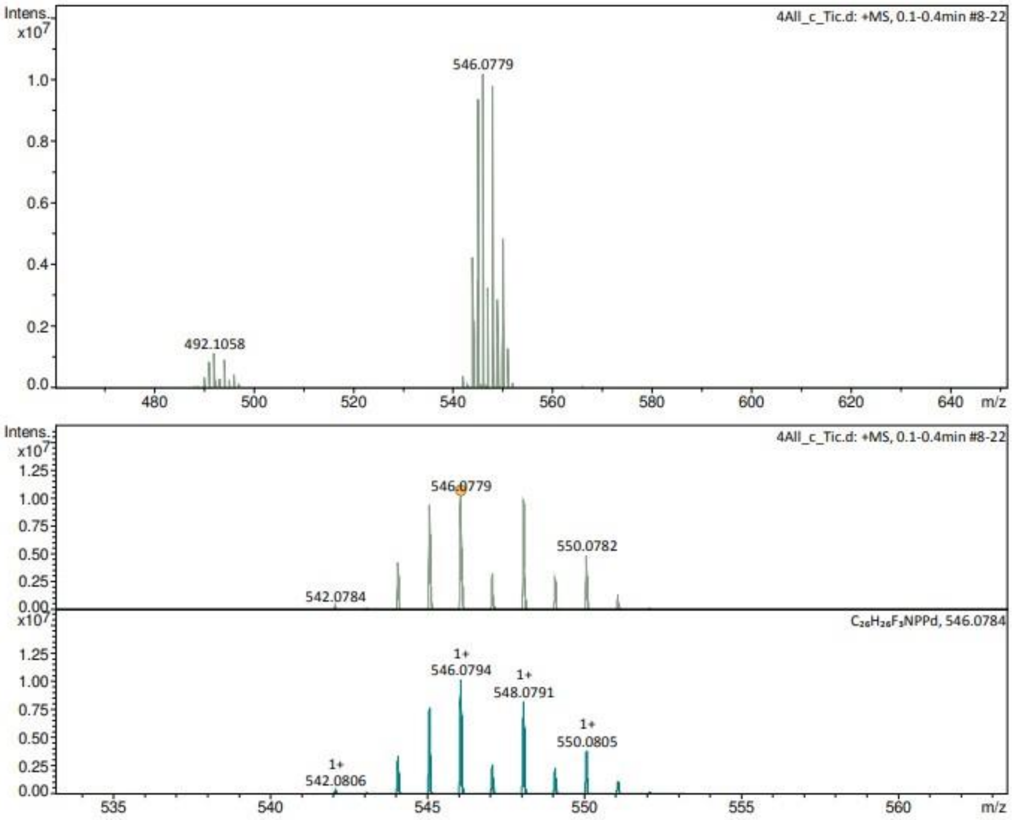

S 76. HRMS spectra of compound ***6c-Tic***

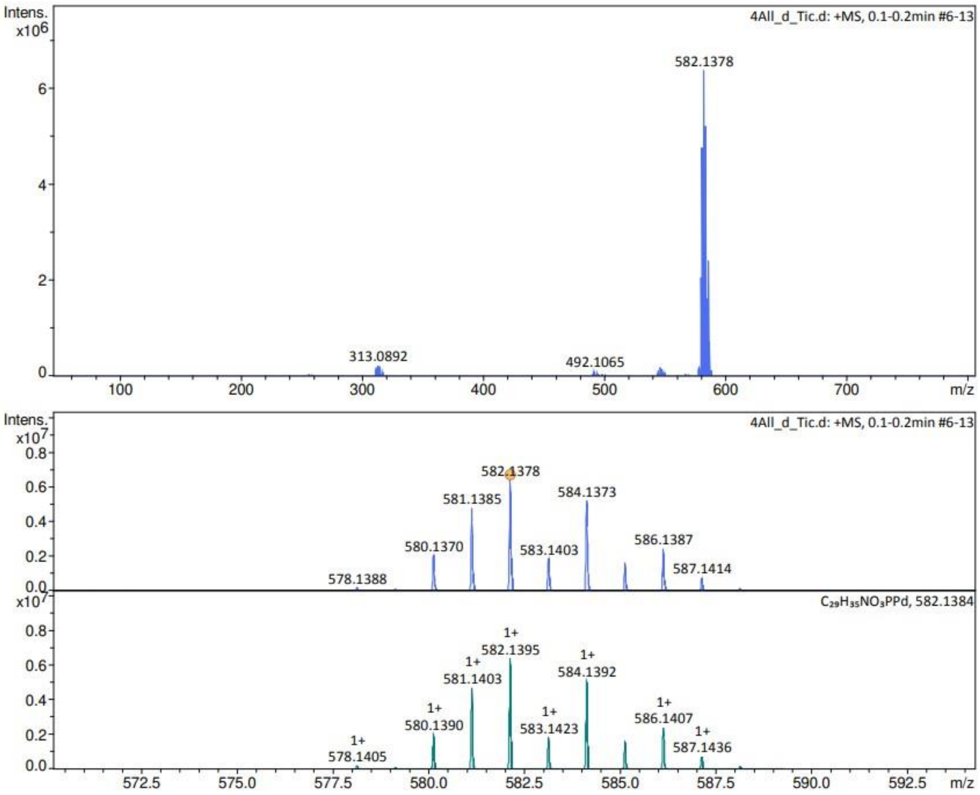

S 77. HRMS spectra of compound ***6d-Tic***

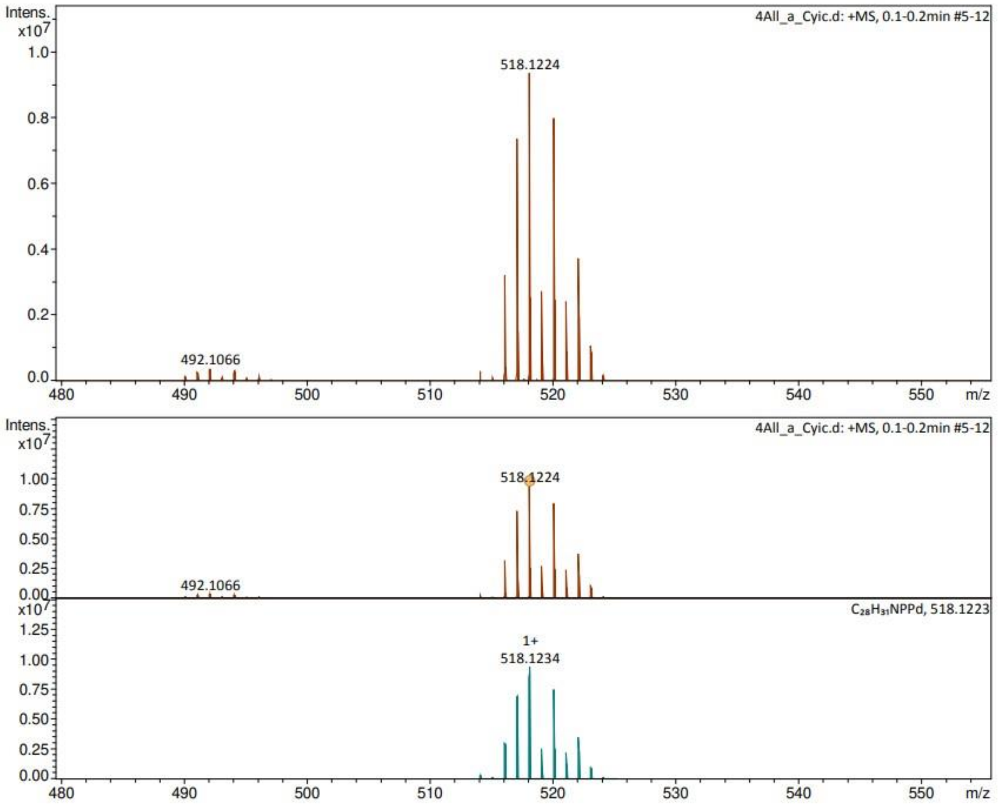

*S 78. HRMS spectra of compound **6a-Cyic***

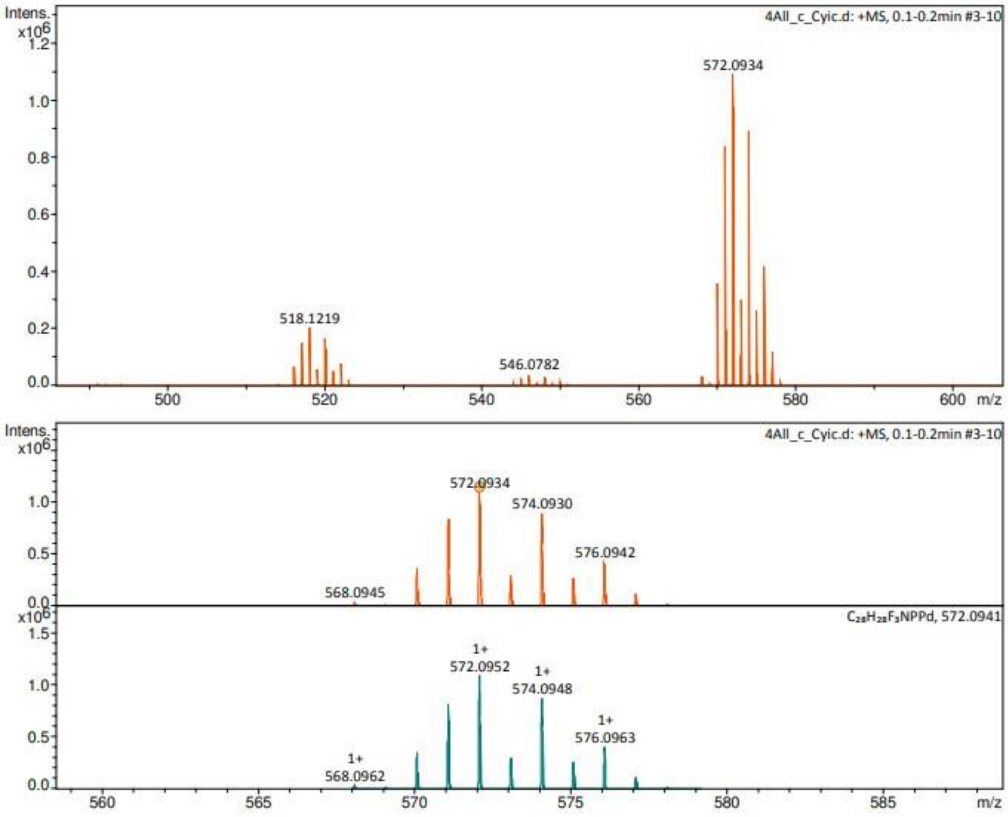

*S 79. HRMS spectra of compound **6c-Cyic***

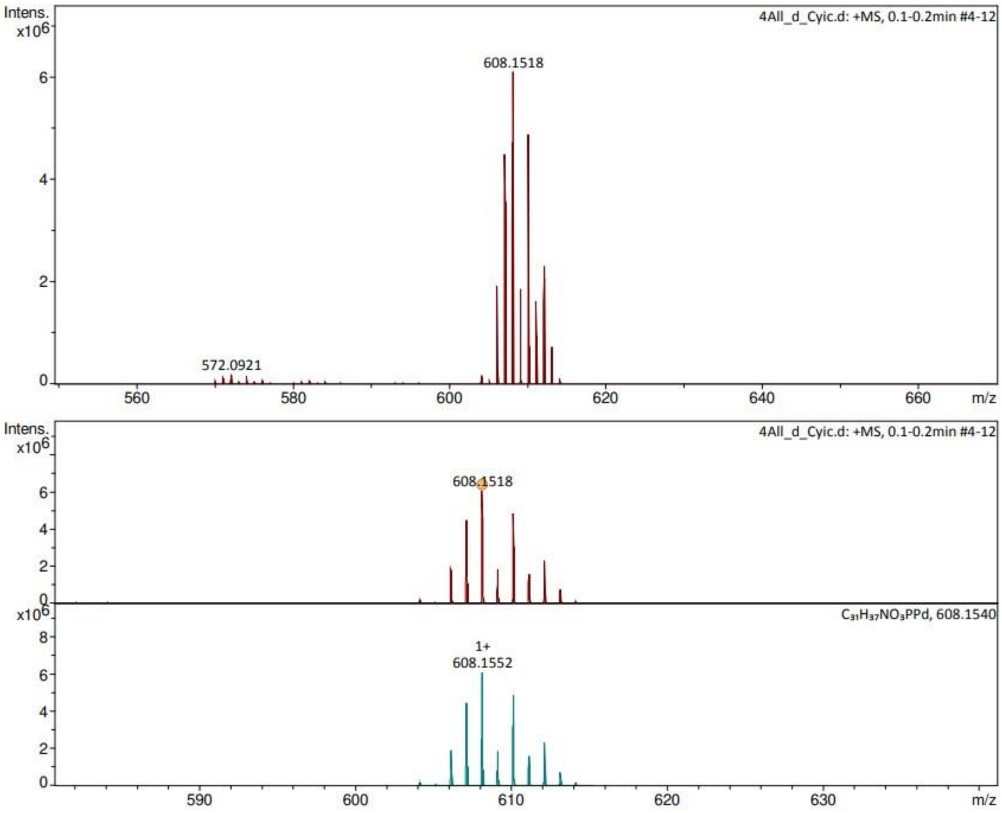

S 80. HRMS spectra of compound ***6d-Cyic***

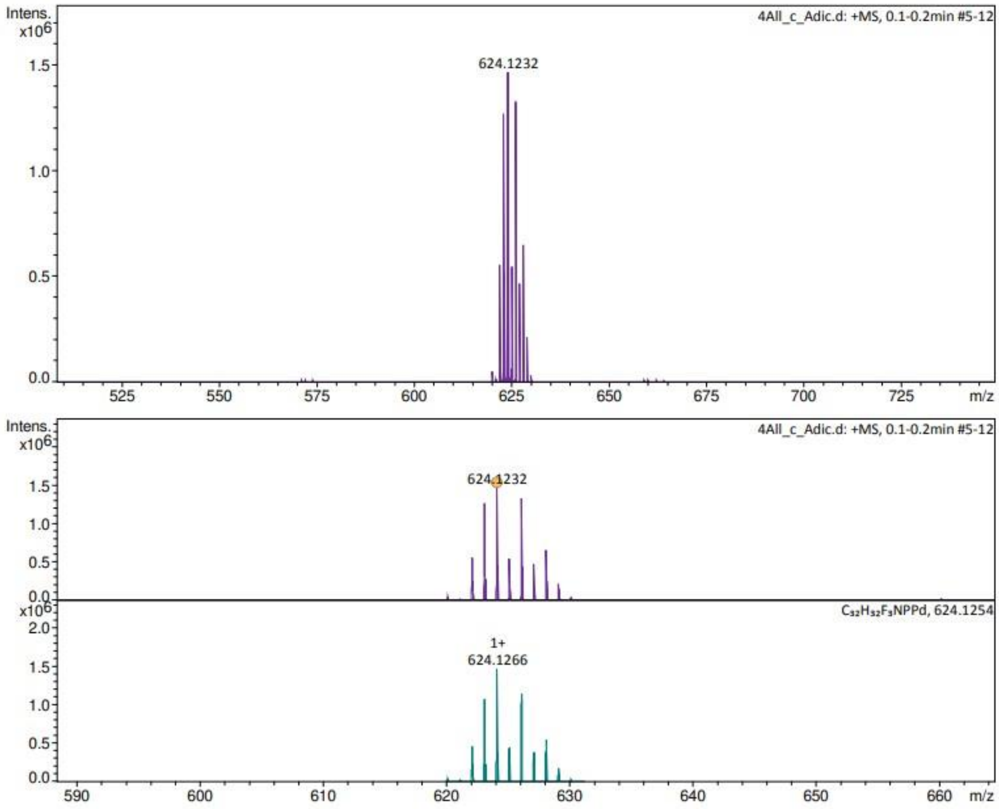

S 81. HRMS spectra of compound ***6c-Adic***

Crystal Structure Determination

Table S 3. Crystallographic data

Compound	3a-Tic@100 K	3c-Tic@100 K	3d-Tic@100 K	i3d-Tic@100 K	3b-Tic@298 K
Formula	$[PdC_{32}H_{31}NP](ClO_4)\cdot CHCl_3$	$[PdC_{32}H_{28}F_3NP](ClO_4)$	$[PdC_{35}H_{37}NO_3P](ClO_4)$	$[PdC_{35}H_{37}NO_3P](ClO_4)$	$[PdC_{32}H_{28}Cl_3NP](ClO_4)$
M/g·mol^{-1}	785.76	720.37	756.47	756.47	7769.72
Space group	*P* 2$_1$	*P* 2$_1$/*c*	*P ca*2$_1$	*P ca*2$_1$	*P* 2$_1$/*n*
Crystal system	Monoclinic	Monoclinic	Orthorhombic	Orthorhombic	Monoclinic
a/Å	9.905(2)	15.006(3)	19.569(4)	19.544(4)	10.170(2)
b/Å	34.151(7)	10.701(2)	9.821(2)	9.842(2)	18.904(4)
c/Å	9.978(2)	18.944(4)	17.575(4)	17.561(4)	17.274(3)
α/°	90	90	90	90	90
β/°	90.58(3)	95.65(3)	90	90	90.09(3)
γ/°	90	90	90	90	90
V/Å^3	3375.0(12)	3027.2(11)	3377.7(12)	3377.9(12)	3321.0(11)
Z	4	4	4	4	4
T/K	100(2)	100(2)	100(2)	100(2)	298(2)
D_c/g·cm^{-3}	1.546	1.581	1.488	1.488	1.539
F(000)	1592	1456	1552	1552	1552
μ/mm^{-1}	0.647	0.556	0.497	0.497	0.656
Measured Reflections	79911	66652	77299	77478	78135
Unique Reflections	28759	13152	15352	15086	14452
R_{int}	0.0357	0.0296	0.0194	0.0388	0.0341
Obs. Refl.ns [I≥2σ(I)]	27801	12859	14379	13238	8661
$θ_{min}$- $θ_{max}$/°	1.04 – 31.12	1.19 – 31.10	1.81 – 31.10	1.81 – 31.10	1.39 – 31.10
hkl ranges	-15,15; -56,56; -16,16	-24,25; -17,17; -28,29	-32,32; -15,15; -27,27	-30,30; -16,16; -29,29	-16,16; -31,31; -26,26
R(F^2) (Obs.Refl.ns)	0.0343	0.0262	0.0252	0.0332	0.0591
wR(F^2) (All Refl.ns)	0.0866	0.0690	0.0660	0.0861	0.2077
No. Variables	799	392	467	467	404
Goodness of fit	1.008	1.076	1.062	1.083	1.031
$Δρ_{max}$; $Δρ_{min}$/e·Å^{-3}	2.23; -1.45	0.67; -1.09	0.99; -1.07	0.76; -1.47	1.07; -1.24
CCDC Deposition N.	2290052	2290053	2290054	2290061	2290055

Compound	3c-Cyic@100 K	3d-Adic@100 K	3d-Adic@298 K	3a-Cyic@100 K	3a-Cyic@298 K
Formula	$[PdC_{34}H_{30}F_3NP](ClO_4)$	$[PdC_{41}H_{43}NO_3P](ClO_4)$	$[PdC_{41}H_{43}NO_3P](ClO_4)$	$[PdC_{34}H_{33}NP](ClO_4)$	$[PdC_{34}H_{33}NP](ClO_4)$
M/g·mol^{-1}	746.41	834.58	834.58	692.43	692.43
Space group	*P* 2$_1$/*c*	*P* -1	*P* -1	*P* 2$_1$/*c*	*P* 2$_1$/*c*
Crystal system	Monoclinic	Triclinic	Triclinic	Monoclinic	Monoclinic
a/Å	15.295(3)	12.091(2)	12.186(2)	14.982(3)	15.157(3)
b/Å	10.691(2)	12.295(2)	12.335(2)	13.237(3)	13.336(3)
c/Å	19.120(4)	12.729(3)	13.003(3)	16.829(3)	17.127(3)
α/°	90	82.90(3)	83.54(3)	90	90
β/°	99.74(3)	82.85(3)	81.05(3)	112.78(3)	112.74(3)
γ/°	90	89.24(3)	88.43(3)	90	90
V/Å^3	3081.5(11)	1863.0(7)	1918.6(7)	3077.2(12)	3192.8(13)
Z	4	2	2	4	4
T/K	100(2)	100(2)	298(2)	100(2)	298(2)
D_c/g·cm^{-3}	1.609	1.488	1.445	1.495	1.441
F(000)	1512	860	860	1416	1416
μ/mm^{-1}	0.548	0.457	0.444	0.535	0.516
Measured Reflections	71053	63227	89581	34405	75154
Unique Reflections	13566	16033	16038	13007	14031
R_{int}	0.0189	0.0631	0.0392	0.0395	0.0314
Obs. Refl.ns [I≥2σ(I)]	12785	13563	14298	11398	11737
$θ_{min}$- $θ_{max}$/°	1.18 – 31.10	1.42– 31.17	1.39 – 31.10	1.76 – 31.10	1.74 – 31.09
hkl ranges	-25,25; -17,17; -3!,31	-17,20; -19,20; -21,21	-18,18; -19,19; -20,20	-24,22; -21,21; -26,28	-23,23; -21,21; -26,26
R(F^2) (Obs.Refl.ns)	0.0260	0.0431	0.0405	0.0746	0.0351
wR(F^2) (All Refl.ns)	0.0741	0.1214	0.1263	0.2458	0.1150
No. Variables	406	519	569	409	381
Goodness of fit	1.051	1.085	1.042	1.138	1.041
$Δρ_{max}$; $Δρ_{min}$/e·Å^{-3}	0.88; -1.24	2.15; -1.59	0.83; -0.92	2.70; -4.08	0.69; -0.67
CCDC Deposition N.	2290056	2290058	2290057	2290059	2290060

*Table S 4. Selected palladium distances and angles for **3a-Tic** at 100 K*

3a-Tic (100 K) - [$PdC_{32}H_{31}NP$](ClO_4)							
ASU Molecule 1				ASU Molecule 2			
**Distances**	(Å)	**Angles**	(°)	**Distances**	(Å)	**Angles**	(°)
Pd_11-P_14	2.286(1)	P_14-Pd_11-C_12	91.06(9)	Pd_21-P_24	2.282(1)	P_24-Pd_21-C_22	89.73(9)
Pd_11-C_12	1.983(3)	C1_13-Pd_11-C3_13	61.9(1)	Pd_21-C_22	1.983(3)	C1_23-Pd_21-C3_23	62.0(1)
Pd_11-C1_13	2.245(3)	P_14-Pd_11-C3_13	102.98(8)	Pd_21-C1_23	2.258(3)	P_24-Pd_21-C3_23	101.21(8)
Pd_11-C3_13	2.201(3)	C_12-Pd_11-C1_13	104.6(1)	Pd_21-C3_23	2.190(3)	C_22-Pd_21-C1_23	106.6(1)
Pd_11-C2_13	2.228(3)	HA[b]	13.25(24)	Pd_21-C2_23	2.230(3)	HA[b]	13.52(24)
C=C_Indenyl[a]	1.415(4)	FA[b]	9.84(25)	C=C_Indenyl[a]	1.416(4)	FA[b]	9.20(24)
ΔM···C[b]	0.280(6)	Pd-Indenyl Ave Plane[d]	85.20(6)	ΔM···C[b]	0.283(6)	Pd-Indenyl Ave Plane[d]	84.44(7)
Pd_11···ClO_4^-	4.390(3)			Pd_21···ClO_4^-	4.399(4)		

[a]Average C1=C2=C3 bond length in indenyl ligand; [b]As defined in Table 1 footnotes of Zargarian Coordination Chemistry Reviews 233-234 (2002) 157-176; [c]Shortest distance between palladium centre and anion; [d]Average angle among the mean metal coordination plane and the mean indenyl plane

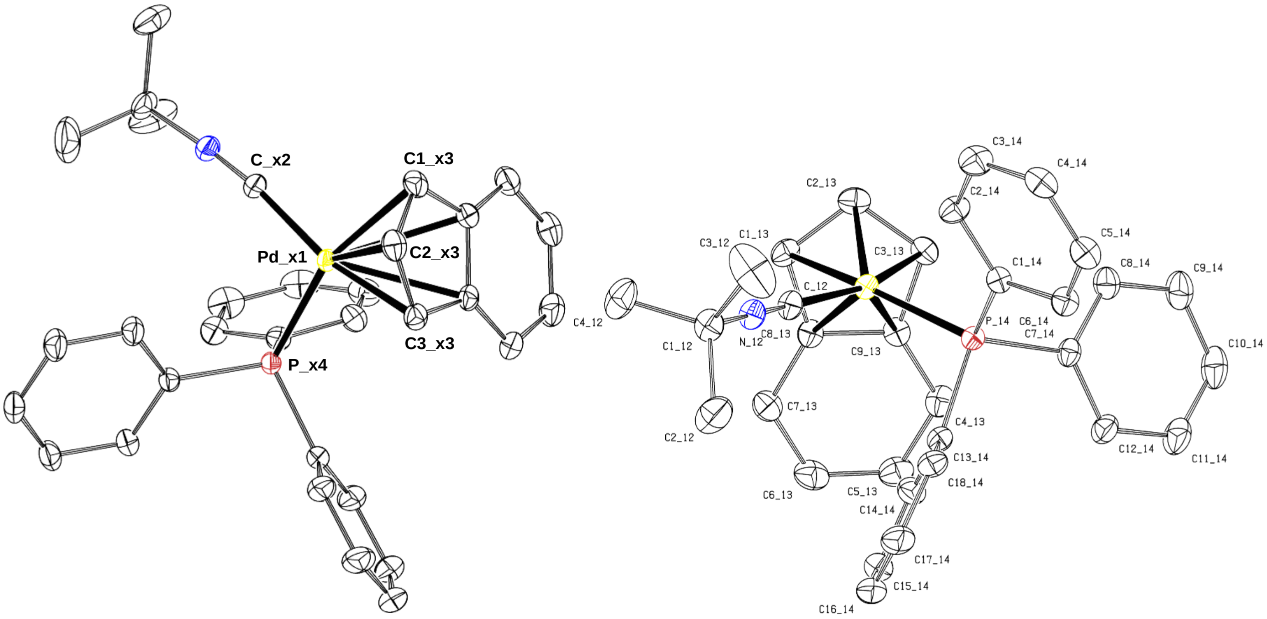

Table S 5. Selected palladium distances and angles for ***3c-Tic*** *at 100 K*

3c-Tic (100 K) - [$PdC_{32}H_{28}F_3NP$](ClO_4)

Distances	(Å)	Angles	(°)
Pd_1-P_4	2.288(1)	P_4-Pd_1-C_2	95.90(3)
Pd_1-C_2	1.978(1)	C1_3-Pd_1-C3_3	62.03(4)
Pd_1-C1_3	2.239(1)	P_4-Pd_1-C3_3	98.81(3)
Pd_1-C3_3	2.184(1)	C_2-Pd_1-C1_3	103.18(4)
Pd_1-C2_3	2.226(1)	HA[b]	14.10(7)
C=C_Indenyl[a]	1.416(1)	FA[b]	12.26(7)
ΔM···C[b]	0.295(2)	Pd-Indenyl Ave Plane[d]	88.20(2)
Pd_1···ClO_4^-	4.600(1)		

[a]Average C1=C2=C3 bond length in indenyl ligand
[b]As defined in Table 1 footnotes of Zargarian Coordination Chemistry Reviews 233-234 (2002) 157-176
[c]Shortest distance between palladium centre and anion
[d]Average angle among the mean metal coordination plane and the mean indenyl plane

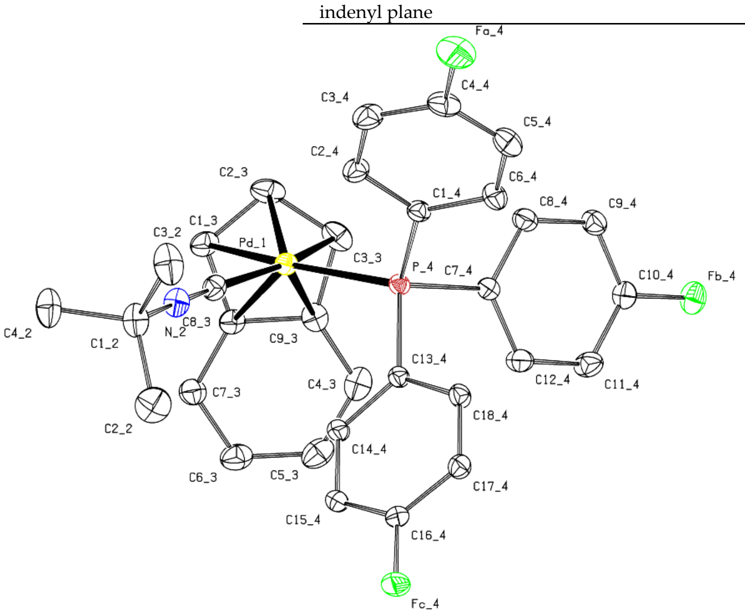

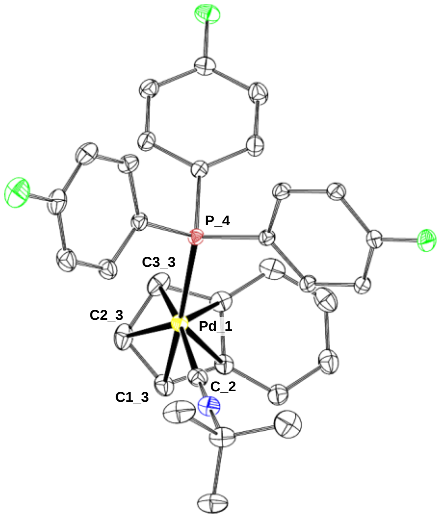

Table S 6. Selected palladium distances and angles for ***3d-Tic*** *at 100 K*

3d-Tic (100 K) - $[PdC_{35}H_{37}NO_3P](ClO_4)$							
**3d-Tic**				**i3d-Tic***			
**Distances**	(Å)	**Angles**	**(°)**	**Distances**	(Å)	**Angles**	**(°)**
Pd_1-P_4	2.287(1)	P_4-Pd_1-C_2	92.19 (5)	Pd_1-P_4	2.286(1)	P_4-Pd_1-C_2	92.23(8)
Pd_1-C_2	1.963(2)	C1_3-Pd_1-C3_3	61.10(7)	Pd_1-C_2	1.966(3)	C1_3-Pd_1-C3_3	62.2(1)
Pd_1-C1_3	2.237(2)	P_4-Pd_1-C3_3	101.52(5)	Pd_1-C1_3	2.232(3)	P_4-Pd_1-C3_3	101.42(8)
Pd_1-C3_3	2.200(2)	C_2-Pd_1-C1_3	104.31(7)	Pd_1-C3_3	2.204(3)	C_2-Pd_1-C1_3	104.2(1)
Pd_1-C2_3	2.228(2)	HA[b]	13.75(13)	Pd_1-C2_3	2.227(3)	HA[b]	13.73(22)
C=C_Indenyl[a]	1.420(4)	FA[b]	12.19(12)	C=C_Indenyl[a]	1.420(5)	FA[b]	12.02(20)
ΔM···C[b]	0.289(4)	Pd-Indenyl Ave Plane[d]	88.98(4)	ΔM···C[b]	0.287(6)	Pd-Indenyl Ave Plane[d]	88.69(6)
Pd_1···ClO_4^-	4.335(6)			Pd_1···ClO_4^-	4.49(1)		

[a]Average C1=C2=C3 bond length in indenyl ligand; [b]As defined in Table 1 footnotes of Zargarian Coordination Chemistry Reviews 233-234 (2002) 157-176; [c]Shortest distance between palladium centre and anion; [d]Average angle among the mean metal coordination plane and the mean indenyl plane; *Enantiomorphic packing

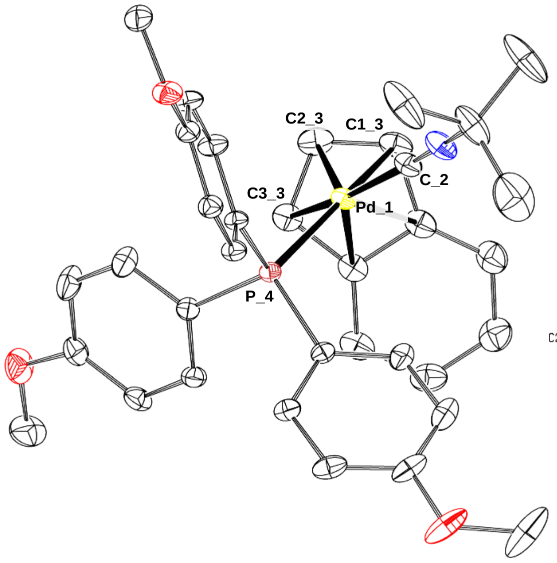

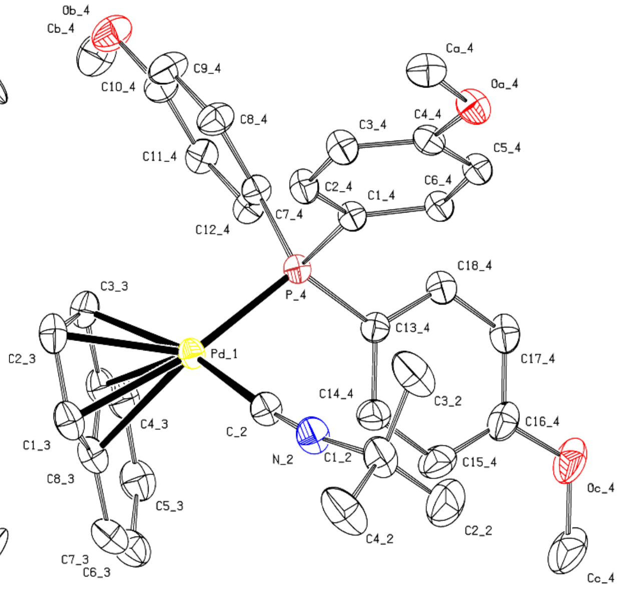

*Table S 7. Selected palladium distances and angles for **3b-Tic** at 298 K*

3b-Tic (298 K) - [$PdC_{32}H_{28}Cl_3NP$](ClO_4)

**Distances**	(Å)	**Angles**	(°)
Pd_1-P_4	2.283(1)	P_4-Pd_1-C_2	91.90(9)
Pd_1-C_2	1.974(3)	C1_3-Pd_1-C3_3	61.6(1)
Pd_1-C1_3	2.259(3)	P_4-Pd_1-C3_3	100.72(8)
Pd_1-C3_3	2.187(3)	C_2-Pd_1-C1_3	105.6(1)
Pd_1-C2_3	2.236(3)	HA[b]	13.12(23)
C=C_Indenyl[a]	1.408(6)	FA[b]	11.13(21)
ΔM···C[b]	0.270(6)	Pd-Indenyl Ave Plane[d]	86.92(6)
Pd_1···ClO_4^-	4.613(9)		

[a]Average C1=C2=C3 bond length in indenyl ligand
[b]As defined in Table 1 footnotes of Zargarian Coordination Chemistry Reviews 233-234 (2002) 157-176
[c]Shortest distance between palladium centre and anion
[d]Average angle among the mean metal coordination plane and the mean indenyl plane

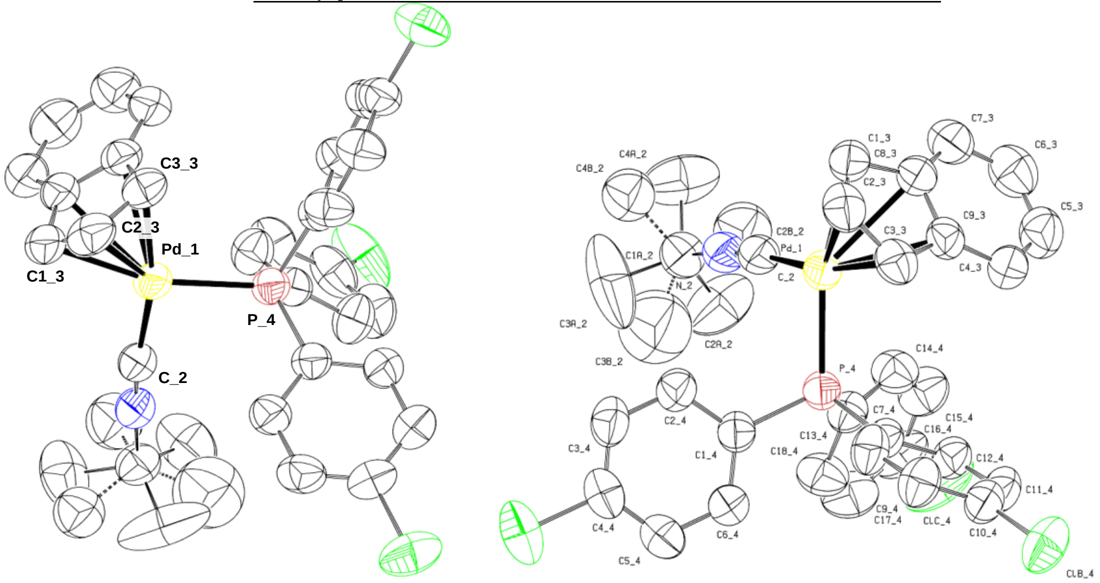

*Table S 8. Selected palladium distances and angles for **3c-Cyic** at 100 K*

3c-Cyic (100 K) - [$PdC_{34}H_{30}F_3NP](ClO_4)$

Distances	(Å)	Angles	(°)
Pd_1-P_4	2.296(1)	P_4-Pd_1-C_2	98.31(3)
Pd_1-C_2	1.983(1)	C1_3-Pd_1-C3_3	61.94(4)
Pd_1-C1_3	2.239(1)	P_4-Pd_1-C3_3	99.11(3)
Pd_1-C3_3	2.189(1)	C_2-Pd_1-C1_3	100.32(4)
Pd_1-C2_3	2.228(1)	HA[b]	14.21(10)
C=C_Indenyl[a]	1.415(2)	FA[b]	12.27(9)
ΔM···C[b]	0.298(2)	Pd-Indenyl Ave Plane[d]	87.07(2)
Pd_1···ClO_4^-	4.766(1)		

[a]Average C1=C2=C3 bond length in indenyl ligand
[b]As defined in Table 1 footnotes of Zargarian Coordination Chemistry Reviews 233-234 (2002) 157-176
[c]Shortest distance between palladium centre and anion
[d]Average angle among the mean metal coordination plane and the mean indenyl plane

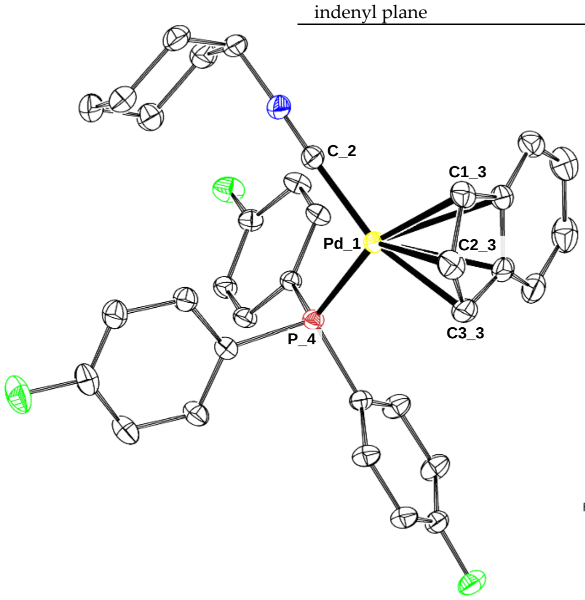

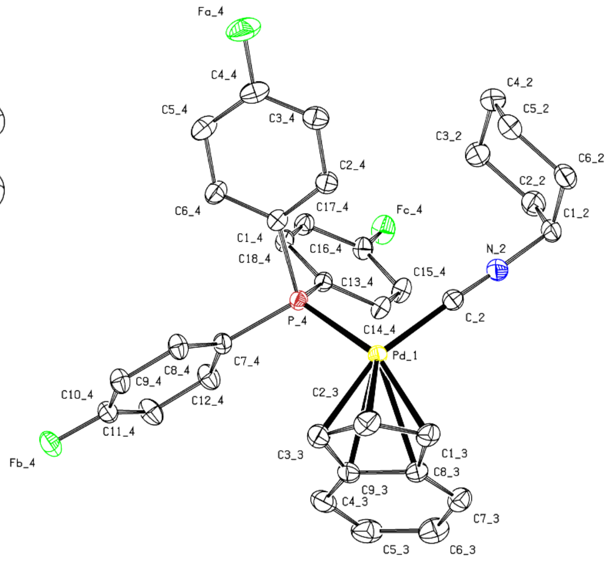

*Table S 9. Selected palladium distances and angles for **3d-Cyic** at 100 K and 298 K*

3d-Cyic - [$PdC_{41}H_{43}NO_3P$](ClO_4)							
ASU@100 K				ASU@298 K			
**Distances**	(Å)	**Angles**	**(°)**	**Distances**	(Å)	**Angles**	**(°)**
Pd_1-P_4	2.298(1)	P_4-Pd_1-C_2	94.63(5)	Pd_1-P_4	2.296(1)	P_4-Pd_1-C_2	94.45(6)
Pd_1-C_2	1.976(2)	C1_3-Pd_1-C3_3	61.68(7)	Pd_1-C_2	1.972(2)	C1_3-Pd_1-C3_3	61.38(9)
Pd_1-C1_3	2.268(2)	P_4-Pd_1-C3_3	102.39(5)	Pd_1-C1_3	2.263(2)	P_4-Pd_1-C3_3	103.10(6)
Pd_1-C3_3	2.195(2)	C_2-Pd_1-C1_3	101.17(7)	Pd_1-C3_3	2.194(3)	C_2-Pd_1-C1_3	100.76(8)
Pd_1-C2_3	2.223(2)	HA[b]	13.81(16)	Pd_1-C2_3	2.220(3)	HA[b]	13.62(19)
C=C_Indenyl[a]	1.414(3)	FA[b]	13.51(16)	C=C_Indenyl[a]	1.410(4)	FA[b]	12.95(17)
ΔM···C[b]	0.311(4)	Pd-Indenyl Ave Plane[d]	85.78(3)	ΔM···C[b]	0.318(4)	Pd-Indenyl Ave Plane[d]	85.01(4)
Pd_1···ClO_4^-	4.539(7)			Pd_1···ClO_4^-	3.86(2)		

[a]Average C1=C2=C3 bond length in indenyl ligand; [b]As defined in Table 1 footnotes of Zargarian Coordination Chemistry Reviews 233-234 (2002) 157-176; [c]Shortest distance between palladium centre and anion; [d]Average angle among the mean metal coordination plane and the mean indenyl plane; [*]Enantiomorphic packing

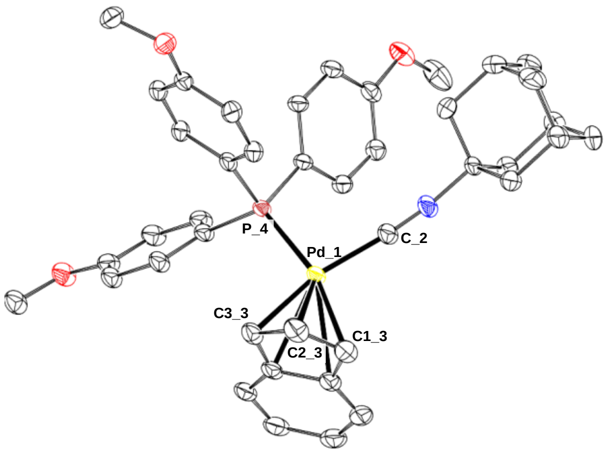

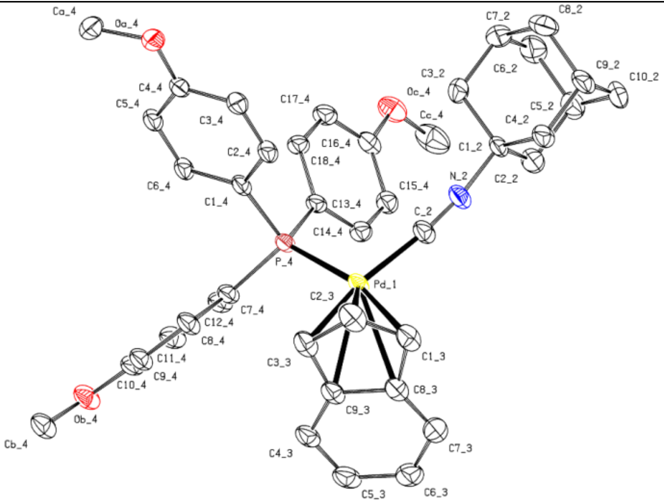

*Table S 10. Selected palladium distances and angles for **EB6x** at 100 K and 298 K*

3a-Cyic -[$PdC_{34}H_{33}NP$](ClO_4)							
ASU@100 K				ASU@298 K			
**Distances**	(Å)	**Angles**	(°)	**Distances**	(Å)	**Angles**	(°)
Pd_1-P_4	2.278(1)	P_4-Pd_1-C_2	94.8(1)	Pd_1-P_4	2.285(1)	P_4-Pd_1-C_2	95.09(5)
Pd_1-C_2	1.974(3)	C1_3-Pd_1-C3_3	62.2(2)	Pd_1-C_2	1.977(2)	C1_3-Pd_1-C3_3	61.46(8)
Pd_1-C1_3	2.251(6)	P_4-Pd_1-C3_3	99.6(1)	Pd_1-C1_3	2.248(2)	P_4-Pd_1-C3_3	100.57(6)
Pd_1-C3_3	2.186(4)	C_2-Pd_1-C1_3	103.5(2)	Pd_1-C3_3	2.178(2)	C_2-Pd_1-C1_3	102.85(7)
Pd_1-C2_3	2.218(5)	HA[b]	13.77(38)	Pd_1-C2_3	2.217(2)	HA[b]	13.62(16)
C=C_Indenyl[a]	1.413(4)	FA[b]	10.28(31)	C=C_Indenyl[a]	1.404(4)	FA[b]	10.36(5)
ΔM···C[b]	0.295(10)	Pd-Indenyl Ave Plane[d]	86.68(8)	ΔM···C[b]	0.304(4)	Pd-Indenyl Ave Plane[d]	86.80(3)
Pd_1···ClO_4^-	4.730(3)			Pd_1···ClO_4^-	4.793(3)		

[a]Average C1=C2=C3 bond length in indenyl ligand; [b]As defined in Table 1 footnotes of Zargarian Coordination Chemistry Reviews 233-234 (2002) 157-176; [c]Shortest distance between palladium centre and anion; [d]Average angle among the mean metal coordination plane and the mean indenyl plane; [*]Enantiomorphic packing

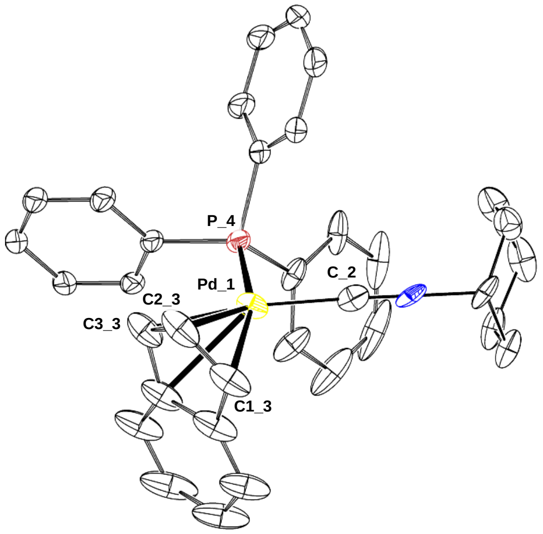

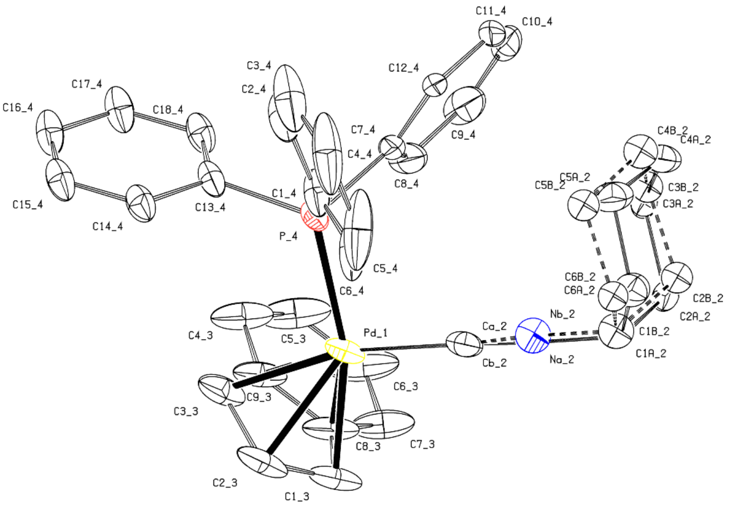

X-ray diffraction analysis - Structural characterization

3a-Tic, **3c-Tic**, **3d-Tic**, **3b-Tic**, **3c-Cyic**, **3d-Adic** and **3a-Cyic** molecules have been crystallized, characterized through XRD and show square planar Palladium coordination spheres (Tables S4-S10). All the crystalline forms bear one crystallographically independent palladium complex each, while **3a-Tic** show 2 molecules in the asymmetric unit (Figure S 82). Each complex bear a perchlorate counterion that balance the palladium centre net positive charge, with a Pd···O distance of ~4.0 Å. Conformations of **3a-Tic**, **3c-Tic**, **3d-Tic**, **3b-Tic**, **3c-Cyic** and **3a-Cyic** are well superimposable with limited phenyl sidechains rotations; minor phosphine rearrangements are found with the bulkier adamantly isocyanide in **3d-Adic** (Figure S 83) – R.M.S.D. < 1 Å among common atoms).

Indenyl ligand is almost perpendicular to the palladium coordination plane with equivalent hinge and fold angles and "slippage" of the ligand (compared to an idealised pentahapto coordination), as measured with HA, FA and ΔM···C parameters [1] respectively (Tables S4-S10).

Crystal packing show hydrophobic contacts among neighbour molecules, involving weak intermolecular π···π and CH···π interactions, among neighbour aromatic rings. Solvent molecules have been found in the crystal packing of **3a-Tic**. Chloroform molecules are bound to perchlorate oxygen atoms in anions through polar contacts (shortest $d_{CH\cdots O}$ = 3.165(5) Å). No phase transitions have been found for samples characterized at 100K and 298 K.

*S 82. Ortep representations of asymmetric unit contents for **3a-Tic** at 100 K (A), **3c-Tic** at 100 K (B), **3d-Tic** at 100 K (C), **3d-Tic** at 100 K (enantiomorphic packing - D), **3b-Tic** at 298 K (E), **3c-Cyic** at 100 K (F), **3d-Adic** at 100 K (G), **3d-Adic** at 298 K (H), **3a-Cyic** at 100 K (I) and **3a-Cyic** at 298 K (L). Ellipsoids dimensions correspond to 50% probability.*

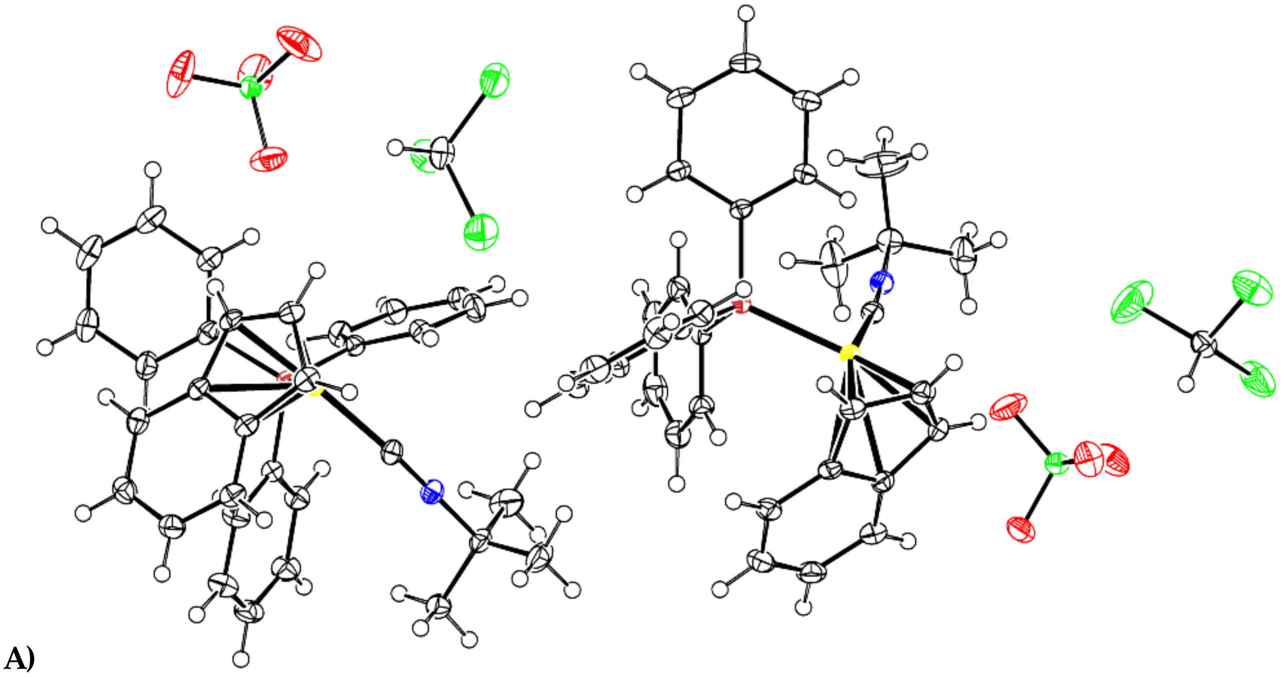

A)

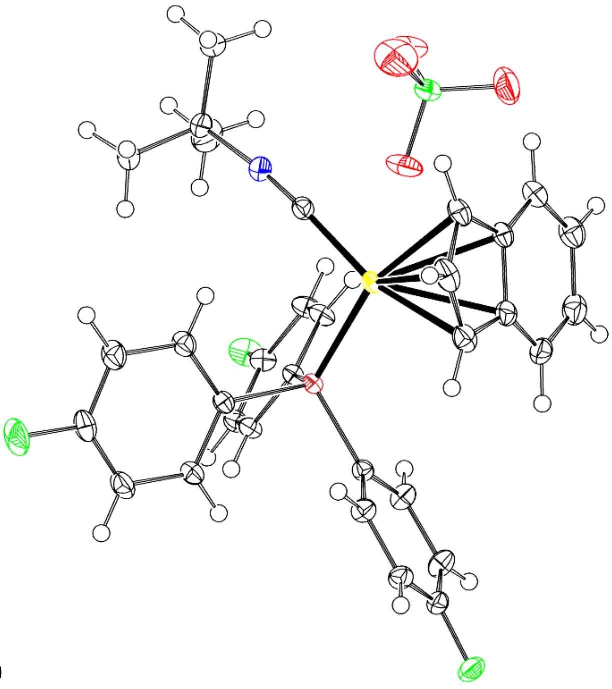

B)

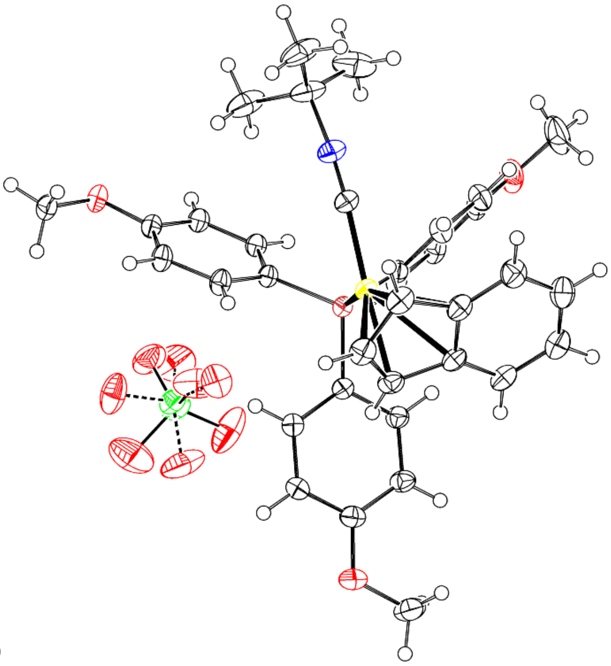

C)

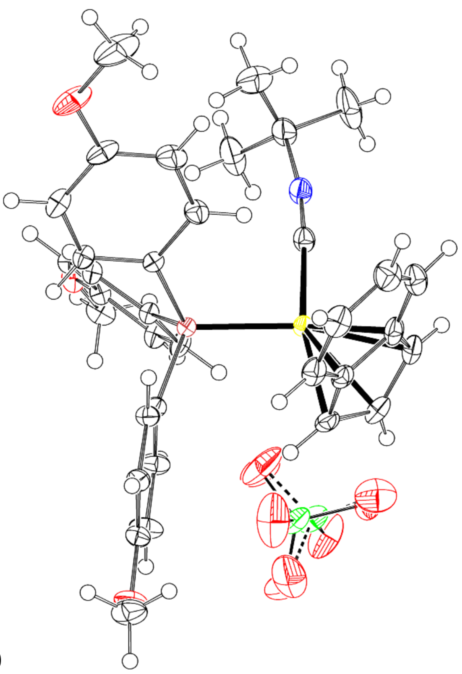

D)

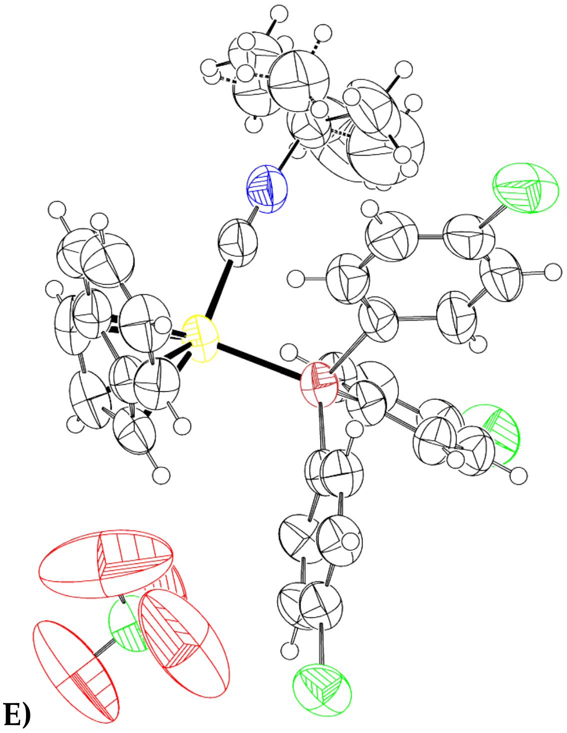

E)

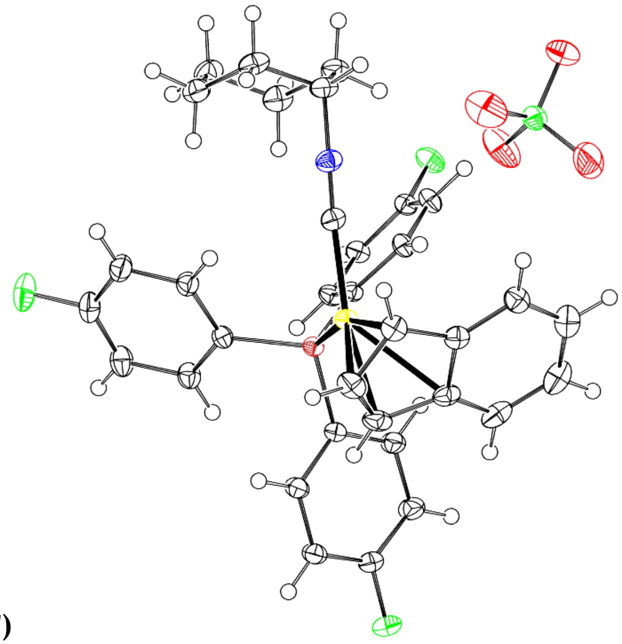

F)

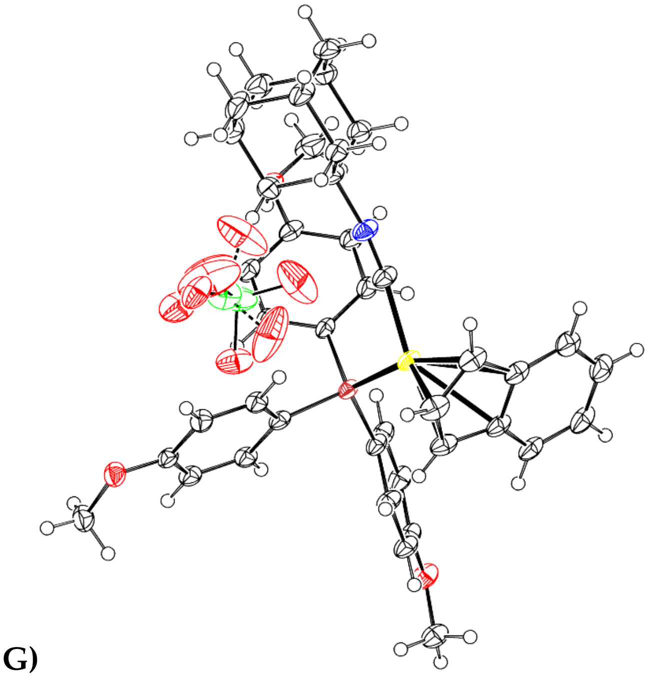

G)

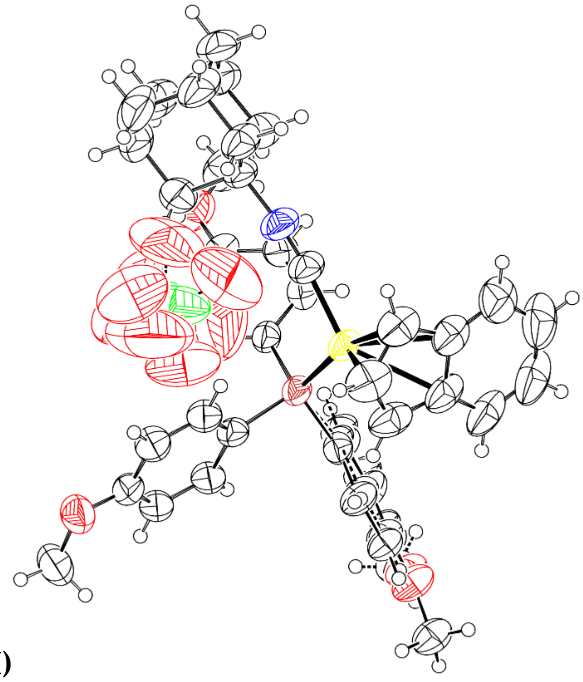

H)

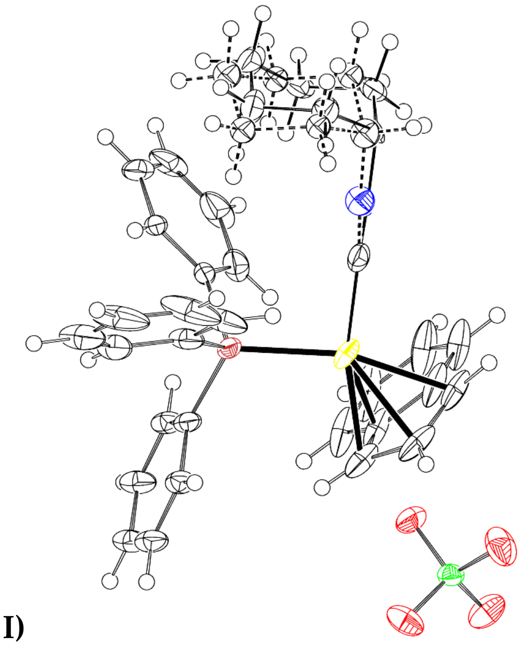

I)

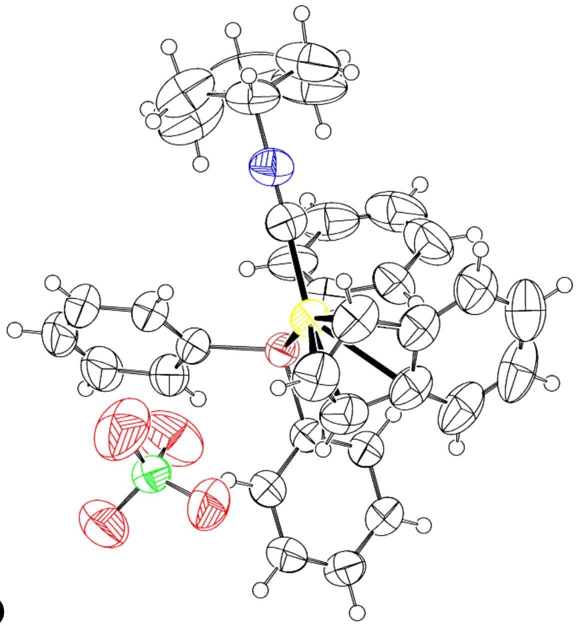

L)

*S 83. Overlap of crystallographically independent molecules bearing tert-butyl isocyanide complexes (**3a-Tic**, **3c-Tic**, **3d-Tic**, **3b-Tic** - yellow sticks), 1-adamantyl isocyanide complexes (**3d-Adic** – blue sticks) and cyclohexyl isocyanide complexes (**3c-Cyic**, **3a-Cyic** - pink sticks). Hydrogens omitted for clarity*

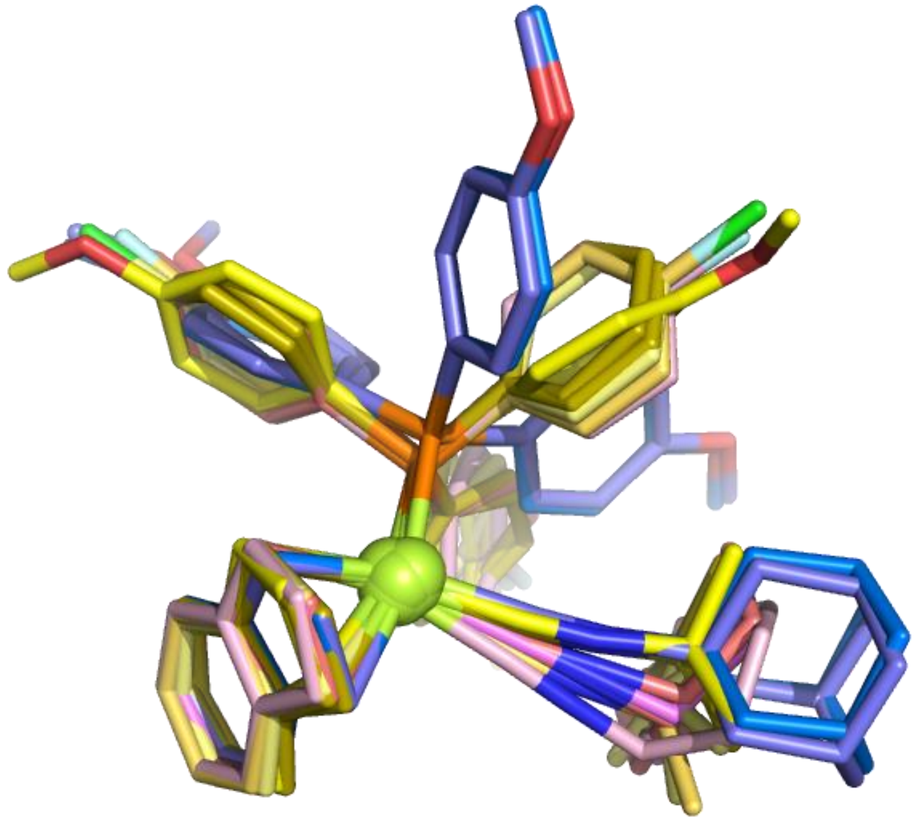

References

1. Zargarian, D. Group 10 Metal Indenyl Complexes. *Coord. Chem. Rev.* **2002**, *233–234*, 157–176, doi:10.1016/S0010-8545(02)00201-1.
